# Proteoform analysis by mass spectrometry reveals post-translational processing of legumins and vicilins in chickpeas (*Cicer arietinum* L.)

**DOI:** 10.1007/s00216-026-06577-0

**Published:** 2026-05-29

**Authors:** Antonella Di Francesco, Aldo Lanzoni, Maria Gaetana Giovanna Pittalà, Rosaria Saletti, Ole N. Jensen, Vincenzo Cunsolo

**Affiliations:** 1 Laboratory of Organic Mass Spectrometry (LOMS), Department of Chemical Sciences, University of Catania, Catania, Italy; 2 Department of Biochemistry and Molecular Biology, University of Southern Denmark, Odense, Denmark

**Keywords:** Chickpea proteins proteolysis, Legumins and vicilins, N-Glycosylated plant proteins, Orbitrap® mass spectrometry, Bottom-up and top-down approaches

## Abstract

The most abundant proteins in pulse seed, including chickpea (*Cicer arietinum *L.), are the 7S globulins (vicilins) and 11S globulins (legumins), which play key roles in plant reproduction and human nutrition and are also recognized as potential allergens. During seed maturation, legumins and vicilins undergo limited proteolysis. Legumins are processed into α- and β-polypeptide chains that remain linked by a disulfide bond, whereas vicilins are cleaved at two sites, generating three lower-molecular-mass polypeptides (α-, β-, and γ-chains). An integrated mass spectrometry approach, combining bottom-up and top-down strategies, was applied to characterize the post-translational processing of legumins and vicilins. Our data suggest that (i) proteolytic cleavages in both legumins and vicilins occur at conserved sequence motifs; (ii) legumin processing is catalyzed by an asparaginyl endopeptidase, whereas vicilin processing is mediated by legumains, a family of cysteine endopeptidases; and (iii) one chickpea vicilin is N-glycosylated and carries a high-mannose oligosaccharide side chain. Limited proteolysis process represents a key physiological mechanism regulating storage protein assembly, stability, and mobilization during seed development and germination while preserving storage functions. Glycosylation of vicilins further modulates their structural and functional properties, although it may also influence digestibility and allergenic potential. The mass spectrometry data have been deposited with the ProteomeXchange with the identifier <PXD078377>.

**Supplementary Information:**

The online version contains supplementary material available at 10.1007/s00216-026-06577-0.

## Introduction

The nutritional importance of pulse crops (e.g., chickpea, lentil, cowpea, and green pea) in the diet of millions of people is constantly growing, especially in developing countries, because they are an excellent source of minerals, proteins, fats, fibers, and carbohydrates [1–4]. Although lacking some essential amino acids, such as methionine and cysteine, pulse crops contain 20–40% of protein by weight, similar to that of animal-derived foods, and much higher than in cereals. Moreover, they play a significant role in both gluten-free regimes and in the diet of vegetarians, since the other food items they consume do not contain much protein [5]. Among pulses, chickpea (*Cicer arietinum*) is considered one of the most popular legume crops worldwide [6] because of its multiple uses and potential health benefits, which include reducing cardiovascular, diabetic, and cancer risks [7]. Like other legumes, the protein fraction of chickpea seed is mainly constituted by the globulin storage proteins, which represent approximately 70% of the total protein fraction and play a fundamental role in plant reproduction and human nutrition [8]. Developing legume seeds accumulate large amounts of globulin storage proteins that serve as carbon and nitrogen reserves during germination and early seedling growth [9]. These proteins are stored in aggregated form within specialized seed organelles known as protein bodies [10]. Globulins comprise two main protein groups that, based on their sedimentation coefficients (*S* = Svedberg unit) [11], can be subdivided into 7S-globulins, usually known as vicilins, and the 11S-type globulins, the legumins. Legumins and vicilins have been found to resist both in vivo and in vitro digestion and have been recognized as chickpea allergens [12, 13]. Both globulin classes represent a mixture of components that exhibit a discrete polymorphism arising not only from the presence of multigene families but also from some post-translational processes, which include proteolytic processing and/or glycosylation [14, 15]. Legumins are first synthesized as a large precursor, the pre-prolegumin, which, after the removal of the signal peptide, leads to a prolegumin, with mass (m) in the range of 50–60 kDa. Then, according to the current understanding of this process, prolegumin aggregates into trimers in endoplasmic reticulum, and then the trimers move to the vacuolar protein bodies where a protease cleaves them into two polypeptide chains, the acidic α-chain with a molecular mass of 32–40 kDa, and the basic β-chain of 20 kDa. Both chains remain linked together by a disulfide bridge between cysteine residues located at highly conserved regions of the α- and β-chains. The peptide bond joining the α- and β-chains of the pro-proteins is located at a highly conserved region and always involves, as the C-terminal amino acid of the α-chain, an asparagine residue. The N-terminal residue of the β-chain is almost always a glycine [14]. The presence of the asparagine at the C-terminus of the large hydrophilic α-chain plays a fundamental role in the recognition by the corresponding processing enzyme because mutation or deletion of this residue renders prolegumin uncleavable in vitro as well as in vivo [16, 17]. Finally, six paired polypeptide chains interact non-covalently to assemble the final hexameric form of legumins (300–400 kDa) [8]. Vicilins, as mature polypeptides, usually show a molecular mass between 45 and 65 kDa and are reported as trimeric proteins of mass ~ 150 to 190 kDa. Unlike legumins, vicilins from other Fabaceae appear sparsely glycosylated, with one or two *N*-linked glycosylation sites located in the C-terminal domain [18–20]. Although always described as proteins lacking cysteine residues [7], a recent MS-based investigation demonstrated that some chickpea vicilins contain this amino acid residue [21]. Some studies demonstrated that vicilins from diverse pulses also exhibited extensive proteolytic processing. For instance, in developing pea seeds, vicilins contain up to two potential sites for proteolytic cleavage, that generate three lower-mass polypeptides, classified as α- (m 20–22 kDa), from the N-terminal, β- (m 13–15 kDa), from the central part of the precursor, and γ-chain (m 12–15 kDa), containing the C-terminus. SDS-PAGE bands probably related to the α + β subunits (33–37 kDa) and β + γ subunits (25–30 kDa) have also been detected [22, 23]. Our recent work [21] showed that in mature chickpea seeds, legumins and vicilins undergo a proteolytic process that produces different lower-mass polypeptides, but a detailed sequence characterization of these proteolytic products is lacking. Here, we report an integrated top-down and bottom-up MS/MS approach aimed, for the first time, at the sequence characterization of the lower-mass products of legumins and vicilins detected in mature chickpea seeds of the Italian genotype “Pascià.”

## Materials and methods

### Chemicals

All chemicals were used without further purification because of the highest purity commercially available. Porcine trypsin was purchased from Promega (Madison, WI, USA). Ammonium bicarbonate (AMBIC), cOmplete Protease Inhibitor Cocktail (Hoffmann-La Roche, Basel, CH), 1,4-dithiothreitol (DTT), iodoacetamide (IAA), Tris-HCl, EDTA, formic acid (FA), boric acid, glacial acetic acid, sodium chloride, hexane, and other reagents were analytical grade and were purchased from Sigma Aldrich (St. Louis, Missouri, USA). Water and acetonitrile (ACN) were OPTIMA® LC/MS grade and were purchased from Fischer Scientific (Milan, Italy). C18 and C4 resins for column packing were purchased from Dr. Maisch HPLC GmbH (Beim Brückle Ammerbuch Entringen, Germany).

### Chickpea sample collection and defatting

The chickpea sample, genotype Pascià, was provided, as a mix of three biological replicates, by the Department of Agriculture, Food, Natural Resources and Engineering (DAFNE) of the University of Foggia (Italy). Chickpea seeds were ground into powder, and then the flour obtained was defatted using hexane of analytical grade to eliminate the lipid fraction from the chickpea flour by solvent extraction at a ratio of 1:3 weight: volume (flour: solvent) for 1 h under stirring at 200 rpm. Subsequently, the mixture was filtered and dried under an extractor hood at room temperature for 24 h. The defatting process of the chickpea flour was repeated twice.

### Protein extraction of the legumin and the vicilin-enriched fractions

**Fig. 1:**
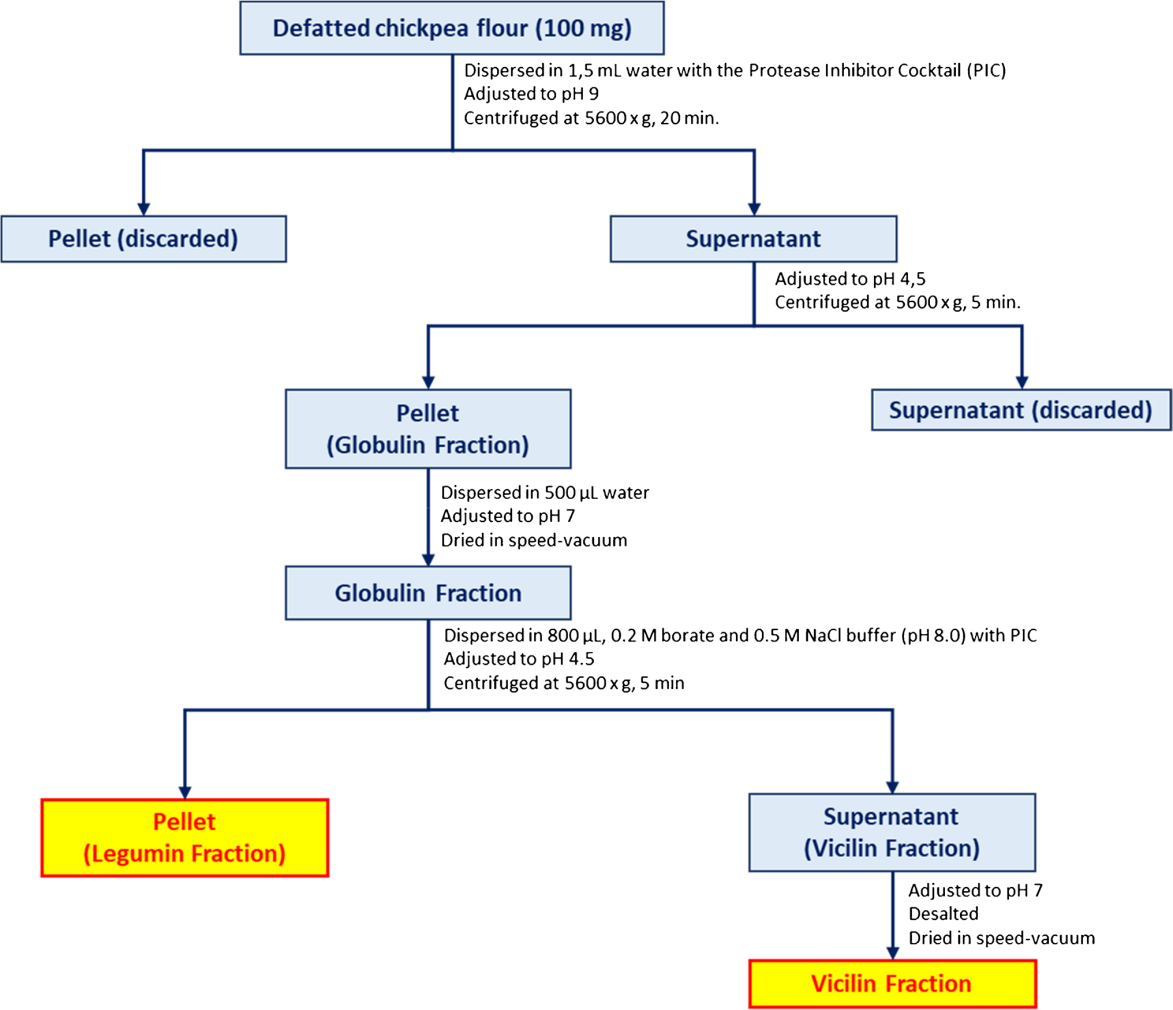
Flowchart for preparation of the enriched vicilins and legumins fractions from the defatted chickpea flour

The legumin and vicilin-enriched fractions were extracted from the defatted chickpea flour as reported by Chang et al. [24] with some modifications, as depicted in Fig. 1. The extraction was carried out in the presence of cOmplete Protease Inhibitor Cocktail, aimed to protect proteins from aminopeptidases, metalloproteases, and serine, cysteine, and aspartic acid proteases. Particularly, 100 mg of defatted pulse flour was dispersed in 1.5 mL of water, containing 60 µL of 25X stock solution of cOmplete Protease Inhibitor Cocktail. Then, the pH was adjusted to 9.0, using 1.0 M NaOH. The solution was stirred at room temperature for 2 h and then centrifuged at 5600 × g for 20 min at 4 °C. The residual pellet was discarded, whereas the pH of the supernatant solution was adjusted to 4.5 using 1.0 M HCl to precipitate globulin proteins. This globulin pellet was separated from the supernatant by centrifugation at 5600 × g for 5 min at 4 °C and re-suspended in 500 µL water by stirring for 2 h at room temperature. The pH of the globulin protein solution was neutralized to 7.0 using 0.1 M NaOH, and finally, globulin protein fraction powder was obtained by drying in a speed-vacuum (SpeedVac SPD1030 System, Thermo Fisher Scientific, Waltham, USA). The separation of legumin and vicilin fractions from the extracted globulin pellet was obtained as already reported [25, 26] with some differences. Particularly, the globulin pellet was dispersed in 800 µL of 0.2 M sodium borate buffer (pH 8.0) containing 0.5 M NaCl, and 60 µL of 25X stock solution of cOmplete Protease Inhibitor Cocktail. Afterwards, the solution was stirred for 2 h at room temperature. Then, the pH solution was adjusted to 4.5 using 6 M of glacial acetic acid to precipitate the legumin fraction. After centrifugation at 5600 × g for 5 min at 4 °C, a pellet mainly consisting of legumins was obtained and separated from the supernatant solution, mostly constituted by vicilins. The pH of this supernatant solution was adjusted to 7.0 using 1.0 M NaOH and desalted by Zeba™ Spin Desalting Columns 7 K MWCO (Thermo Fisher Scientific™) to remove borate buffer and salts. The pellet, containing the legumin fraction, was dissolved in a solution with a pH of 7.0. The protein concentration of legumins and vicilins fractions was determined using the Qubit Protein Assay kit and the Qubit® 1.0 Fluorometer (ThermoFisher Scientific, Milan, Italy) [27]. Finally, both protein fractions were dried in a speed vacuum and stored at −80 °C until use.

### Protein in-solution hydrolysis

An aliquot corresponding to 50 µg of both enriched-protein fractions of vicilins and legumins was suspended in bicarbonate buffer (50 mM, pH 8), reduced, and alkylated as previously reported by Di Francesco et al. [28]. Particularly, about 39 µg of DTT (3 h at 20 °C) and then 94 µg of IAA (1 h in the dark at 20 °C) were used for the reduction and alkylation, respectively. Then, the solution was split into two aliquots for both shotgun and top-down MS analyses. The aliquot for shotgun analysis was digested by porcine trypsin (Sequencing Grade Modified Trypsin, Porcine, lyophilized, Promega) at an enzyme-substrate ratio of 1:50 (overnight, 37 °C). To achieve a final concentration of 25 ng/µL, a 2% aqueous solution of FA was added, resulting in a final volume of 2 mL. The aliquot for top-down analysis was dried in a speed vacuum and finally suspended in 2% FA for top-down MS.

### Mass spectrometry analyses

Survey scans of analytes (i.e., peptides and proteins) and MS data were acquired using Xcalibur v. 4.6 software (Thermo Fisher Scientific). The calibration of the MS systems was performed by using the Pierce FlexMix Calibration Solution (Thermo Fisher Scientific). The deconvolution of the multi-charged ESI mass spectra of the proteins was obtained using the Xtract® deconvolution algorithm (Thermo Scientific).

### LC–MS/MS analysis of protein digests

LC–MS/MS analyses of the peptide mixtures were carried out in triplicate on a ThermoFisher Scientific Orbitrap Lumos Tribrid® mass spectrometer (ThermoFisher Scientific, Bremen, Germany) coupled with a ThermoFisher Scientific EASY-nLC 1000 system (Sunnyvale, CA) liquid chromatography. A total of 5 µL (corresponding to 125 ng) of the peptide mixture were loaded into a homemade pre-column packed with ReproSil-Pur 120 C18-AQ, (75 µm i. d. × 5 cm, 5 µm particle size, 120 Å) [29] and after separated by a homemade analytical-column packed with Reprosil-Pur 120 C18-AQ (75 µm i.d. × 24 cm, 3 µm particle size, 120 Å) [29]. Elution was carried out at a flow rate of 300 nL/min by a linear gradient of solvent B (ACN + 0.1%FA) in the solvent A (H_2_O + 0.1%FA), 2% to 28% in 55 min, 28% to 40% in 22 min, 40% to 100% in 1 min and finished by holding 100% of solvent B for 7 min, and 100% to 2% in 1 min and re-equilibrating the column at 2% of solvent B for 3 min. Peptides were mass analyzed and sequenced by electrospray ionization using a source voltage of 2.2 kV and introduced into the mass spectrometer through a heated ion transfer tube (275 °C). Survey scans of peptide precursors from 200 to 1700 *m*/*z* were performed at 120 K resolution (@ 200 m/z). Tandem MS was performed by isolation at 1.6 m/z with the quadrupole, HCD fragmentation with a normalized collision energy of 30, and high-resolution MS analysis of fragments in the Orbitrap (50 K resolution). Only those precursors with charge states 2–4 and intensity above the threshold of 5000 were sampled for MS2. The dynamic exclusion duration was set to 20 s with a 10 ppm tolerance around the selected precursor and its isotopes. Monoisotopic precursor election was turned on. The instrument was running top speed mode with 3-s cycles, meaning it would continuously perform MS2 events until the list of non-excluded precursors diminished to zero or 3 s, whichever is shorter.

### Top-down LC–MS/MS analysis

Top-down mass spectrometry analysis of the enriched vicilin protein fraction was carried out, for the structural characterization of the proteoforms, in triplicate on a Thermo Fisher Scientific Orbitrap Fusion Lumos mass spectrometer (Thermo Fisher Scientific, Bremen, Germany) coupled online with the Thermo Scientific EASY-nLC 1000 nanosystem (Sunnyvale, CA, USA). In detail, 2 µL (corresponding to 500 ng of protein mixture) of the reduced and alkylated vicilin-enriched fraction was loaded onto a homemade C4 column packed with ReproSil-Pur 100 C4 (100 µm i.d. × 25 cm, 1.8 µm particle size, 100 Å) [29]. The proteins were separated by elution at a flow rate of 250 nL/min at room temperature by a linear gradient of solvent B (ACN + 0.1%FA) in solvent A (H_2_O + 0.1%FA), 10% for 1 min, followed by 10% to 70% in 40 min, 70% to 100% in 5 min. We finished by holding 100% B for 5 min, 100% to 10% in 1 min, and re-equilibrating the column at 2% B for 20 min. Proteins were analyzed by electrospray ionization using a source voltage of 2.2 kV and finally introduced into the mass spectrometer through a heated ion transfer tube (275 °C). Mass spectra of all the precursor ions were acquired at 120 K resolution at *m*/*z* 200, in the range *m*/*z* 200–1800. MS/MS analyses were performed by CID fragmentation with a normalized collision energy of 30, and the MS fragment ions were analyzed in the Orbitrap (high-resolution MS/MS analysis) at 60 K resolution in cycle time mode set to 3 s. Top-down analyses aimed at investigating the *N*-glycosylation of vicilins (performed in duplicate) and the structural characterization of legumin proteoforms (performed in triplicate) were carried out using an Orbitrap Fusion Tribrid (ThermoFisher Scientific, Bremen, Germany) coupled online with the UHPLC ThermoFisher Scientific Dionex UltiMate 3000 RSLCnano system (Sunnyvale, CA). In detail, 1 µL of the reduced and alkylated protein fraction, corresponding to 5 ng, was injected into an Easy-Spray PepMap HPLC capillary column C4 (1500 Å, 4 µm, 150 µm × 150 mm). The proteins were separated by elution at a flow rate of 800 nL/min, at 40 °C, by a linear gradient of solvent B (ACN + 0.1%FA) in the solvent A (H_2_O + 0.1%FA), from 15 to 65% in 82 min, followed by 65% to 95% in 5 min, 95% for 10 min, and then the column was eluted with solvent B from 95 to 15% in 5 min and finally equilibrated at 15% B for 10 min. Eluted proteins were converted to gas-phase by electrospray ionization, source voltage 1.3 kV, and introduced into the mass spectrometer through a heated (275 °C) ion transfer tube. Mass spectra of all the precursor ions were acquired in a range from 200 to 1800 *m*/*z* at a resolution of 120 K (@ 200 *m*/*z*) using the following parameters: RF lens, 80%; auto gain control, 200000; maximum injection time, 100 ms. Top-down MS/MS (TD-MS2) analyses were performed by HCD fragmentation at a normalized collision energy of 15, 22, and 25, and by CID fragmentation at a normalized collision energy of 30. MS fragment ions were analyzed in the Orbitrap (high-resolution MS/MS analysis) at 120 K resolution in cycle time mode set to 3 s.

### Data analysis

Mass spectrometry data and protein sequence analysis were performed using the General Protein Mass Analysis for Windows 9.5 (GPMAW) software (Lighthouse data, Odense, Denmark).

### Shotgun data analysis

The peptide-level LC-/MSMS data were processed by PEAKS Xpro de novo sequencing software (Bioinformatics Solutions Inc., Waterloo, ON, Canada; https://www.bioinfor.com//), which provides automated and accurate de novo peptide sequencing. The de novo amino acid sequences generated by PEAKS were searched against a custom protein database, including only the 18 legumins and vicilins entries of *Cicer arietinum* L. (chickpeas) downloaded from the UniProt database (release May 2025). The database search was carried out, setting trypsin as a proteolytic enzyme, semispecific as the digestion mode, and 3 as the maximum number of allowed missed cleavages. In the first step of the database search, carbamidomethylation of cysteines was set as a fixed modification, whereas the oxidation of methionine, the acetylation (N-terminal protein), formylation, and the transformation of N-terminal glutamine and N-terminal glutamic acid residue into the pyroglutamic acid form were considered variable amino acid modifications. The de novo amino acid sequences unmatched during the first round of the database search were searched, enabling the PEAKS PTM tool to identify as additional modifications the methionine di-oxidation, cysteine di-oxidation, and cysteine trioxidation. Database search was carried out, setting the precursor mass tolerance threshold at 10 ppm and the max fragment mass error at 0.006 Da. Peptide Spectral Matches (PSMs) were validated using a Target Decoy PSM Validator node based on *q*-values at a false discovery rate (FDR) ≤ 1%. PEAKS score thresholds for PSMs were set to achieve FDR values below 1% for PSMs, peptide sequences, and proteins identified from each database search. For both samples analyzed using the shotgun approach, the relative abundance of each identified protein was determined. This value was calculated from the raw “Sample Area” values reported in the PEAKS output for each protein. In the PEAKS software, the protein “Sample Area” is defined as the sum of all peptide features derived from unique supporting peptides. For each LC–MS run, the relative abundance of each protein was then expressed as a percentage by normalizing its “Sample Area” value to the total area of all quantified proteins.

### Top-down data analysis

The full scan MS and MS/MS data obtained from the top-down analyses were initially processed by the MASH Native software in “Discovery mode” (Ge Research Group, University of Wisconsin-Madison) and searched against a protein database including all the reviewed and unreviewed entries of *Cicer arietinum* L. (chickpea) of the UniProt database (Swiss-Prot and TrEMBL sections, release February 2023, 31239 entries). This preliminary database search aimed to find what kind of proteins were present in the sample under investigation. The database searches were carried out using the TopFD as a deconvolution algorithm and the TopPIC as a Database Search algorithm. The number of unexpected PTMs was set to 1, and the precursor mass tolerance threshold was set to 15 ppm, whereas the max fragment mass error was set to 0.020 Da. Then, to validate the results, a Decoy Database Searching was carried out, with a spectrum cutoff value based on *E*-value set to 0.01. Finally, a freely available tool coined ClipsMS (Comprehensive Localization of Internal Protein Sequences) (https://github.com/loolab2020/ClipsMS-Version-1.0.0) [30], and developed to assign both terminal and internal fragments resulting from a top-down mass spectrometry experiment, was used to in-depth characterize the sequence of each polypeptide detected and identified in the preliminary database search. In detail, the raw MS/MS spectra obtained by CID fragmentation of polypeptides were first deconvoluted using the Xtract® deconvolution algorithm (Thermo Scientific); then, every deconvoluted mass list was uploaded into the ClipsMS program together with the selected sequence entry to obtain a matched fragments list. The error for fragment matching was set at 10 ppm, and the smallest internal fragment size was set at 5 amino acids. All the terminal fragments were assigned before considering internal fragments. Then, only *by* internal fragments were searched for and used as putative candidates of uninterpreted signals.

The possible existence of many theoretical fragments with overlapping masses introduces a high risk of false-positive annotation. To prevent false-positive assignments, after the matching process, all putative *by* internal fragment assignments were manually verified, and the following criteria were applied: (i) the putative internal fragments were real peaks rather than noise or isotopes; (ii) when both a terminal and an internal fragment matched the same peak, the terminal fragment was the preferential assignment; (iii) when two terminal fragments matched the same peak, the internal fragment with the lower mass error was selected; (iv) when two terminal fragments matched the same peak with identical mass error, both assignments were reported; however, whenever possible, preference was given to cleavages occurring N-terminal to proline and C-terminal to acidic residues. The last criterion was adopted because it is well known that in the fragmentation of peptides and whole proteins, fragment ions produced N-terminal to proline (proline effect) [31] and cleavage C-terminal to acidic residues, mainly aspartic acid, are over-represented in the MS/MS spectra. The detailed interpretation and comments about the internal fragments are reported in the Supplementary Material.

## Result and discussion

**Fig. 2:**
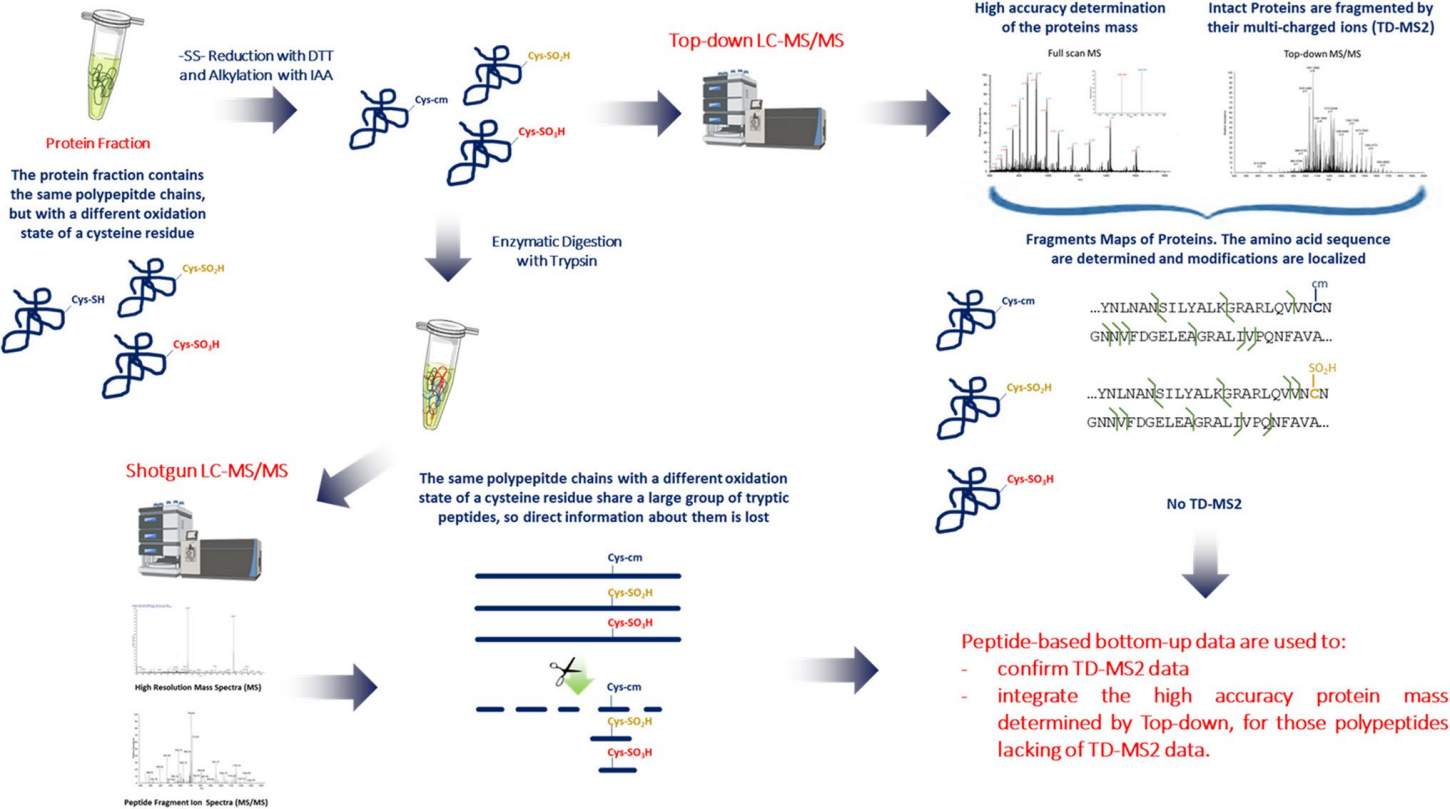
Workflow applied for the structural characterization of the polypeptides generated by specific post-translational cleavages of legumins and vicilins, occurring during maturation of seed chickpeas. It is reported, for example, how, by integrating the top-down and the peptide-based bottom-up data, three polypeptides can be characterized sharing the same amino acid sequence but showing different oxidation states of a cysteine residue

The legumin and vicilin-enriched fractions were obtained as reported in the Materials and Methods section, and both the fractions were investigated by the experimental workflow depicted in Fig. 2.

### Shotgun MS analysis of the legumin-enriched fraction

The peptide-based bottom-up proteomics analysis of the legumin-enriched fraction identified 12 proteins, including five abundant legumins and seven less abundant vicilins, illustrating the ability to enrich for legumins (79%), however, with co-enrichment of vicilins (21%). The three most abundant legumins, constituting about 68% of the total amount of this protein fraction, correspond to the UniProtKB Accession Numbers (Acc. No.) A0A1S2XSB9 (relative abundance 35.2%), A0A1S2XVG1 (17.3%), and A0A3Q7XNW1 (15.2%). Protein identifications and matching peptides in the legumin-enriched fraction are listed in Supplementary Table S1.

### Top-down MS analysis of the legumin-enriched fraction

To characterize the amino acid sequence of the legumins, the reduced and IAA-alkylated legumin-enriched fraction was investigated by LC–MS analysis using a top-down approach for intact protein characterization. Initial processing of the MS data, carried out by the MASH Native software, allowed the identification of polypeptides with an experimental molecular mass of about 20 kDa, attributable to the C-terminal region of the three most abundant legumins identified by the shotgun approach, namely, the entries A0A1S2XSB9, A0A1S2XVG1, and A0A3Q7XNW1.

**Fig. 3:**
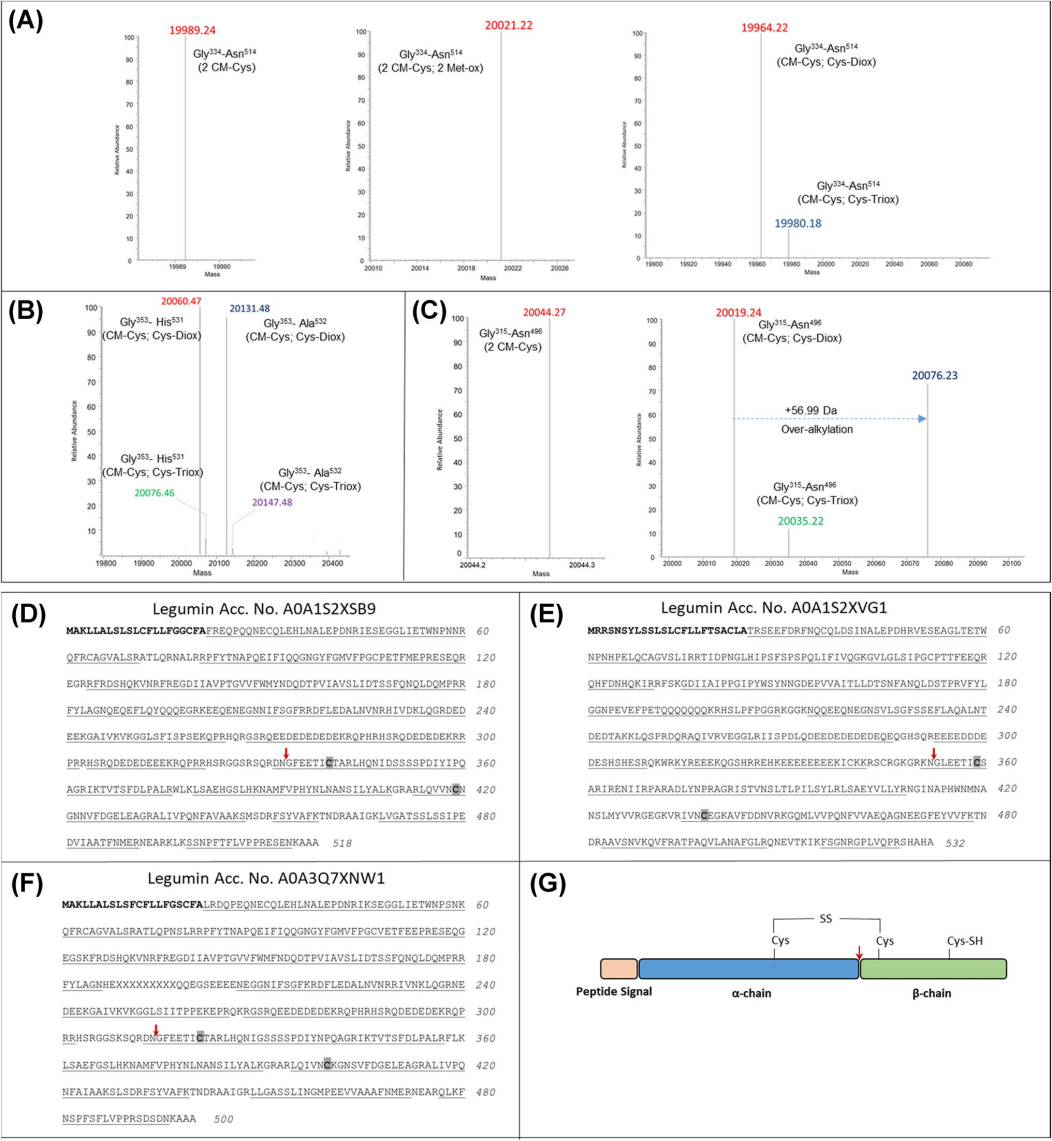
**A**, **B**, and **C** report the monoisotopic deconvoluted mass spectra (mass zero-charge) of the post-translational polypeptide products related to the legumins with Acc. No. A0A1S2XSB9, A0A1S2XVG1, and A0A3Q7XNW1, respectively. **D**, **E**, and **F** show the amino acid sequence of the legumins A0A1S2XSB9, A0A1S2XVG1, and A0A3Q7XNW1, respectively. Peptide signal, lacking in the mature form of the protein, is reported in bold. The sequence characterized via the shotgun approach is underlined. The X symbols, reported in the legumin entry A0A3Q7XNW1, refer to unknown amino acid residues. The cleavage site at the level of the Asn-Gly bond is indicated by a red arrow. The two cysteine residues of the β-chain are reported in italic bold and highlighted in grey. **G** shows the schematic picture of the post-translational proteolytic cleavage generating the α- and β-chains and the putative disulfide inter-chain bond which involves the cysteine located at the N-terminal region of the β-chains. The second cysteine residue of the β-chain, located in the C-terminal cupin domain and not involved in any disulfide bond, is reported with a free thiol group

Figure 3A shows the deconvoluted spectra of four polypeptides having, respectively, monoisotopic molecular masses of 20021.22, 19989.24, 19980.18, and 19964.22 Da (the corresponding multi-charged ESI mass spectra are reported in the Supplementary Figure S1).

The preliminary database search allowed us to relate these components to the amino acid region flanked between the glycine at position 334 and the asparagine at position 514 of the legumin A-like entry with the Acc. No. A0A1S2XSB9 (Fig. 3D). This amino acid region shows two cysteine residues, respectively localized at positions 340 and 419. An in-depth characterization by top-down-MS2 (TD-MS2) was achieved only for the polypeptides with *m*_mono_ 19989.24 and 19,964.22 Da (see the Supplementary Material for details). The component with *m*_mono_ 19,989.24 Da was identified as corresponding to the region Gly^334^-Asn^514^ with Cys^340^ and Cys^419^ in carbamidomethylated form (Fig. 3D; Figures S2 and S3, Table S2). The polypeptide with *m*_mono_ 19964.22 Da, related to the same amino acid region, shows Cys^340^ in carbamidomethylated form and Cys^419^ as sulfinic acid (Figures S4 and S5, Table S3). It is interesting to note that, by the shotgun approach, the cysteine at position 340 was always identified in the carbamidomethylated form. On the contrary, Cys^419^ in some peptides was identified in the di-oxidized form (i.e., as sulfinic acid, Cys-SO_2_H) (Figure S6). For the other two polypeptides with *m*_mono_ 20021.22 and 19980.18 Da, no TD-MS2 data were obtained. However, based upon the accurate intact mass information and taking into account the MS data obtained by the complementary shotgun approach, a putative identification can be obtained. The component with *m*_mono_ 20021.22 Da shows a mass difference of + 31.98 Da with respect to the mass of the polypeptide at 19989.24 Da. This mass shift is reasonably due to the mono-oxidation (+ 15.99 Da) of two out of three methionine residues included in the Gly^334^-Asn^514^ region (more details are reported in the Supplementary Material). Finally, the component with *m*_mono_ 19980.18 Da probably corresponds to the region Gly^334^-Asn^514^ carrying the Cys^340^ as carbamidomethyl-cysteine and the Cys^419^ as sulfonic acid. Indeed, by the shotgun analysis, the Cys^419^ was also identified in tri-oxidized form (i.e., as sulfonic acid, Cys-SO_3_H) as shown in Figure S8. The results of these polypeptides related to the legumin A-like entry with the Acc. No. A0A1S2XSB9 are summarized in Table 1.

**Table 1:** Top-down results about the polypeptide chains related to the Gly^334^-Asn^514^ region of the legumin A-like entry with the Acc. No. A0A1S2XSB9. Each polypeptide chain is reported: the amino acid region, the status of two cysteines at positions 340 and 419, the other detected modifications, the experimentally determined monoisotopic mass, the theoretical monoisotopic mass, and the error in ppm. The last two columns show whether the corresponding proteoform was supported by MS/MS data of the bottom-up and top-down approaches

	**AA region**	**Cys**^**340**^	**Cys**^**419**^	**Other Modif**	**Exp. *m***_**mono**_** (Da)**	**Theor. *m***_**mono**_** (Da)**	**Error (ppm)**	**MS/MS data by shotgun**	**MS/MS data by top-down**
1	Gly^334^-Asn^514^	CM ^a)^	CM	2 Met-ox ^b)^	20021.22	20021.20	0.7	X	
2	Gly^334^-Asn ^514^	CM	CM	-	19989.24	19989.21	1.2	X	X
3	Gly^334^-Asn ^514^	CM	di-ox ^c)^	-	19964.22	19964.20	1.4	X	X
4	Gly^334^-Asn ^514^	CM	tri-ox ^d)^	-	19980.18	19980.19	0.9	X	

^a)^*CM*, carbamidomethylation

^b)^*Met-ox*, methionine oxidized as methionine sulfoxide

^c)^*di-ox*, cysteine di-oxidized as sulfinic acid

^d)^*tri-ox*, cysteine tri-oxidized as sulfonic acid

Figure 3B shows the deconvoluted spectra of four co-eluting polypeptides. The two most abundant polypeptides show experimentally determined monoisotopic masses of 20060.47 and 20131.48 Da. The two minor ones have monoisotopic masses of 20076.46 and 20147.48 Da. The corresponding multi-charged ESI mass spectra are reported in the Supplementary Figure S9. The preliminary analyses allowed us to relate the components with *m*_mono_ 20131.48 and 20147.48 Da with the trait Gly^353^-Ala^532^ of the legumin J-like entry with the Acc. No. A0A1S2XVG1 (Fig. 3E). Instead, the two polypeptides with *m*_mono_ 20060.47 and 20076.46 Da are related to the trait Gly^353^-His^531^ of this legumin, and therefore, both are lacking the C-terminal alanine. This amino acid region shows two cysteine residues, respectively localized at positions 359 and 438. By TD-MS2 analysis (see Supplementary Material, Figures S11 and S12, Table S4), the polypeptide with the experimental *m*_mono_ 20060.47 Da was identified with the region Gly^353^-His^531^ carrying the Cys^359^ as carbamidomethyl-cysteine and the Cys^438^ as sulfinic acid. The minor abundant polypeptide with the *m*_mono_ 20076.46 Da, although no TD-MS2 data were obtained, likely corresponds to the same polypeptide with Cys^438^ as sulfonic acid. In this respect, by the shotgun approach, the cysteine at position 359 was always identified in the carbamidomethylated form, whereas the cysteine residue at position 438 was identified in both di-oxidized and tri-oxidized forms (Figures S10a and S10b). Analogously, the other two polypeptides, with *m*_mono_ 20131.48 and 20147.48 Da, could be related to the region Gly^353^-Ala^532^, carrying different oxidized forms of the cysteine residue at position 438. The component with *m*_mono_ 20131.48 Da could correspond to this region with the Cys^359^ as carbamidomethyl-cysteine and the Cys^438^ as sulfinic acid, whereas the minor polypeptide with *m*_mono_ 20147.48 Da should carry the Cys^438^ as sulfonic acid. The presence of the Cys^438^ as sulfinic acid in the polypeptide with *m*_mono_ 20131.48 Da was confirmed by TD-MS2 (see Supplementary Material, Figures S13 and S14, Table S5). Instead, the assignment of the minor component with *m*_mono_ 20147.48 Da remains putative because no TD-MS2 data were obtained for this polypeptide. The results about these polypeptides related to the legumin A-like entry with the Acc. No. A0A1S2XVG1 are summarized in Table 2.

**Table 2:** Top-down results about the polypeptide chains related to the Gly^353^-Ala^532^ and Gly^353^-His^531^ regions of the legumin A-like entry with the Acc. No. A0A1S2XVG1. Each polypeptide chain is reported: the amino acid region, the status of two cysteines at positions 359 and 438 of the β-chain, the experimentally determined monoisotopic mass, the theoretical monoisotopic mass, and the error in ppm. The last two columns show whether the corresponding proteoform was supported by MS/MS data of the bottom-up and top-down approaches

	**AA region**	**Cys**^**359**^	**Cys**^**438**^	**Exp. *m***_**mono**_** (Da)**	**Theor. *m***_**mono**_** (Da)**	**Error (ppm)**	**MS/MS data by shotgun**	**MS/MS data by top-down**
1	Gly^353^-His^531^	CM ^a)^	di-ox ^b)^	20060.47	20060.46	0.4	X	X
2	Gly^353^-His^531^	CM	tri-ox ^c)^	20076.46	20076.45	0.4	X	
3	Gly^353^-Ala^532^	CM	di-ox	20131.48	20131.50	1.0	X	X
4	Gly^353^-Ala^532^	CM	tri-ox	20147.48	20147.49	1.5	X	

^a)^*CM*, carbamidomethylation

^b)^*di-ox*, cysteine di-oxidized as sulfinic acid

^c)^*tri-ox*, cysteine tri-oxidized as sulfonic acid

Figure 3C shows the deconvoluted spectra of four polypeptides with *m*_mono_ 20044.27, 20019.24, 20035.22, and 20076.23 Da that, by a preliminary analysis, appear related to the region Gly^315^-Asn^496^ of the legumin-like entry with the Acc. No. A0A3Q7XNW1 (Fig. 3F). The corresponding multi-charged ESI mass spectra are reported in the Supplementary Figure S15. The polypeptides with *m*_mono_ 20,044.27 and 20,019.24 Da were unequivocally identified by TD-MS2 of their multi-charged ions (see Supplementary Material, Figures S16–S19, Tables S6 and S7). The component with *m*_mono_ 20044.27 Da corresponds to the region Gly^315^-Asn^496^ carrying both the cysteine residues, located at 321 and 400 positions, as carbamidomethyl-cysteines, whereas the polypeptide with *m*_mono_ 20019.24 Da was identified as the same region Gly^315^-Asn^496^, but carrying Cys^321^ as carbamidomethyl-cysteine and Cys^400^ as sulfinic acid. As already observed for the peptides related to the legumin A0A1S2XSB9, by the shotgun approach, the cysteine at position 321 of the legumin A0A3Q7XNW1 was always identified in the carbamidomethylated form. On the contrary, the Cys^400^ was also identified as sulfinic and sulfonic acid (Figure S20). The identification of a peptide carrying Cys^400^ as sulfinic acid confirms the assignment of the polypeptide at 20019.24 Da, whereas the presence of this cysteine as sulfonic acid might be related to the component at 20035.22 Da. For this component, no TD-MS2 data were obtained; however, it may correspond to the Gly^315^–Asn^496^ region, with Cys^321^ identified as carbamidomethylated cysteine and Cys^400^ as cysteic acid (sulfonic acid). Finally, no TD-MS2 data were obtained for the polypeptide with a monoisotopic mass of 20076.23 Da. But the mass difference of + 56.99 Da observed with respect to the mass of the component at 20019.24 Da could be due to an over-alkylation of the protein that occurred during the sample preparation step of alkylation by IAA (Δ*m* =  + 57.02 Da). The results about these polypeptides related to the legumin A-like entry with the Acc. No. A0A3Q7XNW1 are summarized in Table 3.

**Table 3:** Top-down results about the polypeptide chains related to the Gly^315^-Asn^496^ region of the legumin A-like entry with the Acc. No. A0A3Q7XNW1. Each polypeptide chain is reported: the amino acid region, the status of two cysteines at positions 321 and 400, the experimentally determined monoisotopic mass, the theoretical monoisotopic mass, and the error in ppm. The last two columns show whether the corresponding proteoform was supported by MS/MS data of the bottom-up and top-down approaches

	**AA region**	**Cys**^**321**^	**Cys**^**400**^	**Exp. *m***_**mono**_** (Da)**	**Theor. *m***_**mono**_** (Da)**	**Error (ppm)**	**MS/MS data by shotgun**	**MS/MS data by top-down**
1	Gly^315^-Asn^496^	CM ^a)^	di-ox ^b)^	20019.24	20019.24	0.09	X	X
2	Gly^315^-Asn^496^	CM	tri-ox ^c)^	20035.22	20035.23	−0.4	X	
3	Gly^315^-Asn^496^	CM	CM	20044.27	20044.27	−0.1	X	X
4	Gly^315^-Asn^496^	CM	di-ox	20076.23 ^d)^	20076.26	−1.5	X	

^a)^*CM*, carbamidomethylation

^b)^*di-ox*, cysteine di-oxidized as sulfinic acid

^c)^*tri-ox*, cysteine tri-oxidized as sulfonic acid

^d)^Carrying an additional iodacetamide molecule (over-alkylation)

Overall, in the enriched fraction of legumins, polypeptides related to specific C-terminal regions of the three legumin entries with the Acc. No. A0A1S2XSB9, A0A1S2XVG1, and A0A3Q7XNW1 were identified and characterized. The amino acid sequences of these three legumins, as reported in the TrEMBL section of the UniProt DB, are displayed in Fig. 3D, E, and F. In their mature forms, the legumins A0A1S2XSB9 and A0A3Q7XNW1 show five cysteine residues, whereas the entry A0A1S2XVG1 has seven cysteines. About the legumin entry A0A1S2XSB9, our data revealed a group of polypeptides corresponding to the region Gly^334^-Asn^514^, which differ in post-translational modifications (PTMs) of the cysteine residue at position 419 (Fig. 3D). This residue was identified as carbamidomethyl-cysteine, sulfinic acid, and sulfonic acid. The existence of polypeptides corresponding to this amino acid region of the legumin entry A0A1S2XSB9 can be due to a proteolytic event that cleaves the precursor legumin protein at the level of the Asn^333^-Gly^334^ bond. All these polypeptides lack the last four amino acids KAAA, which are reported in the C-termini of the corresponding entry A0A1S2XSB9 deposited in the UniProt DB. In this respect, it is interesting to note that by the shotgun approach, it was possible to obtain the coverage of about 90% of the whole protein sequence, but this amino acid trait has never been characterized. Altogether, these findings suggest that the precursor legumin protein present in the genotype “Pascià” here employed, in comparison with the entry sequence taken as reference, has a shorter length and is lacking these four C-terminal amino acids. Another group of four polypeptides was related to the legumin entry A0A1S2XVG1. Two polypeptides correspond to the region Gly^353^-Ala^532^, whereas the other two correspond to the region Gly^353^-His^531^, and therefore lack the C-terminal alanine residue (Fig. 3E). As already reported for the polypeptides related to the legumin A0A1S2XSB9, these components also differ in PTMs of a cysteine residue (Cys^438^), which was found as sulfinic or sulfonic acid. All these polypeptides show, as the N-terminal residue, the glycine located at position 353 of the precursor legumin sequence. Therefore, they could represent the products of the proteolytic cleavage occurring at the level of the Asn^352^-Gly^353^ bond.

Finally, three polypeptides related to the region Gly^315^-Asn^496^ of the legumin entry A0A3Q7XNW1 were characterized (Fig. 3 panel F). They could arise from a proteolytic cleavage occurring at the level of the Asn^314^-Gly^315^ bond of the precursor legumin protein. These components differ in the status of the cysteine residue at position 400, which was found as carbamidomethyl-cysteine, sulfinic acid, or sulfonic acid. As already observed for the polypeptides derived by the legumin A0A1S2XSB9, the last C-terminal four amino acids KAAA of the legumin entry A0A3Q7XNW1 were not characterized by the approaches here employed. This result suggests that also this legumin component, in comparison with the entry sequence taken as reference, has a shorter length and is lacking these four amino acids in the C-terminal region. Altogether, these findings highlight two interesting aspects worth elaborating upon. Firstly, all the 20kDa polypeptides derived from the legumins here characterized reasonably correspond to the β-chains that, as already observed in other legumes [8], are produced by the post-translational proteolytic cleavage of their legumin precursor (Fig. 3G). This proteolytic cleavage occurs at a single well-conserved Asn-Gly peptide bond of the precursor legumins, as well as for the 11S seed-storage proteins of a wide variety of plant species [32]. It has been demonstrated that this cleavage event is catalyzed by an asparaginyl endopeptidase that has an absolute specificity for the asparagine on the N-terminal side of the cleaved peptide bond, but shows a lower specificity for the amino acids on the C-terminal side [33].

The other interesting aspect is related to the detection of different oxidation states of the thiol group of one out of two cysteine residues located in the legumin β-chains here characterized. Each of these legumin β-chains shows two cysteine residues located at conserved positions. The first cysteine residue appears in the N-terminal portion of the legumin β-chain, whereas the second one is in the middle of the C-terminal cupin domain (Fig. 3). By similarity with the structure of soybean proglycinin [14], it has previously been hypothesized that the inter-chain disulfide bond linking the α- and β-chains involves the cysteine located in the N-terminal region of the β-polypeptide, whereas the second cysteine residue of the basic chain has a free thiol group. Cysteinyl residues have unique physico-chemical properties and, besides the different functions they play, are also susceptible to various PTMs [34]. Cysteine residues not employed in the formation of disulfide bonds (i.e., free cysteines) carry a thiol group, which can deprotonate as a consequence of various interactions with its environment. Deprotonation generates a thiolate that increases the nucleophilicity of the side chain and the cysteine reactivity [35]. Therefore, these reactive cysteines can undergo many different oxidation states in response to different redox signals. The first reaction of oxidation of a thiol group forms sulfenic acid (–SOH) that is intrinsically unstable and an intermediary to many other oxidation states [36], including the relatively more stable sulfinic (–SO_2_H) and sulfonic (–SO_3_H) acids. Sulfinic and sulfonic acid forms are typically associated with oxidative distress, but while the S-sulfinylation is a reversible status, the formation of the S-sulfonic, which is the most highly oxidized species of thiol, is completely irreversible [37]. MS data revealed that the cysteine in the N-terminal portion of the β-chains here characterized was always identified as carbamidomethyl cysteine, as a consequence of the sample preparation that included the reactions of reduction and alkylation with IAA. Instead, the cysteine residue located in the middle of the C-terminal cupin domain of the β-polypeptide was also detected as sulfinic and sulfonic acid. These findings might suggest, although only indirectly, that also in the chickpea legumins, the inter-chain disulfide bond that links the α- and β-chains involves the cysteine located in the N-terminal region of the β-polypeptide, whereas the cysteine residue situated in the middle of the C-terminal cupin domain is probably present as free cysteine (Fig. 3G).

From a physiological point of view, proteolytic cleavage at a highly conserved site and formation of the interchain disulfide bond are essential post-translational modifications required for the assembly of processed proglobulin trimers into mature 11S hexamers [38, 39]. The strong evolutionary conservation of the cleavage site highlights its critical role in storage protein packaging and function. In addition, the interchain disulfide bond is necessary for establishing the proper three-dimensional conformation required for stable hexamer assembly. Disruption of this bond prevents hexamer formation and can result in premature degradation of storage protein subunits [40]. Collectively, these structural and processing mechanisms are key regulators of storage protein stability and play an important role in controlling nitrogen and carbon sink/source relationships in developing seeds.

### Shotgun MS analysis of the vicilin-enriched fraction

The peptide-based bottom-up proteomics analysis of the vicilin-enriched fraction allowed the identification of 12 proteins, which correspond to those already identified in the legumin-enriched fraction. The group of vicilins includes seven entries with the UniProt Acc. Nos. A0A1S2XQR4, A0A1S2Y087, A0A1S2YZ56, A0A1S2XYZ0, A0A1S2XQ88, Q304D4, and A0A1S2YKD9. This result confirms that a completely selective separation of these two classes of globulins was not achieved, although a partial enrichment of vicilins (41% of this fraction) was obtained. Protein identifications and matching peptides in the vicilin-enriched fraction are listed in Supplementary Table S8.

### Top-down MS analysis of the vicilin-enriched fraction

**Fig. 4:**
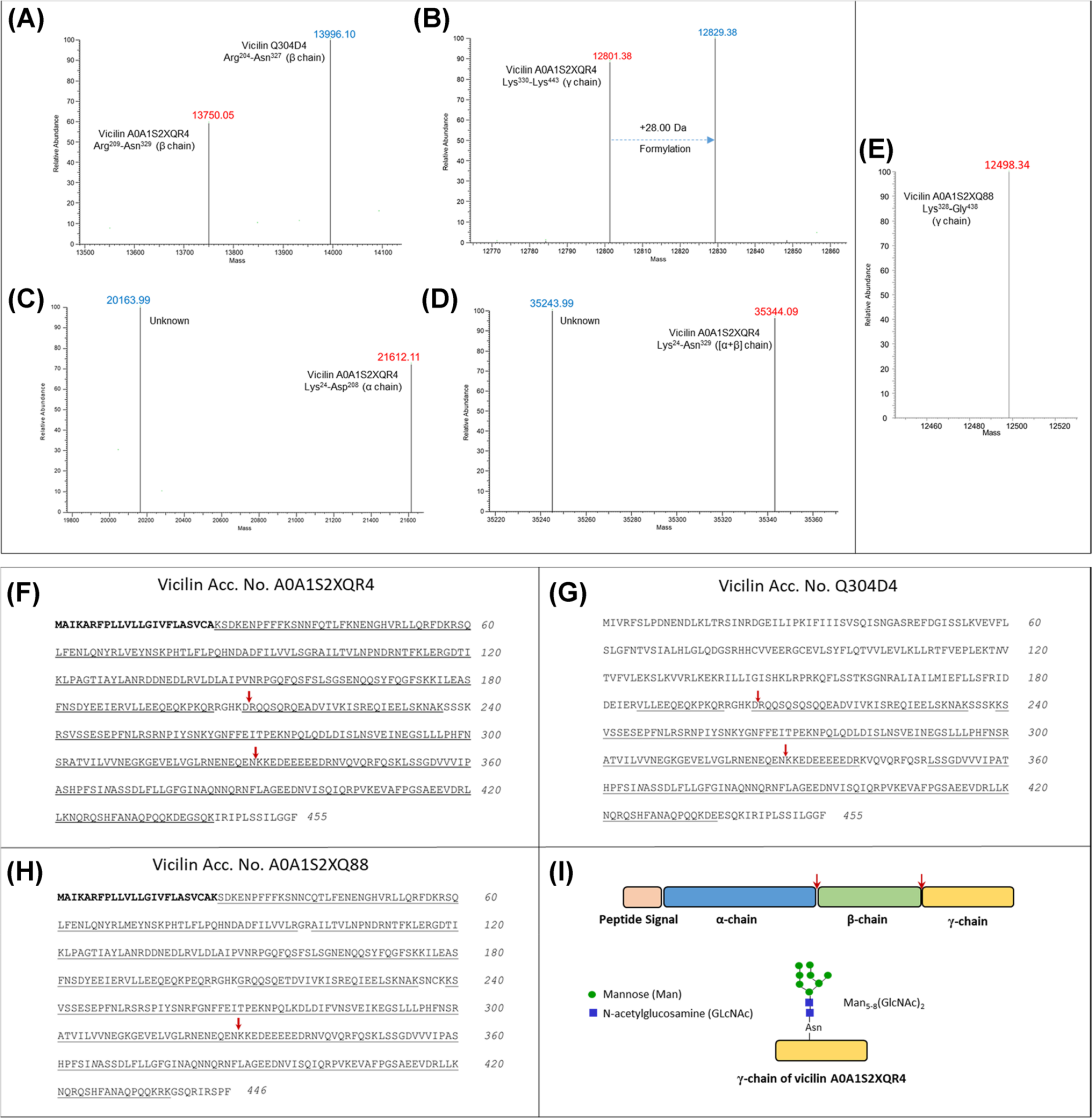
Monoisotopic deconvoluted mass spectra (mass zero charge) of the post-translational polypeptide products coming from precursor vicilins with the Acc. No. A0A1S2XQR4 (**A**–**D**), Q304D4 (**A**), and A0A1S2XQ88 (**E**). The corresponding multi-charged ESI-MS are shown in the Supplementary Material. **F**, **G**, and **H** show the amino acid sequence of the vicilins A0A1S2XQR4, Q304D4, and A0A1S2XQ88, respectively. Peptide signal, lacking in the mature form of the protein, is reported in bold. The length of the peptide signal for the vicilin Q304D4 is not reported in UniProt DB. The sequence characterized via the shotgun approach is underlined. The cleavage sites at the level of α/β and β/γ junctions are indicated by a red arrow. The potential asparagine glycosylation sites are reported in italics. **I** shows the schematic picture of the post-translational proteolytic cleavage generating the α-, β, and γ-chains. The proposed structure of the N-glycan linked to the γ-chain of vicilin A0A1S2XQR4 is also shown with the maximum level of mannose units observed by the MS data. The exact linkages between the terminal mannose residues and the core of Man(GlcNAc)_2_ oligosaccharide were not determined. They were hypothesized by similarity with those reported for vicilin-like proteins of other Fabaceae

The reduced and IAA-alkylated vicilins enriched fraction was also investigated by LC–MS analysis using a top-down approach for intact protein characterization. Using the same approach above described for the legumins, some polypeptides related with specific amino acid regions of the chickpea vicilins with the Acc. No. A0A1S2XQR4, Q304D4, and A0A1S2XQ88 were detected and characterized. In detail, Fig. 4A reports the deconvoluted spectrum of two co-eluting polypeptides with experimental *m*_mono_ of 13750.05 and 13996.10 Da. TD-MS2 of the polypeptide with *m*_mono_ 13750.05 Da (see Supplementary Material, Figures S22 and S23, Table S9) allowed us to identify this component with the amino acid region Arg^209^-Asn^329^ of the vicilin-like protein with the Acc. No. A0A1S2XQR4 (Fig. 4F).

Although no TD-MS2 data were acquired for the component with *m*_mono_ of 13996.10 Da, it reasonably corresponds to the amino acid region Arg^204^-Asn^327^ of the chickpea vicilin with the Acc. No. Q304D4 (Fig. 4G). Figure 4B shows the deconvoluted spectrum of two co-eluting polypeptides with *m*_mono_ 12801.38 and 12829.38 Da. By TD-MS2 (see Supplementary Material, Figures S25 and S26, Table S10), the polypeptide with *m*_mono_ 12801.38 Da was identified with the amino acid region Lys^330^-Lys^443^ of the vicilin-like protein with the Acc. No. A0A1S2XQR4 (Fig. 4F). For the component with *m*_mono_ 12829.38 Da, no TD-MS2 data were obtained. But it probably corresponds to the same polypeptide carrying a formylation (theoretical mass shift + 28.00 Da). Figure 4C and D shows the deconvoluted spectra of two polypeptides with *m*_mono_ of 21612.11 and 35344.09 Da, respectively. Although for these two polypeptides, no TD-MS2 spectra were obtained, their experimentally determined molecular masses correspond, within an error of about 1.5 ppm, to the theoretical masses of the amino acid regions 24–208 and 24–329, respectively, of the vicilin-like protein with the Acc. No. A0A1S2XQR4. These MS spectra also show the presence of two other co-eluting components with *m*_mono_ of 20163.99 and 35243.99 Da that, however, actually remain unidentified. Finally, a polypeptide with *m*_mono_ of 12498.34 Da was also detected (Fig. 4E). By TD-MS2 (Figures S30 and S31, Table S11), it was identified with the amino acid region Lys^328^-Gly^438^ of the vicilin with the Acc. No. A0A1S2XQ88 (Fig. 4H). The multi-charged ESI mass spectra of the polypeptides related to vicilin proteins are reported in the Supplementary Figures S21, S24, S27, S28, and S29. The results of the top-down investigation of the vicilin-enriched fraction are summarized in Table 4.

**Table 4:** The columns report the (i) amino acid regions and the putative fragment chains of the vicilin proteins with the Acc. No. A0A1S2XQR4, Q304D4, and A0A1S2XQ88 and (ii) the experimental and theoretical monoisotopic masses and the error in ppm. The last two columns show whether the corresponding proteoform was supported by MS/MS data of the bottom-up and top-down approaches. Amino acid positions are referred to the whole sequence (i.e., also containing the signal peptide)

**AA region (chain)**	**Exp. *m***_**mono**_** (Da)**	**Theor. *m***_**mono**_** (Da)**	**Error (ppm)**	**MS/MS data by shotgun**	**MS/MS data by top-down**
Vicilin A0A1S2XQR4					
Lys^24^-Asp^208^ (α chain)	21612.11	21612.09	1.3	X	
Arg^209^-Asn^329^ (β chain)	13750.05	13750.03	2.8	X	X
Lys^24^-Asn^329^ ([α + β] chain)	35344.09	35344.11	−0.7	X	
Lys^330^-Lys^443^ (γ chain)	12801.38	12801.39	−1.4	X	X
Lys^330^-Lys^443^ (γ chain) *	12829.38	12829.39	−0.4	X	
Vicilin Q304D4					
Arg^204^-Asn^327^ (β chain)	13996.10	13996.11	−0.3	X	
Vicilin A0A1S2XQ88					
Lys^328^-Gly^438^ (γ chain)	12498.34	12498.34	0.9	X	X

*Formylated form

Overall, in the enriched fraction of vicilins, although six different vicilins were identified by the shotgun approach, the top-down strategy allowed the detection and characterization of a group of polypeptides related to three chickpea vicilins entries, namely, the entries deposited in the UniProt DB with the Acc. No. A0A1S2XQR4, Q304D4, and A0A1S2XQ88. Figure 4F shows the amino acid sequence of the chickpea vicilin-like protein entry with Acc. No. A0A1S2XQR4, that in its mature form does not show any cysteine residue. By the shotgun approach, it was possible to obtain the coverage of the whole protein sequence (412 out of 432 amino acids present in the mature form, 95,4%), except for two tetrapeptides (i.e., RGHK and SSSK) and the last 12 C-terminal amino acids (i.e., IRIPLSSILGGF). In the top-down approach, four polypeptides corresponding to the amino acid regions Lys^24^-Asp^208^, Arg^209^-Asn^329^, Lys^24^-Asn^329^, and Lys^330^-Lys^443^ were detected and characterized (Table 4). By comparison with the homologous vicilins of the pea, these polypeptides might correspond to the so-called α-, β-, (α + β)-, and γ-chains, respectively, that are generated by a proteolytic event at two bonds (Fig. 4I). Specifically, the Asp^208^-Arg^209^ (namely the α/β junction) and the Asn^329^-Lys^330^ (the β/γ junction) peptide bonds. About these proteolytic cleavage sites, it is interesting to note that their locations are similar to those found in pea vicilin [22, 41], being the α/β junction situated in the linker region joining the two cupin domains, and the β/γ junction located in an exposed loop in the C-terminal cupin domain. Moreover, the β/γ junction in both pea and chickpea vicilins shows an asparagine residue at position P1 of the peptide bond. Differently, while the α/β junction, in pea vicilin, is located at the level of a Lys-Asp bond, chickpea corresponds to an Asp-Arg bond. In pea vicilin, it has been reported that the cleavages of these two junctions are catalyzed by two different proteases: a serine protease for the α/β junction and a legumain for the β/γ one [22, 41]. Although out of the scope of the present work, we could hypothesize that the cleavage of the chickpea vicilin at both junction sites might be carried out by the same enzyme family, namely, the legumains. Indeed, legumains are a family of cysteine endopeptidases involved in legume storage protein-limited proteolysis and degradation [42] with a cleavage specificity at the carboxylic side of asparagine and aspartic acid peptide bonds. However, a much lower efficiency of legumains is reported for the aspartic acid. The MS data here reported indeed suggest that the intact vicilin is cleaved first at the β/γ junction and produces as intermediate the [α + β]-polypeptide and the γ-polypeptide. Then, the [α + β]-polypeptide undergoes proteolytic cleavage at the α/β junction, leading to the formation of the α- and β-chains. However, an initial vicilin cleavage at the α/β junction, followed by the cleavage at the β/γ junction, cannot be excluded a priori. The last 12 C-terminal amino acids (i.e., I^444^RIPLSSILGGF^455^) reported in the database entry A0A1S2XQR4 have never been identified in either of the approaches here adopted. Indeed, the γ-polypeptide shows the lysine at position 443, as a C-terminal amino acid. The absence of these 12 C-terminal amino acids could be related to an extensive post-translational endo-proteolytic processing at the C-terminal portion of the pro-vicilin, as already reported for pea vicilin [43]. Finally, the top-down analysis identified two post-translational products related to the vicilins with Acc. No. Q304D4 and A0A1S2XQ88. MS data allowed us to relate the first component with the region Arg^204^-Asn^327^ of the chickpea vicilin-like protein entry Q304D4 (Fig. 4G), and the second one with the region Lys^328^-Gly^438^ of the vicilin entry A0A1S2XQ88 (Fig. 4H). The amino acid region Arg^204^-Asn^327^ reasonably corresponds to the β-chain of the vicilin Q304D4. This is generated by the proteolytic events at the level of the bonds Asp^203^-Arg^204^ (the α/β junction) and the Asn^327^-Lys^328^ (the β/γ junction), both catalyzed by a legumain. The region Lys^328^-Gly^438^ of the vicilin entry A0A1S2XQ88 corresponds to the γ-chain of this vicilin. This polypeptide is produced by a proteolytic event at the level of the bond Asn^327^-Lys^328^ (the β/γ junction), reasonably catalyzed by a legumain. Overall, these findings allow us to reveal the proteolytic processing occurring during seeds development and maturation in chickpea vicilins. In this respect, legume species differ in whether or not the 7S globulins stored in seeds undergo cleavage processes before germination. As examples, vicilin cleavage in developing pea, lentil, and white lupin [44, 45] occurs before germination. In contrast, in soybean and peanut, this event occurs only after germination [46, 47]. Pea cultivars exhibit varying patterns of cleavage during seed maturation: some show cleavage at both sites, others at only one site, while some show no cleavage at all. These differences have been attributed to both the diversity of pea vicilin gene sequences and environmental factors, particularly climate conditions [33]. In the chickpea cultivar “Pascià” investigated here, the processing of native vicilins occurs during seed development and involves, at different steps, both cleavage sites. Considering that the MS analyses were performed on a pooled sample obtained by mixing three biological replicates, the identified proteoforms should be interpreted as representative of the overall combined proteomic profile of these replicates rather than of individual samples. Consequently, the experimental design adopted in this study does not allow us to evaluate the reproducibility or consistency of each proteoform across the separate biological replicates. Likewise, we cannot completely rule out the existence of natural biological variability among individual samples of the same genotype. Nevertheless, the MS data did not reveal the presence of additional proteoforms generated by alternative proteolytic cleavage events. In particular, no evidence was found for proteoforms originating from different cleavage sites, suggesting that the identified proteoforms are the predominant forms detectable under the experimental conditions employed in this study. We may suppose that, like in other legumes, proteolytic processing of chickpea vicilins during seed development may facilitate their subsequent mobilization during germination by rendering the compact bicupin structure more susceptible to degradation. The presence of active proteases during seed maturation could enable rapid amino acid release after germination without compromising storage protein accumulation or quaternary structure. In this respect, tight regulation of protease activity is essential to prevent premature extensive proteolysis and futile cycles of protein synthesis and degradation. Chickpea vicilin-processing proteases, beyond their physiological role, may also have important applications in food and nutraceutical industries. Controlled hydrolysis of legume storage proteins can improve functional properties such as solubility, emulsification, and foaming capacity, while also enhancing the release of bioactive peptides and reducing allergenicity.

### Characterization of the structure of the N-glican linked to the vicilin A0A1S2XQR4

**Fig. 5:**
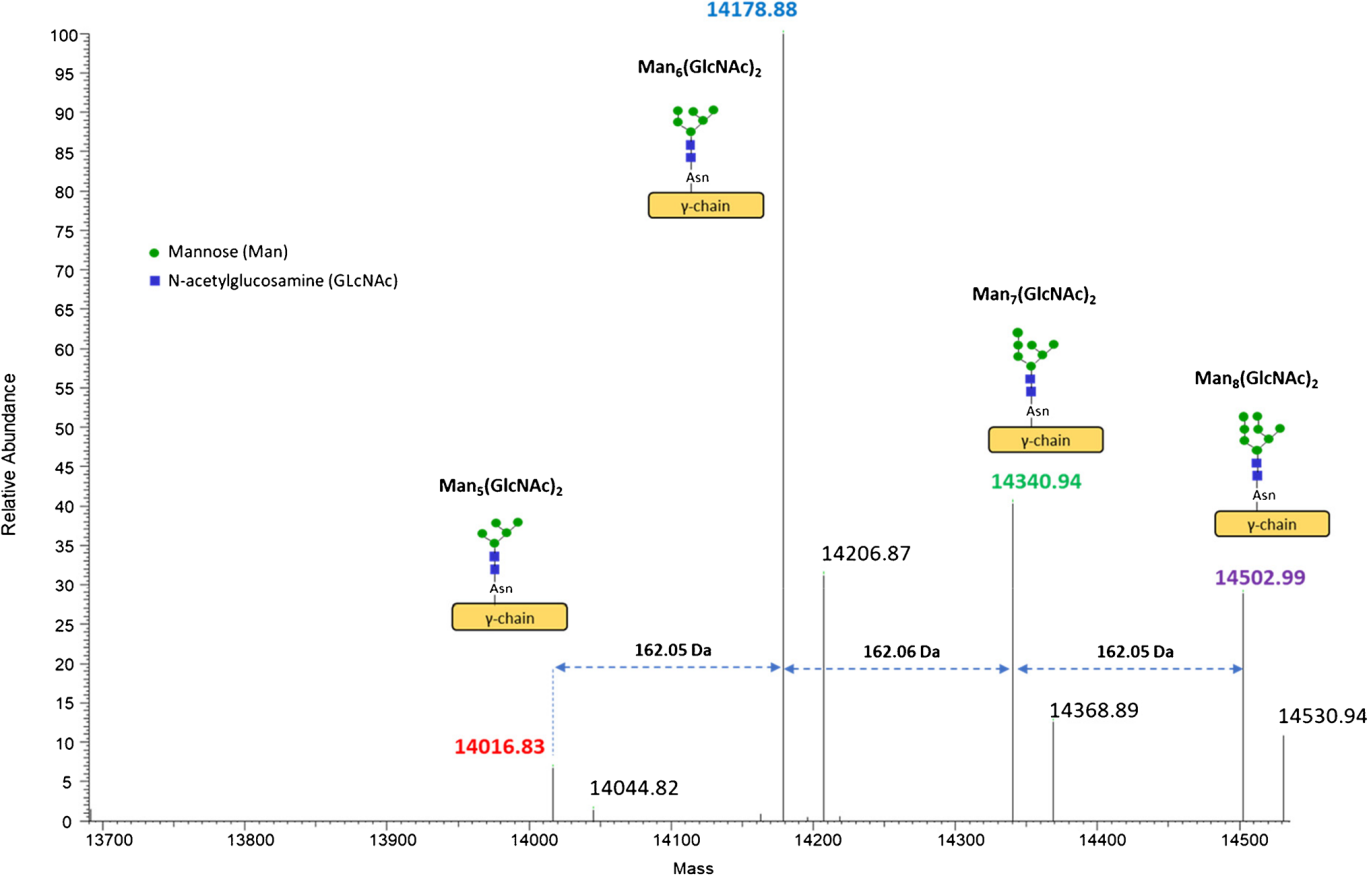
Monoisotopic deconvoluted mass spectrum (mass zero-charge) of four glycosylated forms of the γ-polypeptide product coming from precursor vicilin with the Acc. No. A0A1S2XQR4. Each signal corresponding to a glycosylated form of the γ-polypeptide shows a satellite peak with a mass value of about + 28 Da, probably related to a formylation. The proposed structure of the linked N-glycan is also shown. The exact linkages between the terminal mannose residues and the core of Man(GlcNAc)_2_ oligosaccharide were not determined. They were hypothesized by similarity with those reported for vicilin-like proteins of other Fabaceae

As described in the Introduction, vicilin-like proteins from other Fabaceae have been reported as sparsely *N*-glycosylated. Therefore, considering that the amino acid sequence of the vicilin proteins here detected shows consensus Asn-Xaa-Ser/Thr glycosylation sites, located at position 368 in the vicilin A0A1S2XQR4, 366 in the vicilin A0A1S2XQ88, and at positions 119 and 366 in the vicilin Q304D4, this post-translational modification was investigated. Figure 5 reports the deconvoluted mass spectrum of four co-eluting polypeptides with experimental *m*_mono_ of 14016.83, 14178.88, 14340.94, and 14502.99 Da. The corresponding multi-charged ESI mass spectrum is reported in the Supplementary Figure S32.

These signals differ from each other by about 162 Da, a value which corresponds to the mass of a single unit of mannose (theoretical mass 162.05 Da). Preliminary analysis of TD-MS2 data allowed us to relate the component at 14178.88 Da with the amino acid region Lys^330^-Lys^443^ of the vicilin A0A1S2XQR4 (theoretical mass 12801.39 Da), which shows a unique potential *N*-glycosylation site at position 368 (Fig. 4F). The putative structure of the N-glycan linked to the component at 14178.88 Da was derived by an in-depth interpretation of TD-MS2 data (see Supplementary Material, Figures S33 and S34, Tables S12, S13, and S14). MS data suggest a high-mannose-type structure having the formula Man_6_(GlcNAc)_2_, constituted by a core of two N-acetylglucosamine (GlcNAc) residues linked to the Asn^368^, and six mannose (Man) units at the non-reducing end. The exact linkages between the terminal mannose residues and the core of Man(GlcNAc)_2_ oligosaccharide were not determined. The proposed structure, shown in Fig. 4I, was hypothesized by similarity with those identified for vicilin-like proteins of other Fabaceae [17, 18]. No TD-MS2 data were obtained for the other three polypeptides reported in Fig. 5. But, taking into account that these signals differ from each other by about 162 Da, it can be hypothesized that also these components are glycosyl-forms of the amino acid region Lys^330^-Lys^443^ of the vicilin A0A1S2XQR4, differing from each other by a mannose unit. Consequently, the component at 14016.83 Da shows an oligosaccharide side chain with five mannose units, whereas the components at 14340.94 and 14502.99 Da are the glycosyl-forms with seven and eight mannose units, respectively (Fig. 5). Each of the signals related to these components shows a satellite peak with a mass value of about + 28 Da. These peaks could correspond to the same polypeptides carrying a formylation, as already observed for the not glycosylated form of this amino acid region (see Fig. 4B). The results of the top-down investigation of the glycosylated forms of the γ-chain of the vicilin A0A1S2XQR4 are summarized in Table 5. It is worth noting that no glycosylated peptides were identified or characterized using the complementary bottom-up approach. Indeed, due to their low abundance, glycopeptides generally require dedicated analytical strategies, including enrichment or separation from the non-glycosylated fraction, which was not performed in the present study. However, although this aspect was not within the primary scope of the study, it remains an important topic for further in-depth investigation.

**Table 5:** N-glycosylated forms of the γ-chain (Lys^330^-Lys^443^) of the vicilin protein with the Acc. No. A0A1S2XQR4. The columns report the experimental and theoretical monoisotopic masses, the relative delta mass in ppm, and the hypothesized N-glycan formula by the MS data. Amino acid positions are referred to the whole sequence (i.e., also containing the signal peptide). The last two columns show whether the corresponding glycosylated form was supported by MS/MS data of the bottom-up and top-down approaches

**Vicilin A0A1S2XQR4** **AA region (chain)**	**Exp. *m***_**mono**_** (Da)**	**Theor. *m***_**mono**_** (Da)**	**Error (ppm)**	**N-Glycan formula linked to the Asn**^**368**^	**MS/MS data by shotgun**	**MS/MS data by top-down**
Lys^330^-Lys^443^ (γ chain)	14016.83	14016.81	1.1	Man_5_(GlcNAc)_2_		
Lys^330^-Lys^443^ (γ chain)	14178.88	14178.87	1.0	Man_6_(GlcNAc)_2_		X
Lys^330^-Lys^443^ (γ chain)	14340.94	14340.92	1.0	Man_7_(GlcNAc)_2_		
Lys^330^-Lys^443^ (γ chain)	14502.99	14502.97	0.9	Man_8_(GlcNAc)_2_		

From a physiological perspective, glycosylation may increase hydrophilicity and molecular flexibility of vicilins, but also slightly reduce digestibility by limiting protease accessibility. Thus, this post-translation modification may modulate the allergenic properties of vicilin-like proteins. In fact, vicilins and, to a lesser extent, legumins have already been shown to resist both in vivo and in vitro digestion and represent potential allergens. On the other hand, glycosylation improves the functional properties of chickpea vicilins, including solubility, emulsifying capacity, foaming ability, and thermal stability. These characteristics are particularly important in chickpea-based foods such as protein beverages, hummus, and aquafaba products, where glycosylated vicilins contribute to texture, emulsion stability, and foam formation.

## Conclusions

Integrated bottom-up and top-down mass spectrometry strategies were used to reveal the proteolytic processing of legumins and vicilins in chickpeas (*Cicer arietinum* L.). Overall, MS data suggest that in chickpea legumins, the proteolytic cleavage generating the α- and β-chains is catalyzed by an asparaginyl endopeptidase. Indeed, this cleavage event occurs at a single well-conserved Asn-Gly peptide bond of the precursor legumins, as already observed for the 11S seed-storage proteins of other plant species. Moreover, our data suggest that, similarly to soybean proglycinins, the inter-chain disulfide bond linking the α- and β-chains might involve the cysteine located in the N-terminal region of the β-polypeptide, whereas the cysteine of the C-terminal cupin domain is probably present as free cysteine. On the other hand, characterization of the degradation products of three vicilin proteins evidences that, like in other legumes, chickpea vicilins show two proteolytic cleavage sites located in conserved amino acid regions, showing an asparagine or an aspartate residue at position P1 of the peptide bond cleaved. Consequently, legumains, a family of cysteine endopeptidases with a cleavage specificity at the carboxylic side of asparagine and aspartic acid peptide bonds, might catalyze these proteolytic events. Likely the vicilin-like proteins of other Fabaceae, four *N*-glycosylated forms of the vicilin A0A1S2XQR4 were also detected. They show as an oligosaccharide side-chain a high-mannose structure with the formula Man_5–8_(GlcNAc)_2_ and differ from each other by a mannose unit. Overall, the proteolytic cleavage of chickpea legumins and vicilins represents a key physiological mechanism regulating storage protein assembly, stability, and mobilization during seed development and germination. Glycosylation of vicilins further modulates their structural and functional properties by increasing hydrophilicity, flexibility, and technological performance, although it may also influence digestibility and allergenic potential. Beyond their biological significance, these post-translational modifications have important technological implications, since controlled protein hydrolysis and glycosylation can improve solubility, emulsifying and foaming properties, thermal stability, and the generation of bioactive peptides, supporting the use of chickpea proteins in functional foods, nutraceuticals, and innovative plant-based products. Finally, the bioanalytical strategy adopted here demonstrates the complementarity of different MS-based methods for in-depth characterization of processing events that affect storage proteins in plants.

## Supplementary Information

Below is the link to the electronic supplementary material.

Supplementary file1 (DOCX 8.19 MB)

Supplementary file2 (XLSX 198 KB)

Supplementary file3 (XLSX 202 KB)

## Data Availability

The mass spectrometry proteomics data have been deposited to the ProteomeXchange Consortium (proteomexchange.org) via the PRIDE partner repository with the dataset identifier PXD078377. All ProteomeXchange datasets are publicly available. The datasets can be accessed via the individual repository websites. For PRIDE database, the datasets can be accessed via PRIDE Archive. In addition, datasets can be searched at ProteomeCentral (https://proteomecentral.proteomexchange.org/).
