# Supplementary material for: Proteoform analysis by mass spectrometry reveals post-translational processing of legumins and vicilins in chickpeas (Cicer arietinum L.)
Source: Anal Bioanal Chem. 2026 May 29;418(15):4847–64. doi: 10.1007/s00216-026-06577-0 (PMC13388682; doi:10.1007/s00216-026-06577-0)
Supplement: Supplementary file 1 — Supplementary file1 (DOCX 8.19 MB) [file 216_2026_6577_MOESM1_ESM.docx]

*Supplementary Material*

**Proteoform analysis by mass spectrometry reveals post-translational processing of legumins and vicilins in chickpeas (Cicer arietinum L.)**

Antonella Di Francesco ^a^, Aldo Lanzoni ^a^, Maria Gaetana Giovanna Pittalà ^a^, Rosaria Saletti ^a^, Ole N. Jensen ^b^, and Vincenzo Cunsolo ^a,*^

^a^ Laboratory of Organic Mass Spectrometry (LOMS), Department of Chemical Sciences, University of Catania, Catania, Italy

^b^ Department of Biochemistry and Molecular Biology, University of Southern Denmark, Odense, Denmark

* Correspondence: vcunsolo@unict.it

**Characterization of the Legumin-enriched Fraction**

The complete list of the proteins and the supporting peptides identified in the legumin-enriched fraction are reported in Supplementary Table S1.

***Polypeptides related to the legumin A0A1S2XSB9***

Supplementary Figure S1 shows the multi-charged ESI mass spectra of the components having m_mono_ 20021.2166, 19989.2364, 19980.1759, and 19964.2195 Da, and that are relate to the amino acid region Gly^334^-Asn^514^ of the legumin A-like entry with the Acc. No. A0A1S2XSB9.

**
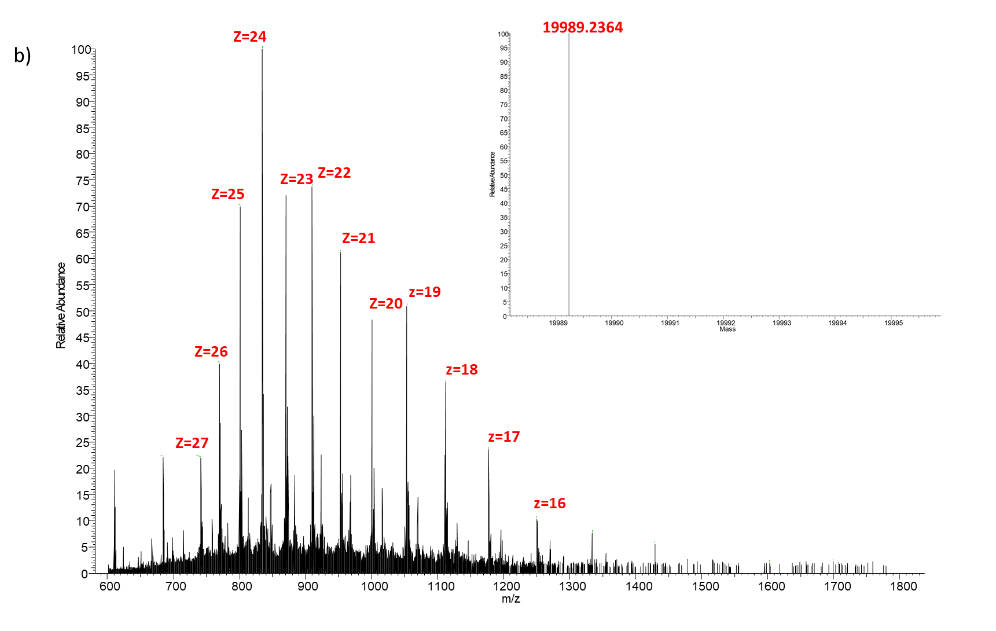

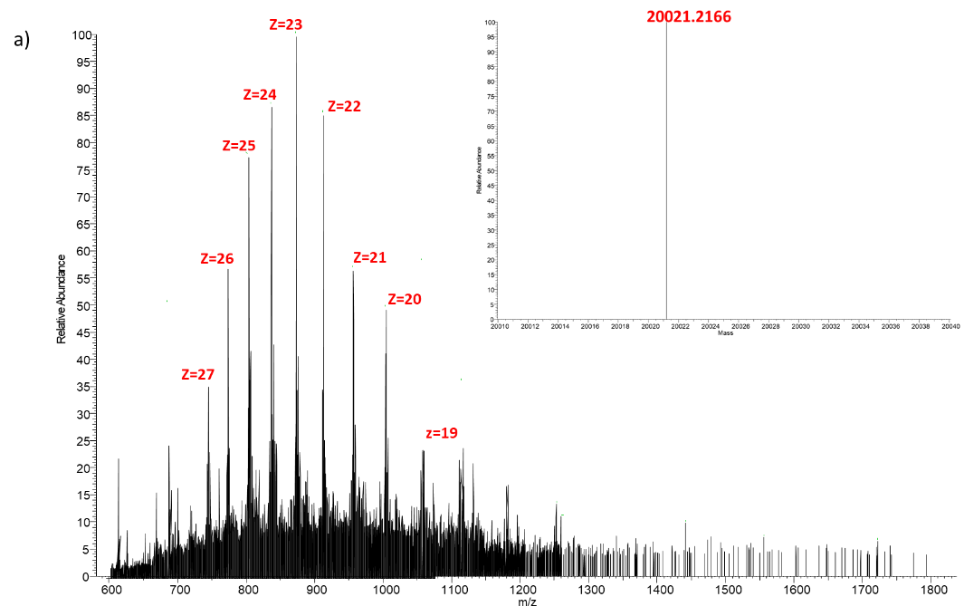

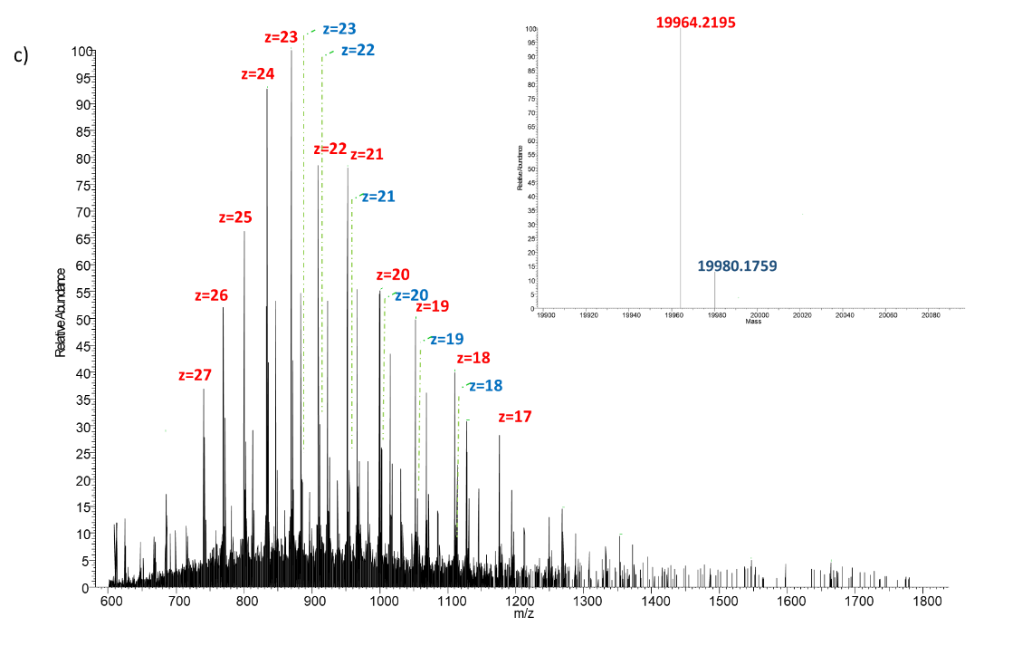
**

**Figure S1.** Multi-charged mass spectrum and monoisotopic deconvoluted mass spectrum (mass zero-charge) of four polypeptides with mass of 20021.2166 (a), 19989.2364 (b), 19980.1759 (c), and 19964.2195 Da (c).

Top-down characterization of the component with the experimental mass 19989.2364 Da was achieved by the MS/MS of its multi-charged ion [M+24H]^24+^ at m/z 834.3929, interpreted by the ClipsMS tool. This polypeptide was identified with the region Gly^334^-Asn^514^ of the legumin A-like entry with Acc. No. A0A1S2XSB9 carrying both the cysteine residues as carbamidomethyl-cysteines. Figure S2a shows the corresponding deconvoluted MS/MS and reports the attributions of the most intense peaks. Interpretation of the signals was carried out using the amino acid region Gly^334^-Asn^514^ of the entry A0A1S2XSB9 as a reference sequence, which was renumbered as Gly^1^-Asn^181^ (Figure S2b). Consequently, the cysteine located in the entire legumin sequence at position 340, in this polypeptide fragment, is at position 7, whereas the Cys^419^ is situated at position 86. Figure S2a shows three peaks, at m/z 2234.0119, 2446.0932, and 2559.1761, which correspond to the *b_20_, b_22_,* and *b_23_* fragments, respectively, of the reference sequence. This group of fragments is diagnostic for the presence of the cysteine at position 7 as carbamidomethyl-cysteine. Moreover, a lot of intense signals didn't match with any terminal fragment, and therefore they were searched as possible internal fragments using the ClipsMS tool. In this respect, it is worth to note that annotation of internal fragment ions is not trivial because a vast number of theoretical internal fragments may be generated from a protein. The possible existence of many theoretical fragments with overlapping masses introduces a high risk of false-positive annotation. To prevent false-positive assignments , after the matching process, all putative internal fragment assignments were manually verified, and the following criteria were applied: (i) the putative internal fragments were real peaks rather than noise or isotopes; (ii) when both a terminal and an internal fragment matched the same peak, the terminal fragment was the preferential assignment; (iii) when two terminal fragments matched the same peak, the internal fragment with the lower mass error was selected; (iv) when two terminal fragments matched the same peak with identical mass error, both assignments were reported but, whenever possible, the enhanced cleavages N-terminal to proline and C-terminal to acidic residues was considered.^[[1]](#endnote-1)^ It should be noted that most of the internal fragments were generated by cleavages N-terminal to proline, which are reported as the most prominent fragmentation events across all internal fragments (proline effect) ^[[2]](#endnote-2)^, improving the protein sequencing. In addition, although at low intensity, the signal at m/z 9643.9278 can be putatively assigned to the internal fragment Phe^2^-Cys^86^, carrying the cysteine at position 86 as carbamidomethyl-cysteine.


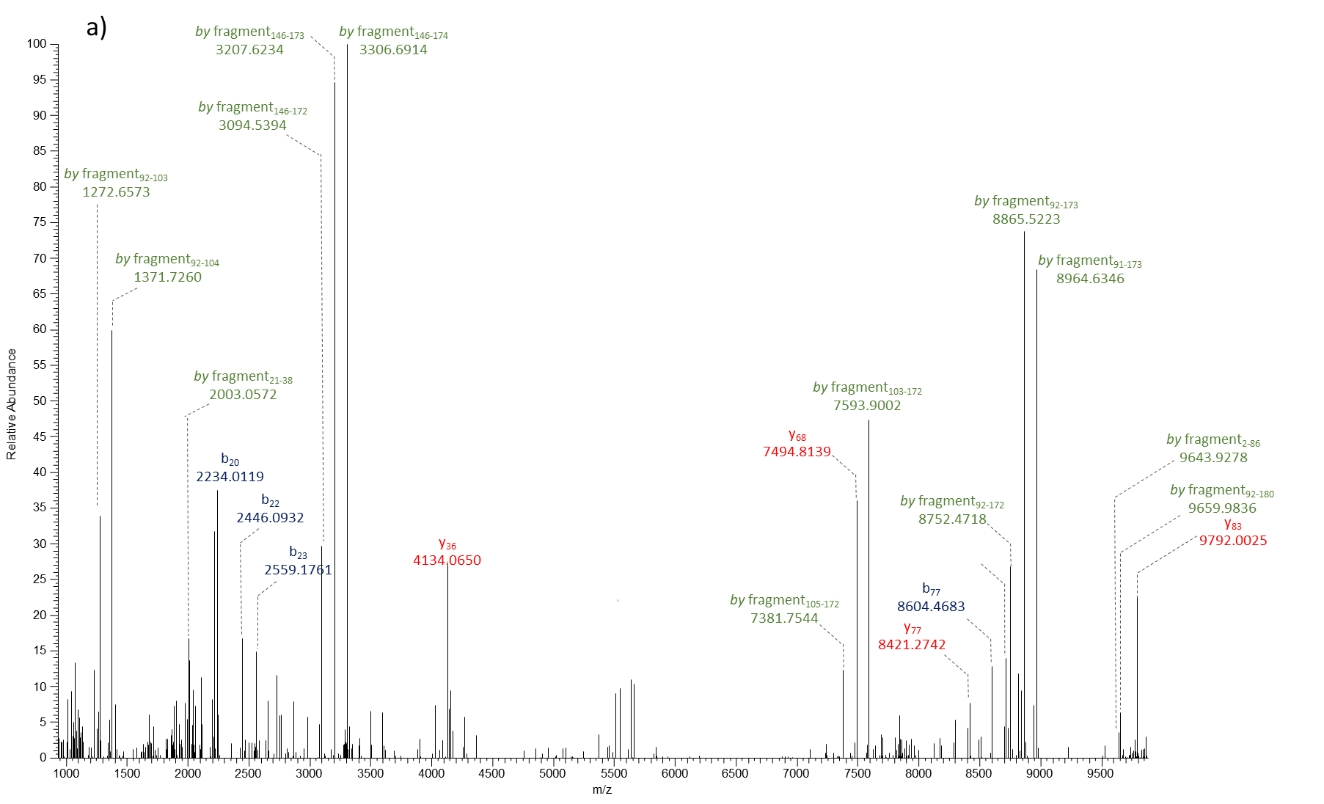


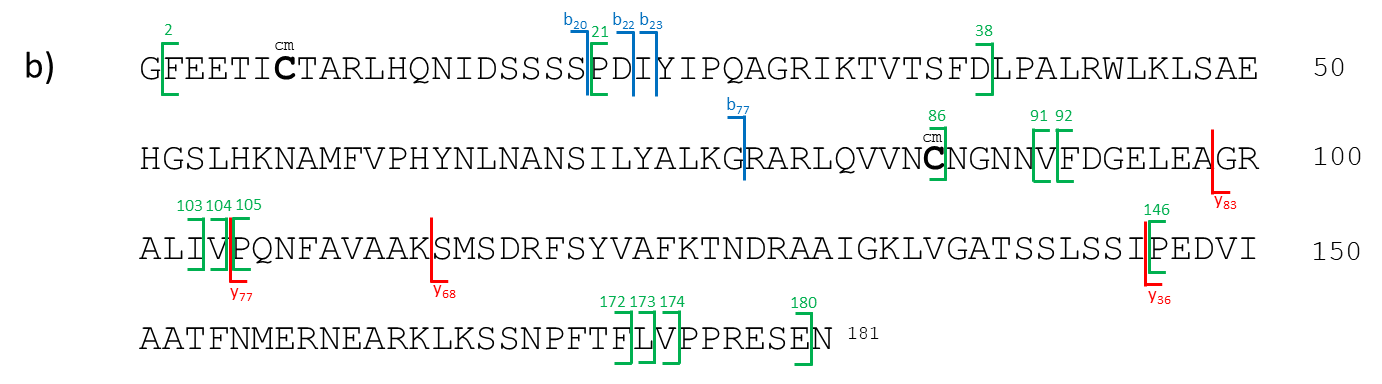


**Figure S2.** a) Deconvoluted MS/MS of the ion at m/z 834.3929, which correspond to the multi-charged ion [M+24H]^24+^ of the polypeptide with m_mono_ 19989.2364 Da. The most abundant y- and b-ions are marked in red and blue, respectively. The most intense internal fragment ions are shown in green. b) Sequence coverage map as obtained by the MS/MS reported in the panel a. This polypeptide corresponds to the region Gly334-Asn514 (here renumbered 1-181) of the legumin A-like entry with Acc. No. A0A1S2XSB9 carrying both the cysteine residues as carbamidomethyl-cysteines. The cysteines are showed in bold and marked as cm (carbamidomethylated). b- and y-fragments are reported in blue and red, respectively. The internal fragments are reported in green.

Following the complete report of the ClipsMS interpretation (Fig.S3 and Table S2).


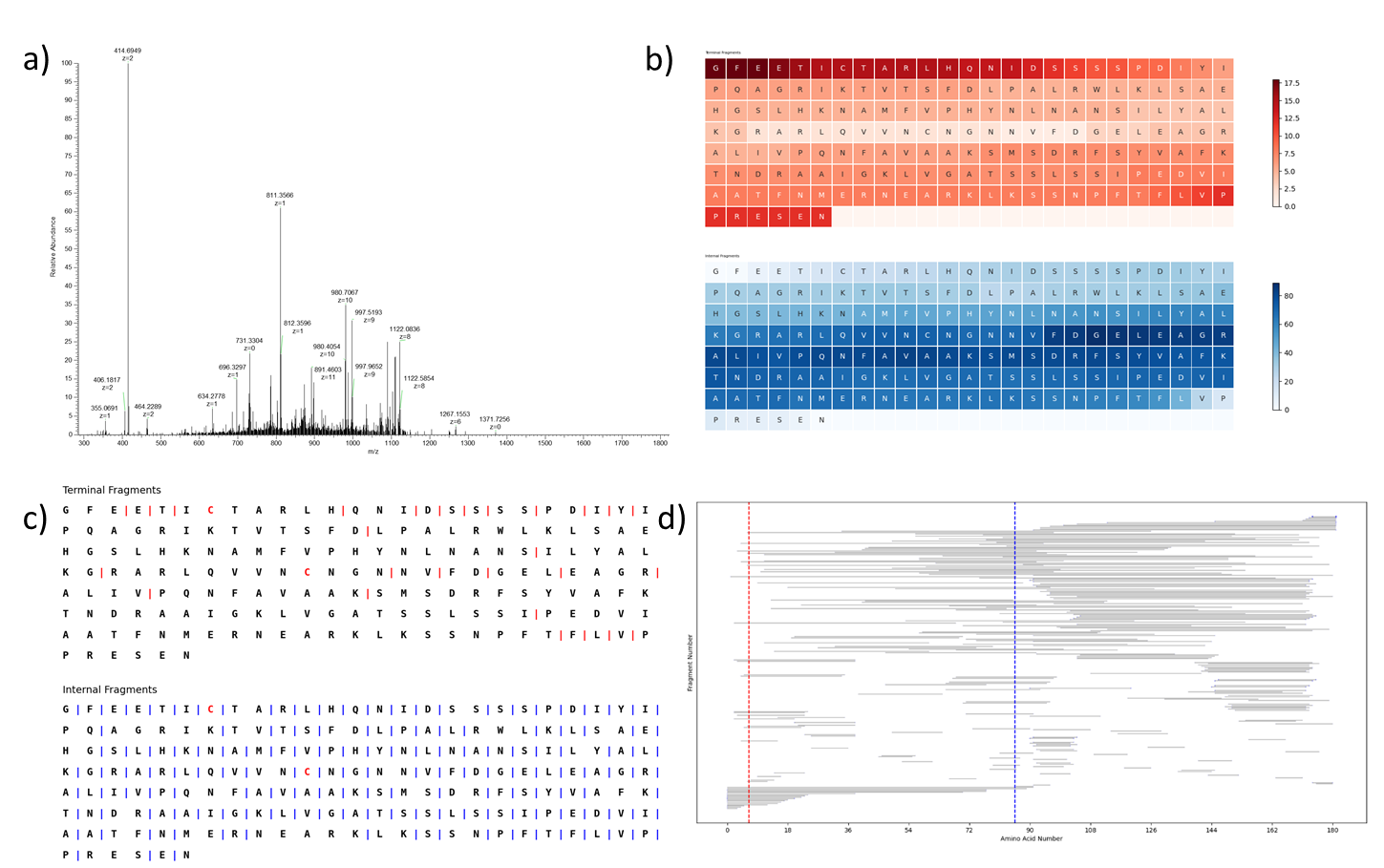


**Figure S3.** a) HCD mass spectrum of the ion at m/z 834.3929 ([M+24H]^24+^); b) sequence coverage map for the terminal and internal fragments. Darker regions indicate more coverage; c) fragment cleavage map indicating the location of inter-amino-acid cleavage sites for terminal and internal fragments; and d) fragment location map indicating the region of the β-chain sequence covered by terminal and internal fragments. The two lines, red and blue, indicate the cysteine residue positions.

**Table S2**. Sample output of ClipsMS related to the CID mass spectrum of the at m/z 834.3929 ([M+24H]^24+^). The Table includes the fragment type, observed mass, theoretical mass, start amino acid, end amino acid, error (ppm), sequence, intensity, and molecular formula. The amino acid positions refer to the sequence reported in Figure S2b

| **Frag Type** | **Localized Mod** | **Observed Mass** | **Theoretical Mass** | **Start AA** | **End AA** | **Error** | **Sequence** | **Intensity** | **Formula** |
| --- | --- | --- | --- | --- | --- | --- | --- | --- | --- |
| B Fragment | 0 | 334.1392 | 334.1397 | 1 | 3 | -1.7 | GFE | 3754.41 | C16 H20 N3 O5 S0 |
| B Fragment | 0 | 463.1816 | 463.1823 | 1 | 4 | -1.7 | GFEE | 4233.77 | C21 H27 N4 O8 S0 |
| B Fragment | 0 | 564.2294 | 564.23 | 1 | 5 | -1.1 | GFEET | 3614.13 | C25 H34 N5 O10 S0 |
| B Fragment | 57.0215 | 1415.6718 | 1415.6737 | 1 | 12 | -1.3 | GFEETICTARLH | 727.63 | C59 H92 N17 O18 S1 |
| B Fragment | 57.0215 | 1770.8586 | 1770.8592 | 1 | 15 | -0.4 | GFEETICTARLHQNI | 285.66 | C74 H117 N22 O23 S1 |
| B Fragment | 57.0215 | 1885.8853 | 1885.8862 | 1 | 16 | -0.5 | GFEETICTARLHQNID | 4058.8 | C78 H122 N23 O26 S1 |
| B Fragment | 57.0215 | 1972.9153 | 1972.9182 | 1 | 17 | -1.5 | GFEETICTARLHQNIDS | 4347.06 | C81 H127 N24 O28 S1 |
| B Fragment | 57.0215 | 2059.9506 | 2059.9502 | 1 | 18 | 0.2 | GFEETICTARLHQNIDSS | 4062.04 | C84 H132 N25 O30 S1 |
| B Fragment | 57.0215 | 2234.0119 | 2234.0143 | 1 | 20 | -1.1 | GFEETICTARLHQNIDSSSS | 20841.89 | C90 H142 N27 O34 S1 |
| B Fragment | 57.0215 | 2446.0932 | 2446.094 | 1 | 22 | -0.3 | GFEETICTARLHQNIDSSSSPD | 9293.89 | C99 H154 N29 O38 S1 |
| B Fragment | 57.0215 | 2559.1761 | 2559.1781 | 1 | 23 | -0.8 | GFEETICTARLHQNIDSSSSPDI | 8274.64 | C105 H165 N30 O39 S1 |
| B Fragment | 57.0215 | 2722.2379 | 2722.2414 | 1 | 24 | -1.3 | GFEETICTARLHQNIDSSSSPDIY | 6523.02 | C114 H174 N31 O41 S1 |
| B Fragment | 57.0215 | 4236.0683 | 4236.0667 | 1 | 38 | 0.4 | GFEETICTARLHQNIDSSSSPDIYIPQAGRIKTVTSFD | 6313.57 | C182 H285 N50 O61 S1 |
| B Fragment | 57.0215 | 7845.9332 | 7845.9446 | 1 | 70 | -1.5 | GFEETICTARLHQNIDSSSSPDIYIPQAGRIKTVTSFDLPALRWLKLSAEHGSLHKNAMFVPHYNLNANS | 1498.38 | C347 H537 N98 O103 S2 |
| B Fragment | 57.0215 | 8604.3889 | 8604.4137 | 1 | 77 | -2.9 | GFEETICTARLHQNIDSSSSPDIYIPQAGRIKTVTSFDLPALRWLKLSAEHGSLHKNAMFVPHYNLNANSILYALKG | 745.89 | C385 H599 N106 O111 S2 |
| B Fragment | 57.0215 | 8604.4683 | 8604.4137 | 1 | 77 | 6.3 | GFEETICTARLHQNIDSSSSPDIYIPQAGRIKTVTSFDLPALRWLKLSAEHGSLHKNAMFVPHYNLNANSILYALKG | 7158.07 | C385 H599 N106 O111 S2 |
| B Fragment | 114.043 | 9986.06 | 9986.1134 | 1 | 89 | -5.4 | GFEETICTARLHQNIDSSSSPDIYIPQAGRIKTVTSFDLPALRWLKLSAEHGSLHKNAMFVPHYNLNANSILYALKGRARLQVVNCNGN | 312.92 | C438 H691 N128 O127 S3 |
| B Fragment | 114.043 | 10199.1972 | 10199.2248 | 1 | 91 | -2.7 | GFEETICTARLHQNIDSSSSPDIYIPQAGRIKTVTSFDLPALRWLKLSAEHGSLHKNAMFVPHYNLNANSILYALKGRARLQVVNCNGNNV | 547.93 | C447 H706 N131 O130 S3 |
| Y Fragment | 0 | 828.3841 | 828.3846 | 175 | 181 | -0.7 | PPRESEN | 361457.5 | C33 H54 N11 O14 S0 |
| Y Fragment | 0 | 927.4524 | 927.453 | 174 | 181 | -0.7 | VPPRESEN | 21423.12 | C38 H63 N12 O15 S0 |
| Y Fragment | 0 | 1040.5365 | 1040.5371 | 173 | 181 | -0.6 | LVPPRESEN | 5248.03 | C44 H74 N13 O16 S0 |
| Y Fragment | 0 | 1187.6044 | 1187.6055 | 172 | 181 | -1 | FLVPPRESEN | 955.96 | C53 H83 N14 O17 S0 |
| Y Fragment | 0 | 4134.065 | 4134.0713 | 146 | 181 | -1.5 | PEDVIAATFNMERNEARKLKSSNPFTFLVPPRESEN | 15048.93 | C181 H286 N51 O58 S1 |
| Y Fragment | 0 | 7494.8139 | 7494.785 | 114 | 181 | 3.9 | SMSDRFSYVAFKTNDRAAIGKLVGATSSLSSIPEDVIAATFNMERNEARKLKSSNPFTFLVPPRESEN | 20040.5 | C328 H523 N92 O105 S2 |
| Y Fragment | 0 | 8421.2742 | 8421.2824 | 105 | 181 | -1 | PQNFAVAAKSMSDRFSYVAFKTNDRAAIGKLVGATSSLSSIPEDVIAATFNMERNEARKLKSSNPFTFLVPPRESEN | 4299.21 | C371 H589 N104 O116 S2 |
| Y Fragment | 0 | 8817.5262 | 8817.556 | 101 | 181 | -3.4 | ALIVPQNFAVAAKSMSDRFSYVAFKTNDRAAIGKLVGATSSLSSIPEDVIAATFNMERNEARKLKSSNPFTFLVPPRESEN | 6588.41 | C391 H625 N108 O120 S2 |
| Y Fragment | 0 | 9230.8077 | 9230.7583 | 97 | 181 | 5.3 | EAGRALIVPQNFAVAAKSMSDRFSYVAFKTNDRAAIGKLVGATSSLSSIPEDVIAATFNMERNEARKLKSSNPFTFLVPPRESEN | 929.34 | C407 H652 N115 O126 S2 |
| Y Fragment | 0 | 9529.8956 | 9529.9064 | 94 | 181 | -1.1 | GELEAGRALIVPQNFAVAAKSMSDRFSYVAFKTNDRAAIGKLVGATSSLSSIPEDVIAATFNMERNEARKLKSSNPFTFLVPPRESEN | 976.12 | C420 H673 N118 O131 S2 |
| Y Fragment | 0 | 9529.9216 | 9529.9064 | 94 | 181 | 1.6 | GELEAGRALIVPQNFAVAAKSMSDRFSYVAFKTNDRAAIGKLVGATSSLSSIPEDVIAATFNMERNEARKLKSSNPFTFLVPPRESEN | 279.16 | C420 H673 N118 O131 S2 |
| Y Fragment | 0 | 9792.0025 | 9792.0018 | 92 | 181 | 0.1 | FDGELEAGRALIVPQNFAVAAKSMSDRFSYVAFKTNDRAAIGKLVGATSSLSSIPEDVIAATFNMERNEARKLKSSNPFTFLVPPRESEN | 12472.56 | C433 H687 N120 O135 S2 |
| BY Int Fragment | 0 | 499.2257 | 499.2259 | 87 | 91 | -0.5 | NGNNV | 4479.29 | C19 H31 N8 O8 S0 |
| BY Int Fragment | 0 | 562.2501 | 562.2508 | 92 | 96 | -1.2 | FDGEL | 1330.69 | C26 H36 N5 O9 S0 |
| BY Int Fragment | 0 | 599.2786 | 599.2784 | 176 | 180 | 0.3 | PRESE | 536.22 | C24 H39 N8 O10 S0 |
| BY Int Fragment | 0 | 696.3305 | 696.3311 | 175 | 180 | -0.9 | PPRESE | 11154.3 | C29 H46 N9 O11 S0 |
| BY Int Fragment | 57.0215 | 739.3715 | 739.3668 | 7 | 12 | 6.3 | CTARLH | 351.05 | C28 H48 N11 O7 S1 |
| BY Int Fragment | 0 | 821.4402 | 821.4377 | 8 | 14 | 3.1 | TARLHQN | 569.69 | C34 H57 N14 O10 S0 |
| BY Int Fragment | 0 | 847.4351 | 847.4308 | 67 | 74 | 5 | NANSILYA | 1593.31 | C38 H59 N10 O12 S0 |
| BY Int Fragment | 0 | 875.4603 | 875.4621 | 164 | 171 | -2.1 | LKSSNPFT | 2118.94 | C40 H63 N10 O12 S0 |
| BY Int Fragment | 0 | 877.462 | 877.4639 | 10 | 16 | -2.2 | RLHQNID | 2591.86 | C37 H61 N14 O11 S0 |
| BY Int Fragment | 0 | 896.4724 | 896.4724 | 144 | 152 | 0 | SIPEDVIAA | 3946.76 | C40 H66 N9 O14 S0 |
| BY Int Fragment | 0 | 897.4757 | 897.4789 | 94 | 102 | -3.5 | GELEAGRAL | 1845.97 | C38 H65 N12 O13 S0 |
| BY Int Fragment | 0 | 902.4132 | 902.4102 | 15 | 23 | 3.3 | IDSSSSPDI | 2373.75 | C37 H60 N9 O17 S0 |
| BY Int Fragment | 0 | 903.4719 | 903.4782 | 135 | 144 | -7 | LVGATSSLSS | 1674.98 | C38 H67 N10 O15 S0 |
| BY Int Fragment | 0 | 903.4719 | 903.4782 | 136 | 145 | -7 | VGATSSLSSI | 1674.98 | C38 H67 N10 O15 S0 |
| BY Int Fragment | 57.0215 | 941.4938 | 941.4986 | 79 | 86 | -5.1 | ARLQVVNC | 915.54 | C37 H66 N13 O10 S1 |
| BY Int Fragment | 0 | 944.4954 | 944.4876 | 119 | 126 | 8.2 | FSYVAFKT | 1571.11 | C48 H66 N9 O11 S0 |
| BY Int Fragment | 0 | 975.4519 | 975.453 | 92 | 100 | -1.2 | FDGELEAGR | 1437.93 | C42 H63 N12 O15 S0 |
| BY Int Fragment | 57.0215 | 984.5095 | 984.5044 | 80 | 87 | 5.2 | RLQVVNCN | 178.94 | C38 H67 N14 O11 S1 |
| BY Int Fragment | 0 | 1010.5608 | 1010.5629 | 94 | 103 | -2.1 | GELEAGRALI | 1686.72 | C44 H76 N13 O14 S0 |
| BY Int Fragment | 0 | 1012.5027 | 1012.5058 | 93 | 102 | -3.1 | DGELEAGRAL | 4554.29 | C42 H70 N13 O16 S0 |
| BY Int Fragment | 0 | 1028.54 | 1028.5346 | 53 | 61 | 5.2 | SLHKNAMFV | 530.19 | C47 H74 N13 O11 S1 |
| BY Int Fragment | 0 | 1041.5411 | 1041.5463 | 141 | 150 | -5 | SLSSIPEDVI | 1438.89 | C46 H77 N10 O17 S0 |
| BY Int Fragment | 0 | 1046.4891 | 1046.4902 | 92 | 101 | -1 | FDGELEAGRA | 2446.53 | C45 H68 N13 O16 S0 |
| BY Int Fragment | 57.0215 | 1055.5422 | 1055.5415 | 79 | 87 | 0.6 | ARLQVVNCN | 2715.87 | C41 H72 N15 O12 S1 |
| BY Int Fragment | 0 | 1060.5531 | 1060.5534 | 46 | 55 | -0.3 | KLSAEHGSLH | 1544.65 | C46 H74 N15 O14 S0 |
| BY Int Fragment | 0 | 1060.5531 | 1060.5534 | 47 | 56 | -0.3 | LSAEHGSLHK | 1544.65 | C46 H74 N15 O14 S0 |
| BY Int Fragment | 0 | 1093.5533 | 1093.5459 | 110 | 119 | 6.8 | VAAKSMSDRF | 336.88 | C47 H77 N14 O14 S1 |
| BY Int Fragment | 57.0215 | 1099.4998 | 1099.495 | 82 | 91 | 4.4 | QVVNCNGNNV | 335.3 | C41 H68 N15 O15 S1 |
| BY Int Fragment | 0 | 1100.5765 | 1100.5775 | 167 | 176 | -0.9 | SNPFTFLVPP | 552.36 | C55 H78 N11 O13 S0 |
| BY Int Fragment | 0 | 1109.6304 | 1109.6313 | 94 | 104 | -0.9 | GELEAGRALIV | 1652.7 | C49 H85 N14 O15 S0 |
| BY Int Fragment | 0 | 1125.5902 | 1125.5899 | 93 | 103 | 0.3 | DGELEAGRALI | 8319.77 | C48 H81 N14 O17 S0 |
| BY Int Fragment | 0 | 1139.6544 | 1139.6531 | 26 | 36 | 1.1 | PQAGRIKTVTS | 130.73 | C49 H87 N16 O15 S0 |
| BY Int Fragment | 0 | 1139.6544 | 1139.6531 | 126 | 136 | 1.1 | TNDRAAIGKLV | 130.73 | C49 H87 N16 O15 S0 |
| BY Int Fragment | 0 | 1159.5729 | 1159.5742 | 92 | 102 | -1.2 | FDGELEAGRAL | 12119.03 | C51 H79 N14 O17 S0 |
| BY Int Fragment | 0 | 1224.6577 | 1224.6583 | 93 | 104 | -0.5 | DGELEAGRALIV | 6877.97 | C53 H90 N15 O18 S0 |
| BY Int Fragment | 0 | 1258.6413 | 1258.6426 | 91 | 102 | -1.1 | VFDGELEAGRAL | 3616.79 | C56 H88 N15 O18 S0 |
| BY Int Fragment | 0 | 1272.6573 | 1272.6583 | 92 | 103 | -0.8 | FDGELEAGRALI | 18800.45 | C57 H90 N15 O18 S0 |
| BY Int Fragment | 57.0215 | 1308.6833 | 1308.6842 | 5 | 15 | -0.7 | TICTARLHQNI | 153.6 | C53 H91 N18 O15 S1 |
| BY Int Fragment | 0 | 1343.7315 | 1343.7212 | 156 | 166 | 7.6 | MERNEARKLKS | 1219.18 | C55 H99 N20 O17 S1 |
| BY Int Fragment | 0 | 1371.726 | 1371.7267 | 91 | 103 | -0.5 | VFDGELEAGRALI | 33219.59 | C62 H99 N16 O19 S0 |
| BY Int Fragment | 0 | 1371.726 | 1371.7267 | 92 | 104 | -0.5 | FDGELEAGRALIV | 33219.59 | C62 H99 N16 O19 S0 |
| BY Int Fragment | 0 | 1401.7492 | 1401.7485 | 26 | 38 | 0.5 | PQAGRIKTVTSFD | 4197.59 | C62 H101 N18 O19 S0 |
| BY Int Fragment | 0 | 1677.8962 | 1677.8959 | 24 | 38 | 0.2 | YIPQAGRIKTVTSFD | 3395.57 | C77 H121 N20 O22 S0 |
| BY Int Fragment | 0 | 1742.8636 | 1742.8609 | 117 | 131 | 1.5 | DRFSYVAFKTNDRAA | 296.57 | C78 H116 N23 O23 S0 |
| BY Int Fragment | 0 | 1748.8196 | 1748.8326 | 50 | 64 | -7.4 | EHGSLHKNAMFVPHY | 298.78 | C80 H114 N23 O20 S1 |
| BY Int Fragment | 0 | 1818.8478 | 1818.8592 | 115 | 129 | -6.3 | MSDRFSYVAFKTNDR | 1493.44 | C80 H120 N23 O24 S1 |
| BY Int Fragment | 0 | 1857.9626 | 1857.9494 | 92 | 108 | 7.1 | FDGELEAGRALIVPQNF | 1808.02 | C85 H129 N22 O25 S0 |
| BY Int Fragment | 57.0215 | 1865.9817 | 1865.9875 | 74 | 90 | -3.1 | ALKGRARLQVVNCNGNN | 2268.67 | C74 H130 N29 O22 S1 |
| BY Int Fragment | 57.0215 | 1882.872 | 1882.8712 | 6 | 22 | 0.4 | ICTARLHQNIDSSSSPD | 3265.08 | C74 H121 N24 O28 S1 |
| BY Int Fragment | 57.0215 | 1882.872 | 1882.8712 | 7 | 23 | 0.4 | CTARLHQNIDSSSSPDI | 3265.08 | C74 H121 N24 O28 S1 |
| BY Int Fragment | 0 | 1906.0105 | 1906.0069 | 22 | 38 | 1.9 | DIYIPQAGRIKTVTSFD | 1051.4 | C87 H137 N22 O26 S0 |
| BY Int Fragment | 0 | 1929.9964 | 1930.0069 | 164 | 180 | -5.4 | LKSSNPFTFLVPPRESE | 1137.07 | C89 H137 N22 O26 S0 |
| BY Int Fragment | 0 | 2003.0572 | 2003.0597 | 21 | 38 | -1.2 | PDIYIPQAGRIKTVTSFD | 9345.61 | C92 H144 N23 O27 S0 |
| BY Int Fragment | 0 | 2004.016 | 2004.008 | 153 | 169 | 4 | TFNMERNEARKLKSSNP | 369.88 | C84 H139 N28 O27 S1 |
| BY Int Fragment | 0 | 2004.016 | 2004.008 | 155 | 171 | 4 | NMERNEARKLKSSNPFT | 369.88 | C84 H139 N28 O27 S1 |
| BY Int Fragment | 57.0215 | 2029.9244 | 2029.9244 | 3 | 20 | 0 | EETICTARLHQNIDSSSS | 2610.94 | C79 H130 N25 O32 S1 |
| BY Int Fragment | 0 | 2117.1015 | 2117.0873 | 16 | 35 | 6.7 | DSSSSPDIYIPQAGRIKTVT | 2630.99 | C92 H150 N25 O32 S0 |
| BY Int Fragment | 0 | 2198.1614 | 2198.1604 | 91 | 111 | 0.4 | VFDGELEAGRALIVPQNFAVA | 1613.54 | C101 H157 N26 O29 S0 |
| BY Int Fragment | 57.0215 | 2226.0464 | 2226.0456 | 4 | 23 | 0.4 | ETICTARLHQNIDSSSSPDI | 412.4 | C89 H146 N27 O34 S1 |
| BY Int Fragment | 57.0215 | 2241.2012 | 2241.2034 | 72 | 91 | -1 | LYALKGRARLQVVNCNGNNV | 3418.27 | C94 H159 N32 O26 S1 |
| BY Int Fragment | 57.0215 | 2355.0861 | 2355.0882 | 3 | 23 | -0.9 | EETICTARLHQNIDSSSSPDI | 1176.98 | C94 H153 N28 O37 S1 |
| BY Int Fragment | 57.0215 | 2518.146 | 2518.1515 | 3 | 24 | -2.2 | EETICTARLHQNIDSSSSPDIY | 1258.19 | C103 H162 N29 O39 S1 |
| BY Int Fragment | 0 | 2583.3143 | 2583.3137 | 152 | 173 | 0.2 | ATFNMERNEARKLKSSNPFTFL | 1361.33 | C115 H180 N33 O33 S1 |
| BY Int Fragment | 0 | 2654.3476 | 2654.3508 | 150 | 172 | -1.2 | IAATFNMERNEARKLKSSNPFTF | 4528.93 | C118 H185 N34 O34 S1 |
| BY Int Fragment | 0 | 2654.3476 | 2654.3508 | 151 | 173 | -1.2 | AATFNMERNEARKLKSSNPFTFL | 4528.93 | C118 H185 N34 O34 S1 |
| BY Int Fragment | 0 | 2753.4161 | 2753.4192 | 149 | 172 | -1.1 | VIAATFNMERNEARKLKSSNPFTF | 3342.48 | C123 H194 N35 O35 S1 |
| BY Int Fragment | 0 | 2753.4161 | 2753.4192 | 151 | 174 | -1.1 | AATFNMERNEARKLKSSNPFTFLV | 3342.48 | C123 H194 N35 O35 S1 |
| BY Int Fragment | 0 | 2767.4328 | 2767.4348 | 150 | 173 | -0.7 | IAATFNMERNEARKLKSSNPFTFL | 3419.24 | C124 H196 N35 O35 S1 |
| BY Int Fragment | 57.0215 | 2802.4737 | 2802.4468 | 71 | 95 | 9.6 | ILYALKGRARLQVVNCNGNNVFDGE | 377.44 | C120 H194 N37 O35 S1 |
| BY Int Fragment | 57.0215 | 2802.4737 | 2802.4468 | 72 | 96 | 9.6 | LYALKGRARLQVVNCNGNNVFDGEL | 377.44 | C120 H194 N37 O35 S1 |
| BY Int Fragment | 0 | 2815.4626 | 2815.4672 | 156 | 179 | -1.6 | MERNEARKLKSSNPFTFLVPPRES | 824.16 | C124 H200 N37 O36 S1 |
| BY Int Fragment | 0 | 2859.4953 | 2859.4921 | 131 | 158 | 1.1 | AIGKLVGATSSLSSIPEDVIAATFNMER | 206.61 | C125 H208 N33 O41 S1 |
| BY Int Fragment | 0 | 2864.4968 | 2864.5153 | 120 | 147 | -6.5 | SYVAFKTNDRAAIGKLVGATSSLSSIPE | 484 | C127 H207 N34 O41 S0 |
| BY Int Fragment | 0 | 2866.5026 | 2866.5033 | 149 | 173 | -0.2 | VIAATFNMERNEARKLKSSNPFTFL | 4440.71 | C129 H205 N36 O36 S1 |
| BY Int Fragment | 0 | 2866.5026 | 2866.5033 | 150 | 174 | -0.2 | IAATFNMERNEARKLKSSNPFTFLV | 4440.71 | C129 H205 N36 O36 S1 |
| BY Int Fragment | 0 | 2884.4911 | 2884.5192 | 40 | 64 | -9.7 | PALRWLKLSAEHGSLHKNAMFVPHY | 474.13 | C135 H203 N38 O31 S1 |
| BY Int Fragment | 0 | 2947.4728 | 2947.4731 | 146 | 171 | -0.1 | PEDVIAATFNMERNEARKLKSSNPFT | 827.32 | C128 H204 N37 O41 S1 |
| BY Int Fragment | 0 | 3060.5683 | 3060.5783 | 138 | 165 | -3.3 | ATSSLSSIPEDVIAATFNMERNEARKLK | 117.76 | C131 H219 N38 O44 S1 |
| BY Int Fragment | 0 | 3094.5394 | 3094.5415 | 146 | 172 | -0.7 | PEDVIAATFNMERNEARKLKSSNPFTF | 16507.33 | C137 H213 N38 O42 S1 |
| BY Int Fragment | 57.0215 | 3115.6315 | 3115.6218 | 66 | 93 | 3.1 | LNANSILYALKGRARLQVVNCNGNNVFD | 324.02 | C133 H217 N42 O39 S1 |
| BY Int Fragment | 0 | 3207.6234 | 3207.6256 | 91 | 120 | -0.7 | VFDGELEAGRALIVPQNFAVAAKSMSDRFS | 52516.38 | C143 H224 N39 O43 S1 |
| BY Int Fragment | 0 | 3207.6234 | 3207.6256 | 145 | 172 | -0.7 | IPEDVIAATFNMERNEARKLKSSNPFTF | 52516.38 | C143 H224 N39 O43 S1 |
| BY Int Fragment | 0 | 3207.6234 | 3207.6256 | 146 | 173 | -0.7 | PEDVIAATFNMERNEARKLKSSNPFTFL | 52516.38 | C143 H224 N39 O43 S1 |
| BY Int Fragment | 0 | 3278.6729 | 3278.6528 | 48 | 77 | 6.1 | SAEHGSLHKNAMFVPHYNLNANSILYALKG | 1101.04 | C148 H225 N42 O41 S1 |
| BY Int Fragment | 57.0215 | 3301.6967 | 3301.6859 | 66 | 95 | 3.3 | LNANSILYALKGRARLQVVNCNGNNVFDGE | 663.64 | C140 H227 N44 O43 S1 |
| BY Int Fragment | 57.0215 | 3301.6967 | 3301.6859 | 67 | 96 | 3.3 | NANSILYALKGRARLQVVNCNGNNVFDGEL | 663.64 | C140 H227 N44 O43 S1 |
| BY Int Fragment | 0 | 3306.6914 | 3306.694 | 146 | 174 | -0.8 | PEDVIAATFNMERNEARKLKSSNPFTFLV | 55417.96 | C148 H233 N40 O44 S1 |
| BY Int Fragment | 0 | 3306.6914 | 3306.694 | 147 | 175 | -0.8 | EDVIAATFNMERNEARKLKSSNPFTFLVP | 55417.96 | C148 H233 N40 O44 S1 |
| BY Int Fragment | 57.0215 | 3316.6694 | 3316.6856 | 68 | 97 | -4.9 | ANSILYALKGRARLQVVNCNGNNVFDGELE | 746.39 | C141 H228 N43 O44 S1 |
| BY Int Fragment | 57.0215 | 3316.6694 | 3316.6856 | 69 | 98 | -4.9 | NSILYALKGRARLQVVNCNGNNVFDGELEA | 746.39 | C141 H228 N43 O44 S1 |
| BY Int Fragment | 57.0215 | 3323.6926 | 3323.7165 | 5 | 34 | -7.2 | TICTARLHQNIDSSSSPDIYIPQAGRIKTV | 2465.44 | C141 H233 N42 O45 S1 |
| BY Int Fragment | 57.0215 | 3323.6926 | 3323.7165 | 6 | 35 | -7.2 | ICTARLHQNIDSSSSPDIYIPQAGRIKTVT | 2465.44 | C141 H233 N42 O45 S1 |
| BY Int Fragment | 0 | 3401.7467 | 3401.7634 | 109 | 141 | -4.9 | AVAAKSMSDRFSYVAFKTNDRAAIGKLVGATSS | 969.43 | C149 H242 N43 O46 S1 |
| BY Int Fragment | 0 | 3407.7458 | 3407.7416 | 144 | 173 | 1.2 | SIPEDVIAATFNMERNEARKLKSSNPFTFL | 1578.49 | C152 H240 N41 O46 S1 |
| BY Int Fragment | 0 | 3494.7733 | 3494.7737 | 142 | 172 | -0.1 | LSSIPEDVIAATFNMERNEARKLKSSNPFTF | 3683.22 | C155 H245 N42 O48 S1 |
| BY Int Fragment | 0 | 3494.7733 | 3494.7737 | 143 | 173 | -0.1 | SSIPEDVIAATFNMERNEARKLKSSNPFTFL | 3683.22 | C155 H245 N42 O48 S1 |
| BY Int Fragment | 0 | 3506.806 | 3506.8101 | 144 | 174 | -1.2 | SIPEDVIAATFNMERNEARKLKSSNPFTFLV | 1047.41 | C157 H249 N42 O47 S1 |
| BY Int Fragment | 0 | 3593.8395 | 3593.8421 | 143 | 174 | -0.7 | SSIPEDVIAATFNMERNEARKLKSSNPFTFLV | 3562.36 | C160 H254 N43 O49 S1 |
| BY Int Fragment | 0 | 3607.8607 | 3607.8577 | 142 | 173 | 0.8 | LSSIPEDVIAATFNMERNEARKLKSSNPFTFL | 1022.45 | C161 H256 N43 O49 S1 |
| BY Int Fragment | 0 | 3694.8858 | 3694.8898 | 141 | 173 | -1.1 | SLSSIPEDVIAATFNMERNEARKLKSSNPFTFL | 613.65 | C164 H261 N44 O51 S1 |
| BY Int Fragment | 0 | 3700.9334 | 3700.9156 | 144 | 176 | 4.8 | SIPEDVIAATFNMERNEARKLKSSNPFTFLVPP | 218.3 | C167 H263 N44 O49 S1 |
| BY Int Fragment | 0 | 3880.9884 | 3880.9902 | 140 | 174 | -0.5 | SSLSSIPEDVIAATFNMERNEARKLKSSNPFTFLV | 708.38 | C172 H275 N46 O54 S1 |
| BY Int Fragment | 57.0215 | 3902.9366 | 3902.9342 | 4 | 38 | 0.6 | ETICTARLHQNIDSSSSPDIYIPQAGRIKTVTSFD | 1511.88 | C166 H266 N47 O56 S1 |
| BY Int Fragment | 57.0215 | 4031.9759 | 4031.9768 | 3 | 38 | -0.2 | EETICTARLHQNIDSSSSPDIYIPQAGRIKTVTSFD | 4115.32 | C171 H273 N48 O59 S1 |
| BY Int Fragment | 0 | 4088.1107 | 4088.1022 | 105 | 143 | 2.1 | PQNFAVAAKSMSDRFSYVAFKTNDRAAIGKLVGATSSLS | 1405.8 | C181 H288 N51 O55 S1 |
| BY Int Fragment | 0 | 4175.1277 | 4175.1343 | 105 | 144 | -1.6 | PQNFAVAAKSMSDRFSYVAFKTNDRAAIGKLVGATSSLSS | 2123.98 | C184 H293 N52 O57 S1 |
| BY Int Fragment | 0 | 4288.2246 | 4288.2183 | 105 | 145 | 1.5 | PQNFAVAAKSMSDRFSYVAFKTNDRAAIGKLVGATSSLSSI | 367.6 | C190 H304 N53 O58 S1 |
| BY Int Fragment | 0 | 4288.2246 | 4288.2183 | 106 | 146 | 1.5 | QNFAVAAKSMSDRFSYVAFKTNDRAAIGKLVGATSSLSSIP | 367.6 | C190 H304 N53 O58 S1 |
| BY Int Fragment | 0 | 4368.2507 | 4368.2194 | 93 | 133 | 7.2 | DGELEAGRALIVPQNFAVAAKSMSDRFSYVAFKTNDRAAIG | 1798.14 | C193 H304 N55 O59 S1 |
| BY Int Fragment | 57.0215 | 5020.5349 | 5020.5323 | 48 | 92 | 0.5 | SAEHGSLHKNAMFVPHYNLNANSILYALKGRARLQVVNCNGNNVF | 263.47 | C219 H341 N68 O61 S2 |
| BY Int Fragment | 0 | 5079.6675 | 5079.6513 | 15 | 60 | 3.2 | IDSSSSPDIYIPQAGRIKTVTSFDLPALRWLKLSAEHGSLHKNAMF | 798.98 | C230 H361 N62 O66 S1 |
| BY Int Fragment | 57.0215 | 5157.6969 | 5157.6837 | 69 | 116 | 2.5 | NSILYALKGRARLQVVNCNGNNVFDGELEAGRALIVPQNFAVAAKSMS | 176.36 | C223 H364 N67 O66 S2 |
| BY Int Fragment | 57.0215 | 5166.6029 | 5166.6014 | 55 | 100 | 0.3 | HKNAMFVPHYNLNANSILYALKGRARLQVVNCNGNNVFDGELEAGR | 71.96 | C224 H351 N70 O64 S2 |
| BY Int Fragment | 0 | 5246.7346 | 5246.7837 | 28 | 74 | -9.4 | AGRIKTVTSFDLPALRWLKLSAEHGSLHKNAMFVPHYNLNANSILYA | 544.75 | C241 H374 N67 O63 S1 |
| BY Int Fragment | 0 | 5246.7346 | 5246.7837 | 33 | 79 | -9.4 | TVTSFDLPALRWLKLSAEHGSLHKNAMFVPHYNLNANSILYALKGRA | 544.75 | C241 H374 N67 O63 S1 |
| BY Int Fragment | 0 | 5371.7845 | 5371.7896 | 92 | 142 | -1 | FDGELEAGRALIVPQNFAVAAKSMSDRFSYVAFKTNDRAAIGKLVGATSSL | 1893.08 | C240 H381 N66 O72 S1 |
| BY Int Fragment | 57.0215 | 5374.8086 | 5374.8053 | 71 | 119 | 0.6 | ILYALKGRARLQVVNCNGNNVFDGELEAGRALIVPQNFAVAAKSMSDRF | 122.61 | C235 H379 N70 O67 S2 |
| BY Int Fragment | 0 | 5458.804 | 5458.8117 | 12 | 60 | -1.4 | HQNIDSSSSPDIYIPQAGRIKTVTSFDLPALRWLKLSAEHGSLHKNAMF | 979.3 | C245 H382 N69 O71 S1 |
| BY Int Fragment | 0 | 5545.8506 | 5545.8537 | 92 | 144 | -0.6 | FDGELEAGRALIVPQNFAVAAKSMSDRFSYVAFKTNDRAAIGKLVGATSSLSS | 5481.06 | C246 H391 N68 O76 S1 |
| BY Int Fragment | 57.0215 | 5609.9273 | 5609.9009 | 75 | 125 | 4.7 | LKGRARLQVVNCNGNNVFDGELEAGRALIVPQNFAVAAKSMSDRFSYVAFK | 730.27 | C246 H392 N73 O70 S2 |
| BY Int Fragment | 57.0215 | 5610.9316 | 5610.885 | 72 | 122 | 8.3 | LYALKGRARLQVVNCNGNNVFDGELEAGRALIVPQNFAVAAKSMSDRFSYV | 378.34 | C246 H391 N72 O71 S2 |
| BY Int Fragment | 0 | 5612.9401 | 5612.9376 | 20 | 69 | 0.4 | SPDIYIPQAGRIKTVTSFDLPALRWLKLSAEHGSLHKNAMFVPHYNLNAN | 316.42 | C257 H396 N71 O69 S1 |
| BY Int Fragment | 0 | 5612.9401 | 5612.9376 | 21 | 70 | 0.4 | PDIYIPQAGRIKTVTSFDLPALRWLKLSAEHGSLHKNAMFVPHYNLNANS | 316.42 | C257 H396 N71 O69 S1 |
| BY Int Fragment | 0 | 5658.9365 | 5658.9377 | 92 | 145 | -0.2 | FDGELEAGRALIVPQNFAVAAKSMSDRFSYVAFKTNDRAAIGKLVGATSSLSSI | 5775.02 | C252 H402 N69 O77 S1 |
| BY Int Fragment | 57.0215 | 5825.0326 | 5824.9916 | 75 | 127 | 7 | LKGRARLQVVNCNGNNVFDGELEAGRALIVPQNFAVAAKSMSDRFSYVAFKTN | 319.5 | C254 H405 N76 O74 S2 |
| BY Int Fragment | 57.0215 | 5840.0443 | 5840.0065 | 58 | 110 | 6.5 | AMFVPHYNLNANSILYALKGRARLQVVNCNGNNVFDGELEAGRALIVPQNFAV | 897.32 | C259 H406 N75 O72 S2 |
| BY Int Fragment | 57.0215 | 5840.0443 | 5840.0065 | 59 | 111 | 6.5 | MFVPHYNLNANSILYALKGRARLQVVNCNGNNVFDGELEAGRALIVPQNFAVA | 897.32 | C259 H406 N75 O72 S2 |
| BY Int Fragment | 0 | 7107.6849 | 7107.6823 | 109 | 174 | 0.4 | AVAAKSMSDRFSYVAFKTNDRAAIGKLVGATSSLSSIPEDVIAATFNMERNEARKLKSSNPFTFLV | 714.34 | C315 H506 N87 O96 S2 |
| BY Int Fragment | 0 | 7233.6741 | 7233.6889 | 114 | 179 | -2 | SMSDRFSYVAFKTNDRAAIGKLVGATSSLSSIPEDVIAATFNMERNEARKLKSSNPFTFLVPPRES | 388.51 | C319 H508 N89 O99 S2 |
| BY Int Fragment | 0 | 7240.7888 | 7240.8151 | 100 | 166 | -3.6 | RALIVPQNFAVAAKSMSDRFSYVAFKTNDRAAIGKLVGATSSLSSIPEDVIAATFNMERNEARKLKS | 1124.25 | C319 H521 N92 O96 S2 |
| BY Int Fragment | 0 | 7297.8265 | 7297.8365 | 99 | 166 | -1.4 | GRALIVPQNFAVAAKSMSDRFSYVAFKTNDRAAIGKLVGATSSLSSIPEDVIAATFNMERNEARKLKS | 446.39 | C321 H524 N93 O97 S2 |
| BY Int Fragment | 57.0215 | 7336.868 | 7336.874 | 71 | 138 | -0.8 | ILYALKGRARLQVVNCNGNNVFDGELEAGRALIVPQNFAVAAKSMSDRFSYVAFKTNDRAAIGKLVGA | 185.34 | C324 H522 N95 O92 S2 |
| BY Int Fragment | 0 | 7381.7544 | 7381.7525 | 105 | 172 | 0.3 | PQNFAVAAKSMSDRFSYVAFKTNDRAAIGKLVGATSSLSSIPEDVIAATFNMERNEARKLKSSNPFTF | 6859.58 | C327 H516 N91 O100 S2 |
| BY Int Fragment | 57.0215 | 7440.7811 | 7440.7798 | 3 | 68 | 0.2 | EETICTARLHQNIDSSSSPDIYIPQAGRIKTVTSFDLPALRWLKLSAEHGSLHKNAMFVPHYNLNA | 398.89 | C329 H514 N93 O97 S2 |
| BY Int Fragment | 57.0215 | 7572.8216 | 7572.8319 | 83 | 154 | -1.4 | VVNCNGNNVFDGELEAGRALIVPQNFAVAAKSMSDRFSYVAFKTNDRAAIGKLVGATSSLSSIPEDVIAATF | 386.49 | C332 H526 N91 O104 S2 |
| BY Int Fragment | 57.0215 | 7575.8871 | 7575.8529 | 54 | 121 | 4.5 | LHKNAMFVPHYNLNANSILYALKGRARLQVVNCNGNNVFDGELEAGRALIVPQNFAVAAKSMSDRFSY | 1066.94 | C335 H523 N98 O94 S3 |
| BY Int Fragment | 0 | 7593.9002 | 7593.905 | 103 | 172 | -0.6 | IVPQNFAVAAKSMSDRFSYVAFKTNDRAAIGKLVGATSSLSSIPEDVIAATFNMERNEARKLKSSNPFTF | 26267.82 | C338 H536 N93 O102 S2 |
| BY Int Fragment | 0 | 7593.9002 | 7593.905 | 104 | 173 | -0.6 | VPQNFAVAAKSMSDRFSYVAFKTNDRAAIGKLVGATSSLSSIPEDVIAATFNMERNEARKLKSSNPFTFL | 26267.82 | C338 H536 N93 O102 S2 |
| BY Int Fragment | 0 | 7593.9002 | 7593.905 | 105 | 174 | -0.6 | PQNFAVAAKSMSDRFSYVAFKTNDRAAIGKLVGATSSLSSIPEDVIAATFNMERNEARKLKSSNPFTFLV | 26267.82 | C338 H536 N93 O102 S2 |
| BY Int Fragment | 0 | 7593.9002 | 7593.905 | 106 | 175 | -0.6 | QNFAVAAKSMSDRFSYVAFKTNDRAAIGKLVGATSSLSSIPEDVIAATFNMERNEARKLKSSNPFTFLVP | 26267.82 | C338 H536 N93 O102 S2 |
| BY Int Fragment | 0 | 7692.9669 | 7692.9734 | 104 | 174 | -0.9 | VPQNFAVAAKSMSDRFSYVAFKTNDRAAIGKLVGATSSLSSIPEDVIAATFNMERNEARKLKSSNPFTFLV | 1893.64 | C343 H545 N94 O103 S2 |
| BY Int Fragment | 57.0215 | 7700.8887 | 7700.8905 | 82 | 154 | -0.2 | QVVNCNGNNVFDGELEAGRALIVPQNFAVAAKSMSDRFSYVAFKTNDRAAIGKLVGATSSLSSIPEDVIAATF | 238.43 | C337 H534 N93 O106 S2 |
| BY Int Fragment | 0 | 7706.9847 | 7706.9891 | 102 | 172 | -0.6 | LIVPQNFAVAAKSMSDRFSYVAFKTNDRAAIGKLVGATSSLSSIPEDVIAATFNMERNEARKLKSSNPFTF | 1652.32 | C344 H547 N94 O103 S2 |
| BY Int Fragment | 0 | 7706.9847 | 7706.9891 | 103 | 173 | -0.6 | IVPQNFAVAAKSMSDRFSYVAFKTNDRAAIGKLVGATSSLSSIPEDVIAATFNMERNEARKLKSSNPFTFL | 1652.32 | C344 H547 N94 O103 S2 |
| BY Int Fragment | 57.0215 | 7725.952 | 7726.0286 | 69 | 140 | -9.9 | NSILYALKGRARLQVVNCNGNNVFDGELEAGRALIVPQNFAVAAKSMSDRFSYVAFKTNDRAAIGKLVGATS | 297.56 | C338 H545 N100 O100 S2 |
| BY Int Fragment | 0 | 7785.1244 | 7785.1391 | 14 | 83 | -1.9 | NIDSSSSPDIYIPQAGRIKTVTSFDLPALRWLKLSAEHGSLHKNAMFVPHYNLNANSILYALKGRARLQV | 264.19 | C352 H557 N100 O98 S1 |
| BY Int Fragment | 57.0215 | 7807.0646 | 7807.0236 | 78 | 150 | 5.2 | RARLQVVNCNGNNVFDGELEAGRALIVPQNFAVAAKSMSDRFSYVAFKTNDRAAIGKLVGATSSLSSIPEDVI | 1615.58 | C339 H548 N99 O105 S2 |
| BY Int Fragment | 57.0215 | 7824.1006 | 7824.0766 | 67 | 139 | 3.1 | NANSILYALKGRARLQVVNCNGNNVFDGELEAGRALIVPQNFAVAAKSMSDRFSYVAFKTNDRAAIGKLVGAT | 692.15 | C342 H551 N102 O101 S2 |
| BY Int Fragment | 57.0215 | 7901.1683 | 7901.2065 | 23 | 92 | -4.8 | IYIPQAGRIKTVTSFDLPALRWLKLSAEHGSLHKNAMFVPHYNLNANSILYALKGRARLQVVNCNGNNVF | 250.2 | C357 H562 N103 O93 S2 |
| BY Int Fragment | 0 | 7915.1496 | 7915.1175 | 98 | 171 | 4.1 | AGRALIVPQNFAVAAKSMSDRFSYVAFKTNDRAAIGKLVGATSSLSSIPEDVIAATFNMERNEARKLKSSNPFT | 260.96 | C349 H563 N100 O106 S2 |
| BY Int Fragment | 57.0215 | 7923.1411 | 7923.1504 | 41 | 112 | -1.2 | ALRWLKLSAEHGSLHKNAMFVPHYNLNANSILYALKGRARLQVVNCNGNNVFDGELEAGRALIVPQNFAVAA | 1065.75 | C353 H556 N105 O96 S2 |
| BY Int Fragment | 57.0215 | 7980.1902 | 7980.2083 | 42 | 113 | -2.3 | LRWLKLSAEHGSLHKNAMFVPHYNLNANSILYALKGRARLQVVNCNGNNVFDGELEAGRALIVPQNFAVAAK | 279.82 | C356 H563 N106 O96 S2 |
| BY Int Fragment | 0 | 7998.2016 | 7998.2505 | 14 | 85 | -6.1 | NIDSSSSPDIYIPQAGRIKTVTSFDLPALRWLKLSAEHGSLHKNAMFVPHYNLNANSILYALKGRARLQVVN | 693.92 | C361 H572 N103 O101 S1 |
| BY Int Fragment | 0 | 8175.214 | 8175.2699 | 98 | 173 | -6.8 | AGRALIVPQNFAVAAKSMSDRFSYVAFKTNDRAAIGKLVGATSSLSSIPEDVIAATFNMERNEARKLKSSNPFTFL | 1586.62 | C364 H583 N102 O108 S2 |
| BY Int Fragment | 0 | 8191.2301 | 8191.2285 | 97 | 172 | 0.2 | EAGRALIVPQNFAVAAKSMSDRFSYVAFKTNDRAAIGKLVGATSSLSSIPEDVIAATFNMERNEARKLKSSNPFTF | 1006.87 | C363 H579 N102 O110 S2 |
| BY Int Fragment | 0 | 8289.2593 | 8289.2289 | 105 | 180 | 3.7 | PQNFAVAAKSMSDRFSYVAFKTNDRAAIGKLVGATSSLSSIPEDVIAATFNMERNEARKLKSSNPFTFLVPPRESE | 1254.97 | C367 H581 N102 O113 S2 |
| BY Int Fragment | 0 | 8304.3161 | 8304.3125 | 96 | 172 | 0.4 | LEAGRALIVPQNFAVAAKSMSDRFSYVAFKTNDRAAIGKLVGATSSLSSIPEDVIAATFNMERNEARKLKSSNPFTF | 2988.3 | C369 H590 N103 O111 S2 |
| BY Int Fragment | 0 | 8304.3161 | 8304.3125 | 97 | 173 | 0.4 | EAGRALIVPQNFAVAAKSMSDRFSYVAFKTNDRAAIGKLVGATSSLSSIPEDVIAATFNMERNEARKLKSSNPFTFL | 2988.3 | C369 H590 N103 O111 S2 |
| BY Int Fragment | 0 | 8403.4051 | 8403.3809 | 97 | 174 | 2.9 | EAGRALIVPQNFAVAAKSMSDRFSYVAFKTNDRAAIGKLVGATSSLSSIPEDVIAATFNMERNEARKLKSSNPFTFLV | 2383.09 | C374 H599 N104 O112 S2 |
| BY Int Fragment | 0 | 8490.376 | 8490.3766 | 94 | 172 | -0.1 | GELEAGRALIVPQNFAVAAKSMSDRFSYVAFKTNDRAAIGKLVGATSSLSSIPEDVIAATFNMERNEARKLKSSNPFTF | 1429.94 | C376 H600 N105 O115 S2 |
| BY Int Fragment | 0 | 8516.4532 | 8516.465 | 96 | 174 | -1.4 | LEAGRALIVPQNFAVAAKSMSDRFSYVAFKTNDRAAIGKLVGATSSLSSIPEDVIAATFNMERNEARKLKSSNPFTFLV | 1721.86 | C380 H610 N105 O113 S2 |
| BY Int Fragment | 57.0215 | 8517.3945 | 8517.3248 | 51 | 127 | 8.2 | HGSLHKNAMFVPHYNLNANSILYALKGRARLQVVNCNGNNVFDGELEAGRALIVPQNFAVAAKSMSDRFSYVAFKTN | 111.48 | C377 H586 N111 O106 S3 |
| BY Int Fragment | 57.0215 | 8701.4736 | 8701.5253 | 19 | 96 | -5.9 | SSPDIYIPQAGRIKTVTSFDLPALRWLKLSAEHGSLHKNAMFVPHYNLNANSILYALKGRARLQVVNCNGNNVFDGEL | 2438.54 | C389 H610 N111 O109 S2 |
| BY Int Fragment | 0 | 8718.484 | 8718.4876 | 93 | 173 | -0.4 | DGELEAGRALIVPQNFAVAAKSMSDRFSYVAFKTNDRAAIGKLVGATSSLSSIPEDVIAATFNMERNEARKLKSSNPFTFL | 7789.87 | C386 H616 N107 O119 S2 |
| BY Int Fragment | 0 | 8752.4718 | 8752.4719 | 92 | 172 | 0 | FDGELEAGRALIVPQNFAVAAKSMSDRFSYVAFKTNDRAAIGKLVGATSSLSSIPEDVIAATFNMERNEARKLKSSNPFTF | 14870.37 | C389 H614 N107 O119 S2 |
| BY Int Fragment | 0 | 8799.5087 | 8799.5818 | 94 | 175 | -8.3 | GELEAGRALIVPQNFAVAAKSMSDRFSYVAFKTNDRAAIGKLVGATSSLSSIPEDVIAATFNMERNEARKLKSSNPFTFLVP | 295.37 | C392 H627 N108 O118 S2 |
| BY Int Fragment | 57.0215 | 8863.581 | 8863.6482 | 10 | 88 | -7.6 | RLHQNIDSSSSPDIYIPQAGRIKTVTSFDLPALRWLKLSAEHGSLHKNAMFVPHYNLNANSILYALKGRARLQVVNCNG | 602.32 | C393 H624 N117 O110 S2 |
| BY Int Fragment | 0 | 8865.5223 | 8865.556 | 92 | 173 | -3.8 | FDGELEAGRALIVPQNFAVAAKSMSDRFSYVAFKTNDRAAIGKLVGATSSLSSIPEDVIAATFNMERNEARKLKSSNPFTFL | 40918.35 | C395 H625 N108 O120 S2 |
| BY Int Fragment | 0 | 8964.6346 | 8964.6244 | 91 | 173 | 1.1 | VFDGELEAGRALIVPQNFAVAAKSMSDRFSYVAFKTNDRAAIGKLVGATSSLSSIPEDVIAATFNMERNEARKLKSSNPFTFL | 37983.38 | C400 H634 N109 O121 S2 |
| BY Int Fragment | 0 | 8964.6346 | 8964.6244 | 92 | 174 | 1.1 | FDGELEAGRALIVPQNFAVAAKSMSDRFSYVAFKTNDRAAIGKLVGATSSLSSIPEDVIAATFNMERNEARKLKSSNPFTFLV | 37983.38 | C400 H634 N109 O121 S2 |
| BY Int Fragment | 57.0215 | 9510.8576 | 9510.8762 | 76 | 163 | -2 | KGRARLQVVNCNGNNVFDGELEAGRALIVPQNFAVAAKSMSDRFSYVAFKTNDRAAIGKLVGATSSLSSIPEDVIAATFNMERNEARK | 224.39 | C410 H665 N124 O127 S3 |
| BY Int Fragment | 114.043 | 9643.9278 | 9643.9846 | 2 | 86 | -5.9 | FEETICTARLHQNIDSSSSPDIYIPQAGRIKTVTSFDLPALRWLKLSAEHGSLHKNAMFVPHYNLNANSILYALKGRARLQVVNC | 2047.69 | C426 H673 N122 O121 S3 |
| BY Int Fragment | 0 | 9659.9386 | 9659.9483 | 92 | 180 | -1 | FDGELEAGRALIVPQNFAVAAKSMSDRFSYVAFKTNDRAAIGKLVGATSSLSSIPEDVIAATFNMERNEARKLKSSNPFTFLVPPRESE | 3526.3 | C429 H679 N118 O132 S2 |
| BY Int Fragment | 57.0215 | 9675.0389 | 9675.0181 | 61 | 150 | 2.2 | VPHYNLNANSILYALKGRARLQVVNCNGNNVFDGELEAGRALIVPQNFAVAAKSMSDRFSYVAFKTNDRAAIGKLVGATSSLSSIPEDVI | 273.72 | C426 H681 N122 O128 S2 |
| BY Int Fragment | 57.0215 | 9696.9718 | 9696.9847 | 58 | 147 | -1.3 | AMFVPHYNLNANSILYALKGRARLQVVNCNGNNVFDGELEAGRALIVPQNFAVAAKSMSDRFSYVAFKTNDRAAIGKLVGATSSLSSIPE | 151.95 | C428 H679 N122 O126 S3 |
| BY Int Fragment | 57.0215 | 9724.9799 | 9724.9874 | 11 | 97 | -0.8 | LHQNIDSSSSPDIYIPQAGRIKTVTSFDLPALRWLKLSAEHGSLHKNAMFVPHYNLNANSILYALKGRARLQVVNCNGNNVFDGELE | 296.06 | C431 H675 N124 O126 S2 |
| BY Int Fragment | 57.0215 | 9731.9939 | 9732.0283 | 64 | 154 | -3.5 | YNLNANSILYALKGRARLQVVNCNGNNVFDGELEAGRALIVPQNFAVAAKSMSDRFSYVAFKTNDRAAIGKLVGATSSLSSIPEDVIAATF | 287.95 | C429 H684 N121 O130 S2 |
| BY Int Fragment | 57.0215 | 9740.0109 | 9739.9619 | 12 | 99 | 5 | HQNIDSSSSPDIYIPQAGRIKTVTSFDLPALRWLKLSAEHGSLHKNAMFVPHYNLNANSILYALKGRARLQVVNCNGNNVFDGELEAG | 948.88 | C430 H672 N125 O127 S2 |
| BY Int Fragment | 57.0215 | 9799.0028 | 9799.0289 | 50 | 139 | -2.7 | EHGSLHKNAMFVPHYNLNANSILYALKGRARLQVVNCNGNNVFDGELEAGRALIVPQNFAVAAKSMSDRFSYVAFKTNDRAAIGKLVGAT | 396.3 | C432 H681 N128 O124 S3 |
| BY Int Fragment | 57.0215 | 9822.0143 | 9822.0865 | 60 | 150 | -7.4 | FVPHYNLNANSILYALKGRARLQVVNCNGNNVFDGELEAGRALIVPQNFAVAAKSMSDRFSYVAFKTNDRAAIGKLVGATSSLSSIPEDVI | 128.05 | C435 H690 N123 O129 S2 |
| BY Int Fragment | 114.043 | 9842.0261 | 9842.0348 | 7 | 94 | -0.9 | CTARLHQNIDSSSSPDIYIPQAGRIKTVTSFDLPALRWLKLSAEHGSLHKNAMFVPHYNLNANSILYALKGRARLQVVNCNGNNVFDG | 713.06 | C431 H679 N128 O124 S3 |
| BY Int Fragment | 0 | 9858.0396 | 9858.06 | 89 | 179 | -2.1 | NNVFDGELEAGRALIVPQNFAVAAKSMSDRFSYVAFKTNDRAAIGKLVGATSSLSSIPEDVIAATFNMERNEARKLKSSNPFTFLVPPRES | 791.76 | C437 H693 N122 O134 S2 |
| BY Int Fragment | 57.0215 | 9869.0465 | 9869.0872 | 63 | 154 | -4.1 | HYNLNANSILYALKGRARLQVVNCNGNNVFDGELEAGRALIVPQNFAVAAKSMSDRFSYVAFKTNDRAAIGKLVGATSSLSSIPEDVIAATF | 1711.29 | C435 H691 N124 O131 S2 |
| BY Int Fragment | 114.043 | 9884.052 | 9884.1181 | 5 | 92 | -6.7 | TICTARLHQNIDSSSSPDIYIPQAGRIKTVTSFDLPALRWLKLSAEHGSLHKNAMFVPHYNLNANSILYALKGRARLQVVNCNGNNVF | 331.65 | C435 H689 N128 O123 S3 |
| BY Int Fragment | 57.0215 | 9896.0311 | 9896.0631 | 12 | 100 | -3.2 | HQNIDSSSSPDIYIPQAGRIKTVTSFDLPALRWLKLSAEHGSLHKNAMFVPHYNLNANSILYALKGRARLQVVNCNGNNVFDGELEAGR | 872.69 | C436 H684 N129 O128 S2 |
| BY Int Fragment | 57.0215 | 9911.0137 | 9911.08 | 58 | 149 | -6.7 | AMFVPHYNLNANSILYALKGRARLQVVNCNGNNVFDGELEAGRALIVPQNFAVAAKSMSDRFSYVAFKTNDRAAIGKLVGATSSLSSIPEDV | 225.29 | C437 H693 N124 O130 S3 |
| BY Int Fragment | 57.0215 | 9911.0395 | 9911.08 | 58 | 149 | -4.1 | AMFVPHYNLNANSILYALKGRARLQVVNCNGNNVFDGELEAGRALIVPQNFAVAAKSMSDRFSYVAFKTNDRAAIGKLVGATSSLSSIPEDV | 564.09 | C437 H693 N124 O130 S3 |
| BY Int Fragment | 57.0215 | 9935.0652 | 9935.0535 | 83 | 174 | 1.2 | VVNCNGNNVFDGELEAGRALIVPQNFAVAAKSMSDRFSYVAFKTNDRAAIGKLVGATSSLSSIPEDVIAATFNMERNEARKLKSSNPFTFLV | 258.09 | C436 H693 N122 O134 S3 |
| BY Int Fragment | 57.0215 | 9943.0842 | 9943.091 | 65 | 157 | -0.7 | NLNANSILYALKGRARLQVVNCNGNNVFDGELEAGRALIVPQNFAVAAKSMSDRFSYVAFKTNDRAAIGKLVGATSSLSSIPEDVIAATFNME | 338.32 | C434 H697 N124 O134 S3 |
| BY Int Fragment | 57.0215 | 9967.1118 | 9967.1002 | 12 | 101 | 1.2 | HQNIDSSSSPDIYIPQAGRIKTVTSFDLPALRWLKLSAEHGSLHKNAMFVPHYNLNANSILYALKGRARLQVVNCNGNNVFDGELEAGRA | 1123.37 | C439 H689 N130 O129 S2 |
| BY Int Fragment | 114.043 | 9971.0851 | 9971.0774 | 7 | 95 | 0.8 | CTARLHQNIDSSSSPDIYIPQAGRIKTVTSFDLPALRWLKLSAEHGSLHKNAMFVPHYNLNANSILYALKGRARLQVVNCNGNNVFDGE | 420.38 | C436 H686 N129 O127 S3 |
| BY Int Fragment | 57.0215 | 9984.0782 | 9984.1381 | 33 | 122 | -6 | TVTSFDLPALRWLKLSAEHGSLHKNAMFVPHYNLNANSILYALKGRARLQVVNCNGNNVFDGELEAGRALIVPQNFAVAAKSMSDRFSYV | 556.68 | C446 H696 N127 O125 S3 |
| BY Int Fragment | 114.043 | 10013.1271 | 10013.1607 | 4 | 92 | -3.4 | ETICTARLHQNIDSSSSPDIYIPQAGRIKTVTSFDLPALRWLKLSAEHGSLHKNAMFVPHYNLNANSILYALKGRARLQVVNCNGNNVF | 675.54 | C440 H696 N129 O126 S3 |
| BY Int Fragment | 57.0215 | 10027.0737 | 10027.1664 | 41 | 130 | -9.2 | ALRWLKLSAEHGSLHKNAMFVPHYNLNANSILYALKGRARLQVVNCNGNNVFDGELEAGRALIVPQNFAVAAKSMSDRFSYVAFKTNDRA | 870 | C445 H697 N132 O124 S3 |
| BY Int Fragment | 57.0215 | 10027.0737 | 10027.1664 | 42 | 131 | -9.2 | LRWLKLSAEHGSLHKNAMFVPHYNLNANSILYALKGRARLQVVNCNGNNVFDGELEAGRALIVPQNFAVAAKSMSDRFSYVAFKTNDRAA | 870 | C445 H697 N132 O124 S3 |
| BY Int Fragment | 57.0215 | 10027.0737 | 10027.1664 | 43 | 132 | -9.2 | RWLKLSAEHGSLHKNAMFVPHYNLNANSILYALKGRARLQVVNCNGNNVFDGELEAGRALIVPQNFAVAAKSMSDRFSYVAFKTNDRAAI | 870 | C445 H697 N132 O124 S3 |
| BY Int Fragment | 57.0215 | 10030.1049 | 10030.0906 | 84 | 176 | 1.4 | VNCNGNNVFDGELEAGRALIVPQNFAVAAKSMSDRFSYVAFKTNDRAAIGKLVGATSSLSSIPEDVIAATFNMERNEARKLKSSNPFTFLVPP | 198.57 | C441 H698 N123 O135 S3 |
| BY Int Fragment | 57.0215 | 10040.146 | 10040.1622 | 76 | 168 | -1.6 | KGRARLQVVNCNGNNVFDGELEAGRALIVPQNFAVAAKSMSDRFSYVAFKTNDRAAIGKLVGATSSLSSIPEDVIAATFNMERNEARKLKSSN | 580.12 | C432 H704 N131 O135 S3 |
| BY Int Fragment | 114.043 | 10056.1153 | 10056.1665 | 5 | 94 | -5.1 | TICTARLHQNIDSSSSPDIYIPQAGRIKTVTSFDLPALRWLKLSAEHGSLHKNAMFVPHYNLNANSILYALKGRARLQVVNCNGNNVFDG | 2296.37 | C441 H697 N130 O127 S3 |
| BY Int Fragment | 57.0215 | 10077.1279 | 10077.1278 | 81 | 173 | 0 | LQVVNCNGNNVFDGELEAGRALIVPQNFAVAAKSMSDRFSYVAFKTNDRAAIGKLVGATSSLSSIPEDVIAATFNMERNEARKLKSSNPFTFL | 429.07 | C442 H703 N124 O136 S3 |
| BY Int Fragment | 57.0215 | 10086.1437 | 10086.177 | 50 | 142 | -3.3 | EHGSLHKNAMFVPHYNLNANSILYALKGRARLQVVNCNGNNVFDGELEAGRALIVPQNFAVAAKSMSDRFSYVAFKTNDRAAIGKLVGATSSL | 383.99 | C444 H702 N131 O129 S3 |
| BY Int Fragment | 57.0215 | 10099.1294 | 10099.1669 | 78 | 170 | -3.7 | RARLQVVNCNGNNVFDGELEAGRALIVPQNFAVAAKSMSDRFSYVAFKTNDRAAIGKLVGATSSLSSIPEDVIAATFNMERNEARKLKSSNPF | 1386.91 | C438 H705 N130 O135 S3 |
| BY Int Fragment | 57.0215 | 10106.1432 | 10106.1543 | 64 | 157 | -1.1 | YNLNANSILYALKGRARLQVVNCNGNNVFDGELEAGRALIVPQNFAVAAKSMSDRFSYVAFKTNDRAAIGKLVGATSSLSSIPEDVIAATFNME | 263.66 | C443 H706 N125 O136 S3 |
| BY Int Fragment | 57.0215 | 10129.1399 | 10129.1591 | 83 | 176 | -1.9 | VVNCNGNNVFDGELEAGRALIVPQNFAVAAKSMSDRFSYVAFKTNDRAAIGKLVGATSSLSSIPEDVIAATFNMERNEARKLKSSNPFTFLVPP | 553.56 | C446 H707 N124 O136 S3 |
| BY Int Fragment | 57.0215 | 10138.1171 | 10138.207 | 57 | 150 | -8.9 | NAMFVPHYNLNANSILYALKGRARLQVVNCNGNNVFDGELEAGRALIVPQNFAVAAKSMSDRFSYVAFKTNDRAAIGKLVGATSSLSSIPEDVI | 144.16 | C447 H710 N127 O133 S3 |
| BY Int Fragment | 57.0215 | 10156.2463 | 10156.1884 | 77 | 170 | 5.7 | GRARLQVVNCNGNNVFDGELEAGRALIVPQNFAVAAKSMSDRFSYVAFKTNDRAAIGKLVGATSSLSSIPEDVIAATFNMERNEARKLKSSNPF | 545.3 | C440 H708 N131 O136 S3 |
| BY Int Fragment | 57.0215 | 10209.2233 | 10209.2441 | 57 | 151 | -2 | NAMFVPHYNLNANSILYALKGRARLQVVNCNGNNVFDGELEAGRALIVPQNFAVAAKSMSDRFSYVAFKTNDRAAIGKLVGATSSLSSIPEDVIA | 290.48 | C450 H715 N128 O134 S3 |
| BY Int Fragment | 114.043 | 10213.164 | 10213.204 | 7 | 97 | -3.9 | CTARLHQNIDSSSSPDIYIPQAGRIKTVTSFDLPALRWLKLSAEHGSLHKNAMFVPHYNLNANSILYALKGRARLQVVNCNGNNVFDGELE | 705.48 | C447 H704 N131 O131 S3 |
| BY Int Fragment | 57.0215 | 10231.2017 | 10231.2702 | 35 | 126 | -6.7 | TSFDLPALRWLKLSAEHGSLHKNAMFVPHYNLNANSILYALKGRARLQVVNCNGNNVFDGELEAGRALIVPQNFAVAAKSMSDRFSYVAFKT | 1156.28 | C459 H713 N130 O127 S3 |

Top-down characterization of the component with the experimental mass 19964.2195 Da was achieved by the MS/MS of its multi-charged ion [M+23H]^23+^, at m/z 869.5397, interpreted by the ClipsMS tool using the criteria above reported for internal fragments. This polypeptide was identified with the region Gly334-Asn514 of the legumin A-like entry with Acc. No. A0A1S2XSB9 carrying the Cys340 in the carbamidomethylated form and the Cys419 as sulfinic acid. Figure S4a shows the corresponding deconvoluted MS/MS and reports the attributions of the most intense peaks. In this case, the Gly334-Asn514 region carrying the Cys340 as carbamidomethyl-cysteine, and the Cys419 as sulfinic acid, was used as a reference sequence and renumbered as explained above (see Figure S4b). In detail, this MS/MS shows a peak at m/z 10075.1049 that corresponds to the b90 fragment, which includes both the cysteine residues, one as carbamidomethyl-cysteine and the other as sulfinic acid. Another group of signals was assigned to putative internal fragments. This group includes the peaks at m/z 2216.0016, 5527.8426, 7842.1177, 9870.0262, and 10173.1727, which can be related to the putative internal fragments Ile72-Val91, Ala74-Phe124, Pro21-Asn90, Leu39-Asn127, and Lys46-Thr139, carrying the cysteine 86 (i.e., the cysteine at position 419 in the entire sequence entry) as sulfinic acid.


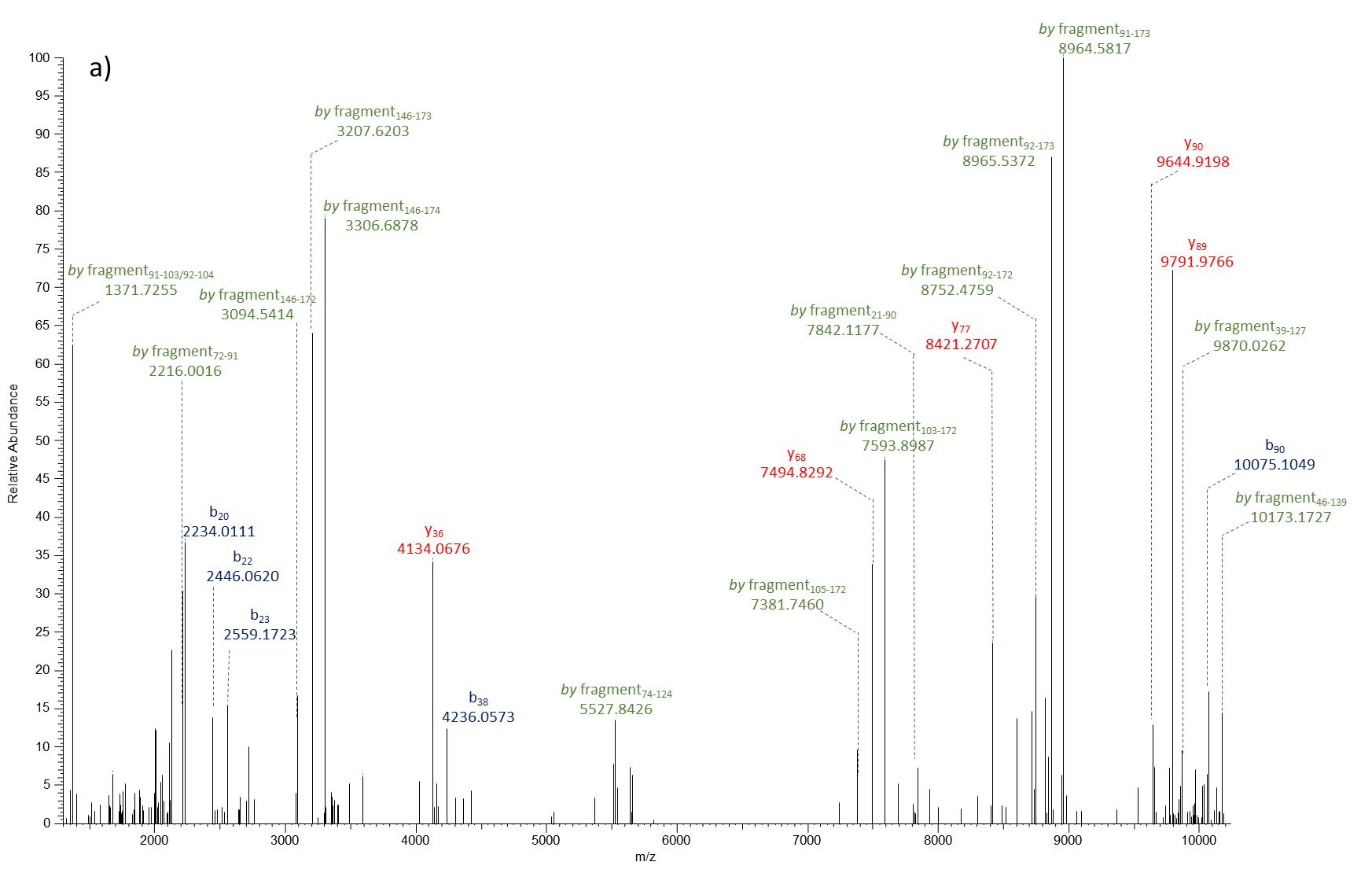


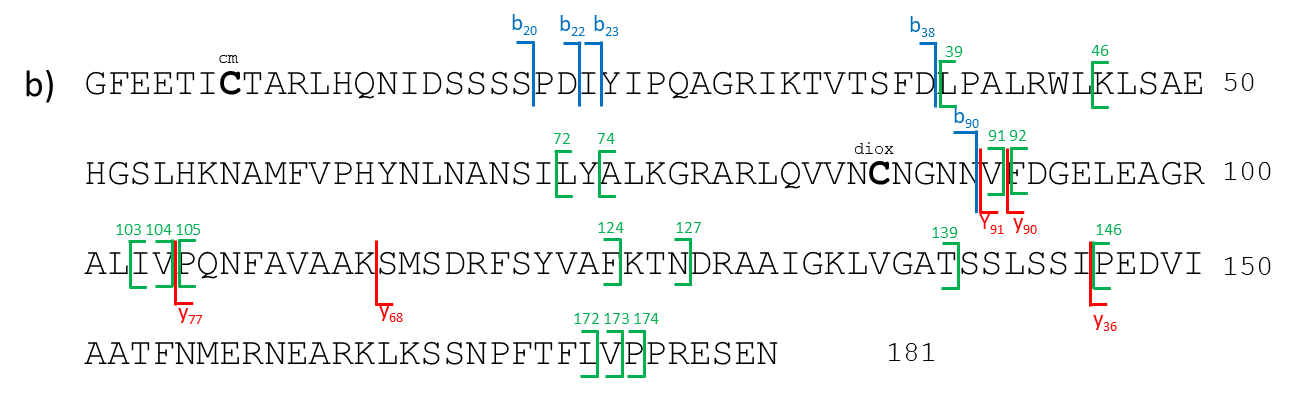


**Figure S4.** a) Deconvoluted MS/MS of the ion at m/z 869.5397, which correspond to the multi-charged ion [M+23H]^23+^ of the polypeptide with m_mono_ 19964.2195 Da. The most abundant y- and b-ions are marked in red and blue, respectively. The most intense internal fragment ions are shown in green. b) Sequence coverage map as obtained by the MS/MS reported in the panel a. This polypeptide corresponds to the region Gly334-Asn514 (here renumbered 1-181) of the legumin A-like entry with Acc. No. A0A1S2XSB9 carrying Cys7 (i.e., Cys340 in the entire legumin sequence) as carbamidomethyl-cysteine and Cys86 (i.e., Cys419 of the entire legumin sequence) as sulfinic acid. The cysteines are shown in bold and marked as cm (carbamidomethylated) and di-ox, respectively. b- and y-fragments are reported in blue and red, respectively. The internal fragments are reported in green.

Following the complete report of the ClipsMS interpretation (Fig.S5 and Table S3) of the multi-charged [M+23H]^23+^ at m/z 869.5397 of the polypeptide with m_mono_ 19964.2195 Da.


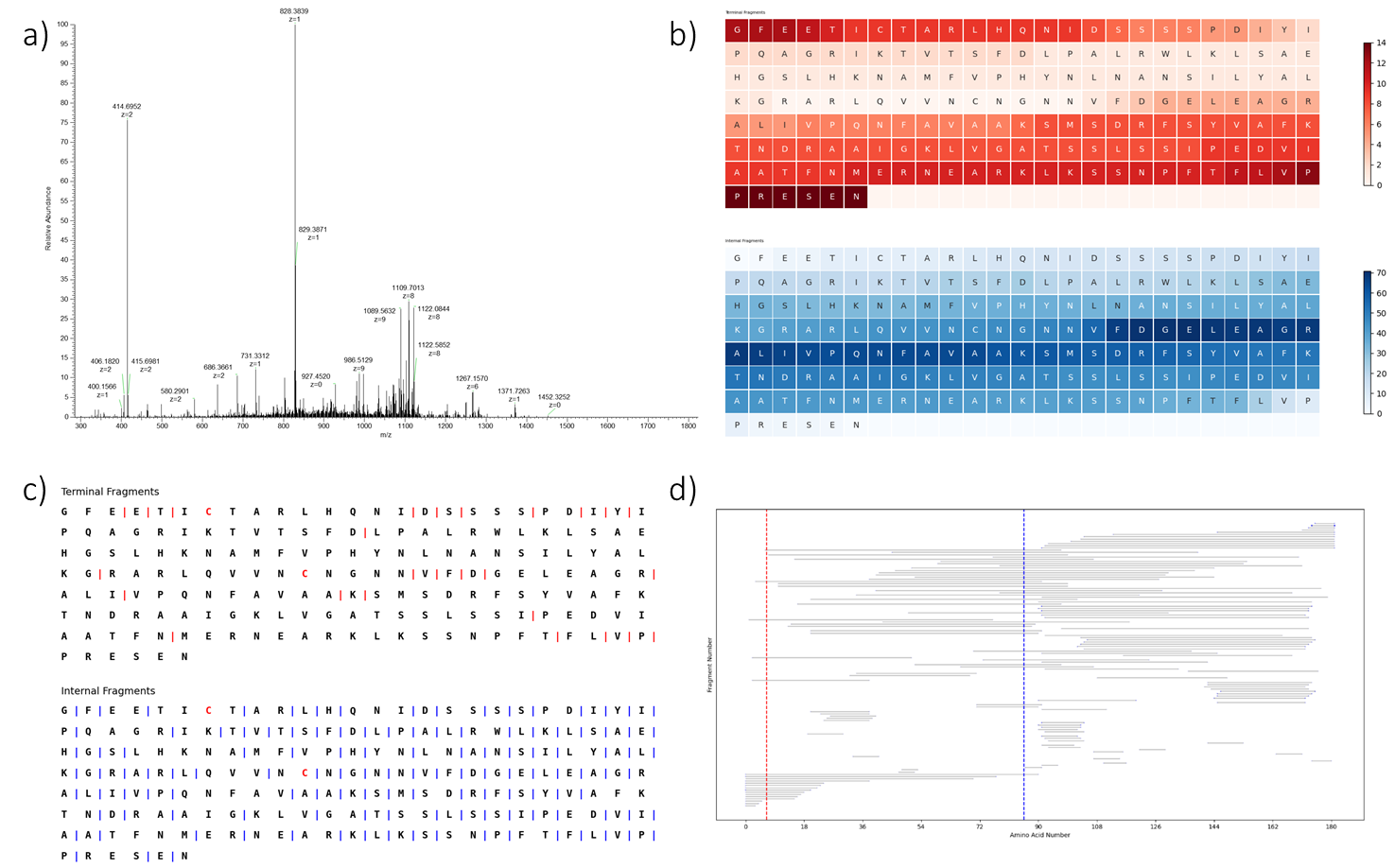


**Figure S5.** a) HCD mass spectrum of the ion at m/z 869.5397 ([M+23H]^23+^); b) sequence coverage map for the terminal and internal fragments. Darker regions indicate more coverage; c) fragment cleavage map indicating the location of inter-amino-acid cleavage sites for terminal and internal fragments); and d) fragment location map indicating the region of the β-chain sequence covered by terminal and internal fragments. The two lines, red and blue, indicate the cysteine residue positions.

**Table S3**. Sample output of ClipsMS related to the CID mass spectrum of the at m/z 869.5397 ([M+23H]^23+^). The Table includes the fragment type, observed mass, theoretical mass, start amino acid, end amino acid, error (ppm), sequence, intensity, and molecular formula. The amino acid positions refer to the sequence reported in Figure S4b.

| **Frag Type** | **Localized Mod** | **Observed Mass** | **Theoretical Mass** | **Start AA** | **End AA** | **Error** | **Sequence** | **Intensity** | **Formula** |
| --- | --- | --- | --- | --- | --- | --- | --- | --- | --- |
| B Fragment | 0.0000 | 334.1391 | 334.1397 | 1 | 3 | -2.0 | GFE | 3617.5200 | C16 H20 N3 O5 S0 |
| B Fragment | 0.0000 | 463.1816 | 463.1823 | 1 | 4 | -1.7 | GFEE | 3373.8700 | C21 H27 N4 O8 S0 |
| B Fragment | 0.0000 | 564.2293 | 564.2300 | 1 | 5 | -1.3 | GFEET | 2579.1600 | C25 H34 N5 O10 S0 |
| B Fragment | 57.0215 | 1770.8651 | 1770.8592 | 1 | 15 | 3.3 | GFEETICTARLHQNI | 2320.8500 | C74 H117 N22 O23 S1 |
| B Fragment | 57.0215 | 1885.8840 | 1885.8862 | 1 | 16 | -1.2 | GFEETICTARLHQNID | 890.1500 | C78 H122 N23 O26 S1 |
| B Fragment | 57.0215 | 1972.9167 | 1972.9182 | 1 | 17 | -0.8 | GFEETICTARLHQNIDS | 874.9200 | C81 H127 N24 O28 S1 |
| B Fragment | 57.0215 | 2059.9517 | 2059.9502 | 1 | 18 | 0.7 | GFEETICTARLHQNIDSS | 2850.0500 | C84 H132 N25 O30 S1 |
| B Fragment | 57.0215 | 2234.0111 | 2234.0143 | 1 | 20 | -1.4 | GFEETICTARLHQNIDSSSS | 16290.9900 | C90 H142 N27 O34 S1 |
| B Fragment | 57.0215 | 2446.0920 | 2446.0940 | 1 | 22 | -0.8 | GFEETICTARLHQNIDSSSSPD | 6136.3100 | C99 H154 N29 O38 S1 |
| B Fragment | 57.0215 | 2559.1723 | 2559.1781 | 1 | 23 | -2.3 | GFEETICTARLHQNIDSSSSPDI | 6872.2800 | C105 H165 N30 O39 S1 |
| B Fragment | 57.0215 | 2722.2389 | 2722.2414 | 1 | 24 | -0.9 | GFEETICTARLHQNIDSSSSPDIY | 4468.6300 | C114 H174 N31 O41 S1 |
| B Fragment | 57.0215 | 4236.0573 | 4236.0667 | 1 | 38 | -2.2 | GFEETICTARLHQNIDSSSSPDIYIPQAGRIKTVTSFD | 5487.9500 | C182 H285 N50 O61 S1 |
| B Fragment | 57.0215 | 7845.9208 | 7845.9446 | 1 | 70 | -3.0 | GFEETICTARLHQNIDSSSSPDIYIPQAGRIKTVTSFDLPALRWLKLSAEHGSLHKNAMFVPHYNLNANS | 682.9000 | C347 H537 N98 O103 S2 |
| B Fragment | 57.0215 | 8604.4207 | 8604.4137 | 1 | 77 | 0.8 | GFEETICTARLHQNIDSSSSPDIYIPQAGRIKTVTSFDLPALRWLKLSAEHGSLHKNAMFVPHYNLNANSILYALKG | 6118.3400 | C385 H599 N106 O111 S2 |
| B Fragment | 89.0113 | 10075.1049 | 10075.1246 | 1 | 90 | -2.0 | GFEETICTARLHQNIDSSSSPDIYIPQAGRIKTVTSFDLPALRWLKLSAEHGSLHKNAMFVPHYNLNANSILYALKGRARLQVVNCNGNN | 7655.7100 | C442 H697 N130 O129 S3 |
| Y Fragment | 0.0000 | 731.3313 | 731.3319 | 176 | 181 | -0.8 | PRESEN | 24956.1500 | C28 H47 N10 O13 S0 |
| Y Fragment | 0.0000 | 828.3837 | 828.3846 | 175 | 181 | -1.1 | PPRESEN | 310789.2800 | C33 H54 N11 O14 S0 |
| Y Fragment | 0.0000 | 927.4519 | 927.4530 | 174 | 181 | -1.3 | VPPRESEN | 22382.4600 | C38 H63 N12 O15 S0 |
| Y Fragment | 0.0000 | 1187.6051 | 1187.6055 | 172 | 181 | -0.4 | FLVPPRESEN | 1510.7300 | C53 H83 N14 O17 S0 |
| Y Fragment | 0.0000 | 4134.0676 | 4134.0713 | 146 | 181 | -0.9 | PEDVIAATFNMERNEARKLKSSNPFTFLVPPRESEN | 15128.3800 | C181 H286 N51 O58 S1 |
| Y Fragment | 0.0000 | 7494.8292 | 7494.7850 | 114 | 181 | 5.9 | SMSDRFSYVAFKTNDRAAIGKLVGATSSLSSIPEDVIAATFNMERNEARKLKSSNPFTFLVPPRESEN | 14975.2300 | C328 H523 N92 O105 S2 |
| Y Fragment | 0.0000 | 8421.2707 | 8421.2824 | 105 | 181 | -1.4 | PQNFAVAAKSMSDRFSYVAFKTNDRAAIGKLVGATSSLSSIPEDVIAATFNMERNEARKLKSSNPFTFLVPPRESEN | 10439.7400 | C371 H589 N104 O116 S2 |
| Y Fragment | 0.0000 | 8520.3332 | 8520.3508 | 104 | 181 | -2.1 | VPQNFAVAAKSMSDRFSYVAFKTNDRAAIGKLVGATSSLSSIPEDVIAATFNMERNEARKLKSSNPFTFLVPPRESEN | 936.1500 | C376 H598 N105 O117 S2 |
| Y Fragment | 0.0000 | 8817.5506 | 8817.5560 | 101 | 181 | -0.6 | ALIVPQNFAVAAKSMSDRFSYVAFKTNDRAAIGKLVGATSSLSSIPEDVIAATFNMERNEARKLKSSNPFTFLVPPRESEN | 7279.8900 | C391 H625 N108 O120 S2 |
| Y Fragment | 0.0000 | 9529.8698 | 9529.9064 | 94 | 181 | -3.8 | GELEAGRALIVPQNFAVAAKSMSDRFSYVAFKTNDRAAIGKLVGATSSLSSIPEDVIAATFNMERNEARKLKSSNPFTFLVPPRESEN | 2132.2000 | C420 H673 N118 O131 S2 |
| Y Fragment | 0.0000 | 9644.9198 | 9644.9334 | 93 | 181 | -1.4 | DGELEAGRALIVPQNFAVAAKSMSDRFSYVAFKTNDRAAIGKLVGATSSLSSIPEDVIAATFNMERNEARKLKSSNPFTFLVPPRESEN | 5736.5700 | C424 H678 N119 O134 S2 |
| Y Fragment | 0.0000 | 9791.9766 | 9792.0018 | 92 | 181 | -2.6 | FDGELEAGRALIVPQNFAVAAKSMSDRFSYVAFKTNDRAAIGKLVGATSSLSSIPEDVIAATFNMERNEARKLKSSNPFTFLVPPRESEN | 31964.3400 | C433 H687 N120 O135 S2 |
| BY Int Fragment | 0.0000 | 482.1987 | 482.1994 | 48 | 52 | -1.5 | SAEHG | 972.6500 | C19 H28 N7 O8 S0 |
| BY Int Fragment | 0.0000 | 482.1987 | 482.1994 | 49 | 53 | -1.5 | AEHGS | 972.6500 | C19 H28 N7 O8 S0 |
| BY Int Fragment | 0.0000 | 499.2253 | 499.2259 | 87 | 91 | -1.3 | NGNNV | 5554.0100 | C19 H31 N8 O8 S0 |
| BY Int Fragment | 0.0000 | 562.2498 | 562.2508 | 92 | 96 | -1.7 | FDGEL | 1193.6200 | C26 H36 N5 O9 S0 |
| BY Int Fragment | 0.0000 | 691.3064 | 691.3080 | 111 | 117 | -2.3 | AAKSMSD | 2709.6200 | C27 H47 N8 O11 S1 |
| BY Int Fragment | 0.0000 | 696.3300 | 696.3311 | 175 | 180 | -1.7 | PPRESE | 7076.9200 | C29 H46 N9 O11 S0 |
| BY Int Fragment | 0.0000 | 806.4171 | 806.4229 | 108 | 115 | -7.2 | FAVAAKSM | 1522.0200 | C37 H60 N9 O9 S1 |
| BY Int Fragment | 0.0000 | 831.4273 | 831.4247 | 34 | 41 | 3.1 | VTSFDLPA | 2442.1700 | C39 H59 N8 O12 S0 |
| BY Int Fragment | 0.0000 | 875.4613 | 875.4621 | 164 | 171 | -1.0 | LKSSNPFT | 3902.9600 | C40 H63 N10 O12 S0 |
| BY Int Fragment | 0.0000 | 893.4637 | 893.4550 | 108 | 116 | 9.8 | FAVAAKSMS | 3862.2100 | C40 H65 N10 O11 S1 |
| BY Int Fragment | 0.0000 | 932.4913 | 932.4948 | 122 | 129 | -3.8 | VAFKTNDR | 1149.8100 | C41 H66 N13 O12 S0 |
| BY Int Fragment | 0.0000 | 1010.5619 | 1010.5629 | 94 | 103 | -1.0 | GELEAGRALI | 655.8100 | C44 H76 N13 O14 S0 |
| BY Int Fragment | 0.0000 | 1046.4886 | 1046.4902 | 92 | 101 | -1.5 | FDGELEAGRA | 1131.6900 | C45 H68 N13 O16 S0 |
| BY Int Fragment | 0.0000 | 1084.5602 | 1084.5521 | 143 | 153 | 7.5 | SSIPEDVIAAT | 1734.7200 | C47 H78 N11 O18 S0 |
| BY Int Fragment | 0.0000 | 1109.6308 | 1109.6313 | 94 | 104 | -0.5 | GELEAGRALIV | 1303.4800 | C49 H85 N14 O15 S0 |
| BY Int Fragment | 0.0000 | 1125.5902 | 1125.5899 | 93 | 103 | 0.3 | DGELEAGRALI | 6219.7600 | C48 H81 N14 O17 S0 |
| BY Int Fragment | 0.0000 | 1159.5726 | 1159.5742 | 92 | 102 | -1.4 | FDGELEAGRAL | 10535.0900 | C51 H79 N14 O17 S0 |
| BY Int Fragment | 0.0000 | 1198.6164 | 1198.6215 | 20 | 30 | -4.3 | SPDIYIPQAGR | 2170.5700 | C54 H84 N15 O16 S0 |
| BY Int Fragment | 0.0000 | 1224.6556 | 1224.6583 | 93 | 104 | -2.2 | DGELEAGRALIV | 2975.5300 | C53 H90 N15 O18 S0 |
| BY Int Fragment | 0.0000 | 1258.6412 | 1258.6426 | 91 | 102 | -1.2 | VFDGELEAGRAL | 2695.3400 | C56 H88 N15 O18 S0 |
| BY Int Fragment | 0.0000 | 1272.6569 | 1272.6583 | 92 | 103 | -1.1 | FDGELEAGRALI | 16066.9100 | C57 H90 N15 O18 S0 |
| BY Int Fragment | 0.0000 | 1371.7255 | 1371.7267 | 91 | 103 | -0.9 | VFDGELEAGRALI | 27671.6500 | C62 H99 N16 O19 S0 |
| BY Int Fragment | 0.0000 | 1371.7255 | 1371.7267 | 92 | 104 | -0.9 | FDGELEAGRALIV | 27671.6500 | C62 H99 N16 O19 S0 |
| BY Int Fragment | 0.0000 | 1514.8295 | 1514.8326 | 25 | 38 | -2.0 | IPQAGRIKTVTSFD | 1243.1500 | C68 H112 N19 O20 S0 |
| BY Int Fragment | 0.0000 | 1514.8295 | 1514.8326 | 26 | 39 | -2.0 | PQAGRIKTVTSFDL | 1243.1500 | C68 H112 N19 O20 S0 |
| BY Int Fragment | 0.0000 | 1514.8295 | 1514.8326 | 27 | 40 | -2.0 | QAGRIKTVTSFDLP | 1243.1500 | C68 H112 N19 O20 S0 |
| BY Int Fragment | 0.0000 | 1677.8922 | 1677.8959 | 24 | 38 | -2.2 | YIPQAGRIKTVTSFD | 2861.3200 | C77 H121 N20 O22 S0 |
| BY Int Fragment | 0.0000 | 2003.0557 | 2003.0597 | 21 | 38 | -2.0 | PDIYIPQAGRIKTVTSFD | 5509.5400 | C92 H144 N23 O27 S0 |
| BY Int Fragment | 0.0000 | 2099.0911 | 2099.0920 | 92 | 111 | -0.4 | FDGELEAGRALIVPQNFAVA | 692.1400 | C96 H148 N25 O28 S0 |
| BY Int Fragment | 31.9898 | 2117.0962 | 2117.1032 | 72 | 90 | -3.3 | LYALKGRARLQVVNCNGNN | 1374.7900 | C89 H150 N31 O25 S1 |
| BY Int Fragment | 31.9898 | 2216.1702 | 2216.1717 | 72 | 91 | -0.7 | LYALKGRARLQVVNCNGNNV | 2498.1000 | C94 H159 N32 O26 S1 |
| BY Int Fragment | 0.0000 | 3094.5414 | 3094.5415 | 146 | 172 | 0.0 | PEDVIAATFNMERNEARKLKSSNPFTF | 7419.6700 | C137 H213 N38 O42 S1 |
| BY Int Fragment | 0.0000 | 3207.6203 | 3207.6256 | 91 | 120 | -1.6 | VFDGELEAGRALIVPQNFAVAAKSMSDRFS | 28349.5300 | C143 H224 N39 O43 S1 |
| BY Int Fragment | 0.0000 | 3207.6203 | 3207.6256 | 145 | 172 | -1.6 | IPEDVIAATFNMERNEARKLKSSNPFTF | 28349.5300 | C143 H224 N39 O43 S1 |
| BY Int Fragment | 0.0000 | 3207.6203 | 3207.6256 | 146 | 173 | -1.6 | PEDVIAATFNMERNEARKLKSSNPFTFL | 28349.5300 | C143 H224 N39 O43 S1 |
| BY Int Fragment | 0.0000 | 3306.6884 | 3306.6940 | 146 | 174 | -1.7 | PEDVIAATFNMERNEARKLKSSNPFTFLV | 34973.1900 | C148 H233 N40 O44 S1 |
| BY Int Fragment | 0.0000 | 3306.6884 | 3306.6940 | 147 | 175 | -1.7 | EDVIAATFNMERNEARKLKSSNPFTFLVP | 34973.1900 | C148 H233 N40 O44 S1 |
| BY Int Fragment | 0.0000 | 3407.7444 | 3407.7416 | 144 | 173 | 0.8 | SIPEDVIAATFNMERNEARKLKSSNPFTFL | 1100.6100 | C152 H240 N41 O46 S1 |
| BY Int Fragment | 0.0000 | 3494.7671 | 3494.7737 | 142 | 172 | -1.9 | LSSIPEDVIAATFNMERNEARKLKSSNPFTF | 2341.3000 | C155 H245 N42 O48 S1 |
| BY Int Fragment | 0.0000 | 3494.7671 | 3494.7737 | 143 | 173 | -1.9 | SSIPEDVIAATFNMERNEARKLKSSNPFTFL | 2341.3000 | C155 H245 N42 O48 S1 |
| BY Int Fragment | 0.0000 | 3593.8345 | 3593.8421 | 143 | 174 | -2.1 | SSIPEDVIAATFNMERNEARKLKSSNPFTFLV | 2743.6000 | C160 H254 N43 O49 S1 |
| BY Int Fragment | 57.0215 | 4031.9689 | 4031.9768 | 3 | 38 | -2.0 | EETICTARLHQNIDSSSSPDIYIPQAGRIKTVTSFD | 2497.3200 | C171 H273 N48 O59 S1 |
| BY Int Fragment | 0.0000 | 4143.1281 | 4143.1179 | 109 | 148 | 2.4 | AVAAKSMSDRFSYVAFKTNDRAAIGKLVGATSSLSSIPED | 945.7300 | C181 H293 N50 O59 S1 |
| BY Int Fragment | 0.0000 | 4174.1256 | 4174.1444 | 33 | 69 | -4.5 | TVTSFDLPALRWLKLSAEHGSLHKNAMFVPHYNLNAN | 1000.3600 | C191 H290 N53 O51 S1 |
| BY Int Fragment | 0.0000 | 4174.1256 | 4174.1444 | 35 | 71 | -4.5 | TSFDLPALRWLKLSAEHGSLHKNAMFVPHYNLNANSI | 1000.3600 | C191 H290 N53 O51 S1 |
| BY Int Fragment | 0.0000 | 4304.2128 | 4304.2020 | 137 | 176 | 2.5 | GATSSLSSIPEDVIAATFNMERNEARKLKSSNPFTFLVPP | 1496.4400 | C191 H304 N51 O60 S1 |
| BY Int Fragment | 0.0000 | 4368.2467 | 4368.2194 | 93 | 133 | 6.2 | DGELEAGRALIVPQNFAVAAKSMSDRFSYVAFKTNDRAAIG | 1467.2700 | C193 H304 N55 O59 S1 |
| BY Int Fragment | 31.9898 | 4425.2690 | 4425.2844 | 67 | 107 | -3.5 | NANSILYALKGRARLQVVNCNGNNVFDGELEAGRALIVPQN | 1890.4500 | C190 H311 N60 O58 S1 |
| BY Int Fragment | 31.9898 | 5057.5708 | 5057.5261 | 53 | 97 | 8.8 | SLHKNAMFVPHYNLNANSILYALKGRARLQVVNCNGNNVFDGELE | 710.0100 | C222 H347 N66 O64 S2 |
| BY Int Fragment | 0.0000 | 5371.7844 | 5371.7896 | 92 | 142 | -1.0 | FDGELEAGRALIVPQNFAVAAKSMSDRFSYVAFKTNDRAAIGKLVGATSSL | 1491.1400 | C240 H381 N66 O72 S1 |
| BY Int Fragment | 31.9898 | 5527.8426 | 5527.8114 | 74 | 124 | 5.6 | ALKGRARLQVVNCNGNNVFDGELEAGRALIVPQNFAVAAKSMSDRFSYVAF | 5996.3900 | C243 H385 N72 O70 S2 |
| BY Int Fragment | 57.0215 | 5546.8524 | 5546.8490 | 3 | 51 | 0.6 | EETICTARLHQNIDSSSSPDIYIPQAGRIKTVTSFDLPALRWLKLSAEH | 2114.8400 | C243 H387 N68 O75 S1 |
| BY Int Fragment | 0.0000 | 5657.9309 | 5657.9133 | 117 | 168 | 3.1 | DRFSYVAFKTNDRAAIGKLVGATSSLSSIPEDVIAATFNMERNEARKLKSSN | 706.6400 | C246 H399 N72 O79 S1 |
| BY Int Fragment | 0.0000 | 5658.9275 | 5658.9377 | 92 | 145 | -1.8 | FDGELEAGRALIVPQNFAVAAKSMSDRFSYVAFKTNDRAAIGKLVGATSSLSSI | 2859.2700 | C252 H402 N69 O77 S1 |
| BY Int Fragment | 31.9898 | 7240.7999 | 7240.8051 | 71 | 137 | -0.7 | ILYALKGRARLQVVNCNGNNVFDGELEAGRALIVPQNFAVAAKSMSDRFSYVAFKTNDRAAIGKLVG | 1230.8200 | C321 H517 N94 O91 S2 |
| BY Int Fragment | 0.0000 | 7381.7460 | 7381.7525 | 105 | 172 | -0.9 | PQNFAVAAKSMSDRFSYVAFKTNDRAAIGKLVGATSSLSSIPEDVIAATFNMERNEARKLKSSNPFTF | 4283.9300 | C327 H516 N91 O100 S2 |
| BY Int Fragment | 0.0000 | 7593.8987 | 7593.9050 | 103 | 172 | -0.8 | IVPQNFAVAAKSMSDRFSYVAFKTNDRAAIGKLVGATSSLSSIPEDVIAATFNMERNEARKLKSSNPFTF | 21037.1300 | C338 H536 N93 O102 S2 |
| BY Int Fragment | 0.0000 | 7593.8987 | 7593.9050 | 104 | 173 | -0.8 | VPQNFAVAAKSMSDRFSYVAFKTNDRAAIGKLVGATSSLSSIPEDVIAATFNMERNEARKLKSSNPFTFL | 21037.1300 | C338 H536 N93 O102 S2 |
| BY Int Fragment | 0.0000 | 7593.8987 | 7593.9050 | 105 | 174 | -0.8 | PQNFAVAAKSMSDRFSYVAFKTNDRAAIGKLVGATSSLSSIPEDVIAATFNMERNEARKLKSSNPFTFLV | 21037.1300 | C338 H536 N93 O102 S2 |
| BY Int Fragment | 0.0000 | 7593.8987 | 7593.9050 | 106 | 175 | -0.8 | QNFAVAAKSMSDRFSYVAFKTNDRAAIGKLVGATSSLSSIPEDVIAATFNMERNEARKLKSSNPFTFLVP | 21037.1300 | C338 H536 N93 O102 S2 |
| BY Int Fragment | 0.0000 | 7692.9623 | 7692.9734 | 104 | 174 | -1.4 | VPQNFAVAAKSMSDRFSYVAFKTNDRAAIGKLVGATSSLSSIPEDVIAATFNMERNEARKLKSSNPFTFLV | 2337.0300 | C343 H545 N94 O103 S2 |
| BY Int Fragment | 0.0000 | 7825.1019 | 7825.0593 | 93 | 165 | 5.4 | DGELEAGRALIVPQNFAVAAKSMSDRFSYVAFKTNDRAAIGKLVGATSSLSSIPEDVIAATFNMERNEARKLK | 672.0700 | C343 H557 N98 O107 S2 |
| BY Int Fragment | 31.9898 | 7842.1177 | 7842.1176 | 21 | 90 | 0.0 | PDIYIPQAGRIKTVTSFDLPALRWLKLSAEHGSLHKNAMFVPHYNLNANSILYALKGRARLQVVNCNGNN | 3256.5000 | C352 H556 N103 O95 S2 |
| BY Int Fragment | 31.9898 | 7941.1862 | 7941.1861 | 21 | 91 | 0.0 | PDIYIPQAGRIKTVTSFDLPALRWLKLSAEHGSLHKNAMFVPHYNLNANSILYALKGRARLQVVNCNGNNV | 2026.0700 | C357 H565 N104 O96 S2 |
| BY Int Fragment | 0.0000 | 8175.2145 | 8175.2699 | 98 | 173 | -6.8 | AGRALIVPQNFAVAAKSMSDRFSYVAFKTNDRAAIGKLVGATSSLSSIPEDVIAATFNMERNEARKLKSSNPFTFL | 871.0200 | C364 H583 N102 O108 S2 |
| BY Int Fragment | 31.9898 | 8304.3133 | 8304.3138 | 14 | 88 | -0.1 | NIDSSSSPDIYIPQAGRIKTVTSFDLPALRWLKLSAEHGSLHKNAMFVPHYNLNANSILYALKGRARLQVVNCNG | 1599.0100 | C370 H586 N107 O105 S2 |
| BY Int Fragment | 31.9898 | 8304.3133 | 8304.3138 | 15 | 89 | -0.1 | IDSSSSPDIYIPQAGRIKTVTSFDLPALRWLKLSAEHGSLHKNAMFVPHYNLNANSILYALKGRARLQVVNCNGN | 1599.0100 | C370 H586 N107 O105 S2 |
| BY Int Fragment | 31.9898 | 8418.2846 | 8418.3251 | 43 | 118 | -4.8 | RWLKLSAEHGSLHKNAMFVPHYNLNANSILYALKGRARLQVVNCNGNNVFDGELEAGRALIVPQNFAVAAKSMSDR | 339.1700 | C371 H588 N113 O104 S3 |
| BY Int Fragment | 57.0215 | 8490.3192 | 8490.3707 | 2 | 76 | -6.1 | FEETICTARLHQNIDSSSSPDIYIPQAGRIKTVTSFDLPALRWLKLSAEHGSLHKNAMFVPHYNLNANSILYALK | 1062.3600 | C381 H593 N104 O109 S2 |
| BY Int Fragment | 0.0000 | 8718.4589 | 8718.4876 | 93 | 173 | -3.3 | DGELEAGRALIVPQNFAVAAKSMSDRFSYVAFKTNDRAAIGKLVGATSSLSSIPEDVIAATFNMERNEARKLKSSNPFTFL | 6497.5100 | C386 H616 N107 O119 S2 |
| BY Int Fragment | 0.0000 | 8752.4759 | 8752.4719 | 92 | 172 | 0.4 | FDGELEAGRALIVPQNFAVAAKSMSDRFSYVAFKTNDRAAIGKLVGATSSLSSIPEDVIAATFNMERNEARKLKSSNPFTF | 13120.8700 | C389 H614 N107 O119 S2 |
| BY Int Fragment | 31.9898 | 8834.5320 | 8834.4583 | 51 | 130 | 8.3 | HGSLHKNAMFVPHYNLNANSILYALKGRARLQVVNCNGNNVFDGELEAGRALIVPQNFAVAAKSMSDRFSYVAFKTNDRA | 661.8700 | C390 H608 N117 O111 S3 |
| BY Int Fragment | 0.0000 | 8865.5372 | 8865.5560 | 92 | 173 | -2.1 | FDGELEAGRALIVPQNFAVAAKSMSDRFSYVAFKTNDRAAIGKLVGATSSLSSIPEDVIAATFNMERNEARKLKSSNPFTFL | 38494.4700 | C395 H625 N108 O120 S2 |
| BY Int Fragment | 0.0000 | 8964.5817 | 8964.6244 | 91 | 173 | -4.8 | VFDGELEAGRALIVPQNFAVAAKSMSDRFSYVAFKTNDRAAIGKLVGATSSLSSIPEDVIAATFNMERNEARKLKSSNPFTFL | 44186.2100 | C400 H634 N109 O121 S2 |
| BY Int Fragment | 0.0000 | 8964.5817 | 8964.6244 | 92 | 174 | -4.8 | FDGELEAGRALIVPQNFAVAAKSMSDRFSYVAFKTNDRAAIGKLVGATSSLSSIPEDVIAATFNMERNEARKLKSSNPFTFLV | 44186.2100 | C400 H634 N109 O121 S2 |
| BY Int Fragment | 31.9898 | 8979.6022 | 8979.6002 | 17 | 97 | 0.2 | SSSSPDIYIPQAGRIKTVTSFDLPALRWLKLSAEHGSLHKNAMFVPHYNLNANSILYALKGRARLQVVNCNGNNVFDGELE | 1678.6000 | C400 H627 N114 O116 S2 |
| BY Int Fragment | 0.0000 | 9063.6679 | 9063.6928 | 91 | 174 | -2.8 | VFDGELEAGRALIVPQNFAVAAKSMSDRFSYVAFKTNDRAAIGKLVGATSSLSSIPEDVIAATFNMERNEARKLKSSNPFTFLV | 763.8700 | C405 H643 N110 O122 S2 |
| BY Int Fragment | 31.9898 | 9099.6963 | 9099.7530 | 21 | 102 | -6.2 | PDIYIPQAGRIKTVTSFDLPALRWLKLSAEHGSLHKNAMFVPHYNLNANSILYALKGRARLQVVNCNGNNVFDGELEAGRAL | 763.1100 | C408 H643 N118 O113 S2 |
| BY Int Fragment | 0.0000 | 9530.8871 | 9530.9057 | 92 | 179 | -2.0 | FDGELEAGRALIVPQNFAVAAKSMSDRFSYVAFKTNDRAAIGKLVGATSSLSSIPEDVIAATFNMERNEARKLKSSNPFTFLVPPRES | 373.4500 | C424 H672 N117 O129 S2 |
| BY Int Fragment | 31.9898 | 9659.9307 | 9659.9219 | 35 | 121 | 0.9 | TSFDLPALRWLKLSAEHGSLHKNAMFVPHYNLNANSILYALKGRARLQVVNCNGNNVFDGELEAGRALIVPQNFAVAAKSMSDRFSY | 3299.3300 | C432 H671 N124 O121 S3 |
| BY Int Fragment | 31.9898 | 9673.9590 | 9673.9129 | 79 | 168 | 4.8 | ARLQVVNCNGNNVFDGELEAGRALIVPQNFAVAAKSMSDRFSYVAFKTNDRAAIGKLVGATSSLSSIPEDVIAATFNMERNEARKLKSSN | 701.3400 | C418 H677 N124 O132 S3 |
| BY Int Fragment | 31.9898 | 9774.9742 | 9774.9965 | 41 | 128 | -2.3 | ALRWLKLSAEHGSLHKNAMFVPHYNLNANSILYALKGRARLQVVNCNGNNVFDGELEAGRALIVPQNFAVAAKSMSDRFSYVAFKTND | 3238.2700 | C436 H680 N127 O122 S3 |
| BY Int Fragment | 0.0000 | 9813.0112 | 9813.0497 | 87 | 177 | -3.9 | NGNNVFDGELEAGRALIVPQNFAVAAKSMSDRFSYVAFKTNDRAAIGKLVGATSSLSSIPEDVIAATFNMERNEARKLKSSNPFTFLVPPR | 466.1900 | C435 H690 N123 O132 S2 |
| BY Int Fragment | 31.9898 | 9827.9888 | 9828.0143 | 11 | 99 | -2.6 | LHQNIDSSSSPDIYIPQAGRIKTVTSFDLPALRWLKLSAEHGSLHKNAMFVPHYNLNANSILYALKGRARLQVVNCNGNNVFDGELEAG | 346.9800 | C436 H683 N126 O128 S2 |
| BY Int Fragment | 31.9898 | 9828.0213 | 9828.0143 | 11 | 99 | 0.7 | LHQNIDSSSSPDIYIPQAGRIKTVTSFDLPALRWLKLSAEHGSLHKNAMFVPHYNLNANSILYALKGRARLQVVNCNGNNVFDGELEAG | 300.6000 | C436 H683 N126 O128 S2 |
| BY Int Fragment | 89.0113 | 9840.9808 | 9841.0606 | 4 | 91 | -8.1 | ETICTARLHQNIDSSSSPDIYIPQAGRIKTVTSFDLPALRWLKLSAEHGSLHKNAMFVPHYNLNANSILYALKGRARLQVVNCNGNNV | 1406.1600 | C431 H687 N128 O125 S3 |
| BY Int Fragment | 31.9898 | 9870.0262 | 9870.1064 | 39 | 127 | -8.1 | LPALRWLKLSAEHGSLHKNAMFVPHYNLNANSILYALKGRARLQVVNCNGNNVFDGELEAGRALIVPQNFAVAAKSMSDRFSYVAFKTN | 4201.6100 | C443 H693 N128 O121 S3 |
| BY Int Fragment | 31.9898 | 9912.0307 | 9912.0864 | 53 | 144 | -5.6 | SLHKNAMFVPHYNLNANSILYALKGRARLQVVNCNGNNVFDGELEAGRALIVPQNFAVAAKSMSDRFSYVAFKTNDRAAIGKLVGATSSLSS | 698.1200 | C437 H695 N128 O128 S3 |
| BY Int Fragment | 31.9898 | 9931.0390 | 9931.0976 | 41 | 129 | -5.9 | ALRWLKLSAEHGSLHKNAMFVPHYNLNANSILYALKGRARLQVVNCNGNNVFDGELEAGRALIVPQNFAVAAKSMSDRFSYVAFKTNDR | 728.8700 | C442 H692 N131 O123 S3 |
| BY Int Fragment | 31.9898 | 9931.0390 | 9931.0976 | 42 | 130 | -5.9 | LRWLKLSAEHGSLHKNAMFVPHYNLNANSILYALKGRARLQVVNCNGNNVFDGELEAGRALIVPQNFAVAAKSMSDRFSYVAFKTNDRA | 728.8700 | C442 H692 N131 O123 S3 |
| BY Int Fragment | 31.9898 | 9944.0789 | 9944.1027 | 47 | 138 | -2.4 | LSAEHGSLHKNAMFVPHYNLNANSILYALKGRARLQVVNCNGNNVFDGELEAGRALIVPQNFAVAAKSMSDRFSYVAFKTNDRAAIGKLVGA | 428.3700 | C440 H695 N130 O126 S3 |
| BY Int Fragment | 31.9898 | 9969.0741 | 9969.1079 | 52 | 144 | -3.4 | GSLHKNAMFVPHYNLNANSILYALKGRARLQVVNCNGNNVFDGELEAGRALIVPQNFAVAAKSMSDRFSYVAFKTNDRAAIGKLVGATSSLSS | 3164.8500 | C439 H698 N129 O129 S3 |
| BY Int Fragment | 31.9898 | 10019.0451 | 10019.0818 | 79 | 171 | -3.7 | ARLQVVNCNGNNVFDGELEAGRALIVPQNFAVAAKSMSDRFSYVAFKTNDRAAIGKLVGATSSLSSIPEDVIAATFNMERNEARKLKSSNPFT | 369.7300 | C436 H700 N127 O136 S3 |
| BY Int Fragment | 31.9898 | 10040.1073 | 10040.1767 | 60 | 153 | -6.9 | FVPHYNLNANSILYALKGRARLQVVNCNGNNVFDGELEAGRALIVPQNFAVAAKSMSDRFSYVAFKTNDRAAIGKLVGATSSLSSIPEDVIAAT | 2273.5500 | C445 H707 N126 O133 S2 |
| BY Int Fragment | 31.9898 | 10040.1073 | 10040.1767 | 61 | 154 | -6.9 | VPHYNLNANSILYALKGRARLQVVNCNGNNVFDGELEAGRALIVPQNFAVAAKSMSDRFSYVAFKTNDRAAIGKLVGATSSLSSIPEDVIAATF | 2273.5500 | C445 H707 N126 O133 S2 |
| BY Int Fragment | 31.9898 | 10114.1212 | 10114.2148 | 16 | 107 | -9.3 | DSSSSPDIYIPQAGRIKTVTSFDLPALRWLKLSAEHGSLHKNAMFVPHYNLNANSILYALKGRARLQVVNCNGNNVFDGELEAGRALIVPQN | 785.6500 | C449 H709 N130 O131 S2 |
| BY Int Fragment | 31.9898 | 10131.1292 | 10131.1567 | 77 | 170 | -2.7 | GRARLQVVNCNGNNVFDGELEAGRALIVPQNFAVAAKSMSDRFSYVAFKTNDRAAIGKLVGATSSLSSIPEDVIAATFNMERNEARKLKSSNPF | 2126.5000 | C440 H708 N131 O136 S3 |
| BY Int Fragment | 31.9898 | 10156.1653 | 10156.2002 | 8 | 99 | -3.4 | TARLHQNIDSSSSPDIYIPQAGRIKTVTSFDLPALRWLKLSAEHGSLHKNAMFVPHYNLNANSILYALKGRARLQVVNCNGNNVFDGELEAG | 711.1300 | C449 H707 N132 O132 S2 |
| BY Int Fragment | 31.9898 | 10173.1727 | 10173.2454 | 46 | 139 | -7.1 | KLSAEHGSLHKNAMFVPHYNLNANSILYALKGRARLQVVNCNGNNVFDGELEAGRALIVPQNFAVAAKSMSDRFSYVAFKTNDRAAIGKLVGAT | 6399.8800 | C450 H714 N133 O129 S3 |
| BY Int Fragment | 89.0113 | 10188.1703 | 10188.1723 | 7 | 97 | -0.2 | CTARLHQNIDSSSSPDIYIPQAGRIKTVTSFDLPALRWLKLSAEHGSLHKNAMFVPHYNLNANSILYALKGRARLQVVNCNGNNVFDGELE | 587.2600 | C447 H704 N131 O131 S3 |

Following the the MS/MS of the triply-charged molecular ion at m/z 727.6729 (Figure S6). The database search, carried out by PEAKS, identified this ion with the tryptic peptide Leu^414^-Arg^433^ of the legumin with Acc. No. A0A1S2XSB9, carrying the cysteine residue at position 419 as sulfinic acid (Cys-SO_2_H).


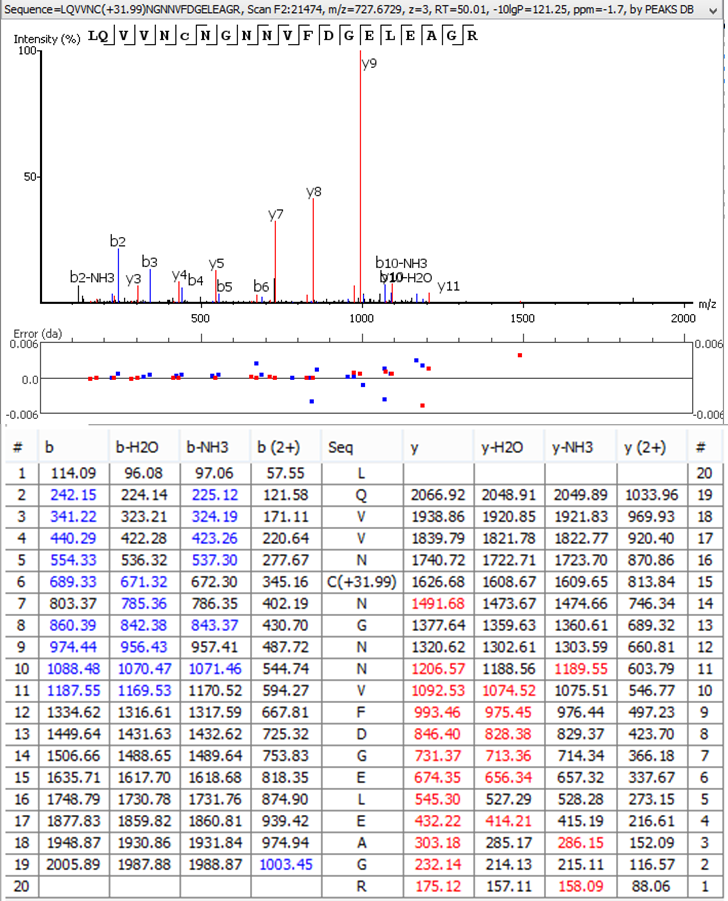


**Figure S6.** PEAKS report of the MS/MS of the ion at m/z 727.6729.

The component with m_mono_ 20021.22 Da shows a mass difference of +31.98 Da respect to the mass of the polypeptide at 19989.24 Da, above described. The observed mass shift could be due to the mono-oxidation (+15.99 Da) of two out of three methionine residues included in the Gly334-Asn514 region. Otherwise, it can be due to the di-oxidation of a methionine residue to methionine sulfone (+31.98 Da). But, taking into account the results of the shotgun approach, the hypothesis of the di-oxidation of a methionine residue should be excluded. Figure S7 shows the MS/MS of the ions at m/z 760.8503 (a) and 842.4227 (b), respectively. These ions were matched by the PEAKS software with the amino acid traits Asn^390^-Asn^402^ and Leu^468^-Arg^491^ of the legumin A-like entry with the Acc. No. A0A1S2XSB9, carrying the methionine residues at position 392 and 489 as methionine sulfoxide (+15.9949 Da), respectively. It should be highlighted that the region Asn390-Asn402 is common with the legumins with the Acc. No. A0A3Q7XNW1 and Q9SMJ4.


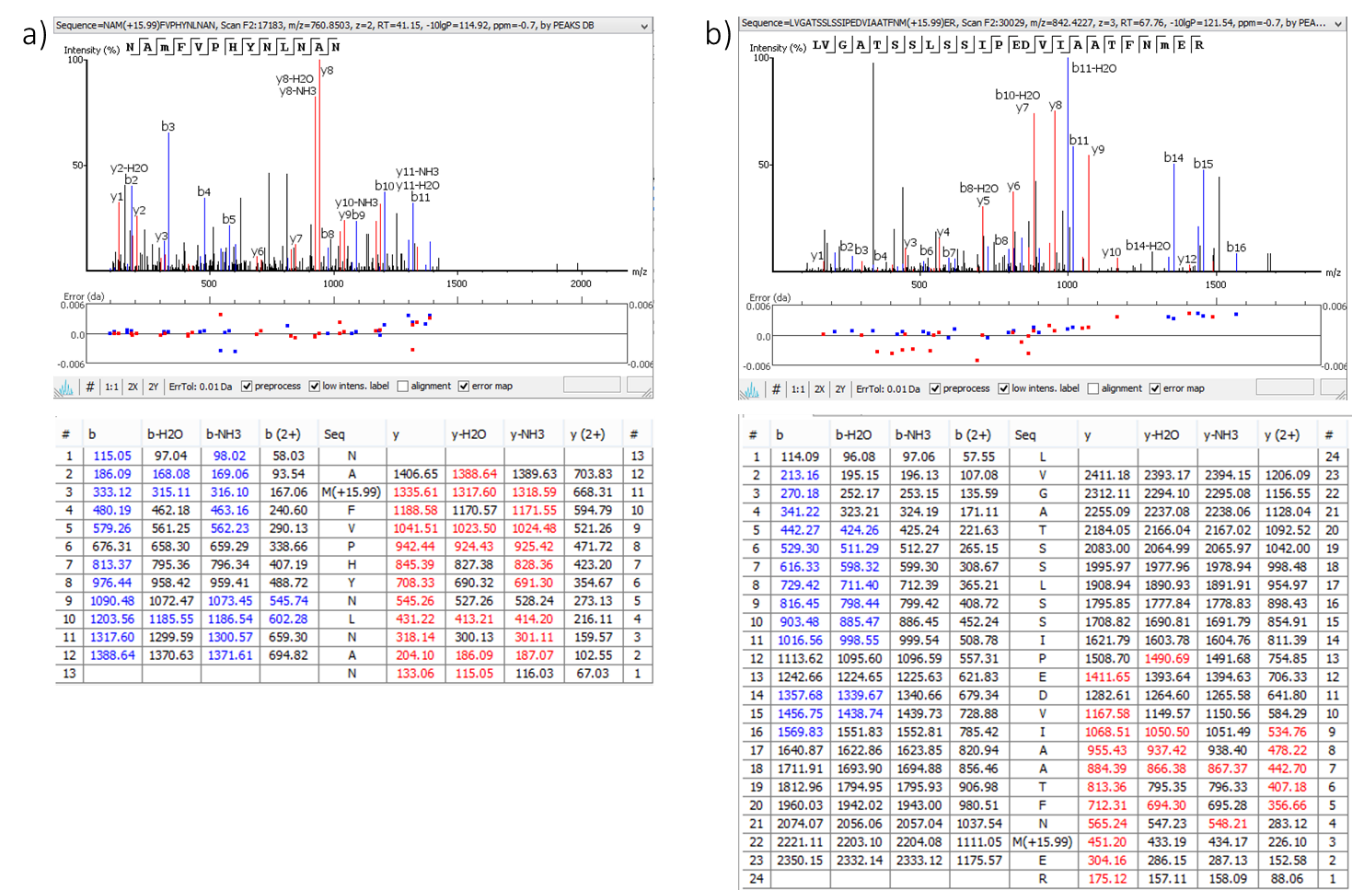


**Figure S7.** PEAKS report of the MS/MS of the ions at m/z 760.8503 (a) and 842.4227 (b).

Following the MS/MS of the doubly-charged molecular ion at m/z 1099.0031 (Figure S8) detected in the shotgun approach of the enriched fraction of legumins. The database search, carried out by PEAKS, identified this ion with the tryptic peptide Leu^414^-Arg^433^ of the legumin with Acc. No. A0A1S2XSB9, carrying the cysteine residue at position 419 as sulfonic acid, Cys-SO_3_H.


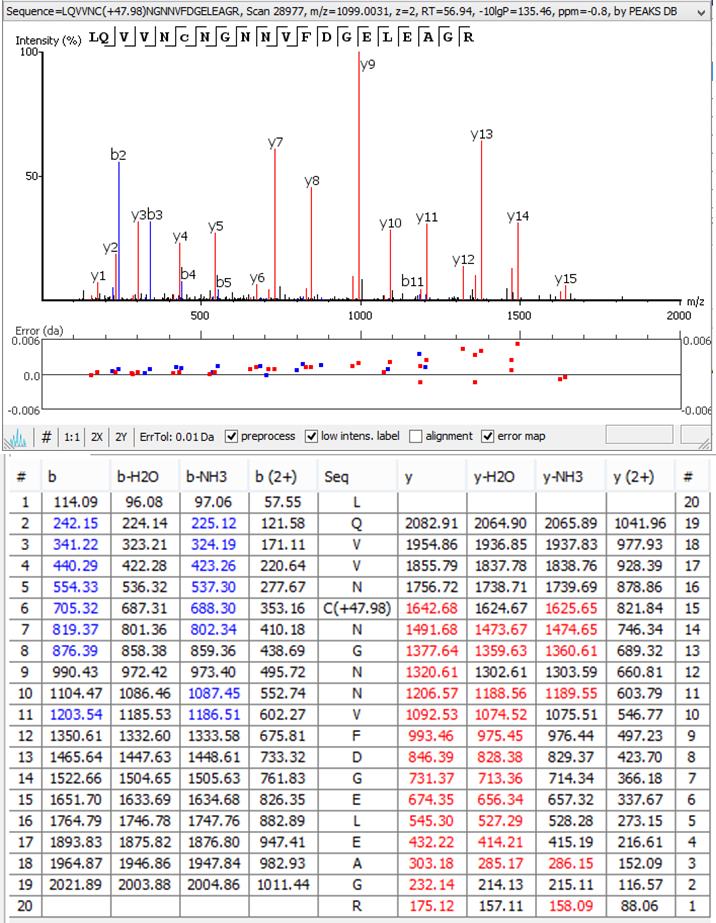


**Figure S8.** PEAKS report of the MS/MS of the ion at m/z 1099.0031.

***Polypeptides related to the legumin A0A1S2XVG1***

Figure S9 reports the multi-charged ESI mass spectrum of four co-eluting polypeptides showing the experimentally determined monoisotopic masses of 20060.4701, 20076.4647, 20131.4791, and 20147.4786 Da, which are related with the amino acid region Gly^353^-His^507^ or Gly^353^-Ala^508^ of the legumin J-like entry with the Acc. No. A0A1S2XVG1.


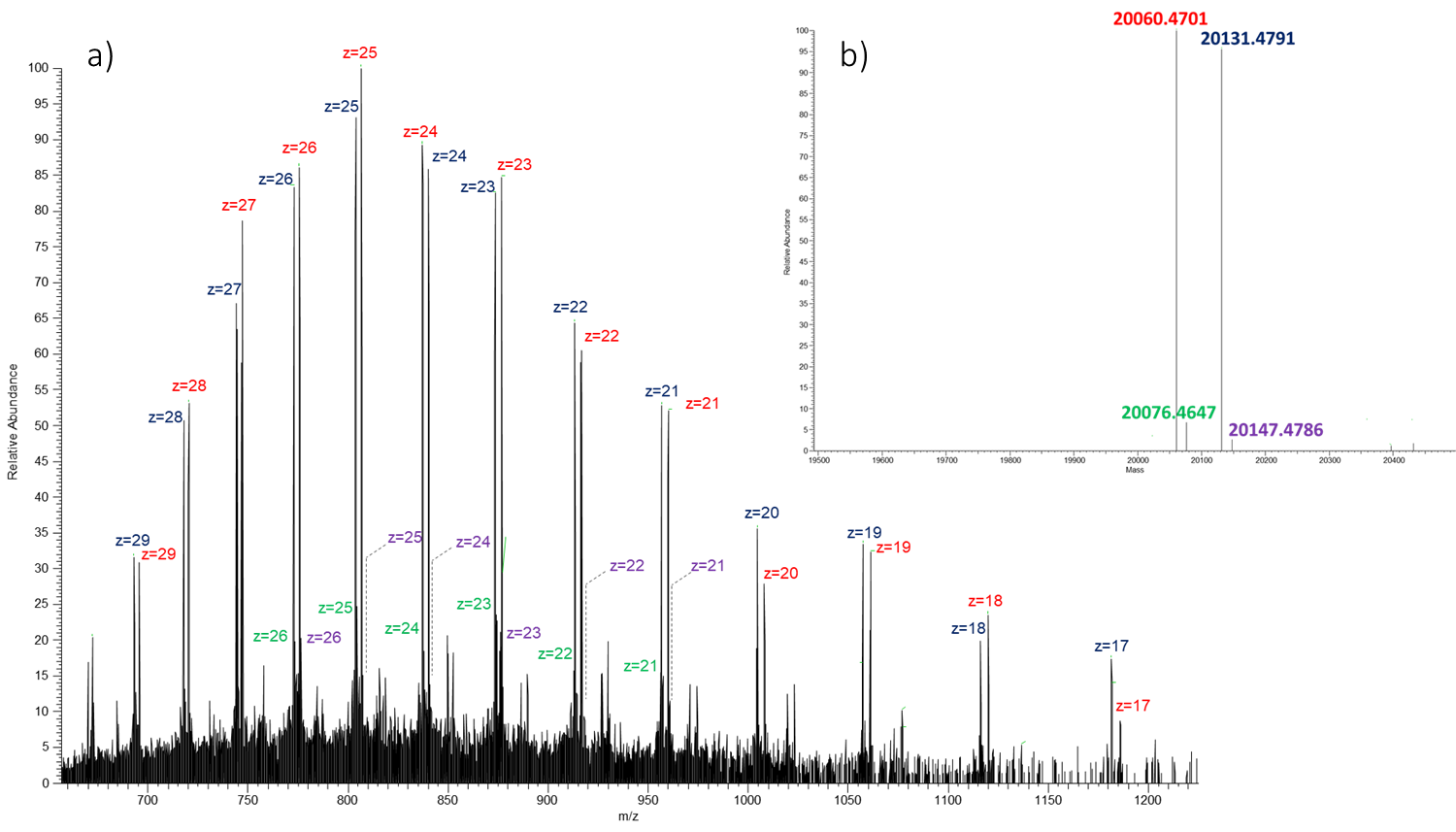


**Figure S9.** a) Multi-charged mass spectrum and b) monoisotopic deconvoluted mass spectrum (mass zero-charge) of the four coeluting polypeptides with m_mono_ 20060.4701, 20076.4647, 20131.4791 and 20147.4786 Da.

Following the MS/MS of the doubly-charged molecular ion at m/z 855.9087 and the triply-charged molecular ion at m/z 704.0283 detected in the shotgun approach of the enriched fraction of legumins. The database search, carried out by PEAKS, identified these ions with the tryptic peptides Ile^435^-Arg^449^ (Fig. S10a) and Val^433^-Lys^450^ (Fig.S10b), respectively, of the legumin with Acc. No. A0A1S2XVG1. Particularly, the first peptide carries the cysteine residue at position 438 as sulfinic acid (+31.99 Da), whereas the second one shows this cysteine as sulfonic acid (+47.98 Da).


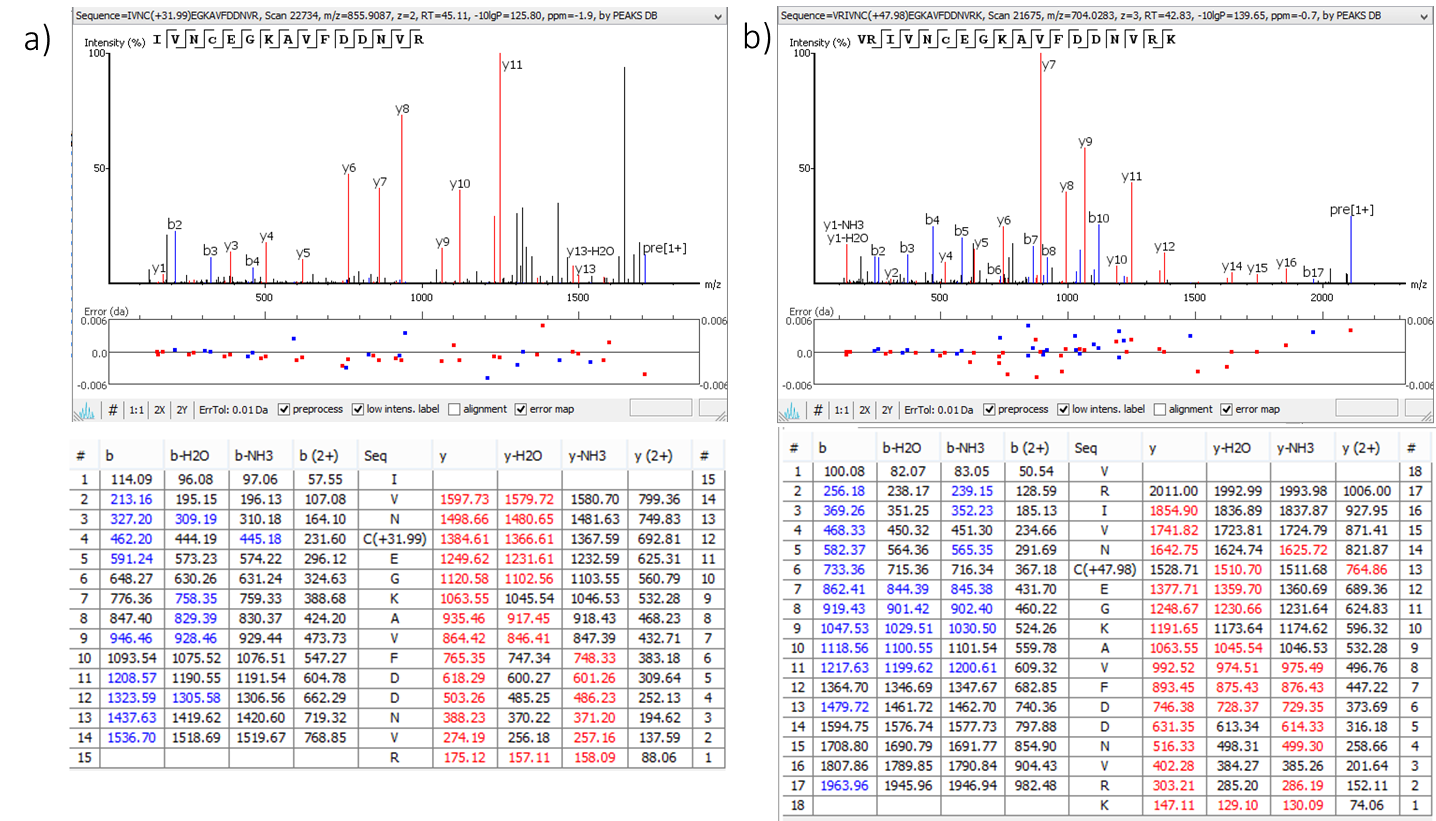


**Figure S10.** PEAKS report of the MS/MS of the ions at m/z 855.9087 (a) and 704.0283 (b).

Top-down characterization of the component with the experimental mass 20060.4701 Da was achieved by the MS/MS of its multi-charged ion [M+24H]^24+^ at m/z 837.3197, interpreted by the ClipsMS tool using the criteria above reported for internal fragments. This polypeptide was identified with the region Gly353-His531 of the legumin J-like entry with the Acc. No. A0A1S2XVG1 carrying the Cys359 in the carbamidomethylated form and the Cys438 as sulfinic acid. The deconvoluted MS/MS of this ion is reported in Figure S11a. The assignments of the MS signals refer to this amino acid region renumbered as Gly1-His179 and carrying the cysteine at position 7 (i.e., the Cys359 in the whole legumin sequence) as carbamidomethyl-cysteine and the cysteine 86 (i.e., the Cys438) as sulfinic acid (Figure S11b). In particular, the peaks at m/z 8817.5346 and 9742.9716 appear diagnostic for the presence of cysteine 86 as sulfinic acid. Indeed, they might be assigned to two putative internal fragments, the Leu37-Ala114 and Ser70-Asn157 (or Asn69-Gln156) regions, respectively, which include this cysteine as sulfinic acid.


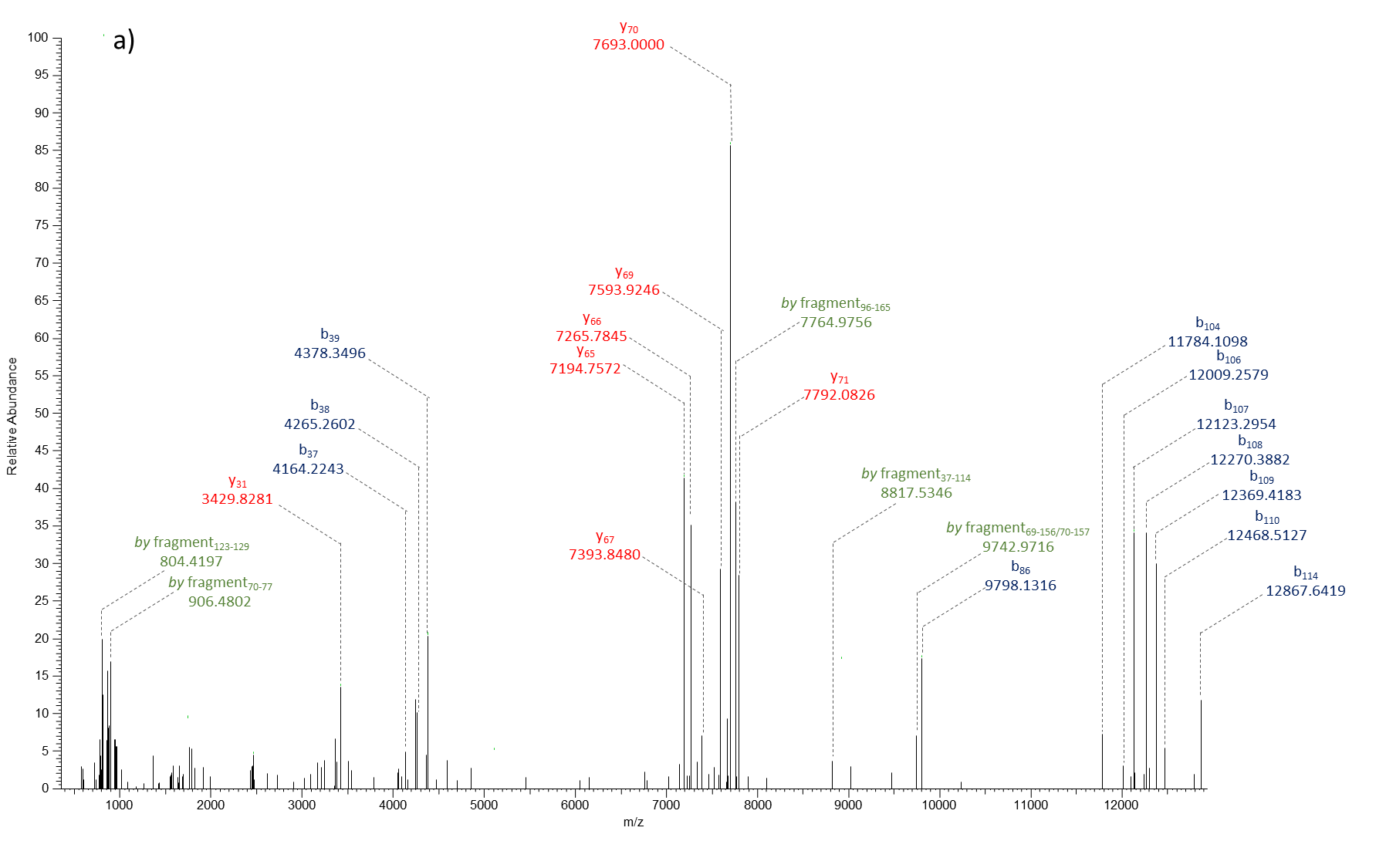


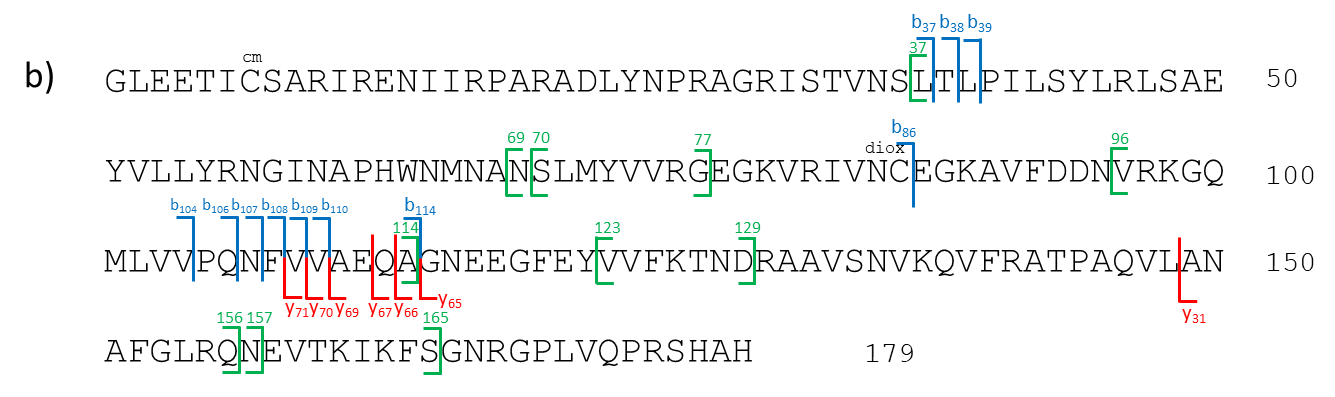


**Figure S11.** a) Deconvoluted MS/MS of the ion at m/z 837.3197, which correspond to the multi-charged ion [M+24H]^24+^ of the polypeptide with m_mono_ 20060.4701 Da. The most abundant y- and b-ions are marked in red and blue, respectively. The most intense internal fragment ions are shown in green. b) Sequence coverage map as obtained by the MS/MS reported in the panel a. This polypeptide corresponds to the region Gly353-His531 (here renumbered 1-179) of the legumin J-like entry with Acc. No. A0A1S2XVG1 carrying Cys7 (i.e Cys359 in the entire legumin sequence) as carbamidomethyl-cysteine and Cys86 (i.e. Cys438 of the entire legumin sequence) as sulfinic acid. The cysteines are shown in bold and marked as cm (carbamidomethylated) and di-ox, respectively. b- and y-fragments are reported in blue and red, respectively. The internal fragments are reported in green.

Following the complete report of the ClipsMS interpretation (Fig.S12 and Table S4) of the multi-charged ion [M+24H]^24+^ at m/z 837.3197 of the polypeptide with m_mono_ 20060.4701 Da, which corresponds to the region Gly^353^-His^531^ of the legumin J-like entry with the Acc. No. A0A1S2XVG1 carrying the Cys^359^ as carbamidomethyl-cysteine and the Cys^438^ as sulfinic acid.


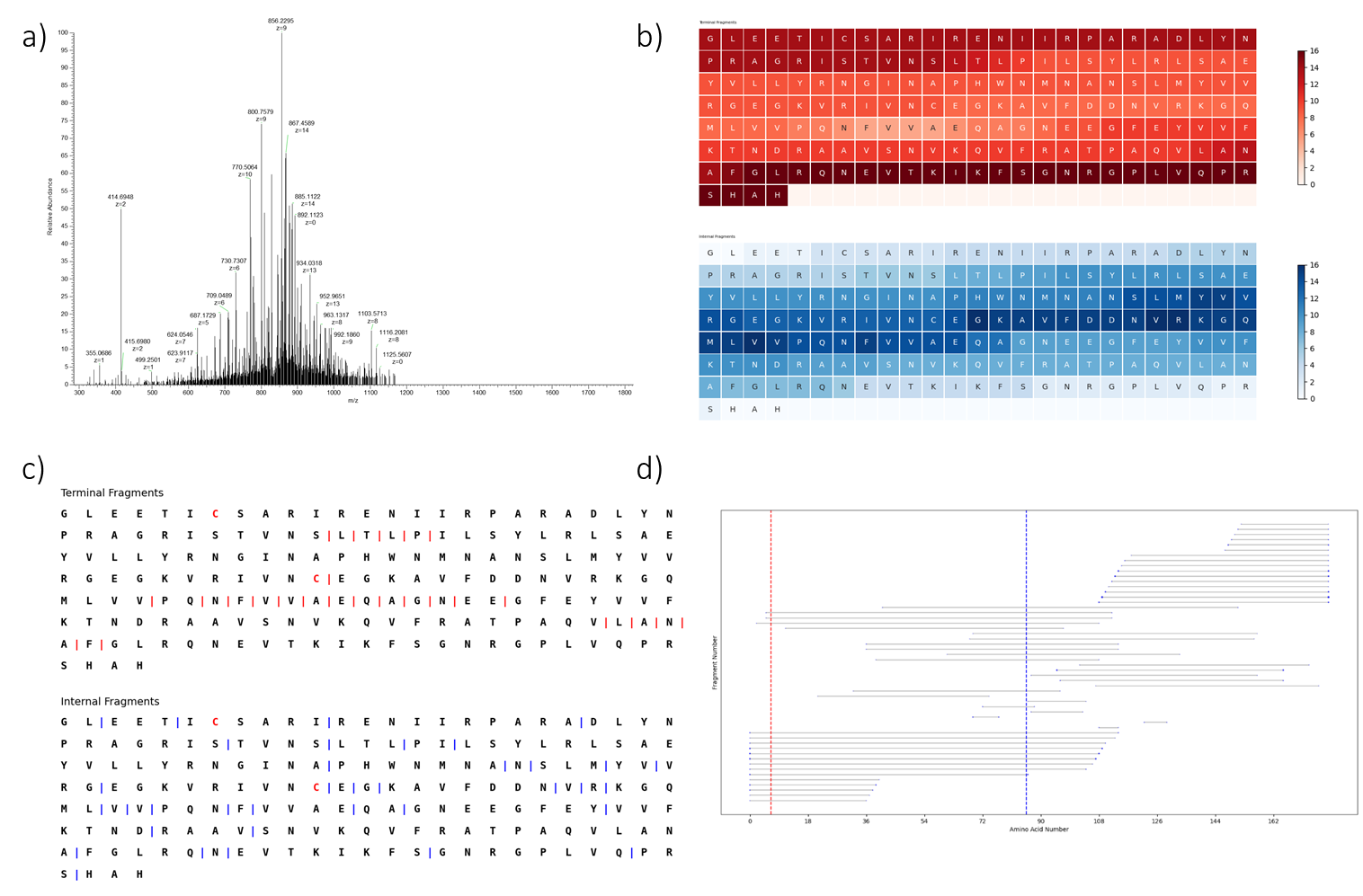


**Figure S12.** a) HCD mass spectrum of the ion at m/z 837.3197 ([M+24H]^24+^); b) sequence coverage map for the terminal and internal fragments. Darker regions indicate more coverage; c) fragment cleavage map indicating the location of inter-amino-acid cleavage sites for terminal and internal fragments); and d) fragment location map indicating the region of the β-chain sequence covered by terminal and internal fragments. The two lines, red and blue, indicate the cysteine residue positions.

**Table S4**. Sample output of ClipsMS related to the CID mass spectrum of the at m/z 837.3197 ([M+24H]^24+^). The Table includes the fragment type, observed mass, theoretical mass, start amino acid, end amino acid, error (ppm), sequence, intensity, and molecular formula. The amino acid positions refer to the sequence reported in Figure S11b.

| **Frag Type** | **Localized Mod** | **Observed Mass** | **Theoretical Mass** | **Start AA** | **End AA** | **Error** | **Sequence** | **Intensity** | **Formula** |
| --- | --- | --- | --- | --- | --- | --- | --- | --- | --- |
| B Fragment | 57.0215 | 4051.1203 | 4051.1367 | 1 | 36 | -4.1 | GLEETICSARIRENIIRPARADLYNPRAGRISTVNS | 1266.15 | C169 H286 N57 O53 S1 |
| B Fragment | 57.0215 | 4164.2243 | 4164.2207 | 1 | 37 | 0.8 | GLEETICSARIRENIIRPARADLYNPRAGRISTVNSL | 643.2 | C175 H297 N58 O54 S1 |
| B Fragment | 57.0215 | 4265.2602 | 4265.2684 | 1 | 38 | -1.9 | GLEETICSARIRENIIRPARADLYNPRAGRISTVNSLT | 4818.58 | C179 H304 N59 O56 S1 |
| B Fragment | 57.0215 | 4378.3496 | 4378.3525 | 1 | 39 | -0.7 | GLEETICSARIRENIIRPARADLYNPRAGRISTVNSLTL | 9585.79 | C185 H315 N60 O57 S1 |
| B Fragment | 57.0215 | 4475.4081 | 4475.4053 | 1 | 40 | 0.6 | GLEETICSARIRENIIRPARADLYNPRAGRISTVNSLTLP | 629.14 | C190 H322 N61 O58 S1 |
| B Fragment | 89.0113 | 9798.1316 | 9798.1421 | 1 | 86 | -1.1 | GLEETICSARIRENIIRPARADLYNPRAGRISTVNSLTLPILSYLRLSAEYVLLYRNGINAPHWNMNANSLMYVVRGEGKVRIVNC | 8151.49 | C428 H698 N129 O121 S4 |
| B Fragment | 89.0113 | 11784.1098 | 11784.1778 | 1 | 104 | -5.8 | GLEETICSARIRENIIRPARADLYNPRAGRISTVNSLTLPILSYLRLSAEYVLLYRNGINAPHWNMNANSLMYVVRGEGKVRIVNCEGKAVFDDNVRKGQMLVV | 3457.34 | C515 H841 N154 O147 S5 |
| B Fragment | 89.0113 | 12009.2579 | 12009.2892 | 1 | 106 | -2.6 | GLEETICSARIRENIIRPARADLYNPRAGRISTVNSLTLPILSYLRLSAEYVLLYRNGINAPHWNMNANSLMYVVRGEGKVRIVNCEGKAVFDDNVRKGQMLVVPQ | 1506.29 | C525 H856 N157 O150 S5 |
| B Fragment | 89.0113 | 12123.2954 | 12123.3321 | 1 | 107 | -3.0 | GLEETICSARIRENIIRPARADLYNPRAGRISTVNSLTLPILSYLRLSAEYVLLYRNGINAPHWNMNANSLMYVVRGEGKVRIVNCEGKAVFDDNVRKGQMLVVPQN | 16058.2 | C529 H862 N159 O152 S5 |
| B Fragment | 89.0113 | 12270.3882 | 12270.4005 | 1 | 108 | -1.0 | GLEETICSARIRENIIRPARADLYNPRAGRISTVNSLTLPILSYLRLSAEYVLLYRNGINAPHWNMNANSLMYVVRGEGKVRIVNCEGKAVFDDNVRKGQMLVVPQNF | 16056.08 | C538 H871 N160 O153 S5 |
| B Fragment | 89.0113 | 12369.4183 | 12369.4689 | 1 | 109 | -4.1 | GLEETICSARIRENIIRPARADLYNPRAGRISTVNSLTLPILSYLRLSAEYVLLYRNGINAPHWNMNANSLMYVVRGEGKVRIVNCEGKAVFDDNVRKGQMLVVPQNFV | 14134.43 | C543 H880 N161 O154 S5 |
| B Fragment | 89.0113 | 12468.5127 | 12468.5373 | 1 | 110 | -2.0 | GLEETICSARIRENIIRPARADLYNPRAGRISTVNSLTLPILSYLRLSAEYVLLYRNGINAPHWNMNANSLMYVVRGEGKVRIVNCEGKAVFDDNVRKGQMLVVPQNFVV | 2575.6 | C548 H889 N162 O155 S5 |
| B Fragment | 89.0113 | 12796.6401 | 12796.6756 | 1 | 113 | -2.8 | GLEETICSARIRENIIRPARADLYNPRAGRISTVNSLTLPILSYLRLSAEYVLLYRNGINAPHWNMNANSLMYVVRGEGKVRIVNCEGKAVFDDNVRKGQMLVVPQNFVVAEQ | 958.68 | C561 H909 N166 O161 S5 |
| B Fragment | 89.0113 | 12867.6419 | 12867.7127 | 1 | 114 | -5.5 | GLEETICSARIRENIIRPARADLYNPRAGRISTVNSLTLPILSYLRLSAEYVLLYRNGINAPHWNMNANSLMYVVRGEGKVRIVNCEGKAVFDDNVRKGQMLVVPQNFVVAEQA | 5555.97 | C564 H914 N167 O162 S5 |
| Y Fragment | 0 | 3026.6432 | 3026.6507 | 153 | 179 | -2.5 | GLRQNEVTKIKFSGNRGPLVQPRSHAH | 684.91 | C131 H217 N46 O37 S0 |
| Y Fragment | 0 | 3173.7071 | 3173.7191 | 152 | 179 | -3.8 | FGLRQNEVTKIKFSGNRGPLVQPRSHAH | 1650.21 | C140 H226 N47 O38 S0 |
| Y Fragment | 0 | 3244.7496 | 3244.7563 | 151 | 179 | -2.1 | AFGLRQNEVTKIKFSGNRGPLVQPRSHAH | 1794.2 | C143 H231 N48 O39 S0 |
| Y Fragment | 0 | 3358.7895 | 3358.7992 | 150 | 179 | -2.9 | NAFGLRQNEVTKIKFSGNRGPLVQPRSHAH | 3162.69 | C147 H237 N50 O41 S0 |
| Y Fragment | 0 | 3429.8281 | 3429.8363 | 149 | 179 | -2.4 | ANAFGLRQNEVTKIKFSGNRGPLVQPRSHAH | 6407.94 | C150 H242 N51 O42 S0 |
| Y Fragment | 0 | 3542.9148 | 3542.9204 | 148 | 179 | -1.6 | LANAFGLRQNEVTKIKFSGNRGPLVQPRSHAH | 1145.81 | C156 H253 N52 O43 S0 |
| Y Fragment | 0 | 6765.6094 | 6765.6142 | 119 | 179 | -0.7 | GFEYVVFKTNDRAAVSNVKQVFRATPAQVLANAFGLRQNEVTKIKFSGNRGPLVQPRSHAH | 1094.71 | C303 H480 N93 O84 S0 |
| Y Fragment | 0 | 7023.6931 | 7023.6994 | 117 | 179 | -0.9 | EEGFEYVVFKTNDRAAVSNVKQVFRATPAQVLANAFGLRQNEVTKIKFSGNRGPLVQPRSHAH | 1906.66 | C313 H494 N95 O90 S0 |
| Y Fragment | 0 | 7137.7556 | 7137.7423 | 116 | 179 | 1.9 | NEEGFEYVVFKTNDRAAVSNVKQVFRATPAQVLANAFGLRQNEVTKIKFSGNRGPLVQPRSHAH | 1544.62 | C317 H500 N97 O92 S0 |
| Y Fragment | 0 | 7194.7572 | 7194.7638 | 115 | 179 | -0.9 | GNEEGFEYVVFKTNDRAAVSNVKQVFRATPAQVLANAFGLRQNEVTKIKFSGNRGPLVQPRSHAH | 19475.56 | C319 H503 N98 O93 S0 |
| Y Fragment | 0 | 7265.7845 | 7265.8009 | 114 | 179 | -2.3 | AGNEEGFEYVVFKTNDRAAVSNVKQVFRATPAQVLANAFGLRQNEVTKIKFSGNRGPLVQPRSHAH | 16516.54 | C322 H508 N99 O94 S0 |
| Y Fragment | 0 | 7393.8480 | 7393.8595 | 113 | 179 | -1.6 | QAGNEEGFEYVVFKTNDRAAVSNVKQVFRATPAQVLANAFGLRQNEVTKIKFSGNRGPLVQPRSHAH | 3329.89 | C327 H516 N101 O96 S0 |
| Y Fragment | 0 | 7522.8910 | 7522.9021 | 112 | 179 | -1.5 | EQAGNEEGFEYVVFKTNDRAAVSNVKQVFRATPAQVLANAFGLRQNEVTKIKFSGNRGPLVQPRSHAH | 1387.14 | C332 H523 N102 O99 S0 |
| Y Fragment | 0 | 7593.9246 | 7593.9392 | 111 | 179 | -1.9 | AEQAGNEEGFEYVVFKTNDRAAVSNVKQVFRATPAQVLANAFGLRQNEVTKIKFSGNRGPLVQPRSHAH | 13748.04 | C335 H528 N103 O100 S0 |
| Y Fragment | 0 | 7693.0000 | 7693.0076 | 110 | 179 | -1.0 | VAEQAGNEEGFEYVVFKTNDRAAVSNVKQVFRATPAQVLANAFGLRQNEVTKIKFSGNRGPLVQPRSHAH | 40296.74 | C340 H537 N104 O101 S0 |
| Y Fragment | 0 | 7792.0826 | 7792.0760 | 109 | 179 | 0.8 | VVAEQAGNEEGFEYVVFKTNDRAAVSNVKQVFRATPAQVLANAFGLRQNEVTKIKFSGNRGPLVQPRSHAH | 13390.01 | C345 H546 N105 O102 S0 |
| BY Int Fragment | 0 | 598.3185 | 598.3195 | 109 | 114 | -1.7 | VVAEQA | 1291.93 | C26 H44 N7 O9 S0 |
| BY Int Fragment | 0 | 804.4197 | 804.4250 | 123 | 129 | -6.7 | VVFKTND | 2333.56 | C37 H58 N9 O11 S0 |
| BY Int Fragment | 0 | 906.4802 | 906.4866 | 70 | 77 | -7.1 | SLMYVVRG | 8021.03 | C41 H68 N11 O10 S1 |
| BY Int Fragment | 0 | 1758.9284 | 1758.9320 | 88 | 103 | -2.0 | GKAVFDDNVRKGQMLV | 1293.15 | C77 H128 N23 O22 S1 |
| BY Int Fragment | 31.9898 | 1791.9313 | 1791.9170 | 73 | 88 | 8.0 | YVVRGEGKVRIVNCEG | 2524.88 | C76 H127 N24 O22 S1 |
| BY Int Fragment | 0 | 1987.0388 | 1987.0430 | 87 | 104 | -2.1 | EGKAVFDDNVRKGQMLVV | 776.98 | C87 H144 N25 O26 S1 |
| BY Int Fragment | 0 | 6049.1607 | 6049.1368 | 22 | 74 | 3.9 | DLYNPRAGRISTVNSLTLPILSYLRLSAEYVLLYRNGINAPHWNMNANSLMYV | 547.75 | C274 H428 N75 O76 S2 |
| BY Int Fragment | 31.9898 | 7223.7714 | 7223.7020 | 33 | 96 | 9.6 | TVNSLTLPILSYLRLSAEYVLLYRNGINAPHWNMNANSLMYVVRGEGKVRIVNCEGKAVFDDNV | 856.07 | C322 H510 N89 O92 S3 |
| BY Int Fragment | 0 | 7575.9142 | 7575.9789 | 108 | 176 | -8.5 | FVVAEQAGNEEGFEYVVFKTNDRAAVSNVKQVFRATPAQVLANAFGLRQNEVTKIKFSGNRGPLVQPRS | 921.75 | C339 H534 N99 O99 S0 |
| BY Int Fragment | 0 | 7664.9756 | 7665.0340 | 97 | 165 | -7.6 | RKGQMLVVPQNFVVAEQAGNEEGFEYVVFKTNDRAAVSNVKQVFRATPAQVLANAFGLRQNEVTKIKFS | 4424.74 | C344 H545 N98 O99 S1 |
| BY Int Fragment | 0 | 7677.9780 | 7677.9565 | 88 | 157 | 2.8 | GKAVFDDNVRKGQMLVVPQNFVVAEQAGNEEGFEYVVFKTNDRAAVSNVKQVFRATPAQVLANAFGLRQN | 868.37 | C342 H536 N99 O101 S1 |
| BY Int Fragment | 0 | 7764.0252 | 7764.1024 | 96 | 165 | -9.9 | VRKGQMLVVPQNFVVAEQAGNEEGFEYVVFKTNDRAAVSNVKQVFRATPAQVLANAFGLRQNEVTKIKFS | 17977.4 | C349 H554 N99 O100 S1 |
| BY Int Fragment | 0 | 7773.0365 | 7773.0841 | 103 | 173 | -6.1 | VVPQNFVVAEQAGNEEGFEYVVFKTNDRAAVSNVKQVFRATPAQVLANAFGLRQNEVTKIKFSGNRGPLVQ | 799.01 | C349 H549 N100 O102 S0 |
| BY Int Fragment | 31.9898 | 7893.0369 | 7893.0553 | 40 | 108 | -2.3 | PILSYLRLSAEYVLLYRNGINAPHWNMNANSLMYVVRGEGKVRIVNCEGKAVFDDNVRKGQMLVVPQNF | 808.44 | C353 H557 N100 O96 S4 |
| BY Int Fragment | 31.9898 | 8097.0174 | 8096.9844 | 62 | 133 | 4.1 | PHWNMNANSLMYVVRGEGKVRIVNCEGKAVFDDNVRKGQMLVVPQNFVVAEQAGNEEGFEYVVFKTNDRAAV | 653.28 | C356 H553 N102 O105 S4 |
| BY Int Fragment | 31.9898 | 8817.5053 | 8817.5833 | 37 | 114 | -8.9 | LTLPILSYLRLSAEYVLLYRNGINAPHWNMNANSLMYVVRGEGKVRIVNCEGKAVFDDNVRKGQMLVVPQNFVVAEQA | 1318.67 | C395 H629 N110 O109 S4 |
| BY Int Fragment | 31.9898 | 8817.5346 | 8817.5833 | 37 | 114 | -5.5 | LTLPILSYLRLSAEYVLLYRNGINAPHWNMNANSLMYVVRGEGKVRIVNCEGKAVFDDNVRKGQMLVVPQNFVVAEQA | 1767.55 | C395 H629 N110 O109 S4 |
| BY Int Fragment | 31.9898 | 9742.9716 | 9743.0013 | 69 | 156 | -3.1 | NSLMYVVRGEGKVRIVNCEGKAVFDDNVRKGQMLVVPQNFVVAEQAGNEEGFEYVVFKTNDRAAVSNVKQVFRATPAQVLANAFGLRQ | 694.27 | C430 H684 N125 O126 S3 |
| BY Int Fragment | 31.9898 | 9742.9716 | 9743.0013 | 70 | 157 | -3.1 | SLMYVVRGEGKVRIVNCEGKAVFDDNVRKGQMLVVPQNFVVAEQAGNEEGFEYVVFKTNDRAAVSNVKQVFRATPAQVLANAFGLRQN | 694.27 | C430 H684 N125 O126 S3 |
| BY Int Fragment | 31.9898 | 9799.1631 | 9799.1340 | 12 | 97 | 3.0 | RENIIRPARADLYNPRAGRISTVNSLTLPILSYLRLSAEYVLLYRNGINAPHWNMNANSLMYVVRGEGKVRIVNCEGKAVFDDNVR | 590.88 | C432 H696 N131 O122 S3 |
| BY Int Fragment | 89.0113 | 12100.2226 | 12100.2950 | 3 | 108 | -6.0 | EETICSARIRENIIRPARADLYNPRAGRISTVNSLTLPILSYLRLSAEYVLLYRNGINAPHWNMNANSLMYVVRGEGKVRIVNCEGKAVFDDNVRKGQMLVVPQNF | 800.85 | C530 H857 N158 O151 S5 |
| BY Int Fragment | 89.0113 | 12139.3157 | 12139.3787 | 6 | 112 | -5.2 | ICSARIRENIIRPARADLYNPRAGRISTVNSLTLPILSYLRLSAEYVLLYRNGINAPHWNMNANSLMYVVRGEGKVRIVNCEGKAVFDDNVRKGQMLVVPQNFVVAE | 1006.66 | C534 H866 N159 O149 S5 |
| BY Int Fragment | 89.0113 | 12139.3540 | 12139.3787 | 6 | 112 | -2.0 | ICSARIRENIIRPARADLYNPRAGRISTVNSLTLPILSYLRLSAEYVLLYRNGINAPHWNMNANSLMYVVRGEGKVRIVNCEGKAVFDDNVRKGQMLVVPQNFVVAE | 1070.51 | C534 H866 N159 O149 S5 |
| BY Int Fragment | 31.9898 | 12301.3739 | 12301.2753 | 42 | 151 | 8.0 | LSYLRLSAEYVLLYRNGINAPHWNMNANSLMYVVRGEGKVRIVNCEGKAVFDDNVRKGQMLVVPQNFVVAEQAGNEEGFEYVVFKTNDRAAVSNVKQVFRATPAQVLANA | 1341.93 | C547 H859 N156 O158 S4 |

Top-down characterization of the component with the experimental mass 20131.4791 Da was achieved by the MS/MS of its multi-charged ion [M+24H]^24+^ at m/z 840.3639, interpreted by the ClipsMS tool using the criteria above reported for internal fragments. This polypeptide was identified with the region Gly353-Ala532 of the legumin J-like entry with the Acc. No. A0A1S2XVG1 carrying the Cys359 in the carbamidomethylated form and the Cys438 as sulfinic acid. The deconvoluted MS/MS of this ion is reported in Figure S13a. In detail, this spectrum shows a group of signals above m/z 9700 which are attributable to a set of b-fragments carrying both the cysteine residues, respectively modified as carbamidomethyl-cysteine and as sulfinic acid (Figure 13b). Two signals at m/z 4265.2820 and 4378.3606 correspond to the fragments b38 and b39, respectively, with the cysteine 7 (i.e., the Cys359 of the whole legumin) as carbamidomethyl-cysteine. Finally, the peak at m/z 7693.0279 might be related to the putative internal fragment 33-100, including cysteine 86 (i.e., Cys438) as sulfinic acid.


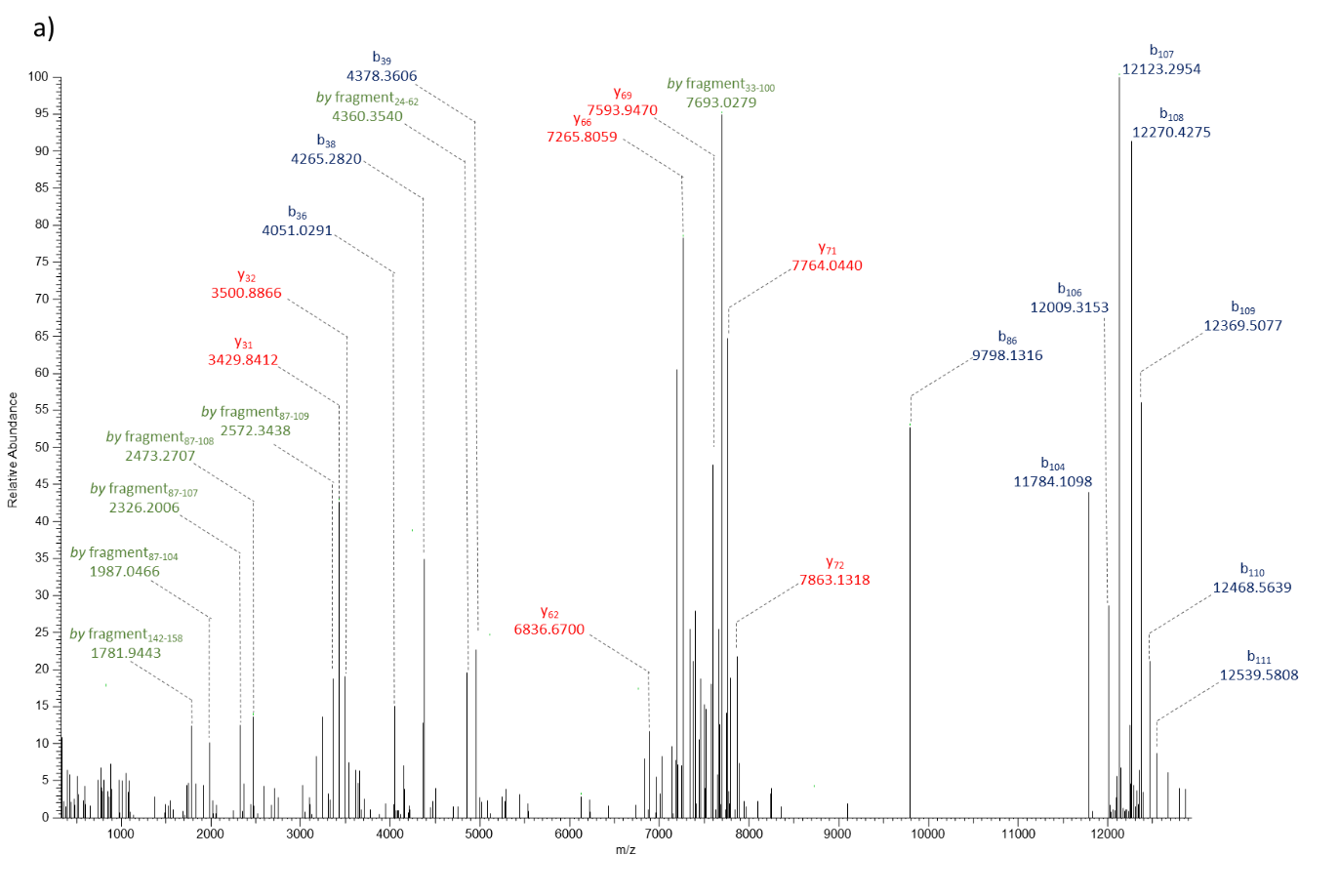


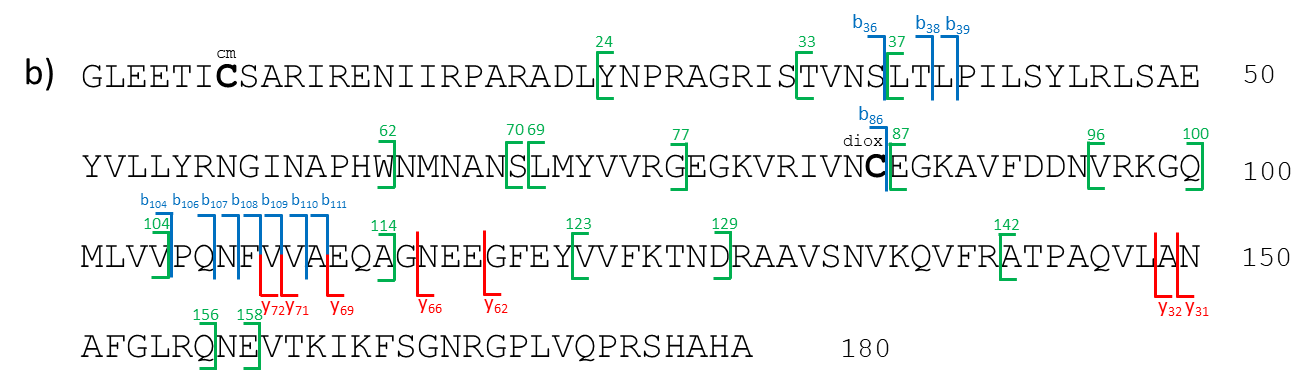


**Figure S13.** a) Deconvoluted MS/MS of the ion at m/z 840.3639, which correspond to the multi-charged ion [M+24H]^24+^ of the polypeptide with m_mono_ 20131.4791 Da. The most abundant y- and b-ions are marked in red and blue, respectively. The most intense internal fragment ions are shown in green. b) Sequence coverage map as obtained by the MS/MS reported in the panel a. This polypeptide corresponds to the region Gly353-Ala532 (here renumbered 1-180) of the legumin J-like entry with Acc. No. A0A1S2XVG1 carrying Cys^7^ (i.e., Cys^359^ in the entire legumin sequence) as carbamidomethyl-cysteine and Cys^86^ (i.e., Cys^438^ of the entire legumin sequence) as sulfinic acid. The cysteines are shown in bold and marked as cm (carbamidomethylated) and di-ox, respectively. b- and y-fragments are reported in blue and red, respectively. The internal fragments are reported in green.

Following the complete report of the ClipsMS interpretation (Fig.S14 and Table S5) of the multi-charged ion [M+24H]^24+^ at m/z 840.3639 of the polypeptide with m_mono_ 20131.4791 Da, which is related to the region Gly^353^-Ala^532^ of the legumin J-like entry with the Acc. No. A0A1S2XVG1 with the Cys^359^ as carbamidomethyl-cysteine and the Cys^438^ as sulfinic acid.


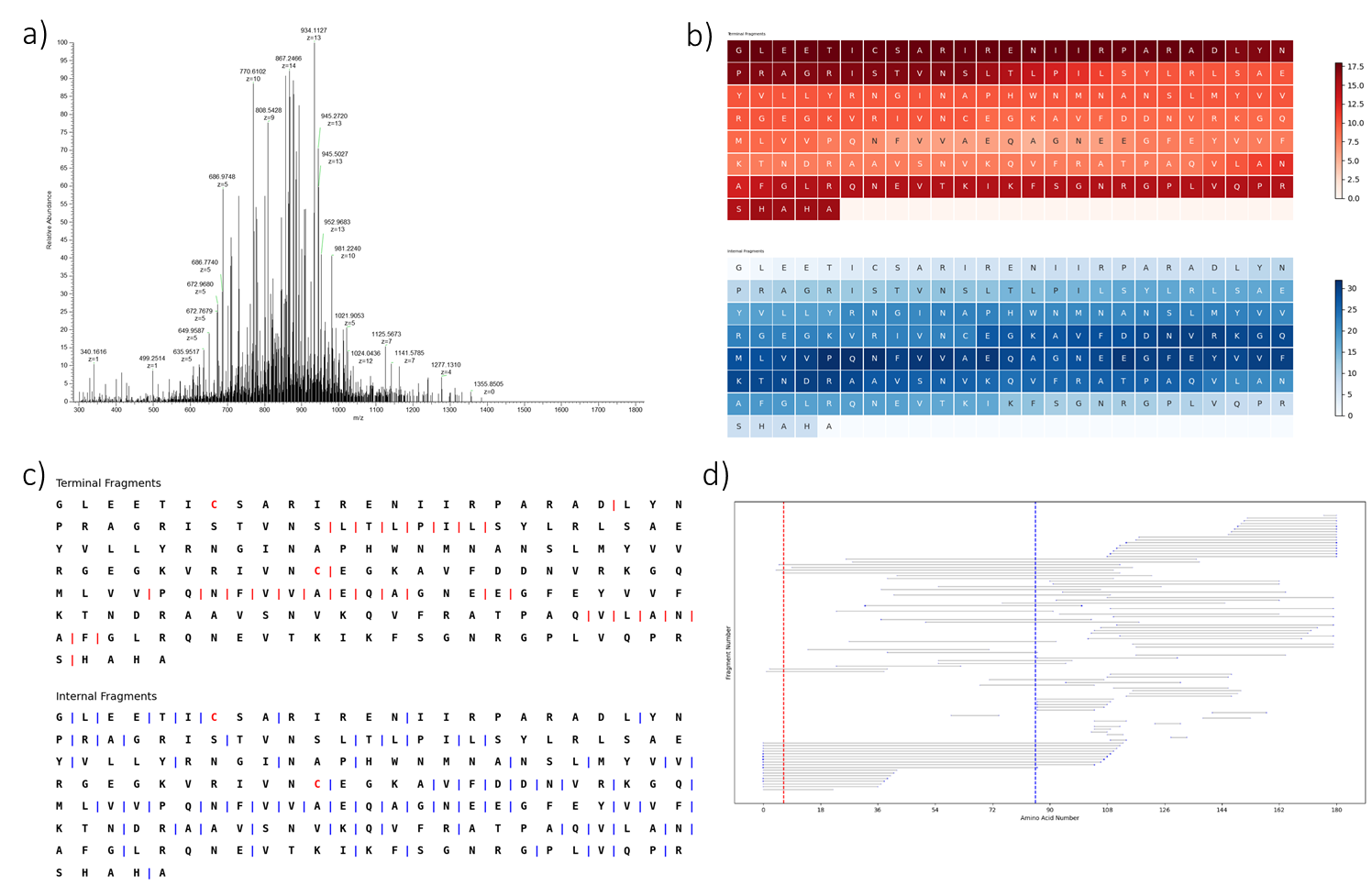


**Figure S14.** a) HCD mass spectrum of the ion at m/z 840.3639 ([M+24H]^24+^); b) sequence coverage map for the terminal and internal fragments. Darker regions indicate more coverage; c) fragment cleavage map indicating the location of inter-amino-acid cleavage sites for terminal and internal fragments); and d) fragment location map indicating the region of the β-chain sequence covered by terminal and internal fragments. The two lines, red and blue, indicate the cysteine residue positions.

**Table S5**. Sample output of ClipsMS related to the CID mass spectrum of the at m/z 840.3639 ([M+24H]^24+^). The Table includes the fragment type, observed mass, theoretical mass, start amino acid, end amino acid, error (ppm), sequence, intensity, and molecular formula. The amino acid positions refer to the sequence reported in Figure S13b in the manuscript.

| **Frag Type** | **Localized Mod** | **Observed Mass** | **Theoretical Mass** | **Start AA** | **End AA** | **Error** | **Sequence** | **Intensity** | **Formula** |
| --- | --- | --- | --- | --- | --- | --- | --- | --- | --- |
| B Fragment | 57.0215 | 2522.3339 | 2522.3257 | 1 | 22 | 3.3 | GLEETICSARIRENIIRPARAD | 490.78 | C103 H178 N35 O33 S1 |
| B Fragment | 57.0215 | 4051.1445 | 4051.1367 | 1 | 36 | 1.9 | GLEETICSARIRENIIRPARADLYNPRAGRISTVNS | 4531 | C169 H286 N57 O53 S1 |
| B Fragment | 57.0215 | 4164.2272 | 4164.2207 | 1 | 37 | 1.5 | GLEETICSARIRENIIRPARADLYNPRAGRISTVNSL | 2903.95 | C175 H297 N58 O54 S1 |
| B Fragment | 57.0215 | 4265.2820 | 4265.2684 | 1 | 38 | 3.2 | GLEETICSARIRENIIRPARADLYNPRAGRISTVNSLT | 25518.38 | C179 H304 N59 O56 S1 |
| B Fragment | 57.0215 | 4378.3606 | 4378.3525 | 1 | 39 | 1.8 | GLEETICSARIRENIIRPARADLYNPRAGRISTVNSLTL | 25885.08 | C185 H315 N60 O57 S1 |
| B Fragment | 57.0215 | 4475.4163 | 4475.4053 | 1 | 40 | 2.5 | GLEETICSARIRENIIRPARADLYNPRAGRISTVNSLTLP | 1560.06 | C190 H322 N61 O58 S1 |
| B Fragment | 57.0215 | 4588.4985 | 4588.4893 | 1 | 41 | 2.0 | GLEETICSARIRENIIRPARADLYNPRAGRISTVNSLTLPI | 6051.36 | C196 H333 N62 O59 S1 |
| B Fragment | 57.0215 | 4701.5815 | 4701.5734 | 1 | 42 | 1.7 | GLEETICSARIRENIIRPARADLYNPRAGRISTVNSLTLPIL | 1157.68 | C202 H344 N63 O60 S1 |
| B Fragment | 89.0113 | 9798.1856 | 9798.1421 | 1 | 86 | 4.4 | GLEETICSARIRENIIRPARADLYNPRAGRISTVNSLTLPILSYLRLSAEYVLLYRNGINAPHWNMNANSLMYVVRGEGKVRIVNC | 39021.16 | C428 H698 N129 O121 S4 |
| B Fragment | 89.0113 | 11784.2042 | 11784.1778 | 1 | 104 | 2.2 | GLEETICSARIRENIIRPARADLYNPRAGRISTVNSLTLPILSYLRLSAEYVLLYRNGINAPHWNMNANSLMYVVRGEGKVRIVNCEGKAVFDDNVRKGQMLVV | 32546.86 | C515 H841 N154 O147 S5 |
| B Fragment | 89.0113 | 12009.3153 | 12009.2892 | 1 | 106 | 2.2 | GLEETICSARIRENIIRPARADLYNPRAGRISTVNSLTLPILSYLRLSAEYVLLYRNGINAPHWNMNANSLMYVVRGEGKVRIVNCEGKAVFDDNVRKGQMLVVPQ | 21248.6 | C525 H856 N157 O150 S5 |
| B Fragment | 89.0113 | 12123.3320 | 12123.3321 | 1 | 107 | 0.0 | GLEETICSARIRENIIRPARADLYNPRAGRISTVNSLTLPILSYLRLSAEYVLLYRNGINAPHWNMNANSLMYVVRGEGKVRIVNCEGKAVFDDNVRKGQMLVVPQN | 74010.9 | C529 H862 N159 O152 S5 |
| B Fragment | 89.0113 | 12270.4275 | 12270.4005 | 1 | 108 | 2.2 | GLEETICSARIRENIIRPARADLYNPRAGRISTVNSLTLPILSYLRLSAEYVLLYRNGINAPHWNMNANSLMYVVRGEGKVRIVNCEGKAVFDDNVRKGQMLVVPQNF | 67672.85 | C538 H871 N160 O153 S5 |
| B Fragment | 89.0113 | 12369.5077 | 12369.4689 | 1 | 109 | 3.1 | GLEETICSARIRENIIRPARADLYNPRAGRISTVNSLTLPILSYLRLSAEYVLLYRNGINAPHWNMNANSLMYVVRGEGKVRIVNCEGKAVFDDNVRKGQMLVVPQNFV | 41545.68 | C543 H880 N161 O154 S5 |
| B Fragment | 89.0113 | 12468.5639 | 12468.5373 | 1 | 110 | 2.1 | GLEETICSARIRENIIRPARADLYNPRAGRISTVNSLTLPILSYLRLSAEYVLLYRNGINAPHWNMNANSLMYVVRGEGKVRIVNCEGKAVFDDNVRKGQMLVVPQNFVV | 15707.51 | C548 H889 N162 O155 S5 |
| B Fragment | 89.0113 | 12539.5808 | 12539.5745 | 1 | 111 | 0.5 | GLEETICSARIRENIIRPARADLYNPRAGRISTVNSLTLPILSYLRLSAEYVLLYRNGINAPHWNMNANSLMYVVRGEGKVRIVNCEGKAVFDDNVRKGQMLVVPQNFVVA | 6478.51 | C551 H894 N163 O156 S5 |
| B Fragment | 89.0113 | 12668.6289 | 12668.6171 | 1 | 112 | 0.9 | GLEETICSARIRENIIRPARADLYNPRAGRISTVNSLTLPILSYLRLSAEYVLLYRNGINAPHWNMNANSLMYVVRGEGKVRIVNCEGKAVFDDNVRKGQMLVVPQNFVVAE | 4610.19 | C556 H901 N164 O159 S5 |
| B Fragment | 89.0113 | 12796.7062 | 12796.6756 | 1 | 113 | 2.4 | GLEETICSARIRENIIRPARADLYNPRAGRISTVNSLTLPILSYLRLSAEYVLLYRNGINAPHWNMNANSLMYVVRGEGKVRIVNCEGKAVFDDNVRKGQMLVVPQNFVVAEQ | 2949.52 | C561 H909 N166 O161 S5 |
| Y Fragment | 0 | 435.2063 | 435.2099 | 177 | 180 | -8.3 | HAHA | 1548.54 | C18 H27 N8 O5 S0 |
| Y Fragment | 0 | 3097.6903 | 3097.6878 | 153 | 180 | 0.8 | GLRQNEVTKIKFSGNRGPLVQPRSHAHA | 2054.38 | C134 H222 N47 O38 S0 |
| Y Fragment | 0 | 3244.7633 | 3244.7563 | 152 | 180 | 2.2 | FGLRQNEVTKIKFSGNRGPLVQPRSHAHA | 10099.43 | C143 H231 N48 O39 S0 |
| Y Fragment | 0 | 3315.7961 | 3315.7934 | 151 | 180 | 0.8 | AFGLRQNEVTKIKFSGNRGPLVQPRSHAHA | 2422.37 | C146 H236 N49 O40 S0 |
| Y Fragment | 0 | 3429.8412 | 3429.8363 | 150 | 180 | 1.4 | NAFGLRQNEVTKIKFSGNRGPLVQPRSHAHA | 31582.94 | C150 H242 N51 O42 S0 |
| Y Fragment | 0 | 3500.8866 | 3500.8734 | 149 | 180 | 3.8 | ANAFGLRQNEVTKIKFSGNRGPLVQPRSHAHA | 14183.7 | C153 H247 N52 O43 S0 |
| Y Fragment | 0 | 3613.9652 | 3613.9575 | 148 | 180 | 2.1 | LANAFGLRQNEVTKIKFSGNRGPLVQPRSHAHA | 4842.26 | C159 H258 N53 O44 S0 |
| Y Fragment | 0 | 3713.0388 | 3713.0259 | 147 | 180 | 3.5 | VLANAFGLRQNEVTKIKFSGNRGPLVQPRSHAHA | 1956.67 | C164 H267 N54 O45 S0 |
| Y Fragment | 0 | 6836.6700 | 6836.6513 | 119 | 180 | 2.7 | GFEYVVFKTNDRAAVSNVKQVFRATPAQVLANAFGLRQNEVTKIKFSGNRGPLVQPRSHAHA | 5922.1 | C306 H485 N94 O85 S0 |
| Y Fragment | 0 | 6965.7131 | 6965.6939 | 118 | 180 | 2.8 | EGFEYVVFKTNDRAAVSNVKQVFRATPAQVLANAFGLRQNEVTKIKFSGNRGPLVQPRSHAHA | 4127.81 | C311 H492 N95 O88 S0 |
| Y Fragment | 0 | 7265.8059 | 7265.8009 | 115 | 180 | 0.7 | GNEEGFEYVVFKTNDRAAVSNVKQVFRATPAQVLANAFGLRQNEVTKIKFSGNRGPLVQPRSHAHA | 57955.94 | C322 H508 N99 O94 S0 |
| Y Fragment | 0 | 7464.8988 | 7464.8966 | 113 | 180 | 0.3 | QAGNEEGFEYVVFKTNDRAAVSNVKQVFRATPAQVLANAFGLRQNEVTKIKFSGNRGPLVQPRSHAHA | 13864.39 | C330 H521 N102 O97 S0 |
| Y Fragment | 0 | 7593.9470 | 7593.9392 | 112 | 180 | 1.0 | EQAGNEEGFEYVVFKTNDRAAVSNVKQVFRATPAQVLANAFGLRQNEVTKIKFSGNRGPLVQPRSHAHA | 35260.34 | C335 H528 N103 O100 S0 |
| Y Fragment | 0 | 7664.9976 | 7664.9763 | 111 | 180 | 2.8 | AEQAGNEEGFEYVVFKTNDRAAVSNVKQVFRATPAQVLANAFGLRQNEVTKIKFSGNRGPLVQPRSHAHA | 18867.11 | C338 H533 N104 O101 S0 |
| Y Fragment | 0 | 7764.0440 | 7764.0447 | 110 | 180 | -0.1 | VAEQAGNEEGFEYVVFKTNDRAAVSNVKQVFRATPAQVLANAFGLRQNEVTKIKFSGNRGPLVQPRSHAHA | 47937.35 | C343 H542 N105 O102 S0 |
| Y Fragment | 0 | 7863.1318 | 7863.1131 | 109 | 180 | 2.4 | VVAEQAGNEEGFEYVVFKTNDRAAVSNVKQVFRATPAQVLANAFGLRQNEVTKIKFSGNRGPLVQPRSHAHA | 16142.95 | C348 H551 N106 O103 S0 |
| BY Int Fragment | 0 | 499.2515 | 499.2511 | 110 | 114 | 0.8 | VAEQA | 7427.11 | C21 H35 N6 O8 S0 |
| BY Int Fragment | 0 | 513.2791 | 513.2780 | 129 | 133 | 2.2 | DRAAV | 4261.02 | C21 H37 N8 O7 S0 |
| BY Int Fragment | 0 | 527.2829 | 527.2824 | 109 | 113 | 0.9 | VVAEQ | 2357.04 | C23 H39 N6 O8 S0 |
| BY Int Fragment | 0 | 586.2998 | 586.2984 | 104 | 108 | 2.4 | VPQNF | 3251.24 | C28 H40 N7 O7 S0 |
| BY Int Fragment | 0 | 586.2998 | 586.2984 | 105 | 109 | 2.4 | PQNFV | 3251.24 | C28 H40 N7 O7 S0 |
| BY Int Fragment | 0 | 885.4483 | 885.4465 | 105 | 112 | 2.0 | PQNFVVAE | 5459.1 | C41 H61 N10 O12 S0 |
| BY Int Fragment | 0 | 932.4936 | 932.4948 | 124 | 131 | -1.4 | VFKTNDRA | 6801.79 | C41 H66 N13 O12 S0 |
| BY Int Fragment | 0 | 1084.5441 | 1084.5422 | 105 | 114 | 1.7 | PQNFVVAEQA | 3754.9 | C49 H74 N13 O15 S0 |
| BY Int Fragment | 0 | 1543.8507 | 1543.8380 | 139 | 153 | 8.2 | VFRATPAQVLANAFG | 1303.25 | C72 H111 N20 O18 S0 |
| BY Int Fragment | 0 | 1743.7775 | 1743.7730 | 60 | 74 | 2.6 | NAPHWNMNANSLMYV | 2780.97 | C77 H111 N22 O21 S2 |
| BY Int Fragment | 0 | 1781.9443 | 1781.9293 | 142 | 158 | 8.4 | ATPAQVLANAFGLRQNE | 9194.38 | C78 H125 N24 O24 S0 |
| BY Int Fragment | 0 | 1987.0466 | 1987.0430 | 87 | 104 | 1.8 | EGKAVFDDNVRKGQMLVV | 7578.44 | C87 H144 N25 O26 S1 |
| BY Int Fragment | 0 | 2326.2006 | 2326.1972 | 87 | 107 | 1.4 | EGKAVFDDNVRKGQMLVVPQN | 9288.32 | C101 H165 N30 O31 S1 |
| BY Int Fragment | 0 | 2473.2707 | 2473.2656 | 87 | 108 | 2.0 | EGKAVFDDNVRKGQMLVVPQNF | 10137.52 | C110 H174 N31 O32 S1 |
| BY Int Fragment | 0 | 2572.3438 | 2572.3341 | 87 | 109 | 3.8 | EGKAVFDDNVRKGQMLVVPQNFV | 8008.92 | C115 H183 N32 O33 S1 |
| BY Int Fragment | 0 | 2671.4120 | 2671.4025 | 87 | 110 | 3.6 | EGKAVFDDNVRKGQMLVVPQNFVV | 1315.32 | C120 H192 N33 O34 S1 |
| BY Int Fragment | 0 | 3652.8395 | 3652.8507 | 115 | 147 | -3.1 | GNEEGFEYVVFKTNDRAAVSNVKQVFRATPAQV | 4712.26 | C163 H251 N46 O50 S0 |
| BY Int Fragment | 0 | 3779.9601 | 3779.9504 | 116 | 149 | 2.6 | NEEGFEYVVFKTNDRAAVSNVKQVFRATPAQVLA | 937.51 | C170 H264 N47 O51 S0 |
| BY Int Fragment | 0 | 3779.9601 | 3779.9504 | 117 | 150 | 2.6 | EEGFEYVVFKTNDRAAVSNVKQVFRATPAQVLAN | 937.51 | C170 H264 N47 O51 S0 |
| BY Int Fragment | 0 | 3952.9627 | 3952.9577 | 111 | 146 | 1.3 | AEQAGNEEGFEYVVFKTNDRAAVSNVKQVFRATPAQ | 1532.58 | C174 H267 N50 O56 S0 |
| BY Int Fragment | 31.9898 | 4037.1013 | 4037.0881 | 69 | 104 | 3.3 | NSLMYVVRGEGKVRIVNCEGKAVFDDNVRKGQMLVV | 1416.32 | C174 H291 N52 O50 S3 |
| BY Int Fragment | 0 | 4051.0291 | 4051.0495 | 96 | 131 | -5.0 | VRKGQMLVVPQNFVVAEQAGNEEGFEYVVFKTNDRA | 11147 | C181 H281 N50 O54 S1 |
| BY Int Fragment | 31.9898 | 4062.1134 | 4062.0834 | 72 | 107 | 7.4 | MYVVRGEGKVRIVNCEGKAVFDDNVRKGQMLVVPQN | 777.7 | C175 H290 N53 O50 S3 |
| BY Int Fragment | 0 | 4151.0994 | 4151.0945 | 109 | 146 | 1.2 | VVAEQAGNEEGFEYVVFKTNDRAAVSNVKQVFRATPAQ | 5237.09 | C184 H285 N52 O58 S0 |
| BY Int Fragment | 0 | 4151.0994 | 4151.0945 | 110 | 147 | 1.2 | VAEQAGNEEGFEYVVFKTNDRAAVSNVKQVFRATPAQV | 5237.09 | C184 H285 N52 O58 S0 |
| BY Int Fragment | 57.0215 | 4208.2575 | 4208.2470 | 2 | 38 | 2.5 | LEETICSARIRENIIRPARADLYNPRAGRISTVNSLT | 1209.74 | C177 H301 N58 O55 S1 |
| BY Int Fragment | 57.0215 | 4208.2575 | 4208.2470 | 3 | 39 | 2.5 | EETICSARIRENIIRPARADLYNPRAGRISTVNSLTL | 1209.74 | C177 H301 N58 O55 S1 |
| BY Int Fragment | 0 | 4360.3540 | 4360.3928 | 24 | 62 | -8.9 | YNPRAGRISTVNSLTLPILSYLRLSAEYVLLYRNGINAP | 9509.2 | C199 H320 N55 O55 S0 |
| BY Int Fragment | 31.9898 | 4502.1615 | 4502.1663 | 56 | 95 | -1.1 | RNGINAPHWNMNANSLMYVVRGEGKVRIVNCEGKAVFDDN | 2944.4 | C192 H302 N61 O57 S3 |
| BY Int Fragment | 31.9898 | 4757.2941 | 4757.3358 | 56 | 97 | -8.8 | RNGINAPHWNMNANSLMYVVRGEGKVRIVNCEGKAVFDDNVR | 1115.17 | C203 H323 N66 O59 S3 |
| BY Int Fragment | 0 | 4955.4102 | 4955.4421 | 87 | 130 | -6.4 | EGKAVFDDNVRKGQMLVVPQNFVVAEQAGNEEGFEYVVFKTNDR | 16786.49 | C220 H337 N60 O69 S1 |
| BY Int Fragment | 0 | 5282.8721 | 5282.8226 | 118 | 164 | 9.4 | EGFEYVVFKTNDRAAVSNVKQVFRATPAQVLANAFGLRQNEVTKIKF | 1780.43 | C241 H378 N67 O67 S0 |
| BY Int Fragment | 31.9898 | 5420.8309 | 5420.7969 | 40 | 86 | 6.3 | PILSYLRLSAEYVLLYRNGINAPHWNMNANSLMYVVRGEGKVRIVNC | 4694.48 | C243 H384 N69 O64 S3 |
| BY Int Fragment | 0 | 6433.4910 | 6433.4619 | 15 | 71 | 4.5 | IIRPARADLYNPRAGRISTVNSLTLPILSYLRLSAEYVLLYRNGINAPHWNMNANSL | 1227.36 | C290 H464 N85 O79 S1 |
| BY Int Fragment | 0 | 6876.6798 | 6876.6462 | 118 | 179 | 4.9 | EGFEYVVFKTNDRAAVSNVKQVFRATPAQVLANAFGLRQNEVTKIKFSGNRGPLVQPRSHAH | 878.62 | C308 H485 N94 O86 S0 |
| BY Int Fragment | 0 | 7005.7060 | 7005.6888 | 117 | 179 | 2.4 | EEGFEYVVFKTNDRAAVSNVKQVFRATPAQVLANAFGLRQNEVTKIKFSGNRGPLVQPRSHAH | 2480.72 | C313 H492 N95 O89 S0 |
| BY Int Fragment | 31.9898 | 7264.8161 | 7264.8125 | 28 | 92 | 0.5 | AGRISTVNSLTLPILSYLRLSAEYVLLYRNGINAPHWNMNANSLMYVVRGEGKVRIVNCEGKAVF | 1899.97 | C325 H521 N92 O89 S3 |
| BY Int Fragment | 0 | 7335.8563 | 7335.8203 | 103 | 169 | 4.9 | VVPQNFVVAEQAGNEEGFEYVVFKTNDRAAVSNVKQVFRATPAQVLANAFGLRQNEVTKIKFSGNRG | 18849.44 | C328 H514 N95 O97 S0 |
| BY Int Fragment | 0 | 7375.8652 | 7375.8489 | 113 | 179 | 2.2 | QAGNEEGFEYVVFKTNDRAAVSNVKQVFRATPAQVLANAFGLRQNEVTKIKFSGNRGPLVQPRSHAH | 15661.75 | C327 H514 N101 O95 S0 |
| BY Int Fragment | 0 | 7446.9069 | 7446.8887 | 104 | 171 | 2.4 | VPQNFVVAEQAGNEEGFEYVVFKTNDRAAVSNVKQVFRATPAQVLANAFGLRQNEVTKIKFSGNRGPL | 7846.22 | C334 H523 N96 O98 S0 |
| BY Int Fragment | 0 | 7446.9069 | 7446.8887 | 105 | 172 | 2.4 | PQNFVVAEQAGNEEGFEYVVFKTNDRAAVSNVKQVFRATPAQVLANAFGLRQNEVTKIKFSGNRGPLV | 7846.22 | C334 H523 N96 O98 S0 |
| BY Int Fragment | 0 | 7446.9069 | 7446.8887 | 107 | 174 | 2.4 | NFVVAEQAGNEEGFEYVVFKTNDRAAVSNVKQVFRATPAQVLANAFGLRQNEVTKIKFSGNRGPLVQP | 7846.22 | C334 H523 N96 O98 S0 |
| BY Int Fragment | 0 | 7504.8996 | 7504.8915 | 112 | 179 | 1.1 | EQAGNEEGFEYVVFKTNDRAAVSNVKQVFRATPAQVLANAFGLRQNEVTKIKFSGNRGPLVQPRSHAH | 11310.41 | C332 H521 N102 O98 S0 |
| BY Int Fragment | 31.9898 | 7513.7541 | 7513.7566 | 52 | 118 | -0.3 | VLLYRNGINAPHWNMNANSLMYVVRGEGKVRIVNCEGKAVFDDNVRKGQMLVVPQNFVVAEQAGNEE | 2930.6 | C328 H520 N97 O96 S4 |
| BY Int Fragment | 31.9898 | 7521.9160 | 7521.8959 | 38 | 103 | 2.7 | TLPILSYLRLSAEYVLLYRNGINAPHWNMNANSLMYVVRGEGKVRIVNCEGKAVFDDNVRKGQMLV | 10940.63 | C335 H536 N95 O92 S4 |
| BY Int Fragment | 0 | 7575.9303 | 7575.9286 | 111 | 179 | 0.2 | AEQAGNEEGFEYVVFKTNDRAAVSNVKQVFRATPAQVLANAFGLRQNEVTKIKFSGNRGPLVQPRSHAH | 13365.9 | C335 H526 N103 O99 S0 |
| BY Int Fragment | 0 | 7631.0225 | 7630.9769 | 94 | 162 | 6.0 | DNVRKGQMLVVPQNFVVAEQAGNEEGFEYVVFKTNDRAAVSNVKQVFRATPAQVLANAFGLRQNEVTKI | 937.55 | C339 H539 N98 O101 S1 |
| BY Int Fragment | 31.9898 | 7647.9731 | 7648.0042 | 24 | 91 | -4.1 | YNPRAGRISTVNSLTLPILSYLRLSAEYVLLYRNGINAPHWNMNANSLMYVVRGEGKVRIVNCEGKAV | 4292.39 | C340 H546 N99 O94 S3 |
| BY Int Fragment | 0 | 7675.0086 | 7674.9970 | 110 | 179 | 1.5 | VAEQAGNEEGFEYVVFKTNDRAAVSNVKQVFRATPAQVLANAFGLRQNEVTKIKFSGNRGPLVQPRSHAH | 9356.34 | C340 H535 N104 O100 S0 |
| BY Int Fragment | 31.9898 | 7693.0279 | 7692.9781 | 33 | 100 | 6.5 | TVNSLTLPILSYLRLSAEYVLLYRNGINAPHWNMNANSLMYVVRGEGKVRIVNCEGKAVFDDNVRKGQ | 70297.88 | C341 H545 N98 O97 S3 |
| BY Int Fragment | 31.9898 | 7738.9007 | 7738.9398 | 76 | 145 | -5.1 | RGEGKVRIVNCEGKAVFDDNVRKGQMLVVPQNFVVAEQAGNEEGFEYVVFKTNDRAAVSNVKQVFRATPA | 917.64 | C339 H539 N100 O102 S2 |
| BY Int Fragment | 0 | 7746.0266 | 7746.0038 | 93 | 162 | 2.9 | DDNVRKGQMLVVPQNFVVAEQAGNEEGFEYVVFKTNDRAAVSNVKQVFRATPAQVLANAFGLRQNEVTKI | 10473.59 | C343 H544 N99 O104 S1 |
| BY Int Fragment | 0 | 7774.0599 | 7774.0654 | 109 | 179 | -0.7 | VVAEQAGNEEGFEYVVFKTNDRAAVSNVKQVFRATPAQVLANAFGLRQNEVTKIKFSGNRGPLVQPRSHAH | 2734.52 | C345 H544 N105 O101 S0 |
| BY Int Fragment | 31.9898 | 7782.0000 | 7781.9869 | 42 | 109 | 1.7 | LSYLRLSAEYVLLYRNGINAPHWNMNANSLMYVVRGEGKVRIVNCEGKAVFDDNVRKGQMLVVPQNFV | 1532.44 | C347 H548 N99 O95 S4 |
| BY Int Fragment | 0 | 7791.0904 | 7791.1133 | 95 | 164 | -2.9 | NVRKGQMLVVPQNFVVAEQAGNEEGFEYVVFKTNDRAAVSNVKQVFRATPAQVLANAFGLRQNEVTKIKF | 6770.09 | C350 H555 N100 O100 S1 |
| BY Int Fragment | 31.9898 | 7846.1224 | 7846.0757 | 38 | 106 | 5.9 | TLPILSYLRLSAEYVLLYRNGINAPHWNMNANSLMYVVRGEGKVRIVNCEGKAVFDDNVRKGQMLVVPQ | 904.78 | C350 H560 N99 O96 S4 |
| BY Int Fragment | 31.9898 | 7866.9036 | 7866.8577 | 56 | 125 | 5.8 | RNGINAPHWNMNANSLMYVVRGEGKVRIVNCEGKAVFDDNVRKGQMLVVPQNFVVAEQAGNEEGFEYVVF | 1212.91 | C346 H535 N100 O101 S4 |
| BY Int Fragment | 0 | 7893.0714 | 7893.0722 | 92 | 162 | -0.1 | FDDNVRKGQMLVVPQNFVVAEQAGNEEGFEYVVFKTNDRAAVSNVKQVFRATPAQVLANAFGLRQNEVTKI | 5517.98 | C352 H553 N100 O105 S1 |
| BY Int Fragment | 0 | 7992.1393 | 7992.1406 | 91 | 162 | -0.2 | VFDDNVRKGQMLVVPQNFVVAEQAGNEEGFEYVVFKTNDRAAVSNVKQVFRATPAQVLANAFGLRQNEVTKI | 7456.47 | C357 H562 N101 O106 S1 |
| BY Int Fragment | 31.9898 | 8091.2294 | 8091.1921 | 40 | 110 | 4.6 | PILSYLRLSAEYVLLYRNGINAPHWNMNANSLMYVVRGEGKVRIVNCEGKAVFDDNVRKGQMLVVPQNFVV | 1686.57 | C363 H575 N102 O98 S4 |
| BY Int Fragment | 31.9898 | 9092.5164 | 9092.4920 | 43 | 122 | 2.7 | SYLRLSAEYVLLYRNGINAPHWNMNANSLMYVVRGEGKVRIVNCEGKAVFDDNVRKGQMLVVPQNFVVAEQAGNEEGFEY | 1533.32 | C403 H622 N113 O118 S4 |
| BY Int Fragment | 89.0113 | 12026.3334 | 12026.2946 | 7 | 112 | 3.2 | CSARIRENIIRPARADLYNPRAGRISTVNSLTLPILSYLRLSAEYVLLYRNGINAPHWNMNANSLMYVVRGEGKVRIVNCEGKAVFDDNVRKGQMLVVPQNFVVAE | 1353.25 | C528 H855 N158 O148 S5 |
| BY Int Fragment | 89.0113 | 12040.2936 | 12040.3466 | 5 | 110 | -4.4 | TICSARIRENIIRPARADLYNPRAGRISTVNSLTLPILSYLRLSAEYVLLYRNGINAPHWNMNANSLMYVVRGEGKVRIVNCEGKAVFDDNVRKGQMLVVPQNFVV | 619.5 | C530 H861 N158 O147 S5 |
| BY Int Fragment | 31.9898 | 12078.2848 | 12078.3548 | 10 | 116 | -5.8 | RIRENIIRPARADLYNPRAGRISTVNSLTLPILSYLRLSAEYVLLYRNGINAPHWNMNANSLMYVVRGEGKVRIVNCEGKAVFDDNVRKGQMLVVPQNFVVAEQAGN | 771.02 | C533 H862 N161 O150 S4 |
| BY Int Fragment | 89.0113 | 12139.3472 | 12139.3787 | 6 | 112 | -2.6 | ICSARIRENIIRPARADLYNPRAGRISTVNSLTLPILSYLRLSAEYVLLYRNGINAPHWNMNANSLMYVVRGEGKVRIVNCEGKAVFDDNVRKGQMLVVPQNFVVAE | 4998.24 | C534 H866 N159 O149 S5 |
| BY Int Fragment | 31.9898 | 12186.3635 | 12186.2583 | 29 | 137 | 8.6 | GRISTVNSLTLPILSYLRLSAEYVLLYRNGINAPHWNMNANSLMYVVRGEGKVRIVNCEGKAVFDDNVRKGQMLVVPQNFVVAEQAGNEEGFEYVVFKTNDRAAVSNVK | 768.52 | C541 H858 N153 O158 S4 |
| BY Int Fragment | 31.9898 | 12285.4130 | 12285.3015 | 27 | 136 | 9.1 | RAGRISTVNSLTLPILSYLRLSAEYVLLYRNGINAPHWNMNANSLMYVVRGEGKVRIVNCEGKAVFDDNVRKGQMLVVPQNFVVAEQAGNEEGFEYVVFKTNDRAAVSNV | 3289.46 | C544 H863 N156 O159 S4 |

***Polypeptides related to the legumin A0A3Q7XNW1***

Figure S15 reports the multi-charged ESI mass spectra of four polypeptides having m_mono_ 20044.2672, 20019.2395, 20035.2252, and 20076.2292 Da, that are related the legumin-like entry with the Acc. No. A0A3Q7XNW1.

**
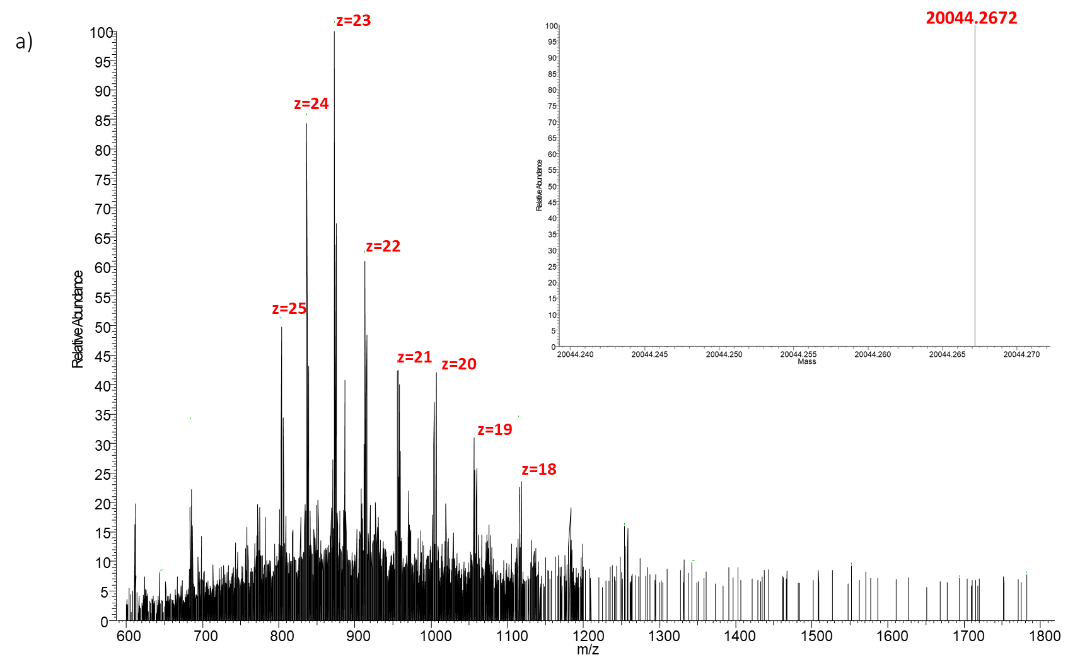
**

**
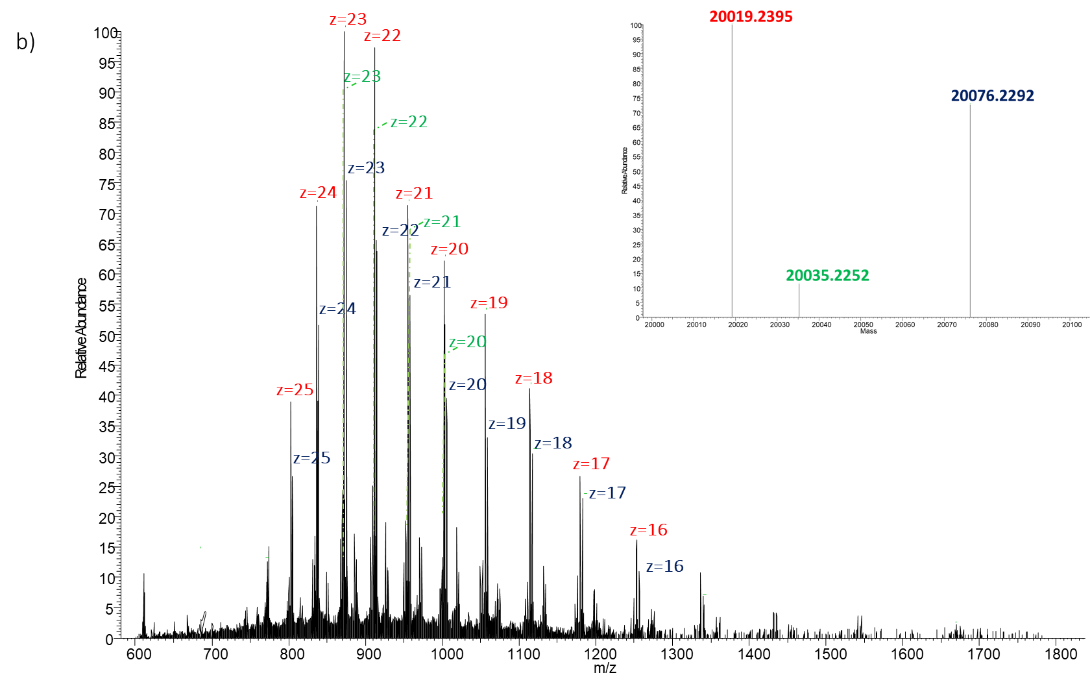
**

**Figure S15.** a) Multi-charged mass spectrum and monoisotopic deconvoluted mass spectrum (mass zero-charge) of the polypeptide with m_mono_ 20044.2672 Da; b) multi-charged mass spectrum and monoisotopic deconvoluted mass spectrum (mass zero-charge) of the three coeluting polypeptides with 20019.2395, 20035.2252 and 20076.2292 Da.

Top-down characterization of the component with the experimental mass 20044.2672 Da was achieved by the MS/MS of its multi-charged ion [M+24H]^24+^ at m/z 836.6845, interpreted by the ClipsMS tool using the criteria above reported for internal fragments. This polypeptide was identified with the region Gly315-Asn496 of the legumin-like entry with the Acc. No. A0A3Q7XNW1 carrying both the cysteine residues, located at 321 and 400 positions, as carbamidomethyl-cysteines. The deconvoluted MS/MS of this ion is reported in Figure S16a. In detail, interpretation of the signals was carried out using the amino acid region Gly315-Asn496 of the entry A0A3Q7XNW1 as a reference sequence, which was renumbered as Gly1-Asn182 (Figure S16b). The signals at m/z 2070.9608, 2176.0017, and 2388.0817 are diagnostic for the presence of the cysteine at position 7 (i.e., Cys321 of the entire legumin sequence) as carbamidomethyl-cysteine. Indeed, they correspond to the b19, b20, and b22 fragments, respectively. Similarly, the signals at m/z 12059.2523 (putative internal fragment Tyr64-Leu173) and 12173.2698 (y111 fragment) confirm that the cysteine residue at position 86 (i.e., Cys400) is carbamidomethylated.


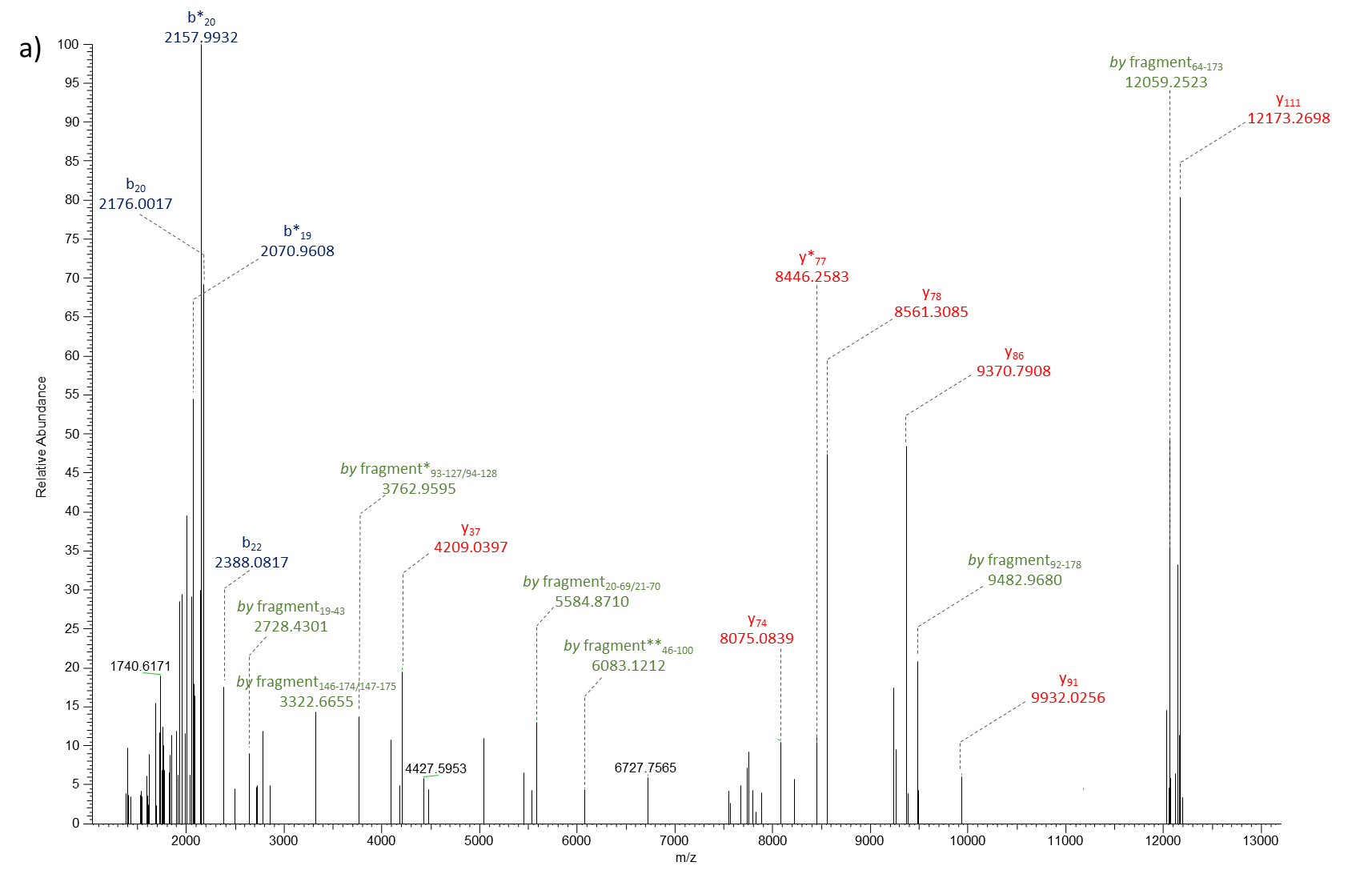


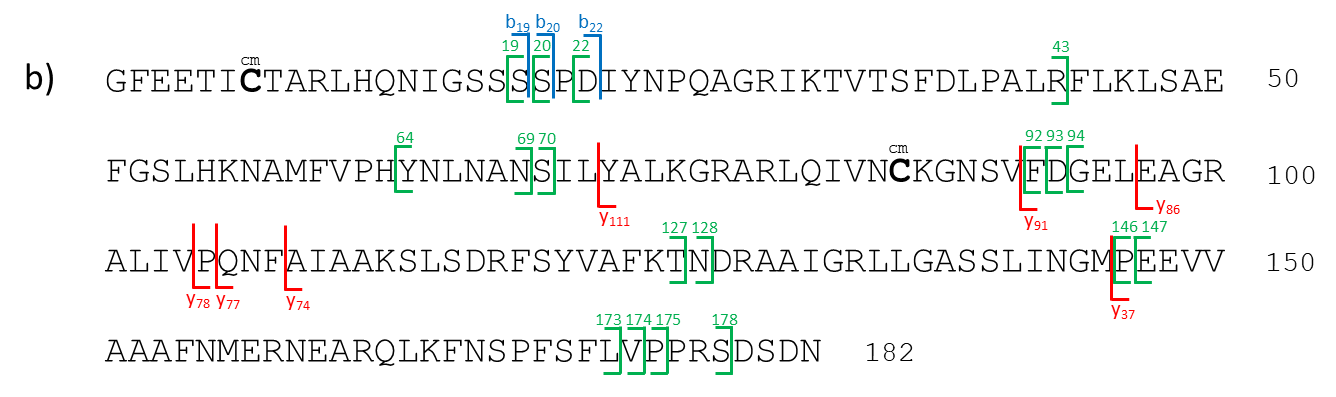


**Figure S16.** a) Deconvoluted MS/MS of the ion at m/z 836.6845, which correspond to the multi-charged ion [M+24H]^24+^ of the polypeptide with m_mono_ 20044.2672 Da. The most abundant y- and b-ions are marked in red and blue, respectively. The most intense internal fragment ions are shown in green. Fragments showing a water loss are marked with an asterisk. Fragments showing an ammonia loss are marked with two asterisks. b) Sequence coverage map as obtained by the MS/MS reported in the panel a. This polypeptide corresponds to the region Gly315-Asn496 (here renumbered 1-182) of the legumin J-like entry with Acc. No. A0A3Q7XNW1 carrying both the Cys^7^ (i.e., Cys^321^ in the entire legumin sequence) and Cys^86^ (i.e., Cys^400^ of the entire legumin sequence) as carbamidomethyl-cysteines. The cysteines are shown in bold and marked as cm (carbamidomethylated). b- and y-fragments are reported in blue and red, respectively. The internal fragments are reported in green.

Following the complete report of the ClipsMS interpretation (Fig. S17, Table S6) of the multi-charged ion [M+24H]^24+^ at m/z 836.6845 of the polypeptide with m_mono_ 20044.2694 Da, which corresponds to the region Gly^315^-Asn^496^ of the legumin-like entry with the Acc. No. A0A3Q7XNW1 carrying both the cysteine residues, located at 321 and 400 positions, as carbamidomethyl-cysteines.


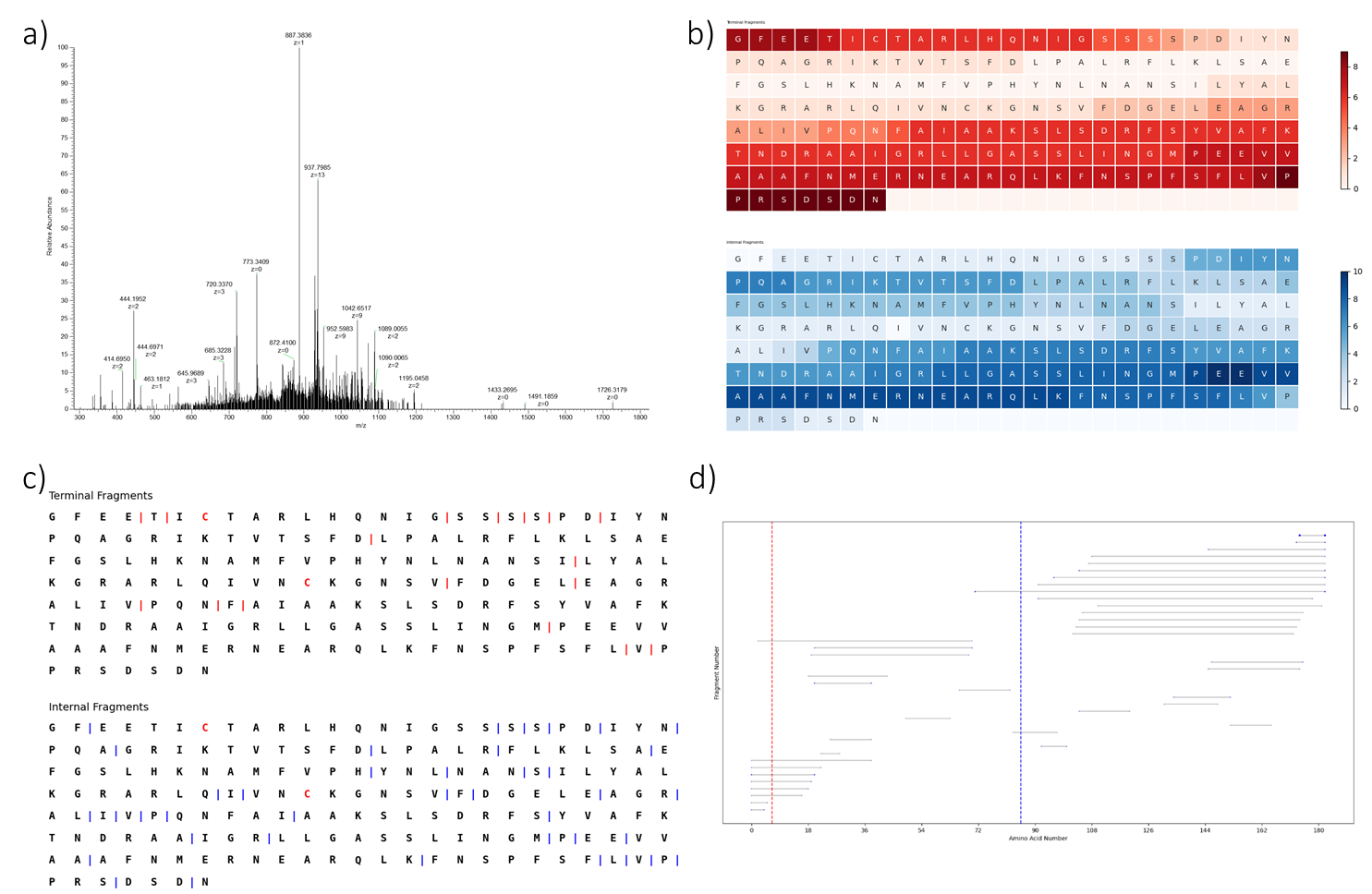


**Figure S17.** a) HCD mass spectrum of the ion at m/z 836.6845 ([M+24H]^24+^); b) sequence coverage map for the terminal and internal fragments. Darker regions indicate more coverage; c) fragment cleavage map indicating the location of inter-amino-acid cleavage sites for terminal and internal fragments); and d) fragment location map indicating the region of the β-chain sequence covered by terminal and internal fragments. The two lines, red and blue, indicate the cysteine residue positions.

**Table S6**. Sample output of ClipsMS related to the CID mass spectrum of the at m/z 836.6845 ([M+24H]^24+^). The Table includes the fragment type, observed mass, theoretical mass, start amino acid, end amino acid, error (ppm), sequence, intensity, and molecular formula. The amino acid positions refer to the sequence reported in Figure 16b in the manuscript.

| **Frag Type** | **Localized Mod** | **Observed Mass** | **Theoretical Mass** | **Start AA** | **End AA** | **Error** | **Sequence** | **Intensity** | **Formula** |
| --- | --- | --- | --- | --- | --- | --- | --- | --- | --- |
| B Fragment | 0 | 463.1813 | 463.1823 | 1 | 4 | -2.3 | GFEE | 2330.49 | C21 H27 N4 O8 S0 |
| B Fragment | 0 | 564.2288 | 564.2300 | 1 | 5 | -2.2 | GFEET | 2382.39 | C25 H34 N5 O10 S0 |
| B Fragment | 57.0215 | 1827.8746 | 1827.8807 | 1 | 16 | -3.3 | GFEETICTARLHQNIG | 1212.96 | C76 H120 N23 O24 S1 |
| B Fragment | 57.0215 | 2001.9425 | 2001.9447 | 1 | 18 | -1.1 | GFEETICTARLHQNIGSS | 1923.28 | C82 H130 N25 O28 S1 |
| B Fragment | 57.0215 | 2088.9681 | 2088.9768 | 1 | 19 | -4.2 | GFEETICTARLHQNIGSSS | 2259.56 | C85 H135 N26 O30 S1 |
| B Fragment | 57.0215 | 2176.0017 | 2176.0088 | 1 | 20 | -3.3 | GFEETICTARLHQNIGSSSS | 9476.94 | C88 H140 N27 O32 S1 |
| B Fragment | 57.0215 | 2388.0817 | 2388.0885 | 1 | 22 | -2.9 | GFEETICTARLHQNIGSSSSPD | 2411.28 | C97 H152 N29 O36 S1 |
| B Fragment | 57.0215 | 4179.0161 | 4179.0200 | 1 | 38 | -0.9 | GFEETICTARLHQNIGSSSSPDIYNPQAGRIKTVTSFD | 676.15 | C178 H278 N51 O60 S1 |
| BY Int Fragment | 0 | 687.3529 | 687.3461 | 23 | 28 | 9.9 | IYNPQA | 654.19 | C32 H47 N8 O9 S0 |
| BY Int Fragment | 0 | 828.3828 | 828.3846 | 93 | 100 | -2.2 | DGELEAGR | 2332.01 | C33 H54 N11 O14 S0 |
| BY Int Fragment | 0 | 1401.7471 | 1401.7485 | 26 | 38 | -1.0 | PQAGRIKTVTSFD | 1336.42 | C62 H101 N18 O19 S0 |
| BY Int Fragment | 57.0215 | 1549.6876 | 1549.6952 | 84 | 97 | -4.9 | VNCKGNSVFDGELE | 486.85 | C63 H98 N17 O23 S1 |
| BY Int Fragment | 0 | 1588.8039 | 1588.8013 | 153 | 165 | 1.6 | AFNMERNEARQLK | 429.4 | C67 H110 N23 O20 S1 |
| BY Int Fragment | 0 | 1595.7751 | 1595.7787 | 50 | 63 | -2.3 | EFGSLHKNAMFVPH | 838.81 | C74 H107 N20 O18 S1 |
| BY Int Fragment | 0 | 1733.9082 | 1733.8969 | 105 | 120 | 6.5 | PQNFAIAAKSLSDRFS | 1605.61 | C78 H121 N22 O23 S0 |
| BY Int Fragment | 0 | 1738.9089 | 1738.9156 | 132 | 148 | -3.9 | IGRLLGASSLINGMPEE | 1213.45 | C75 H128 N21 O24 S1 |
| BY Int Fragment | 0 | 1752.9108 | 1752.9201 | 135 | 152 | -5.3 | LLGASSLINGMPEEVVAA | 1699.79 | C77 H130 N19 O25 S1 |
| BY Int Fragment | 0 | 1770.0152 | 1770.0133 | 67 | 82 | 1.1 | NANSILYALKGRARLQ | 1384.13 | C78 H133 N26 O21 S0 |
| BY Int Fragment | 0 | 2004.0133 | 2004.0185 | 21 | 38 | -2.6 | PDIYNPQAGRIKTVTSFD | 5421.92 | C90 H139 N24 O28 S0 |
| BY Int Fragment | 0 | 2728.4301 | 2728.4417 | 19 | 43 | -4.3 | SSPDIYNPQAGRIKTVTSFDLPALR | 674.9 | C122 H195 N34 O37 S0 |
| BY Int Fragment | 0 | 3322.6655 | 3322.6678 | 146 | 174 | -0.7 | PEEVVAAAFNMERNEARQLKFNSPFSFLV | 1975.85 | C151 H229 N40 O43 S1 |
| BY Int Fragment | 0 | 3322.6655 | 3322.6678 | 147 | 175 | -0.7 | EEVVAAAFNMERNEARQLKFNSPFSFLVP | 1975.85 | C151 H229 N40 O43 S1 |
| BY Int Fragment | 0 | 5584.8710 | 5584.8951 | 20 | 69 | -4.3 | SPDIYNPQAGRIKTVTSFDLPALRFLKLSAEFGSLHKNAMFVPHYNLNAN | 1799.73 | C256 H392 N69 O70 S1 |
| BY Int Fragment | 0 | 5584.8710 | 5584.8951 | 21 | 70 | -4.3 | PDIYNPQAGRIKTVTSFDLPALRFLKLSAEFGSLHKNAMFVPHYNLNANS | 1799.73 | C256 H392 N69 O70 S1 |
| BY Int Fragment | 57.0215 | 7555.8238 | 7555.8067 | 3 | 70 | 2.3 | EETICTARLHQNIGSSSSPDIYNPQAGRIKTVTSFDLPALRFLKLSAEFGSLHKNAMFVPHYNLNANS | 572 | C333 H519 N94 O100 S2 |
| BY Int Fragment | 0 | 7674.9271 | 7674.9530 | 103 | 172 | -3.4 | IVPQNFAIAAKSLSDRFSYVAFKTNDRAAIGRLLGASSLINGMPEEVVAAAFNMERNEARQLKFNSPFSF | 668.81 | C345 H539 N96 O99 S2 |
| BY Int Fragment | 0 | 7674.9271 | 7674.9530 | 104 | 173 | -3.4 | VPQNFAIAAKSLSDRFSYVAFKTNDRAAIGRLLGASSLINGMPEEVVAAAFNMERNEARQLKFNSPFSFL | 668.81 | C345 H539 N96 O99 S2 |
| BY Int Fragment | 0 | 7674.9271 | 7674.9530 | 105 | 174 | -3.4 | PQNFAIAAKSLSDRFSYVAFKTNDRAAIGRLLGASSLINGMPEEVVAAAFNMERNEARQLKFNSPFSFLV | 668.81 | C345 H539 N96 O99 S2 |
| BY Int Fragment | 0 | 7674.9271 | 7674.9530 | 106 | 175 | -3.4 | QNFAIAAKSLSDRFSYVAFKTNDRAAIGRLLGASSLINGMPEEVVAAAFNMERNEARQLKFNSPFSFLVP | 668.81 | C345 H539 N96 O99 S2 |
| BY Int Fragment | 0 | 7758.8832 | 7758.9337 | 111 | 181 | -6.5 | AAKSLSDRFSYVAFKTNDRAAIGRLLGASSLINGMPEEVVAAAFNMERNEARQLKFNSPFSFLVPPRSDSD | 1279.02 | C343 H539 N98 O104 S2 |
| BY Int Fragment | 0 | 9482.8680 | 9482.9110 | 92 | 178 | -4.5 | FDGELEAGRALIVPQNFAIAAKSLSDRFSYVAFKTNDRAAIGRLLGASSLINGMPEEVVAAAFNMERNEARQLKFNSPFSFLVPPRS | 2849.96 | C426 H668 N119 O123 S2 |
| Y Fragment | 0 | 887.3836 | 887.3854 | 175 | 182 | -2.0 | PPRSDSDN | 52061.67 | C34 H55 N12 O16 S0 |
| Y Fragment | 0 | 986.4514 | 986.4538 | 174 | 182 | -2.4 | VPPRSDSDN | 7798.06 | C39 H64 N13 O17 S0 |
| Y Fragment | 0 | 4209.0397 | 4209.0458 | 146 | 182 | -1.5 | PEEVVAAAFNMERNEARQLKFNSPFSFLVPPRSDSDN | 2684.19 | C185 H283 N52 O59 S1 |
| Y Fragment | 0 | 8075.0839 | 8075.1084 | 109 | 182 | -3.0 | AIAAKSLSDRFSYVAFKTNDRAAIGRLLGASSLINGMPEEVVAAAFNMERNEARQLKFNSPFSFLVPPRSDSDN | 1427.48 | C356 H563 N102 O109 S2 |
| Y Fragment | 0 | 8222.1522 | 8222.1768 | 108 | 182 | -3.0 | FAIAAKSLSDRFSYVAFKTNDRAAIGRLLGASSLINGMPEEVVAAAFNMERNEARQLKFNSPFSFLVPPRSDSDN | 786.73 | C365 H572 N103 O110 S2 |
| Y Fragment | 0 | 8561.3085 | 8561.3310 | 105 | 182 | -2.6 | PQNFAIAAKSLSDRFSYVAFKTNDRAAIGRLLGASSLINGMPEEVVAAAFNMERNEARQLKFNSPFSFLVPPRSDSDN | 6499.45 | C379 H593 N108 O115 S2 |
| Y Fragment | 0 | 9370.7908 | 9370.8070 | 97 | 182 | -1.7 | EAGRALIVPQNFAIAAKSLSDRFSYVAFKTNDRAAIGRLLGASSLINGMPEEVVAAAFNMERNEARQLKFNSPFSFLVPPRSDSDN | 6637.9 | C415 H656 N119 O125 S2 |
| Y Fragment | 0 | 9932.0256 | 9932.0505 | 92 | 182 | -2.5 | FDGELEAGRALIVPQNFAIAAKSLSDRFSYVAFKTNDRAAIGRLLGASSLINGMPEEVVAAAFNMERNEARQLKFNSPFSFLVPPRSDSDN | 835.37 | C441 H691 N124 O134 S2 |
| Y Fragment | 57.0215 | 12173.2698 | 12173.3033 | 72 | 182 | -2.8 | LYALKGRARLQIVNCKGNSVFDGELEAGRALIVPQNFAIAAKSLSDRFSYVAFKTNDRAAIGRLLGASSLINGMPEEVVAAAFNMERNEARQLKFNSPFSFLVPPRSDSDN | 11014.33 | C537 H856 N155 O159 S3 |
| B Fragment* | 57.0215 | 1983.9272 | 1983.9341 | 1 | 18 | -3.5 | GFEETICTARLHQNIGSS | 1585.28 | C82 H130 N25 O28 S1 |
| B Fragment* | 57.0215 | 2070.9608 | 2070.9662 | 1 | 19 | -2.6 | GFEETICTARLHQNIGSSS | 7473.26 | C85 H135 N26 O30 S1 |
| B Fragment* | 57.0215 | 2157.9932 | 2157.9982 | 1 | 20 | -2.3 | GFEETICTARLHQNIGSSSS | 13679.35 | C88 H140 N27 O32 S1 |
| B Fragment* | 57.0215 | 7741.8748 | 7741.8860 | 1 | 70 | -1.4 | GFEETICTARLHQNIGSSSSPDIYNPQAGRIKTVTSFDLPALRFLKLSAEFGSLHKNAMFVPHYNLNANS | 994.68 | C344 H531 N96 O102 S2 |
| BY Int Fragment** | 57.0215 | 686.3258 | 686.3290 | 5 | 10 | -4.7 | TICTAR | 571.13 | C26 H48 N9 O8 S1 |
| BY Int Fragment** | 0 | 773.3408 | 773.3352 | 91 | 97 | 7.3 | VFDGELE | 17433.5 | C36 H52 N7 O13 S0 |
| BY Int Fragment* | 0 | 828.3828 | 828.3861 | 58 | 64 | -4.0 | AMFVPHY | 2332.01 | C42 H56 N9 O8 S1 |
| BY Int Fragment** | 57.0215 | 896.4694 | 896.4658 | 81 | 88 | 3.9 | LQIVNCKG | 1036.55 | C37 H66 N11 O10 S1 |
| BY Int Fragment** | 0 | 1403.7606 | 1403.7681 | 41 | 53 | -5.4 | ALRFLKLSAEFGS | 513.54 | C67 H106 N17 O17 S0 |
| BY Int Fragment* | 57.0215 | 1435.6739 | 1435.6747 | 3 | 14 | -0.6 | EETICTARLHQN | 487.4 | C57 H94 N19 O20 S1 |
| BY Int Fragment** | 57.0215 | 1595.7751 | 1595.7595 | 6 | 20 | 9.8 | ICTARLHQNIGSSSS | 838.81 | C63 H107 N22 O22 S1 |
| BY Int Fragment** | 57.0215 | 1693.8078 | 1693.7962 | 3 | 17 | 6.8 | EETICTARLHQNIGS | 320.21 | C68 H113 N22 O24 S1 |
| BY Int Fragment** | 0 | 1747.9009 | 1747.9166 | 160 | 174 | -9.0 | EARQLKFNSPFSFLV | 941.76 | C84 H126 N21 O21 S0 |
| BY Int Fragment* | 0 | 1752.9108 | 1752.9068 | 107 | 122 | 2.3 | NFAIAAKSLSDRFSYV | 1699.79 | C82 H124 N21 O23 S0 |
| BY Int Fragment* | 0 | 1820.0498 | 1820.0501 | 125 | 142 | -0.2 | KTNDRAAIGRLLGASSLI | 904.24 | C79 H141 N26 O24 S0 |
| BY Int Fragment** | 0 | 1849.8725 | 1849.8827 | 10 | 26 | -5.5 | RLHQNIGSSSSPDIYNP | 1562.18 | C80 H124 N25 O27 S0 |
| BY Int Fragment* | 57.0215 | 1897.9830 | 1897.9814 | 77 | 93 | 0.8 | GRARLQIVNCKGNSVFD | 1632.88 | C79 H132 N27 O23 S1 |
| BY Int Fragment* | 57.0215 | 1897.9830 | 1897.9814 | 78 | 94 | 0.8 | RARLQIVNCKGNSVFDG | 1632.88 | C79 H132 N27 O23 S1 |
| BY Int Fragment* | 57.0215 | 1953.9066 | 1953.9083 | 3 | 20 | -0.9 | EETICTARLHQNIGSSSS | 4034.03 | C77 H128 N25 O30 S1 |
| BY Int Fragment* | 0 | 2638.3568 | 2638.3776 | 104 | 127 | -7.9 | VPQNFAIAAKSLSDRFSYVAFKTN | 1248.1 | C123 H187 N32 O34 S0 |
| BY Int Fragment** | 0 | 2728.2632 | 2728.2817 | 137 | 162 | -6.8 | GASSLINGMPEEVVAAAFNMERNEAR | 533.06 | C115 H186 N35 O39 S2 |
| BY Int Fragment** | 0 | 2728.2632 | 2728.2817 | 139 | 163 | -6.8 | SSLINGMPEEVVAAAFNMERNEARQ | 533.06 | C115 H186 N35 O39 S2 |
| BY Int Fragment* | 0 | 3762.9596 | 3762.9602 | 93 | 127 | -0.2 | DGELEAGRALIVPQNFAIAAKSLSDRFSYVAFKTN | 1889.31 | C171 H267 N46 O51 S0 |
| BY Int Fragment* | 0 | 3762.9596 | 3762.9602 | 94 | 128 | -0.2 | GELEAGRALIVPQNFAIAAKSLSDRFSYVAFKTND | 1889.31 | C171 H267 N46 O51 S0 |
| BY Int Fragment** | 57.0215 | 6083.1212 | 6083.1283 | 46 | 100 | -1.2 | KLSAEFGSLHKNAMFVPHYNLNANSILYALKGRARLQIVNCKGNSVFDGELEAGR | 612.26 | C269 H426 N79 O76 S2 |
| BY Int Fragment** | 0 | 7555.8238 | 7555.8430 | 112 | 180 | -2.5 | AKSLSDRFSYVAFKTNDRAAIGRLLGASSLINGMPEEVVAAAFNMERNEARQLKFNSPFSFLVPPRSDS | 572 | C336 H529 N96 O100 S2 |
| BY Int Fragment* | 57.0215 | 7797.9316 | 7797.9486 | 2 | 71 | -2.2 | FEETICTARLHQNIGSSSSPDIYNPQAGRIKTVTSFDLPALRFLKLSAEFGSLHKNAMFVPHYNLNANSI | 600.73 | C348 H539 N96 O102 S2 |
| BY Int Fragment** | 57.0215 | 8075.0839 | 8075.0800 | 2 | 73 | 0.5 | FEETICTARLHQNIGSSSSPDIYNPQAGRIKTVTSFDLPALRFLKLSAEFGSLHKNAMFVPHYNLNANSILY | 1427.48 | C363 H559 N98 O105 S2 |
| BY Int Fragment* | 57.0215 | 9257.7417 | 9257.7997 | 15 | 99 | -6.3 | IGSSSSPDIYNPQAGRIKTVTSFDLPALRFLKLSAEFGSLHKNAMFVPHYNLNANSILYALKGRARLQIVNCKGNSVFDGELEAG | 1307.73 | C414 H652 N115 O120 S2 |
| BY Int Fragment** | 0 | 9482.8680 | 9482.8634 | 88 | 175 | 0.5 | GNSVFDGELEAGRALIVPQNFAIAAKSLSDRFSYVAFKTNDRAAIGRLLGASSLINGMPEEVVAAAFNMERNEARQLKFNSPFSFLVP | 2849.96 | C426 H667 N118 O125 S2 |
| BY Int Fragment* | 57.0215 | 12035.2501 | 12035.3086 | 11 | 120 | -4.9 | LHQNIGSSSSPDIYNPQAGRIKTVTSFDLPALRFLKLSAEFGSLHKNAMFVPHYNLNANSILYALKGRARLQIVNCKGNSVFDGELEAGRALIVPQNFAIAAKSLSDRFS | 2007.44 | C539 H852 N153 O154 S2 |
| BY Int Fragment* | 57.0215 | 12059.2523 | 12059.2756 | 64 | 173 | -1.9 | YNLNANSILYALKGRARLQIVNCKGNSVFDGELEAGRALIVPQNFAIAAKSLSDRFSYVAFKTNDRAAIGRLLGASSLINGMPEEVVAAAFNMERNEARQLKFNSPFSFL | 6742.71 | C537 H852 N153 O155 S3 |
| BY Int Fragment** | 57.0215 | 12148.2575 | 12148.2328 | 48 | 159 | 2.0 | SAEFGSLHKNAMFVPHYNLNANSILYALKGRARLQIVNCKGNSVFDGELEAGRALIVPQNFAIAAKSLSDRFSYVAFKTNDRAAIGRLLGASSLINGMPEEVVAAAFNMERN | 4557.48 | C538 H852 N155 O156 S4 |
| BY Int Fragment* | 57.0215 | 12160.3124 | 12160.4113 | 43 | 154 | -8.1 | RFLKLSAEFGSLHKNAMFVPHYNLNANSILYALKGRARLQIVNCKGNSVFDGELEAGRALIVPQNFAIAAKSLSDRFSYVAFKTNDRAAIGRLLGASSLINGMPEEVVAAAF | 1551.95 | C547 H867 N154 O152 S3 |
| BY Int Fragment* | 57.0215 | 12189.2565 | 12189.2593 | 49 | 160 | -0.2 | AEFGSLHKNAMFVPHYNLNANSILYALKGRARLQIVNCKGNSVFDGELEAGRALIVPQNFAIAAKSLSDRFSYVAFKTNDRAAIGRLLGASSLINGMPEEVVAAAFNMERNE | 467.7 | C540 H854 N155 O157 S4 |
| BY Int Fragment* | 57.0215 | 12189.2565 | 12189.2593 | 50 | 161 | -0.2 | EFGSLHKNAMFVPHYNLNANSILYALKGRARLQIVNCKGNSVFDGELEAGRALIVPQNFAIAAKSLSDRFSYVAFKTNDRAAIGRLLGASSLINGMPEEVVAAAFNMERNEA | 467.7 | C540 H854 N155 O157 S4 |
| Y Fragment** | 0 | 4094.9801 | 4094.9665 | 147 | 182 | 3.3 | EEVVAAAFNMERNEARQLKFNSPFSFLVPPRSDSDN | 1477.32 | C180 H276 N51 O58 S1 |
| Y Fragment* | 0 | 8446.2583 | 8446.2677 | 106 | 182 | -1.1 | QNFAIAAKSLSDRFSYVAFKTNDRAAIGRLLGASSLINGMPEEVVAAAFNMERNEARQLKFNSPFSFLVPPRSDSDN | 1521.52 | C374 H586 N107 O114 S2 |

Top-down characterization of the component with the experimental mass 20019.2395 Da was achieved by the MS/MS of its multi-charged ion [M+22H]^22+^ at m/z 911.4741, interpreted by the ClipsMS tool using the criteria above reported for internal fragments. This polypeptide was identified with the region Gly315-Asn496 of the legumin-like entry with the Acc. No. A0A3Q7XNW1 carrying Cys321 as carbamidomethyl-cysteine and Cys400 as sulfinic acid. The deconvoluted MS/MS of this ion is reported in Figure S18a. In detail, peaks at m/z 2176.0026, 2388.0835, and 4179.0132 are related to b20, b22, and b38 fragments, confirming the carbamidomethylated form of the cysteine at position 7 (i.e., Cys312 of the entire legumin sequence) (Figure S18b). Instead, the signal at m/z 12148.2133 (y111 fragment) allowed us to confirm the hypothesis of the sulfinic acid form of the cysteine 86 (i.e. Cys400). Moreover, a group of signals at m/z 5585.8869, 9128.7191, 9369.8040, 11260.8723, and 12034.1718 which might be assigned to the internal fragments 82-133, 48-130, 48-133, 46-149, and 64-143, respectively, and could confirm the presence of the cysteine 86 as sulfinic acid.


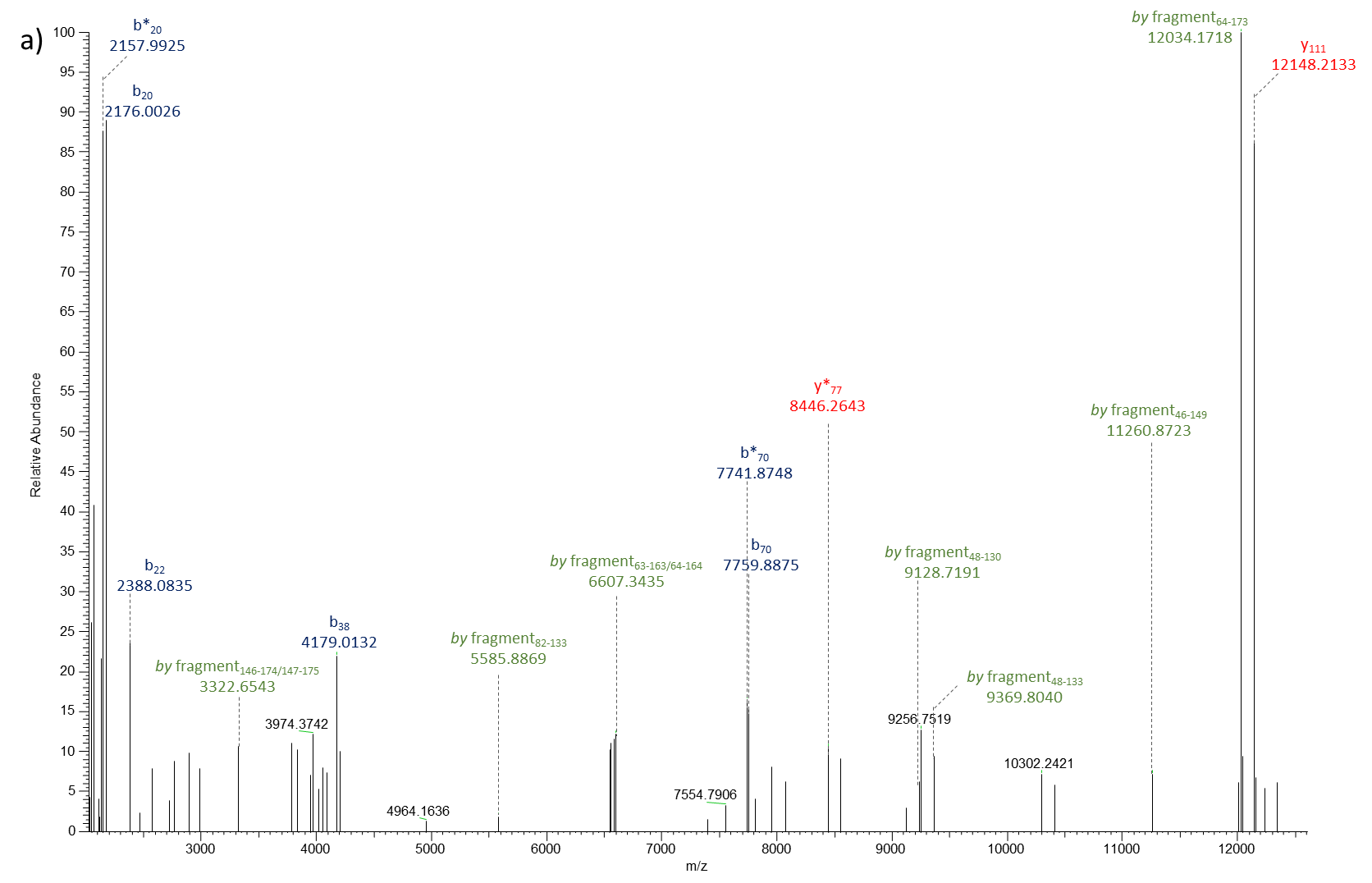


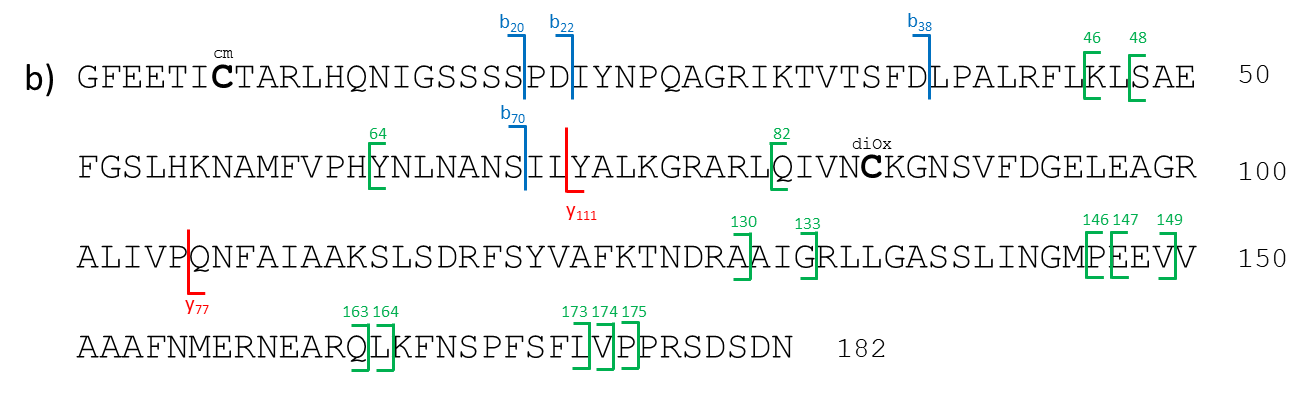


**Figure S18.** a) Deconvoluted MS/MS of the ion at m/z 911.4741, which correspond to the multi-charged ion [M+22H]^22+^ of the polypeptide with m_mono_ 20019.2395 Da. The most abundant y- and b-ions are marked in red and blue, respectively. The most intense internal fragment ions are shown in green. Fragments showing a water loss are marked with an asterisk. b) Sequence coverage map as obtained by the MS/MS shown in the panel a. This polypeptide corresponds to the region Gly315-Asn496 (here renumbered 1-182) of the legumin J-like entry with Acc. No. A0A3Q7XNW1 carrying the Cys^7^ (i.e., Cys^321^ in the entire legumin sequence) as carbamidomethyl-cysteine, and the Cys^86^ (i.e., Cys^400^ of the entire legumin sequence) as sulfinic acid, respectively. The cysteines are shown in bold and marked as cm (carbamidomethylated). b- and y-fragments are reported in blue and red, respectively. The internal fragments are reported in green.

Following the complete report of the ClipsMS interpretation (Fig. S19, Table S7) of the multi-charged ion [M+22H]^22+^ at m/z 911.4741 of the polypeptide with m_mono_ 20019.2395 Da, which fits with the Gly^315^-Asn^496^ region of the legumin with Acc. No. A0A3Q7XNW1 with Cys^321^ as carbamidomethyl-cysteine and Cys^400^ as sulfinic acid


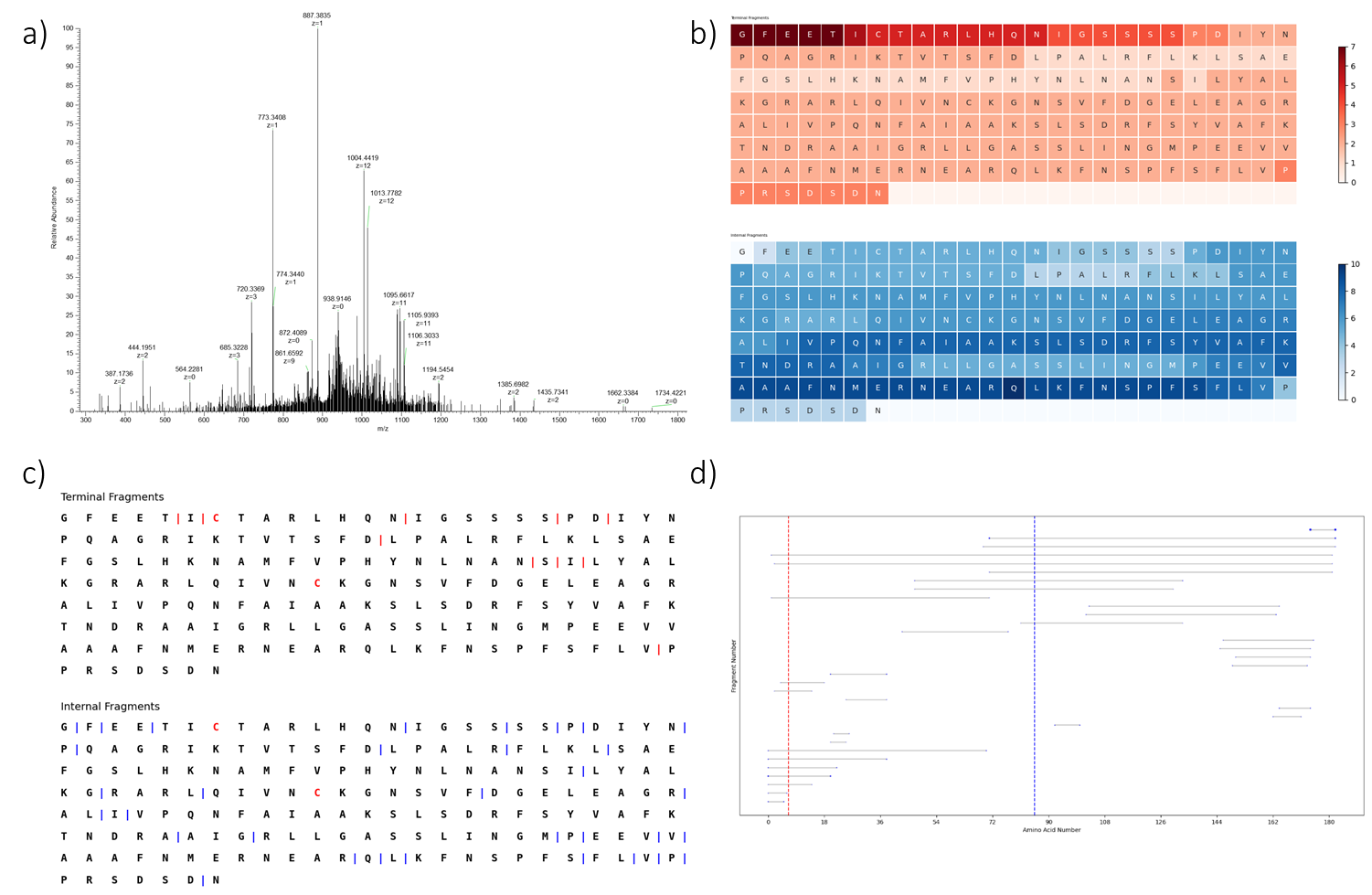


**Figure S19.** a) HCD mass spectrum of the ion at m/z 911.4741 ([M+22H]^22+^); b) sequence coverage map for the terminal and internal fragments. Darker regions indicate more coverage; c) fragment cleavage map indicating the location of inter-amino-acid cleavage sites for terminal and internal fragments); and d) fragment location map indicating the region of the β-chain sequence covered by terminal and internal fragments. The two lines, red and blue, indicate the cysteine residue positions.

**Table S7**. Sample output of ClipsMS related to the CID mass spectrum of the at m/z 911.4741 ([M+22H]^22+^). The Table includes the fragment type, observed mass, theoretical mass, start amino acid, end amino acid, error (ppm), sequence, intensity, and molecular formula. The amino acid positions refer to the sequence reported in Figure S18b.

| **Frag Type** | **Localized Mod** | **Observed Mass** | **Theoretical Mass** | **Start AA** | **End AA** | **Error** | **Sequence** | **Intensity** | **Formula** |
| --- | --- | --- | --- | --- | --- | --- | --- | --- | --- |
| B Fragment | 0 | 564.2282 | 564.2300 | 1 | 5 | -3.3 | GFEET | 3206.85 | C25 H34 N5 O10 S0 |
| B Fragment | 0 | 677.3123 | 677.3141 | 1 | 6 | -2.7 | GFEETI | 2012.17 | C31 H45 N6 O11 S0 |
| B Fragment | 57.0215 | 1657.7667 | 1657.7752 | 1 | 14 | -5.1 | GFEETICTARLHQN | 907.59 | C68 H106 N21 O22 S1 |
| B Fragment | 57.0215 | 2176.0026 | 2176.0088 | 1 | 20 | -2.9 | GFEETICTARLHQNIGSSSS | 15058.22 | C88 H140 N27 O32 S1 |
| B Fragment | 57.0215 | 2388.0835 | 2388.0885 | 1 | 22 | -2.1 | GFEETICTARLHQNIGSSSSPD | 3987.13 | C97 H152 N29 O36 S1 |
| B Fragment | 57.0215 | 4179.0132 | 4179.0200 | 1 | 38 | -1.6 | GFEETICTARLHQNIGSSSSPDIYNPQAGRIKTVTSFD | 3720.14 | C178 H278 N51 O60 S1 |
| B Fragment | 57.0215 | 7759.8875 | 7759.8966 | 1 | 70 | -1.2 | GFEETICTARLHQNIGSSSSPDIYNPQAGRIKTVTSFDLPALRFLKLSAEFGSLHKNAMFVPHYNLNANS | 2620.35 | C344 H531 N96 O102 S2 |
| BY Int Fragment | 0 | 603.2754 | 603.2773 | 21 | 25 | -3.2 | PDIYN | 674.94 | C28 H39 N6 O9 S0 |
| BY Int Fragment | 0 | 603.2754 | 603.2773 | 22 | 26 | -3.2 | DIYNP | 674.94 | C28 H39 N6 O9 S0 |
| BY Int Fragment | 0 | 828.3835 | 828.3846 | 93 | 100 | -1.4 | DGELEAGR | 4338.22 | C33 H54 N11 O14 S0 |
| BY Int Fragment | 0 | 1049.5470 | 1049.5415 | 163 | 171 | 5.3 | QLKFNSPFS | 2033.68 | C50 H73 N12 O13 S0 |
| BY Int Fragment | 0 | 1167.6162 | 1167.6197 | 165 | 174 | -3.0 | KFNSPFSFLV | 2847.92 | C59 H83 N12 O13 S0 |
| BY Int Fragment | 0 | 1401.7456 | 1401.7485 | 26 | 38 | -2.1 | PQAGRIKTVTSFD | 1060.71 | C62 H101 N18 O19 S0 |
| BY Int Fragment | 57.0215 | 1453.6837 | 1453.6853 | 3 | 14 | -1.1 | EETICTARLHQN | 438.03 | C57 H94 N19 O20 S1 |
| BY Int Fragment | 57.0215 | 1539.7724 | 1539.7697 | 5 | 18 | 1.8 | TICTARLHQNIGSS | 644.72 | C61 H104 N21 O20 S1 |
| BY Int Fragment | 0 | 2004.0158 | 2004.0185 | 21 | 38 | -1.4 | PDIYNPQAGRIKTVTSFD | 5091.79 | C90 H139 N24 O28 S0 |
| BY Int Fragment | 0 | 2769.3824 | 2769.3930 | 150 | 173 | -3.8 | VAAAFNMERNEARQLKFNSPFSFL | 1488.73 | C126 H190 N35 O34 S1 |
| BY Int Fragment | 0 | 2769.3824 | 2769.3930 | 151 | 174 | -3.8 | AAAFNMERNEARQLKFNSPFSFLV | 1488.73 | C126 H190 N35 O34 S1 |
| BY Int Fragment | 0 | 3322.6647 | 3322.6678 | 146 | 174 | -0.9 | PEEVVAAAFNMERNEARQLKFNSPFSFLV | 1009.25 | C151 H229 N40 O43 S1 |
| BY Int Fragment | 0 | 3322.6647 | 3322.6678 | 147 | 175 | -0.9 | EEVVAAAFNMERNEARQLKFNSPFSFLVP | 1009.25 | C151 H229 N40 O43 S1 |
| BY Int Fragment | 0 | 3789.9601 | 3789.9938 | 44 | 77 | -8.9 | FLKLSAEFGSLHKNAMFVPHYNLNANSILYALKG | 1883.04 | C178 H270 N45 O45 S1 |
| BY Int Fragment | 31.9898 | 5585.8869 | 5585.8598 | 82 | 133 | 4.8 | QIVNCKGNSVFDGELEAGRALIVPQNFAIAAKSLSDRFSYVAFKTNDRAAIG | 321.84 | C247 H391 N70 O74 S1 |
| BY Int Fragment | 0 | 6607.3435 | 6607.4089 | 103 | 163 | -9.9 | IVPQNFAIAAKSLSDRFSYVAFKTNDRAAIGRLLGASSLINGMPEEVVAAAFNMERNEARQ | 2053.21 | C291 H466 N85 O87 S2 |
| BY Int Fragment | 0 | 6607.3435 | 6607.4089 | 104 | 164 | -9.9 | VPQNFAIAAKSLSDRFSYVAFKTNDRAAIGRLLGASSLINGMPEEVVAAAFNMERNEARQL | 2053.21 | C291 H466 N85 O87 S2 |
| BY Int Fragment | 57.0215 | 7815.9079 | 7815.9592 | 2 | 71 | -6.6 | FEETICTARLHQNIGSSSSPDIYNPQAGRIKTVTSFDLPALRFLKLSAEFGSLHKNAMFVPHYNLNANSI | 315 | C348 H539 N96 O102 S2 |
| BY Int Fragment | 31.9898 | 9128.7191 | 9128.6955 | 48 | 130 | 2.6 | SAEFGSLHKNAMFVPHYNLNANSILYALKGRARLQIVNCKGNSVFDGELEAGRALIVPQNFAIAAKSLSDRFSYVAFKTNDRA | 507.92 | C408 H638 N117 O116 S2 |
| BY Int Fragment | 31.9898 | 9369.8040 | 9369.8382 | 48 | 133 | -3.7 | SAEFGSLHKNAMFVPHYNLNANSILYALKGRARLQIVNCKGNSVFDGELEAGRALIVPQNFAIAAKSLSDRFSYVAFKTNDRAAIG | 1582.74 | C419 H657 N120 O119 S2 |
| BY Int Fragment | 31.9898 | 12016.1185 | 12016.2181 | 72 | 181 | -8.3 | LYALKGRARLQIVNCKGNSVFDGELEAGRALIVPQNFAIAAKSLSDRFSYVAFKTNDRAAIGRLLGASSLINGMPEEVVAAAFNMERNEARQLKFNSPFSFLVPPRSDSD | 1039.68 | C533 H848 N153 O156 S3 |
| BY Int Fragment | 89.0113 | 19684.1235 | 19684.1016 | 3 | 181 | 1.1 | EETICTARLHQNIGSSSSPDIYNPQAGRIKTVTSFDLPALRFLKLSAEFGSLHKNAMFVPHYNLNANSILYALKGRARLQIVNCKGNSVFDGELEAGRALIVPQNFAIAAKSLSDRFSYVAFKTNDRAAIGRLLGASSLINGMPEEVVAAAFNMERNEARQLKFNSPFSFLVPPRSDSD | 1271.84 | C872 H1377 N248 O257 S5 |
| BY Int Fragment | 89.0113 | 19831.0994 | 19831.1701 | 2 | 181 | -3.6 | FEETICTARLHQNIGSSSSPDIYNPQAGRIKTVTSFDLPALRFLKLSAEFGSLHKNAMFVPHYNLNANSILYALKGRARLQIVNCKGNSVFDGELEAGRALIVPQNFAIAAKSLSDRFSYVAFKTNDRAAIGRLLGASSLINGMPEEVVAAAFNMERNEARQLKFNSPFSFLVPPRSDSD | 857.25 | C881 H1386 N249 O258 S5 |
| Y Fragment | 0 | 887.3835 | 887.3854 | 175 | 182 | -2.1 | PPRSDSDN | 50400.5 | C34 H55 N12 O16 S0 |
| Y Fragment | 31.9898 | 12148.2133 | 12148.2716 | 72 | 182 | -4.8 | LYALKGRARLQIVNCKGNSVFDGELEAGRALIVPQNFAIAAKSLSDRFSYVAFKTNDRAAIGRLLGASSLINGMPEEVVAAAFNMERNEARQLKFNSPFSFLVPPRSDSDN | 14566.95 | C537 H856 N155 O159 S3 |
| Y Fragment | 31.9898 | 12348.3458 | 12348.3877 | 70 | 182 | -3.4 | SILYALKGRARLQIVNCKGNSVFDGELEAGRALIVPQNFAIAAKSLSDRFSYVAFKTNDRAAIGRLLGASSLINGMPEEVVAAAFNMERNEARQLKFNSPFSFLVPPRSDSDN | 1046.94 | C546 H872 N157 O162 S3 |
| B Fragment* | 57.0215 | 1983.9284 | 1983.9341 | 1 | 18 | -2.9 | GFEETICTARLHQNIGSS | 3318.04 | C82 H130 N25 O28 S1 |
| B Fragment* | 57.0215 | 2070.9634 | 2070.9662 | 1 | 19 | -1.3 | GFEETICTARLHQNIGSSS | 6911.76 | C85 H135 N26 O30 S1 |
| B Fragment* | 57.0215 | 2157.9925 | 2157.9982 | 1 | 20 | -2.7 | GFEETICTARLHQNIGSSSS | 14843.36 | C88 H140 N27 O32 S1 |
| B Fragment* | 57.0215 | 7741.8748 | 7741.8860 | 1 | 70 | -1.4 | GFEETICTARLHQNIGSSSSPDIYNPQAGRIKTVTSFDLPALRFLKLSAEFGSLHKNAMFVPHYNLNANS | 2798.3 | C344 H531 N96 O102 S2 |
| Y Fragment* | 0 | 8446.2643 | 8446.2677 | 106 | 182 | -0.4 | QNFAIAAKSLSDRFSYVAFKTNDRAAIGRLLGASSLINGMPEEVVAAAFNMERNEARQLKFNSPFSFLVPPRSDSDN | 1792.9 | C374 H586 N107 O114 S2 |
| Y Fragment** | 31.9898 | 17731.1700 | 17731.1641 | 22 | 182 | 0.3 | DIYNPQAGRIKTVTSFDLPALRFLKLSAEFGSLHKNAMFVPHYNLNANSILYALKGRARLQIVNCKGNSVFDGELEAGRALIVPQNFAIAAKSLSDRFSYVAFKTNDRAAIGRLLGASSLINGMPEEVVAAAFNMERNEARQLKFNSPFSFLVPPRSDSDN | 1778.46 | C794 H1251 N224 O229 S4 |
| BY Int Fragment* | 0 | 706.3715 | 706.3671 | 61 | 66 | 6.2 | VPHYNL | 827.05 | C35 H50 N9 O8 S0 |
| BY Int Fragment* | 0 | 706.3715 | 706.3671 | 118 | 123 | 6.2 | RFSYVA | 827.05 | C35 H50 N9 O8 S0 |
| BY Int Fragment** | 0 | 773.3407 | 773.3352 | 91 | 97 | 7.1 | VFDGELE | 36068.28 | C36 H52 N7 O13 S0 |
| BY Int Fragment* | 0 | 828.3835 | 828.3861 | 58 | 64 | -3.2 | AMFVPHY | 4338.22 | C42 H56 N9 O8 S1 |
| BY Int Fragment* | 31.9898 | 841.4297 | 841.4348 | 80 | 86 | -6.1 | RLQIVNC | 2491.71 | C35 H63 N12 O9 S1 |
| BY Int Fragment* | 0 | 935.5390 | 935.5309 | 95 | 103 | 8.7 | ELEAGRALI | 3188.58 | C42 H73 N12 O13 S0 |
| BY Int Fragment* | 0 | 1020.5351 | 1020.5447 | 54 | 62 | -9.5 | LHKNAMFVP | 2060.37 | C49 H76 N13 O10 S1 |
| BY Int Fragment* | 57.0215 | 1435.6716 | 1435.6747 | 3 | 14 | -2.2 | EETICTARLHQN | 431.33 | C57 H94 N19 O20 S1 |
| BY Int Fragment** | 0 | 1443.6387 | 1443.6386 | 13 | 26 | 0.0 | QNIGSSSSPDIYNP | 272.94 | C62 H94 N17 O24 S0 |
| BY Int Fragment** | 0 | 1443.6387 | 1443.6386 | 14 | 27 | 0.0 | NIGSSSSPDIYNPQ | 272.94 | C62 H94 N17 O24 S0 |
| BY Int Fragment** | 0 | 1539.7724 | 1539.7702 | 118 | 130 | 1.4 | RFSYVAFKTNDRA | 644.72 | C71 H106 N21 O19 S0 |
| BY Int Fragment** | 0 | 1546.7829 | 1546.7860 | 93 | 107 | -2.0 | DGELEAGRALIVPQN | 497.13 | C67 H111 N20 O23 S0 |
| BY Int Fragment** | 0 | 1661.7794 | 1661.7958 | 166 | 180 | -9.9 | FNSPFSFLVPPRSDS | 462.71 | C79 H112 N19 O22 S0 |
| BY Int Fragment* | 0 | 1910.9825 | 1910.9759 | 92 | 109 | 3.4 | FDGELEAGRALIVPQNFA | 925.04 | C88 H134 N23 O26 S0 |
| BY Int Fragment** | 0 | 1913.9863 | 1914.0054 | 43 | 59 | -10.0 | RFLKLSAEFGSLHKNAM | 1691.85 | C88 H140 N25 O22 S1 |
| BY Int Fragment* | 57.0215 | 1953.9047 | 1953.9083 | 3 | 20 | -1.9 | EETICTARLHQNIGSSSS | 5722.52 | C77 H128 N25 O30 S1 |
| BY Int Fragment** | 0 | 1980.0306 | 1980.0450 | 62 | 79 | -7.3 | PHYNLNANSILYALKGRA | 2112.53 | C91 H142 N27 O24 S0 |
| BY Int Fragment** | 0 | 2769.3824 | 2769.3590 | 12 | 37 | 8.4 | HQNIGSSSSPDIYNPQAGRIKTVTSF | 1488.73 | C121 H189 N36 O40 S0 |
| BY Int Fragment** | 0 | 3323.6543 | 3323.6875 | 135 | 165 | -10.0 | LLGASSLINGMPEEVVAAAFNMERNEARQLK | 1815.26 | C144 H239 N42 O45 S2 |
| BY Int Fragment* | 0 | 3789.9601 | 3789.9962 | 18 | 52 | -9.5 | SSSPDIYNPQAGRIKTVTSFDLPALRFLKLSAEFG | 1883.04 | C174 H272 N45 O51 S0 |
| BY Int Fragment* | 0 | 3789.9601 | 3789.9962 | 19 | 53 | -9.5 | SSPDIYNPQAGRIKTVTSFDLPALRFLKLSAEFGS | 1883.04 | C174 H272 N45 O51 S0 |
| BY Int Fragment* | 0 | 5585.8869 | 5585.8791 | 14 | 64 | 1.4 | NIGSSSSPDIYNPQAGRIKTVTSFDLPALRFLKLSAEFGSLHKNAMFVPHY | 321.84 | C256 H393 N68 O72 S1 |
| BY Int Fragment* | 0 | 5585.8869 | 5585.8791 | 15 | 65 | 1.4 | IGSSSSPDIYNPQAGRIKTVTSFDLPALRFLKLSAEFGSLHKNAMFVPHYN | 321.84 | C256 H393 N68 O72 S1 |
| BY Int Fragment* | 0 | 5585.8869 | 5585.8791 | 16 | 66 | 1.4 | GSSSSPDIYNPQAGRIKTVTSFDLPALRFLKLSAEFGSLHKNAMFVPHYNL | 321.84 | C256 H393 N68 O72 S1 |
| BY Int Fragment* | 0 | 6588.3630 | 6588.3925 | 14 | 73 | -4.5 | NIGSSSSPDIYNPQAGRIKTVTSFDLPALRFLKLSAEFGSLHKNAMFVPHYNLNANSILY | 1962.77 | C301 H463 N80 O86 S1 |
| BY Int Fragment** | 31.9898 | 7405.7461 | 7405.8100 | 82 | 151 | -8.6 | QIVNCKGNSVFDGELEAGRALIVPQNFAIAAKSLSDRFSYVAFKTNDRAAIGRLLGASSLINGMPEEVVA | 272.46 | C327 H527 N92 O99 S2 |
| BY Int Fragment* | 0 | 7554.7906 | 7554.8590 | 112 | 180 | -9.1 | AKSLSDRFSYVAFKTNDRAAIGRLLGASSLINGMPEEVVAAAFNMERNEARQLKFNSPFSFLVPPRSDS | 549.36 | C336 H529 N96 O100 S2 |
| BY Int Fragment** | 0 | 7961.0423 | 7961.1018 | 91 | 164 | -7.5 | VFDGELEAGRALIVPQNFAIAAKSLSDRFSYVAFKTNDRAAIGRLLGASSLINGMPEEVVAAAFNMERNEARQL | 1381.81 | C353 H564 N101 O106 S2 |
| BY Int Fragment** | 31.9898 | 11260.8723 | 11260.8619 | 46 | 149 | 0.9 | KLSAEFGSLHKNAMFVPHYNLNANSILYALKGRARLQIVNCKGNSVFDGELEAGRALIVPQNFAIAAKSLSDRFSYVAFKTNDRAAIGRLLGASSLINGMPEEV | 1217.36 | C503 H802 N143 O144 S3 |
| BY Int Fragment* | 31.9898 | 12034.1718 | 12034.2439 | 64 | 173 | -6.0 | YNLNANSILYALKGRARLQIVNCKGNSVFDGELEAGRALIVPQNFAIAAKSLSDRFSYVAFKTNDRAAIGRLLGASSLINGMPEEVVAAAFNMERNEARQLKFNSPFSFL | 16898.67 | C537 H852 N153 O155 S3 |
| BY Int Fragment* | 89.0113 | 12050.2067 | 12050.2389 | 3 | 112 | -2.7 | EETICTARLHQNIGSSSSPDIYNPQAGRIKTVTSFDLPALRFLKLSAEFGSLHKNAMFVPHYNLNANSILYALKGRARLQIVNCKGNSVFDGELEAGRALIVPQNFAIAA | 1589.23 | C535 H849 N152 O155 S3 |
| BY Int Fragment* | 31.9898 | 12235.3404 | 12235.3011 | 47 | 159 | 3.2 | LSAEFGSLHKNAMFVPHYNLNANSILYALKGRARLQIVNCKGNSVFDGELEAGRALIVPQNFAIAAKSLSDRFSYVAFKTNDRAAIGRLLGASSLINGMPEEVVAAAFNMERN | 934.67 | C544 H863 N156 O157 S4 |

Following the MS/MS of the triply-charged molecular ion at m/z 733.6852 and the doubly-charged molecular ion at m/z 453.7389 detected in the shotgun approach of the enriched fraction of legumins. The database search, carried out by PEAKS, identified these ions with the tryptic peptides Leu^395^-Arg^414^ (Fig. S20a) and Leu^395^-Gly^402^ (Fig.S20b), respectively, of the legumin with Acc. No. A0A3Q7XNW1. Particularly, the first peptide carries the cysteine residue at position 400 as sulfonic acid, whereas the second one shows this cysteine as sulfinic acid.


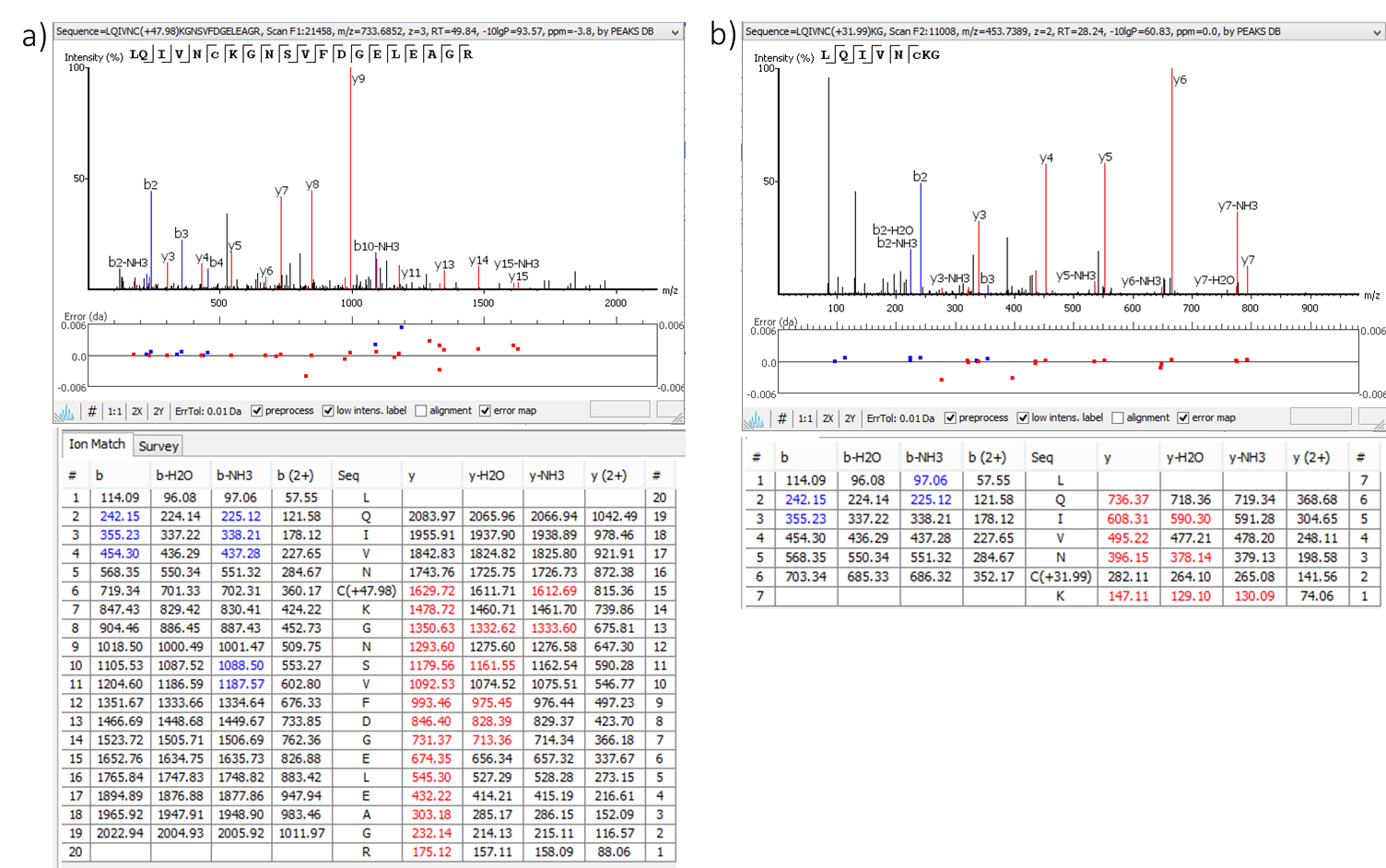


**Figure S20.** PEAKS report of the MS/MS of the ions at m/z 733.6852 (a) and 453.7389 (b).

**Characterization of the Vicilin-enriched Fraction**

The complete list of the proteins and the supporting peptides identified in the vicilin-enriched fraction are reported in Supplementary Table S8.

***Polypeptides related to the legumin A0A1S2XQR4***

Figures S21 reports the multi-charged ESI MS and the corresponding deconvoluted mass spectra of two polypeptides with experimental monoisotopic molecular masses (m_mono_) of 13750.0547 and 13996.1054 Da. These components are related to the amino acidic region Arg^209^-Asn^329^ of the vicilin-like protein with the Acc. No. tr|A0A1S2XQR4, and to the region Arg^204^-Asn^327^ of the unreviewed entry of a chickpea vicilin deposited in UniProt with the Acc. No. Q304D4 (see Figure 11 of the manuscript), respectively.


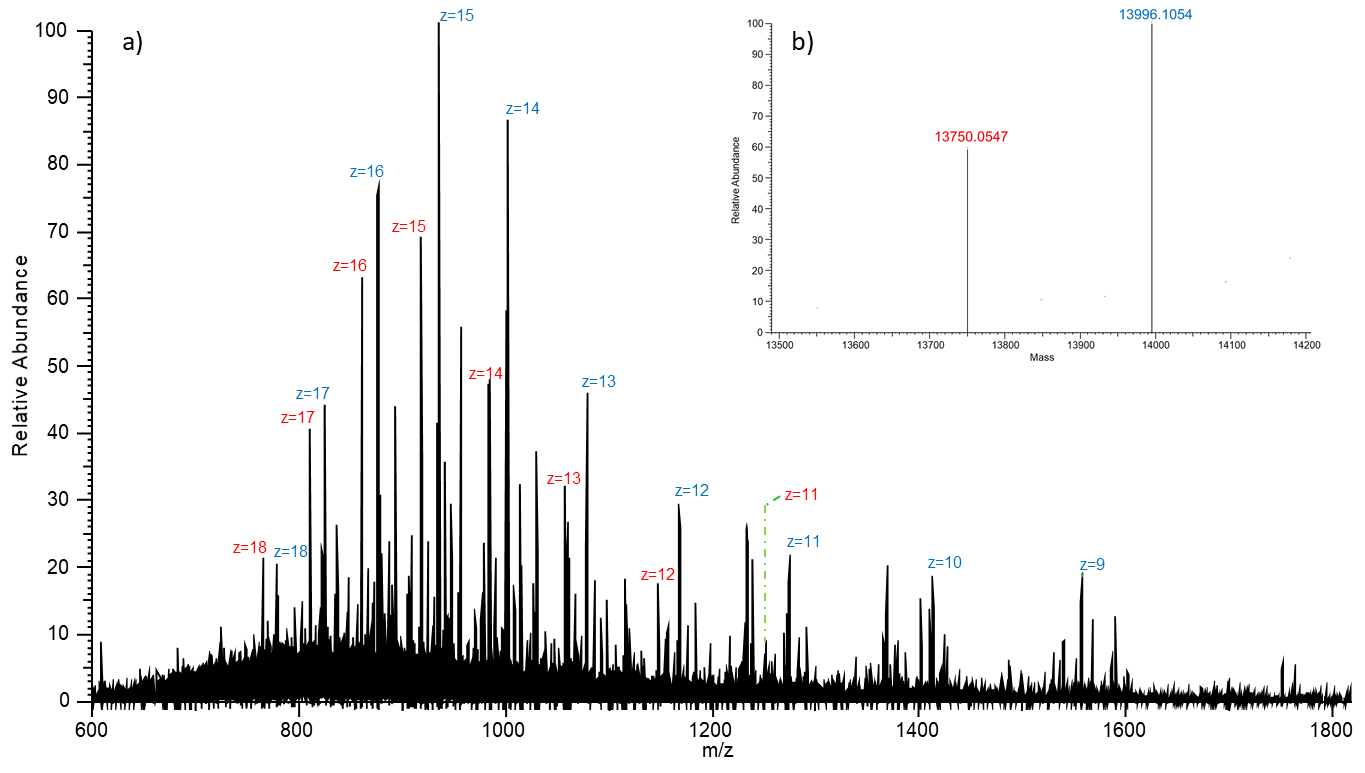


**Figure S21**. a) Multi-charged mass spectrum and b) monoisotopic deconvoluted mass spectrum (mass zero-charge) of the two coeluting polypeptides with m_mono_ 13750.0547 and 13996.1054 Da.

Top-down characterization of the component with the experimental mass 13750.0547 Da was achieved by the MS/MS of its multi-charged ion [M+12H]^12+^ at m/z 1147.4307, interpreted by the ClipsMS tool using the criteria above reported for internal fragments. The deconvoluted MS/MS of this ion is reported in Figure S22a, and the corresponding sequence in Figure S22b.


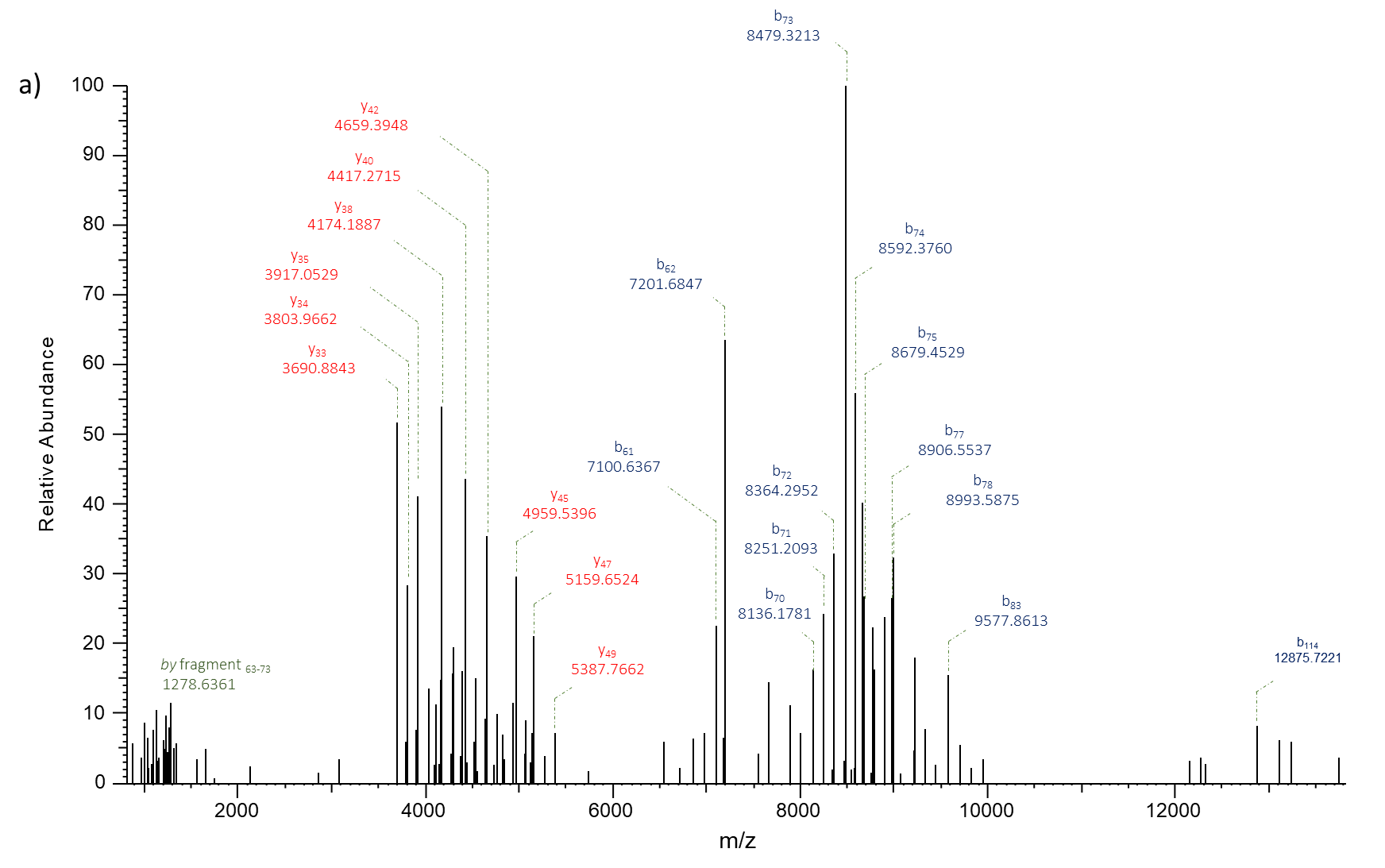


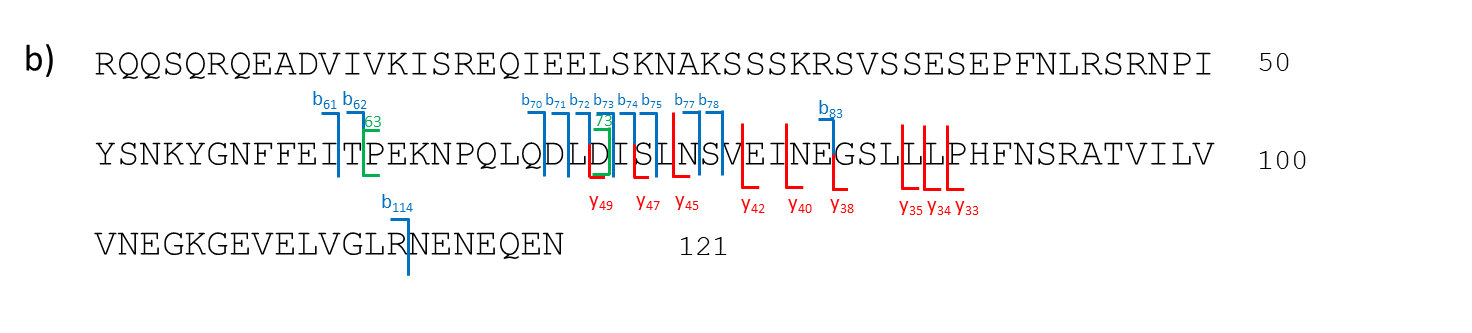


**Figure S22.** a) Deconvoluted MS/MS spectrum of the ion at m/z 1147.4307 which corresponds to the multi-charged ion [M+12H]^12+^ of the polypeptide with m_mono_ 13750.0547 Da. The most abundant y- and b-ions are marked in red and blue, respectively. The most intense internal fragment ions are shown in green; b) Sequence coverage map as obtained by the MS/MS shown in the panel a. This polypeptide corresponds to the region Arg209-Asn329 (here renumbered 1-121) of the vicilin-like protein entry with Acc. No. A0A1S2XQR4. b- and y-fragments are reported in blue and red, respectively. The internal fragments are reported in green.

Following the complete report of the ClipsMS interpretation (Fig. S23, Table S9) of the multi-charged ion [M+12H]^12+^ at m/z 1147.4307 of the polypeptide with m_mono_ 13750.0547 Da, corresponding with the amino acidic region Arg^209^-Asn^329^ of the vicilin-like protein with the Acc. No. tr|A0A1S2XQR4.


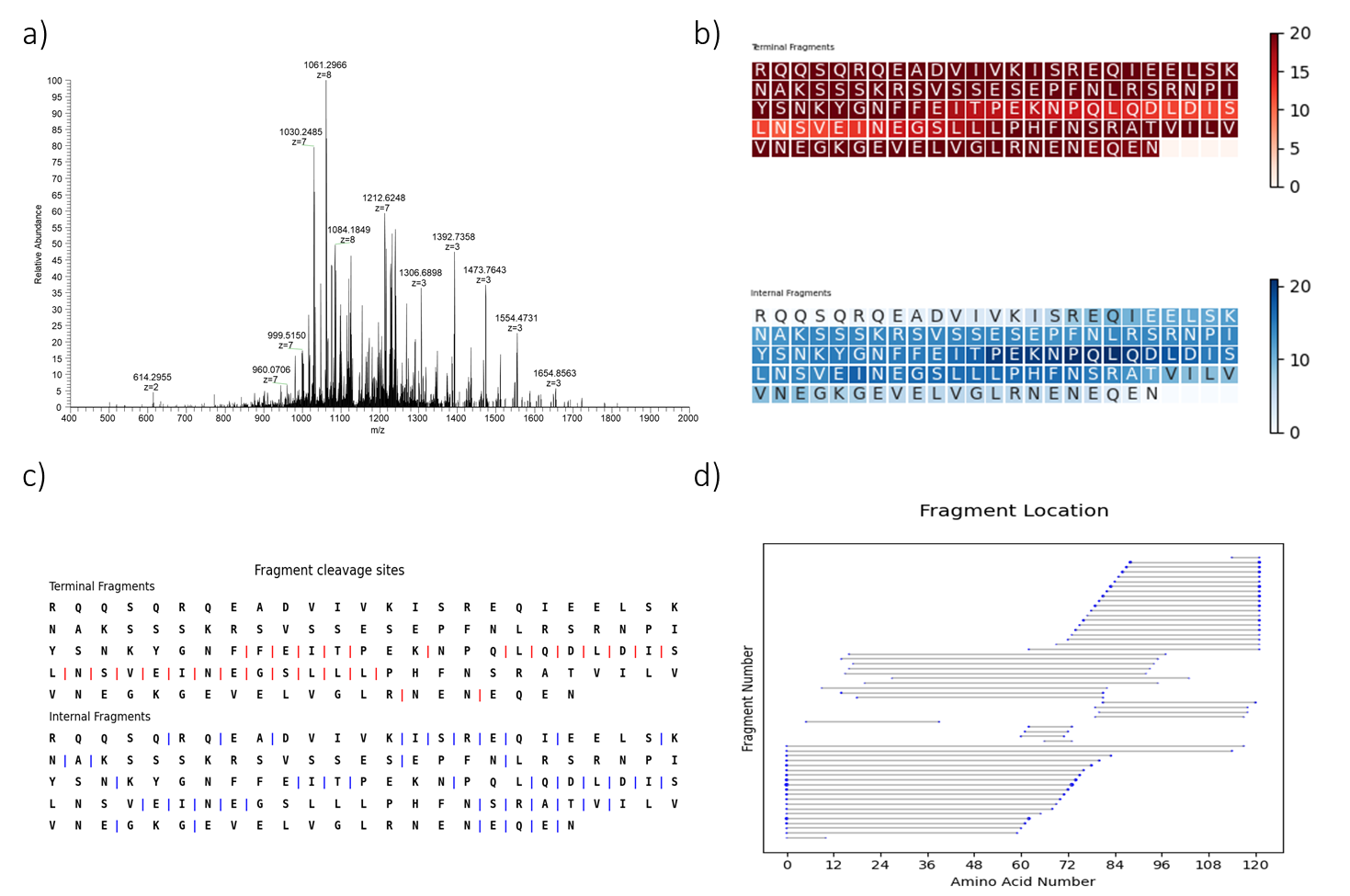


**Figure S23.** a) HCD mass spectrum of the ion at m/z 1147.4307 ([M+12H]^12+^); b) sequence coverage map for the terminal and internal fragments. Darker regions indicate more coverage; c) fragment cleavage map indicating the location of inter-amino-acid cleavage sites for terminal and internal fragments); and d) fragment location map indicating the region of the β-chain sequence covered by terminal and internal fragments.

**Table S9**. Sample output of ClipsMS related to the CID mass spectrum of the ion at m/z 1147.4307 ([M+12H]^12+^). The Table includes the fragment type, observed mass, theoretical mass, start amino acid, end amino acid, error (ppm), sequence, intensity, and molecular formula. The amino acid positions refer to the sequence reported in Figure 22b.

| **Frag Type** | **Observed Mass** | **Theoretical Mass** | **Start AA** | **End AA** | **Error (ppm)** | **Sequence** | **Intensity** | **Formula** |
| --- | --- | --- | --- | --- | --- | --- | --- | --- |
| B Fragment | 6711,454 | 6711,437 | 1 | 58 | 2,5 | RQQSQRQEADVIVKISREQIEELSKNAKSSSKRSVSSESEPFNLRSRNPIYSNKYGNF | 21667,96 | C286 H466 N91 O96 S0 |
| B Fragment | 6858,53 | 6858,506 | 1 | 59 | 3,5 | RQQSQRQEADVIVKISREQIEELSKNAKSSSKRSVSSESEPFNLRSRNPIYSNKYGNFF | 45958,59 | C295 H475 N92 O97 S0 |
| B Fragment | 6987,521 | 6987,548 | 1 | 60 | -3,9 | RQQSQRQEADVIVKISREQIEELSKNAKSSSKRSVSSESEPFNLRSRNPIYSNKYGNFFE | 52039,36 | C300 H482 N93 O100 S0 |
| B Fragment | 7100,637 | 7100,633 | 1 | 61 | 0,6 | RQQSQRQEADVIVKISREQIEELSKNAKSSSKRSVSSESEPFNLRSRNPIYSNKYGNFFEI | 160954,4 | C306 H493 N94 O101 S0 |
| B Fragment | 7201,685 | 7201,68 | 1 | 62 | 0,6 | RQQSQRQEADVIVKISREQIEELSKNAKSSSKRSVSSESEPFNLRSRNPIYSNKYGNFFEIT | 452057,5 | C310 H500 N95 O103 S0 |
| B Fragment | 7555,863 | 7555,871 | 1 | 65 | -0,9 | RQQSQRQEADVIVKISREQIEELSKNAKSSSKRSVSSESEPFNLRSRNPIYSNKYGNFFEITPEK | 30848,81 | C326 H526 N99 O108 S0 |
| B Fragment | 7895,052 | 7895,025 | 1 | 68 | 3,5 | RQQSQRQEADVIVKISREQIEELSKNAKSSSKRSVSSESEPFNLRSRNPIYSNKYGNFFEITPEKNPQ | 82465,3 | C340 H547 N104 O113 S0 |
| B Fragment | 8008,094 | 8008,109 | 1 | 69 | -1,9 | RQQSQRQEADVIVKISREQIEELSKNAKSSSKRSVSSESEPFNLRSRNPIYSNKYGNFFEITPEKNPQL | 52552,81 | C346 H558 N105 O114 S0 |
| B Fragment | 8136,178 | 8136,167 | 1 | 70 | 1,3 | RQQSQRQEADVIVKISREQIEELSKNAKSSSKRSVSSESEPFNLRSRNPIYSNKYGNFFEITPEKNPQLQ | 118382,5 | C351 H566 N107 O116 S0 |
| B Fragment | 8251,213 | 8251,194 | 1 | 71 | 2,3 | RQQSQRQEADVIVKISREQIEELSKNAKSSSKRSVSSESEPFNLRSRNPIYSNKYGNFFEITPEKNPQLQD | 173654 | C355 H571 N108 O119 S0 |
| B Fragment | 8364,295 | 8364,278 | 1 | 72 | 2,0 | RQQSQRQEADVIVKISREQIEELSKNAKSSSKRSVSSESEPFNLRSRNPIYSNKYGNFFEITPEKNPQLQDL | 234510 | C361 H582 N109 O120 S0 |
| B Fragment | 8479,308 | 8479,305 | 1 | 73 | 0,3 | RQQSQRQEADVIVKISREQIEELSKNAKSSSKRSVSSESEPFNLRSRNPIYSNKYGNFFEITPEKNPQLQDLD | 710661 | C365 H587 N110 O123 S0 |
| B Fragment | 8592,377 | 8592,389 | 1 | 74 | -1,5 | RQQSQRQEADVIVKISREQIEELSKNAKSSSKRSVSSESEPFNLRSRNPIYSNKYGNFFEITPEKNPQLQDLDI | 404962,7 | C371 H598 N111 O124 S0 |
| B Fragment | 8679,453 | 8679,422 | 1 | 75 | 3,6 | RQQSQRQEADVIVKISREQIEELSKNAKSSSKRSVSSESEPFNLRSRNPIYSNKYGNFFEITPEKNPQLQDLDIS | 191663,3 | C374 H603 N112 O126 S0 |
| B Fragment | 8792,454 | 8792,506 | 1 | 76 | -5,8 | RQQSQRQEADVIVKISREQIEELSKNAKSSSKRSVSSESEPFNLRSRNPIYSNKYGNFFEITPEKNPQLQDLDISL | 119195,9 | C380 H614 N113 O127 S0 |
| B Fragment | 8993,587 | 8993,581 | 1 | 78 | 0,8 | RQQSQRQEADVIVKISREQIEELSKNAKSSSKRSVSSESEPFNLRSRNPIYSNKYGNFFEITPEKNPQLQDLDISLNS | 230254,8 | C387 H625 N116 O131 S0 |
| B Fragment | 9221,702 | 9221,692 | 1 | 80 | 1,1 | RQQSQRQEADVIVKISREQIEELSKNAKSSSKRSVSSESEPFNLRSRNPIYSNKYGNFFEITPEKNPQLQDLDISLNSVE | 130389,4 | C397 H641 N118 O135 S0 |
| B Fragment | 9577,861 | 9577,861 | 1 | 83 | 0,0 | RQQSQRQEADVIVKISREQIEELSKNAKSSSKRSVSSESEPFNLRSRNPIYSNKYGNFFEITPEKNPQLQDLDISLNSVEINE | 112288,2 | C412 H665 N122 O141 S0 |
| B Fragment | 12875,72 | 12875,72 | 1 | 114 | 0,4 | RQQSQRQEADVIVKISREQIEELSKNAKSSSKRSVSSESEPFNLRSRNPIYSNKYGNFFEITPEKNPQLQDLDISLNSVEINEGSLLLPHFNSRATVILVVNEGKGEVELVGLR | 59455,5 | C561 H913 N164 O183 S0 |
| B Fragment | 13232,87 | 13232,85 | 1 | 117 | 1,6 | RQQSQRQEADVIVKISREQIEELSKNAKSSSKRSVSSESEPFNLRSRNPIYSNKYGNFFEITPEKNPQLQDLDISLNSVEINEGSLLLPHFNSRATVILVVNEGKGEVELVGLRNEN | 43425,31 | C574 H932 N169 O190 S0 |
| Y Fragment | 876,3337 | 876,333 | 115 | 121 | 0,8 | NENEQEN | 41676,46 | C32 H50 N11 O18 S0 |
| Y Fragment | 3690,884 | 3690,883 | 89 | 121 | 0,3 | PHFNSRATVILVVNEGKGEVELVGLRNENEQEN | 367652 | C158 H257 N48 O54 S0 |
| Y Fragment | 3803,966 | 3803,968 | 88 | 121 | -0,3 | LPHFNSRATVILVVNEGKGEVELVGLRNENEQEN | 202571,3 | C164 H268 N49 O55 S0 |
| Y Fragment | 3917,053 | 3917,052 | 87 | 121 | 0,4 | LLPHFNSRATVILVVNEGKGEVELVGLRNENEQEN | 293235,6 | C170 H279 N50 O56 S0 |
| Y Fragment | 4030,141 | 4030,136 | 86 | 121 | 1,4 | LLLPHFNSRATVILVVNEGKGEVELVGLRNENEQEN | 102532,7 | C176 H290 N51 O57 S0 |
| Y Fragment | 4117,172 | 4117,168 | 85 | 121 | 1,1 | SLLLPHFNSRATVILVVNEGKGEVELVGLRNENEQEN | 84929,47 | C179 H295 N52 O59 S0 |
| Y Fragment | 4174,189 | 4174,189 | 84 | 121 | -0,1 | GSLLLPHFNSRATVILVVNEGKGEVELVGLRNENEQEN | 384662,7 | C181 H298 N53 O60 S0 |
| Y Fragment | 4303,227 | 4303,232 | 83 | 121 | -1,1 | EGSLLLPHFNSRATVILVVNEGKGEVELVGLRNENEQEN | 143204,5 | C186 H305 N54 O63 S0 |
| Y Fragment | 4417,271 | 4417,275 | 82 | 121 | -0,7 | NEGSLLLPHFNSRATVILVVNEGKGEVELVGLRNENEQEN | 314746,8 | C190 H311 N56 O65 S0 |
| Y Fragment | 4530,367 | 4530,359 | 81 | 121 | 1,7 | INEGSLLLPHFNSRATVILVVNEGKGEVELVGLRNENEQEN | 111144,6 | C196 H322 N57 O66 S0 |
| Y Fragment | 4659,395 | 4659,401 | 80 | 121 | -1,4 | EINEGSLLLPHFNSRATVILVVNEGKGEVELVGLRNENEQEN | 256685,6 | C201 H329 N58 O69 S0 |
| Y Fragment | 4758,468 | 4758,47 | 79 | 121 | -0,4 | VEINEGSLLLPHFNSRATVILVVNEGKGEVELVGLRNENEQEN | 74252,31 | C206 H338 N59 O70 S0 |
| Y Fragment | 4845,504 | 4845,502 | 78 | 121 | 0,5 | SVEINEGSLLLPHFNSRATVILVVNEGKGEVELVGLRNENEQEN | 25955,74 | C209 H343 N60 O72 S0 |
| Y Fragment | 5072,664 | 5072,629 | 76 | 121 | 6,9 | LNSVEINEGSLLLPHFNSRATVILVVNEGKGEVELVGLRNENEQEN | 65303,69 | C219 H360 N63 O75 S0 |
| Y Fragment | 5159,652 | 5159,661 | 75 | 121 | -1,6 | SLNSVEINEGSLLLPHFNSRATVILVVNEGKGEVELVGLRNENEQEN | 152854,1 | C222 H365 N64 O77 S0 |
| Y Fragment | 5272,743 | 5272,745 | 74 | 121 | -0,4 | ISLNSVEINEGSLLLPHFNSRATVILVVNEGKGEVELVGLRNENEQEN | 28423,73 | C228 H376 N65 O78 S0 |
| Y Fragment | 5387,766 | 5387,772 | 73 | 121 | -1,0 | DISLNSVEINEGSLLLPHFNSRATVILVVNEGKGEVELVGLRNENEQEN | 52452,76 | C232 H381 N66 O81 S0 |
| Y Fragment | 5743,943 | 5743,941 | 70 | 121 | 0,2 | QDLDISLNSVEINEGSLLLPHFNSRATVILVVNEGKGEVELVGLRNENEQEN | 13960,63 | C247 H405 N70 O87 S0 |
| Y Fragment | 6550,377 | 6550,37 | 63 | 121 | 1,1 | PEKNPQLQDLDISLNSVEINEGSLLLPHFNSRATVILVVNEGKGEVELVGLRNENEQEN | 42961,83 | C283 H463 N80 O98 S0 |
| BY Int Fragment | 810,3998 | 810,3992 | 67 | 73 | 0,7 | PQLQDLD | 17608,6 | C35 H56 N9 O13 S0 |
| BY Int Fragment | 857,4398 | 857,4363 | 75 | 82 | 4,0 | SLNSVEIN | 6604,55 | C36 H61 N10 O14 S0 |
| BY Int Fragment | 935,495 | 935,4945 | 63 | 70 | 0,5 | PEKNPQLQ | 12947,88 | C41 H67 N12 O13 S0 |
| BY Int Fragment | 1264,658 | 1264,653 | 61 | 71 | 3,7 | ITPEKNPQLQD | 59051,98 | C55 H90 N15 O19 S0 |
| BY Int Fragment | 1264,658 | 1264,653 | 62 | 72 | 3,7 | TPEKNPQLQDL | 59051,98 | C55 H90 N15 O19 S0 |
| BY Int Fragment | 1278,636 | 1278,632 | 63 | 73 | 2,8 | PEKNPQLQDLD | 83652,34 | C55 H88 N15 O20 S0 |
| BY Int Fragment | 1478,753 | 1478,749 | 63 | 75 | 3,2 | PEKNPQLQDLDIS | 18001,89 | C64 H104 N17 O23 S0 |
| BY Int Fragment | 2491,274 | 2491,25 | 54 | 74 | 9,5 | KYGNFFEITPEKNPQLQDLDI | 23474,84 | C115 H172 N27 O35 S0 |
| BY Int Fragment | 3107,62 | 3107,606 | 44 | 69 | 4,5 | LRSRNPIYSNKYGNFFEITPEKNPQL | 9273,65 | C142 H216 N39 O40 S0 |
| BY Int Fragment | 3786,989 | 3786,994 | 6 | 39 | -1,5 | RQEADVIVKISREQIEELSKNAKSSSKRSVSSES | 44119,35 | C157 H273 N50 O58 S0 |
| BY Int Fragment | 4042,137 | 4042,136 | 83 | 119 | 0,3 | EGSLLLPHFNSRATVILVVNEGKGEVELVGLRNENEQ | 34566,42 | C177 H290 N51 O57 S0 |
| BY Int Fragment | 4042,137 | 4042,136 | 84 | 120 | 0,3 | GSLLLPHFNSRATVILVVNEGKGEVELVGLRNENEQE | 34566,42 | C177 H290 N51 O57 S0 |
| BY Int Fragment | 4141,19 | 4141,204 | 80 | 117 | -3,4 | EINEGSLLLPHFNSRATVILVVNEGKGEVELVGLRNEN | 21132,93 | C182 H299 N52 O58 S0 |
| BY Int Fragment | 4141,19 | 4141,204 | 81 | 118 | -3,4 | INEGSLLLPHFNSRATVILVVNEGKGEVELVGLRNENE | 21132,93 | C182 H299 N52 O58 S0 |
| BY Int Fragment | 4270,243 | 4270,247 | 80 | 118 | -0,9 | EINEGSLLLPHFNSRATVILVVNEGKGEVELVGLRNENE | 37570,06 | C187 H306 N53 O61 S0 |
| BY Int Fragment | 4285,231 | 4285,221 | 82 | 120 | 2,4 | NEGSLLLPHFNSRATVILVVNEGKGEVELVGLRNENEQE | 112859,7 | C186 H303 N54 O62 S0 |
| BY Int Fragment | 7183,67 | 7183,625 | 19 | 81 | 6,3 | QIEELSKNAKSSSKRSVSSESEPFNLRSRNPIYSNKYGNFFEITPEKNPQLQDLDISLNSVEI | 47691,46 | C314 H498 N87 O106 S0 |
| BY Int Fragment | 7668,915 | 7668,885 | 15 | 81 | 4,0 | ISREQIEELSKNAKSSSKRSVSSESEPFNLRSRNPIYSNKYGNFFEITPEKNPQLQDLDISLNSVEI | 104493,6 | C334 H533 N94 O113 S0 |
| BY Int Fragment | 8337,271 | 8337,27 | 10 | 82 | 0,1 | DVIVKISREQIEELSKNAKSSSKRSVSSESEPFNLRSRNPIYSNKYGNFFEITPEKNPQLQDLDISLNSVEIN | 15535,99 | C364 H585 N102 O122 S0 |
| BY Int Fragment | 8478,312 | 8478,267 | 21 | 95 | 5,4 | EELSKNAKSSSKRSVSSESEPFNLRSRNPIYSNKYGNFFEITPEKNPQLQDLDISLNSVEINEGSLLLPHFNSRA | 13015,27 | C371 H584 N105 O123 S0 |
| BY Int Fragment | 8574,39 | 8574,397 | 28 | 103 | -0,8 | KSSSKRSVSSESEPFNLRSRNPIYSNKYGNFFEITPEKNPQLQDLDISLNSVEINEGSLLLPHFNSRATVILVVNE | 17091,35 | C379 H600 N105 O122 S0 |
| BY Int Fragment | 8777,464 | 8777,415 | 16 | 92 | 5,6 | SREQIEELSKNAKSSSKRSVSSESEPFNLRSRNPIYSNKYGNFFEITPEKNPQLQDLDISLNSVEINEGSLLLPHFN | 15317,77 | C384 H605 N108 O128 S0 |
| BY Int Fragment | 8777,464 | 8777,415 | 17 | 93 | 5,6 | REQIEELSKNAKSSSKRSVSSESEPFNLRSRNPIYSNKYGNFFEITPEKNPQLQDLDISLNSVEINEGSLLLPHFNS | 15317,77 | C384 H605 N108 O128 S0 |
| BY Int Fragment | 8777,464 | 8777,415 | 18 | 94 | 5,6 | EQIEELSKNAKSSSKRSVSSESEPFNLRSRNPIYSNKYGNFFEITPEKNPQLQDLDISLNSVEINEGSLLLPHFNSR | 15317,77 | C384 H605 N108 O128 S0 |
| BY Int Fragment | 8887,531 | 8887,572 | 25 | 103 | -4,6 | KNAKSSSKRSVSSESEPFNLRSRNPIYSNKYGNFFEITPEKNPQLQDLDISLNSVEINEGSLLLPHFNSRATVILVVNE | 14335,17 | C392 H623 N110 O126 S0 |
| BY Int Fragment | 8887,531 | 8887,572 | 27 | 106 | -4,6 | AKSSSKRSVSSESEPFNLRSRNPIYSNKYGNFFEITPEKNPQLQDLDISLNSVEINEGSLLLPHFNSRATVILVVNEGKG | 14335,17 | C392 H623 N110 O126 S0 |
| BY Int Fragment | 9204,679 | 9204,669 | 15 | 95 | 1,1 | ISREQIEELSKNAKSSSKRSVSSESEPFNLRSRNPIYSNKYGNFFEITPEKNPQLQDLDISLNSVEINEGSLLLPHFNSRA | 34084,87 | C402 H638 N115 O133 S0 |
| BY Int Fragment | 9204,679 | 9204,669 | 17 | 97 | 1,1 | REQIEELSKNAKSSSKRSVSSESEPFNLRSRNPIYSNKYGNFFEITPEKNPQLQDLDISLNSVEINEGSLLLPHFNSRATV | 34084,87 | C402 H638 N115 O133 S0 |
| BY Int Fragment | 10060,18 | 10060,14 | 8 | 96 | 4,1 | EADVIVKISREQIEELSKNAKSSSKRSVSSESEPFNLRSRNPIYSNKYGNFFEITPEKNPQLQDLDISLNSVEINEGSLLLPHFNSRAT | 16706,12 | C440 H703 N124 O146 S0 |

Figure S24 reports the multi-charged ESI MS and the corresponding deconvoluted spectra of two components with m_mono_ 12801.3807 Da and 12829.3827 Da, that are attributable to the amino acidic region Lys^330^-Lys^443^ of the vicilin-like protein with the Acc. No. tr|A0A1S2XQR4. Particularly, the component with m_mono_ 12829.3827 Da could correspond to this region with a formylation at a lysine residue.


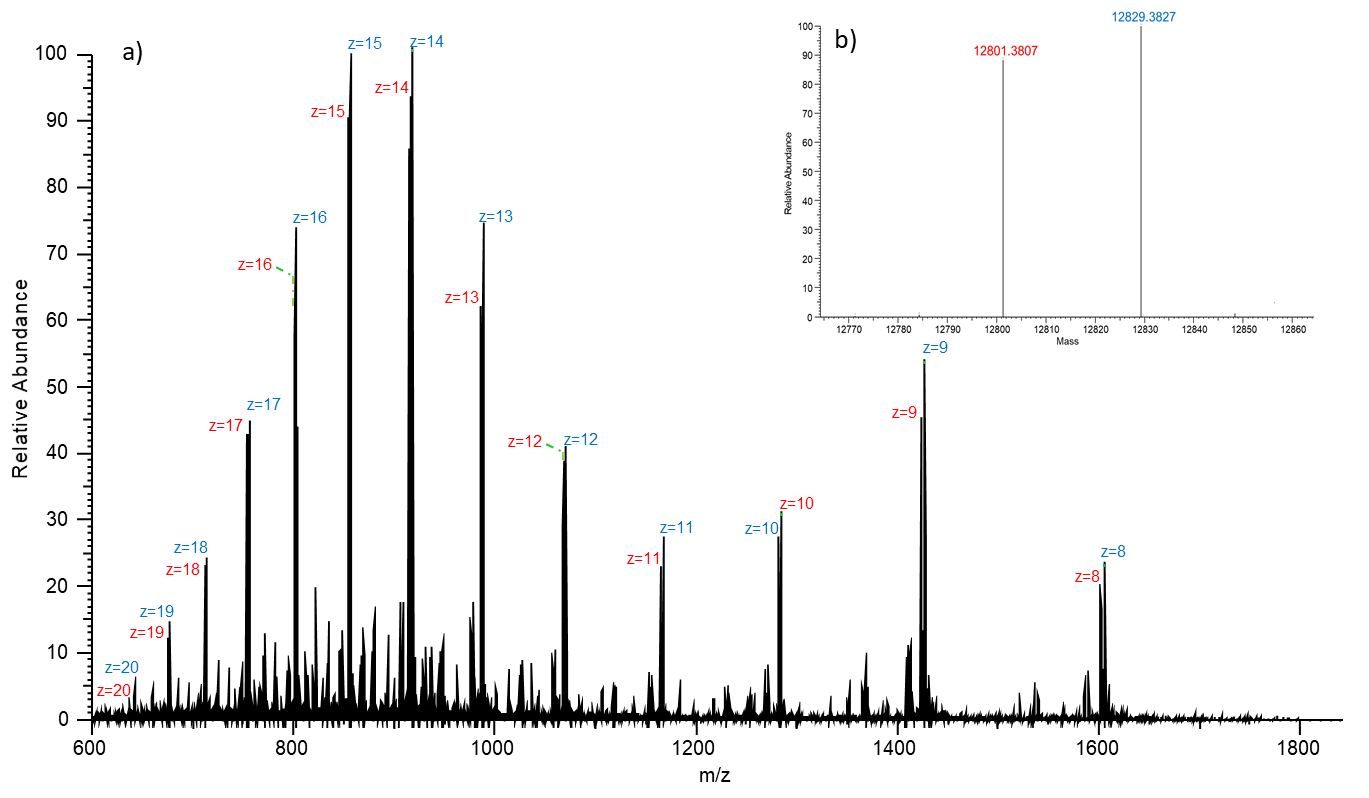


**Figure S24.** a) Multi-charged mass spectrum and b) monoisotopic deconvoluted mass spectrum (mass zero-charge) of the two coeluting polypeptides with MM_mono_ 12801,3807 and 12829,3827 Da.

Following the TD-MS2 of the multi-charged ion at m/z 1281.7458, corresponding to the [M+10H]^10+^ of the polypeptide with m_mono_ 12801.3807 Da.


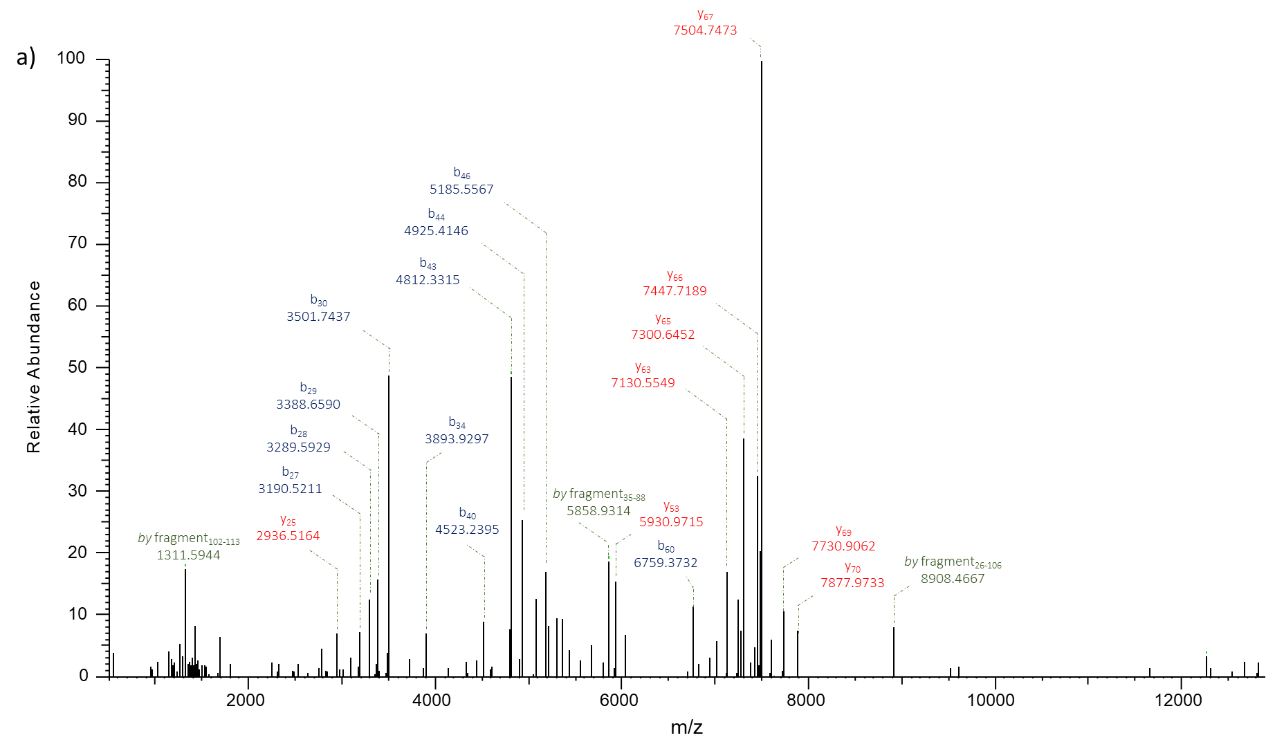


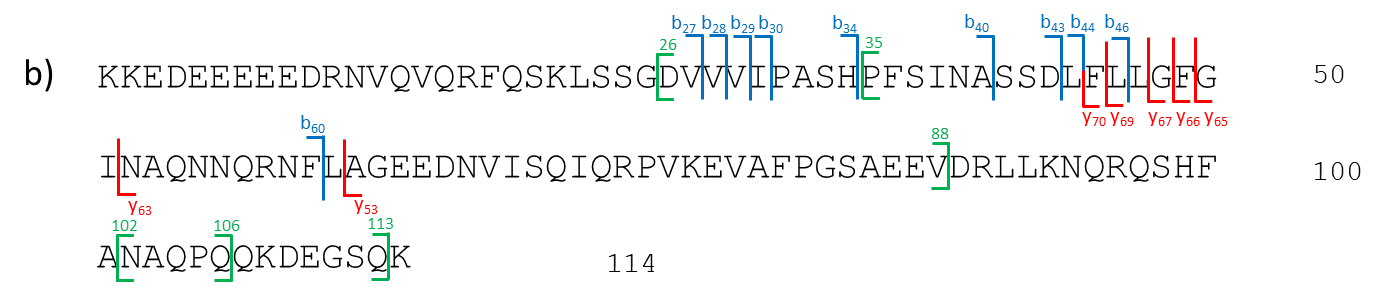


**Figure S25**. a) Deconvoluted MS/MS spectrum of the ion at m/z 1281.7458 which corresponds to the multi-charged ion [M+10H]^10+^ of the polypeptide with m_mono_ 12801.3807 Da. The most abundant y- and b-ions are marked in red and blue, respectively. The most intense internal fragment ions are shown in green. b) Sequence coverage map as obtained by the MS/MS shown in the panel a. This polypeptide corresponds to the region Lys330-Lys443 (here renumbered 1-114) of the vicilin-like protein entry with Acc. No. A0A1S2XQR4. b- and y-fragments are reported in blue and red, respectively. The internal fragments are reported in green.

Following the complete report of the ClipsMS interpretation (Fig. S26, Table S10) of the multi-charged ion [M+10H]^10+^ at m/z 1281.7458 of the polypeptide with m_mono_ 12801.3807 Da. corresponding with the amino acidic region Lys^330^-Lys^443^ of the vicilin-like protein with the Acc. No. tr|A0A1S2XQR4.


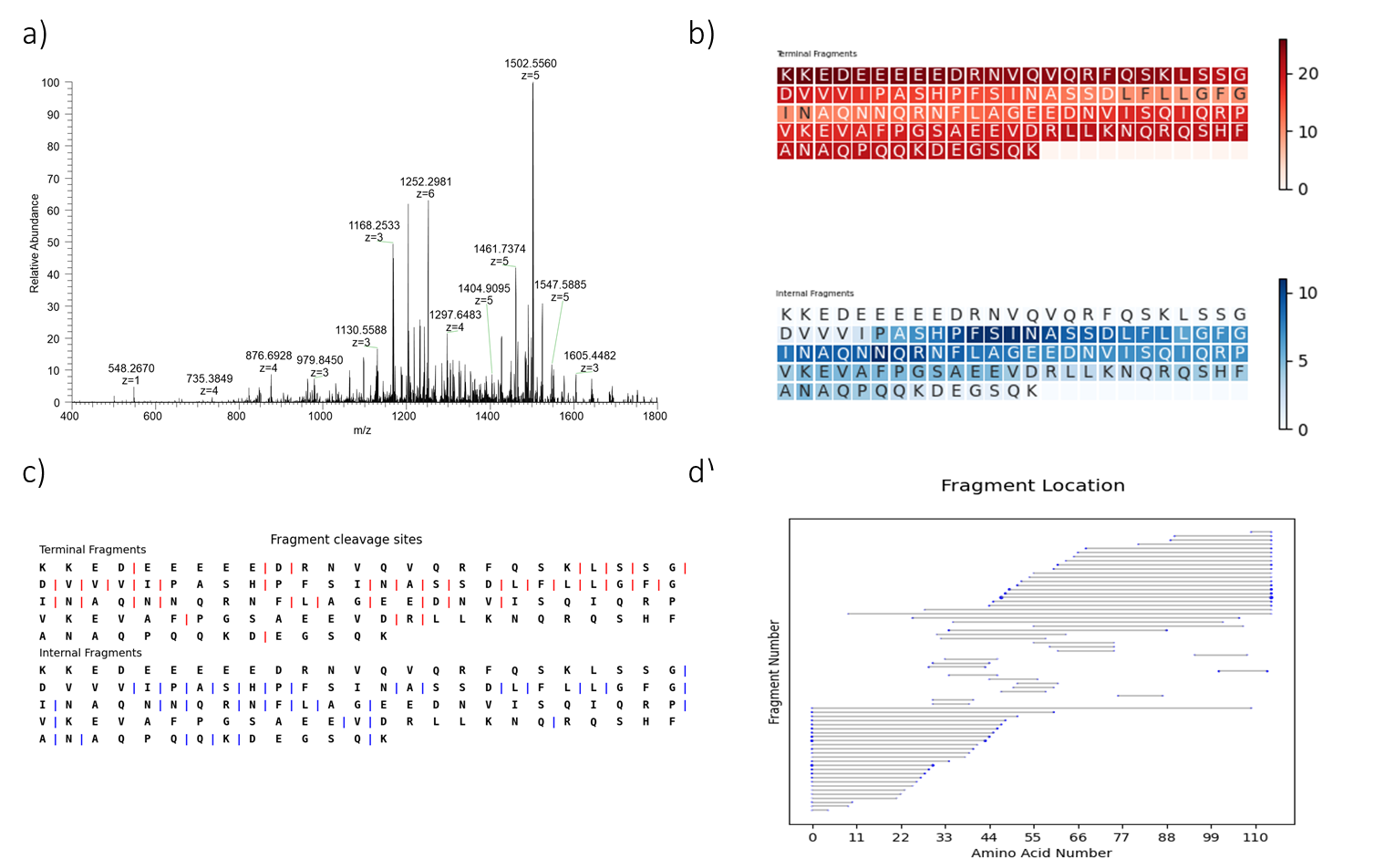


**Figure S26.** a) HCD mass spectrum of the ion at m/z 1281.7458 ([M+10H]^10+^); b) sequence coverage map for the terminal and internal fragments. Darker regions indicate more coverage; c) fragment cleavage map indicating the location of inter-amino-acid cleavage sites for terminal and internal fragments); and d) fragment location map indicating the region of the β-chain sequence covered by terminal and internal fragments.

**Table S10**. Sample output of ClipsMS related to the CID mass spectrum of the ion at m/z 1281.7458 ([M+10H]^10+^). The Table includes the fragment type, observed mass, theoretical mass, start amino acid, end amino acid, error (ppm), sequence, intensity, and molecular formula. The amino acid positions refer to the sequence reported in Figure S25b.

| **Frag Type** | **Observed Mass** | **Theoretical Mass** | **Start AA** | **End AA** | **Error** | **Sequence** | **Intensity** | **Formula** |
| --- | --- | --- | --- | --- | --- | --- | --- | --- |
| B Fragment | 501,266 | 501,267 | 1 | 4 | -0,7 | KKED | 115396,72 | C21 H37 N6 O8 S0 |
| B Fragment | 1146,479 | 1146,480 | 1 | 9 | -0,3 | KKEDEEEEE | 121163,11 | C46 H72 N11 O23 S0 |
| B Fragment | 1261,505 | 1261,507 | 1 | 10 | -1,2 | KKEDEEEEED | 276517,61 | C50 H77 N12 O26 S0 |
| B Fragment | 2632,266 | 2632,260 | 1 | 21 | 2,4 | KKEDEEEEEDRNVQVQRFQSK | 49270,4 | C109 H175 N34 O42 S0 |
| B Fragment | 2745,340 | 2745,344 | 1 | 22 | -1,3 | KKEDEEEEEDRNVQVQRFQSKL | 115418,72 | C115 H186 N35 O43 S0 |
| B Fragment | 2832,378 | 2832,376 | 1 | 23 | 0,6 | KKEDEEEEEDRNVQVQRFQSKLS | 66666,41 | C118 H191 N36 O45 S0 |
| B Fragment | 2976,426 | 2976,429 | 1 | 25 | -1,0 | KKEDEEEEEDRNVQVQRFQSKLSSG | 91542,05 | C123 H199 N38 O48 S0 |
| B Fragment | 3091,457 | 3091,456 | 1 | 26 | 0,3 | KKEDEEEEEDRNVQVQRFQSKLSSGD | 228173,3 | C127 H204 N39 O51 S0 |
| B Fragment | 3190,521 | 3190,525 | 1 | 27 | -1,1 | KKEDEEEEEDRNVQVQRFQSKLSSGDV | 522592,27 | C132 H213 N40 O52 S0 |
| B Fragment | 3289,593 | 3289,593 | 1 | 28 | 0,0 | KKEDEEEEEDRNVQVQRFQSKLSSGDVV | 905833,14 | C137 H222 N41 O53 S0 |
| B Fragment | 3388,659 | 3388,662 | 1 | 29 | -0,7 | KKEDEEEEEDRNVQVQRFQSKLSSGDVVV | 1133062,19 | C142 H231 N42 O54 S0 |
| B Fragment | 3501,744 | 3501,746 | 1 | 30 | -0,6 | KKEDEEEEEDRNVQVQRFQSKLSSGDVVVI | 3500923,23 | C148 H242 N43 O55 S0 |
| B Fragment | 3893,930 | 3893,926 | 1 | 34 | 0,9 | KKEDEEEEEDRNVQVQRFQSKLSSGDVVVIPASH | 509169,25 | C165 H266 N49 O60 S0 |
| B Fragment | 4338,157 | 4338,164 | 1 | 38 | -1,6 | KKEDEEEEEDRNVQVQRFQSKLSSGDVVVIPASHPFSI | 54903,86 | C188 H298 N53 O65 S0 |
| B Fragment | 4452,207 | 4452,207 | 1 | 39 | 0,1 | KKEDEEEEEDRNVQVQRFQSKLSSGDVVVIPASHPFSIN | 206428,51 | C192 H304 N55 O67 S0 |
| B Fragment | 4523,239 | 4523,244 | 1 | 40 | -0,9 | KKEDEEEEEDRNVQVQRFQSKLSSGDVVVIPASHPFSINA | 651267,67 | C195 H309 N56 O68 S0 |
| B Fragment | 4610,275 | 4610,276 | 1 | 41 | -0,3 | KKEDEEEEEDRNVQVQRFQSKLSSGDVVVIPASHPFSINAS | 128582,09 | C198 H314 N57 O70 S0 |
| B Fragment | 4812,331 | 4812,335 | 1 | 43 | -0,7 | KKEDEEEEEDRNVQVQRFQSKLSSGDVVVIPASHPFSINASSD | 3500030,85 | C205 H324 N59 O75 S0 |
| B Fragment | 4925,415 | 4925,419 | 1 | 44 | -0,8 | KKEDEEEEEDRNVQVQRFQSKLSSGDVVVIPASHPFSINASSDL | 1827428,72 | C211 H335 N60 O76 S0 |
| B Fragment | 5072,483 | 5072,487 | 1 | 45 | -0,9 | KKEDEEEEEDRNVQVQRFQSKLSSGDVVVIPASHPFSINASSDLF | 910502,59 | C220 H344 N61 O77 S0 |
| B Fragment | 5185,557 | 5185,571 | 1 | 46 | -2,8 | KKEDEEEEEDRNVQVQRFQSKLSSGDVVVIPASHPFSINASSDLFL | 1232914,27 | C226 H355 N62 O78 S0 |
| B Fragment | 5298,652 | 5298,655 | 1 | 47 | -0,7 | KKEDEEEEEDRNVQVQRFQSKLSSGDVVVIPASHPFSINASSDLFLL | 693176,72 | C232 H366 N63 O79 S0 |
| B Fragment | 5355,668 | 5355,677 | 1 | 48 | -1,7 | KKEDEEEEEDRNVQVQRFQSKLSSGDVVVIPASHPFSINASSDLFLLG | 675767,12 | C234 H369 N64 O80 S0 |
| B Fragment | 5672,862 | 5672,851 | 1 | 51 | 2,1 | KKEDEEEEEDRNVQVQRFQSKLSSGDVVVIPASHPFSINASSDLFLLGFGI | 384013,57 | C251 H392 N67 O83 S0 |
| B Fragment | 6759,373 | 6759,346 | 1 | 60 | 4,0 | KKEDEEEEEDRNVQVQRFQSKLSSGDVVVIPASHPFSINASSDLFLLGFGINAQNNQRNF | 820927,58 | C295 H458 N85 O98 S0 |
| B Fragment | 12255,117 | 12255,140 | 1 | 109 | -1,9 | KKEDEEEEEDRNVQVQRFQSKLSSGDVVVIPASHPFSINASSDLFLLGFGINAQNNQRNFLAGEEDNVISQIQRPVKEVAFPGSAEEVDRLLKNQRQSHFANAQPQQKD | 242682,68 | C532 H837 N158 O176 S0 |
| Y Fragment | 548,267 | 548,267 | 110 | 114 | -0,7 | EGSQK | 290804,27 | C21 H38 N7 O10 S0 |
| Y Fragment | 2780,393 | 2780,419 | 91 | 114 | -9,4 | LLKNQRQSHFANAQPQQKDEGSQK | 342216,43 | C117 H191 N40 O39 S0 |
| Y Fragment | 2936,516 | 2936,520 | 90 | 114 | -1,2 | RLLKNQRQSHFANAQPQQKDEGSQK | 507702,13 | C123 H203 N44 O40 S0 |
| Y Fragment | 3720,853 | 3720,844 | 82 | 114 | 2,6 | PGSAEEVDRLLKNQRQSHFANAQPQQKDEGSQK | 214270,24 | C155 H251 N52 O55 S0 |
| Y Fragment | 5216,682 | 5216,695 | 69 | 114 | -2,5 | ISQIQRPVKEVAFPGSAEEVDRLLKNQRQSHFANAQPQQKDEGSQK | 602545,91 | C224 H364 N71 O73 S0 |
| Y Fragment | 5429,814 | 5429,806 | 67 | 114 | 1,5 | NVISQIQRPVKEVAFPGSAEEVDRLLKNQRQSHFANAQPQQKDEGSQK | 314207,4 | C233 H379 N74 O76 S0 |
| Y Fragment | 5544,825 | 5544,833 | 66 | 114 | -1,5 | DNVISQIQRPVKEVAFPGSAEEVDRLLKNQRQSHFANAQPQQKDEGSQK | 198870,06 | C237 H384 N75 O79 S0 |
| Y Fragment | 5802,906 | 5802,918 | 64 | 114 | -2,1 | EEDNVISQIQRPVKEVAFPGSAEEVDRLLKNQRQSHFANAQPQQKDEGSQK | 171224,6 | C247 H398 N77 O85 S0 |
| Y Fragment | 5930,972 | 5930,977 | 62 | 114 | -0,9 | AGEEDNVISQIQRPVKEVAFPGSAEEVDRLLKNQRQSHFANAQPQQKDEGSQK | 1105964,57 | C252 H406 N79 O87 S0 |
| Y Fragment | 6044,054 | 6044,061 | 61 | 114 | -1,1 | LAGEEDNVISQIQRPVKEVAFPGSAEEVDRLLKNQRQSHFANAQPQQKDEGSQK | 492451,92 | C258 H417 N80 O88 S0 |
| Y Fragment | 6703,355 | 6703,375 | 56 | 114 | -2,9 | NQRNFLAGEEDNVISQIQRPVKEVAFPGSAEEVDRLLKNQRQSHFANAQPQQKDEGSQK | 61064,74 | C286 H458 N91 O96 S0 |
| Y Fragment | 6817,374 | 6817,418 | 55 | 114 | -6,4 | NNQRNFLAGEEDNVISQIQRPVKEVAFPGSAEEVDRLLKNQRQSHFANAQPQQKDEGSQK | 152212,57 | C290 H464 N93 O98 S0 |
| Y Fragment | 7016,509 | 7016,513 | 53 | 114 | -0,7 | AQNNQRNFLAGEEDNVISQIQRPVKEVAFPGSAEEVDRLLKNQRQSHFANAQPQQKDEGSQK | 422492,02 | C298 H477 N96 O101 S0 |
| Y Fragment | 7130,555 | 7130,556 | 52 | 114 | -0,2 | NAQNNQRNFLAGEEDNVISQIQRPVKEVAFPGSAEEVDRLLKNQRQSHFANAQPQQKDEGSQK | 1223089,53 | C302 H483 N98 O103 S0 |
| Y Fragment | 7300,645 | 7300,662 | 50 | 114 | -2,3 | GINAQNNQRNFLAGEEDNVISQIQRPVKEVAFPGSAEEVDRLLKNQRQSHFANAQPQQKDEGSQK | 2784613,35 | C310 H497 N100 O105 S0 |
| Y Fragment | 7447,719 | 7447,730 | 49 | 114 | -1,5 | FGINAQNNQRNFLAGEEDNVISQIQRPVKEVAFPGSAEEVDRLLKNQRQSHFANAQPQQKDEGSQK | 2346834,16 | C319 H506 N101 O106 S0 |
| Y Fragment | 7504,747 | 7504,752 | 48 | 114 | -0,6 | GFGINAQNNQRNFLAGEEDNVISQIQRPVKEVAFPGSAEEVDRLLKNQRQSHFANAQPQQKDEGSQK | 7164929,21 | C321 H509 N102 O107 S0 |
| Y Fragment | 7730,906 | 7730,920 | 46 | 114 | -1,8 | LLGFGINAQNNQRNFLAGEEDNVISQIQRPVKEVAFPGSAEEVDRLLKNQRQSHFANAQPQQKDEGSQK | 769818,26 | C333 H531 N104 O109 S0 |
| Y Fragment | 7877,973 | 7877,988 | 45 | 114 | -1,9 | FLLGFGINAQNNQRNFLAGEEDNVISQIQRPVKEVAFPGSAEEVDRLLKNQRQSHFANAQPQQKDEGSQK | 544406,14 | C342 H540 N105 O110 S0 |
| Y Fragment | 9513,792 | 9513,814 | 29 | 114 | -2,3 | VIPASHPFSINASSDLFLLGFGINAQNNQRNFLAGEEDNVISQIQRPVKEVAFPGSAEEVDRLLKNQRQSHFANAQPQQKDEGSQK | 114420,41 | C416 H653 N124 O133 S0 |
| Y Fragment | 11656,863 | 11656,927 | 10 | 114 | -5,5 | DRNVQVQRFQSKLSSGDVVVIPASHPFSINASSDLFLLGFGINAQNNQRNFLAGEEDNVISQIQRPVKEVAFPGSAEEVDRLLKNQRQSHFANAQPQQKDEGSQK | 107440,79 | C507 H803 N154 O163 S0 |
| BY Int Fragment | 951,467 | 951,468 | 31 | 39 | -1,3 | PASHPFSIN | 127635,77 | C44 H63 N12 O12 S0 |
| BY Int Fragment | 1022,502 | 1022,505 | 31 | 40 | -3,4 | PASHPFSINA | 193288,26 | C47 H68 N13 O13 S0 |
| BY Int Fragment | 1145,558 | 1145,547 | 77 | 87 | 9,3 | KEVAFPGSAEE | 302200,7 | C51 H77 N12 O18 S0 |
| BY Int Fragment | 1200,589 | 1200,587 | 48 | 58 | 2,1 | GFGINAQNNQR | 165292,5 | C50 H78 N19 O16 S0 |
| BY Int Fragment | 1200,589 | 1200,587 | 51 | 60 | 2,1 | INAQNNQRNF | 165292,5 | C50 H78 N19 O16 S0 |
| BY Int Fragment | 1200,589 | 1200,587 | 52 | 61 | 2,1 | NAQNNQRNFL | 165292,5 | C50 H78 N19 O16 S0 |
| BY Int Fragment | 1289,654 | 1289,664 | 45 | 56 | -7,3 | FLLGFGINAQNN | 159465,1 | C60 H89 N16 O16 S0 |
| BY Int Fragment | 1292,648 | 1292,652 | 35 | 46 | -3,1 | PFSINASSDLFL | 238525,07 | C61 H90 N13 O18 S0 |
| BY Int Fragment | 1311,594 | 1311,592 | 102 | 113 | 1,5 | NAQPQQKDEGSQ | 1264204,07 | C52 H83 N18 O22 S0 |
| BY Int Fragment | 1424,687 | 1424,680 | 30 | 43 | 4,6 | IPASHPFSINASSD | 596986,9 | C63 H94 N17 O21 S0 |
| BY Int Fragment | 1424,687 | 1424,680 | 31 | 44 | 4,6 | PASHPFSINASSDL | 596986,9 | C63 H94 N17 O21 S0 |
| BY Int Fragment | 1429,712 | 1429,711 | 34 | 46 | 0,4 | HPFSINASSDLFL | 131118,96 | C67 H97 N16 O19 S0 |
| BY Int Fragment | 1521,768 | 1521,767 | 96 | 108 | 1,0 | RQSHFANAQPQQK | 142947,74 | C65 H101 N24 O19 S0 |
| BY Int Fragment | 1537,759 | 1537,761 | 62 | 75 | -1,3 | AGEEDNVISQIQRP | 132015,38 | C64 H105 N20 O24 S0 |
| BY Int Fragment | 1797,914 | 1797,913 | 60 | 75 | 0,8 | FLAGEEDNVISQIQRP | 162163,89 | C79 H125 N22 O26 S0 |
| BY Int Fragment | 2310,152 | 2310,159 | 56 | 75 | -2,9 | NQRNFLAGEEDNVISQIQRP | 68337,67 | C98 H157 N32 O33 S0 |
| BY Int Fragment | 2829,431 | 2829,407 | 33 | 58 | 8,8 | SHPFSINASSDLFLLGFGINAQNNQR | 85268,83 | C126 H190 N37 O38 S0 |
| BY Int Fragment | 3402,676 | 3402,698 | 32 | 63 | -6,3 | ASHPFSINASSDLFLLGFGINAQNNQRNFLAG | 84105,92 | C153 H229 N44 O45 S0 |
| BY Int Fragment | 5858,931 | 5858,941 | 35 | 88 | -1,7 | PFSINASSDLFLLGFGINAQNNQRNFLAGEEDNVISQIQRPVKEVAFPGSAEEV | 1347972,14 | C261 H402 N71 O83 S0 |
| BY Int Fragment | 5912,957 | 5912,993 | 56 | 107 | -6,1 | NQRNFLAGEEDNVISQIQRPVKEVAFPGSAEEVDRLLKNQRQSHFANAQPQQ | 111139,03 | C255 H404 N81 O82 S0 |
| BY Int Fragment | 7469,725 | 7469,780 | 36 | 102 | -7,4 | FSINASSDLFLLGFGINAQNNQRNFLAGEEDNVISQIQRPVKEVAFPGSAEEVDRLLKNQRQSHFAN | 136897,06 | C329 H512 N97 O103 S0 |
| BY Int Fragment | 8908,467 | 8908,537 | 26 | 106 | -7,9 | DVVVIPASHPFSINASSDLFLLGFGINAQNNQRNFLAGEEDNVISQIQRPVKEVAFPGSAEEVDRLLKNQRQSHFANAQPQ | 586371,64 | C394 H614 N115 O122 S0 |

Figures S27 and S28 show the multi-charged ESI MS and the corresponding deconvoluted spectra of two polypeptides with m_mono_ of 21612.1094 and 35344.0906 Da that are attributable to the amino acidic regions Lys^24^-Asp^208^ and Lys^24^-Asn^329^, respectively, of the vicilin-like protein with the Acc. No. tr|A0A1S2XQR4. The other two co-eluting components with m_mono_ 20163.9871 and 35243.9886 Da, actually remain unidentified.


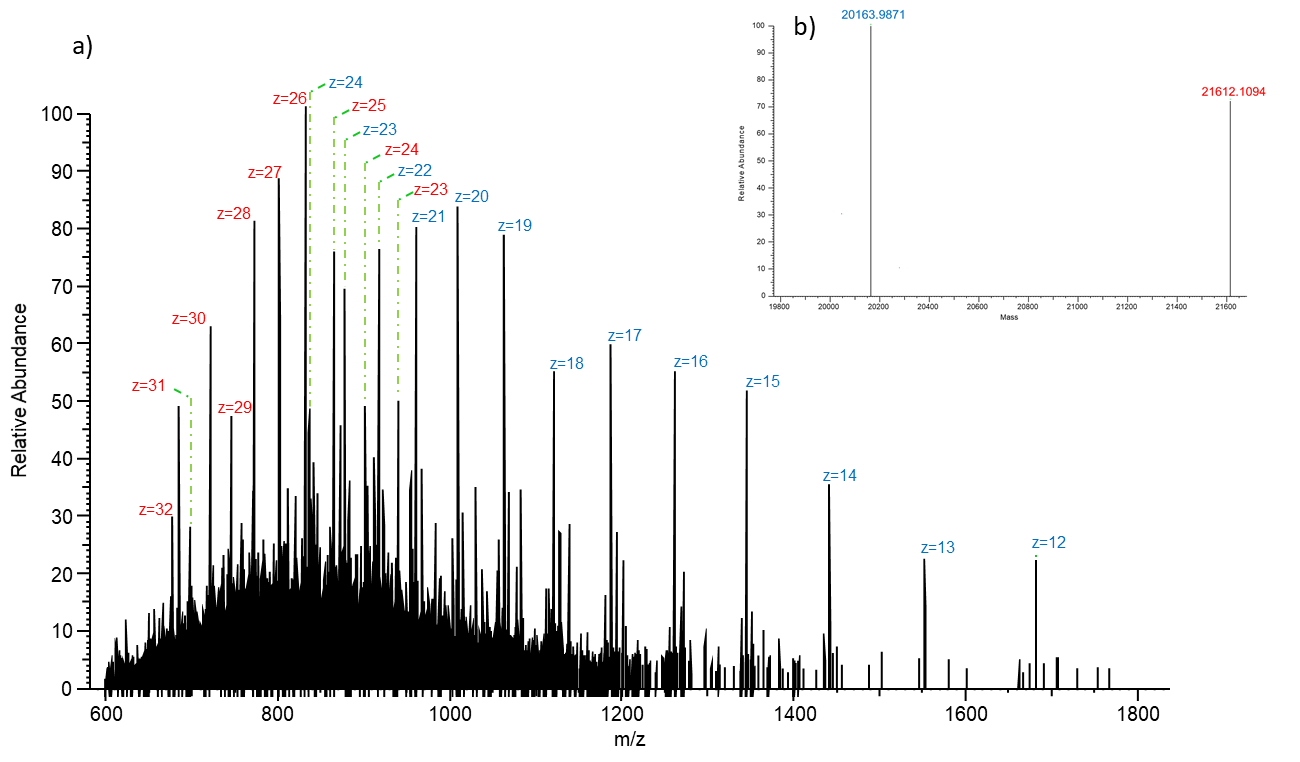


**Figure S27**. a) Multi-charged mass spectrum and b) monoisotopic deconvoluted mass spectrum (mass zero-charge) of the two coeluting polypeptides with m_mono_ 20163.9871 and 21612.1094 Da.

**
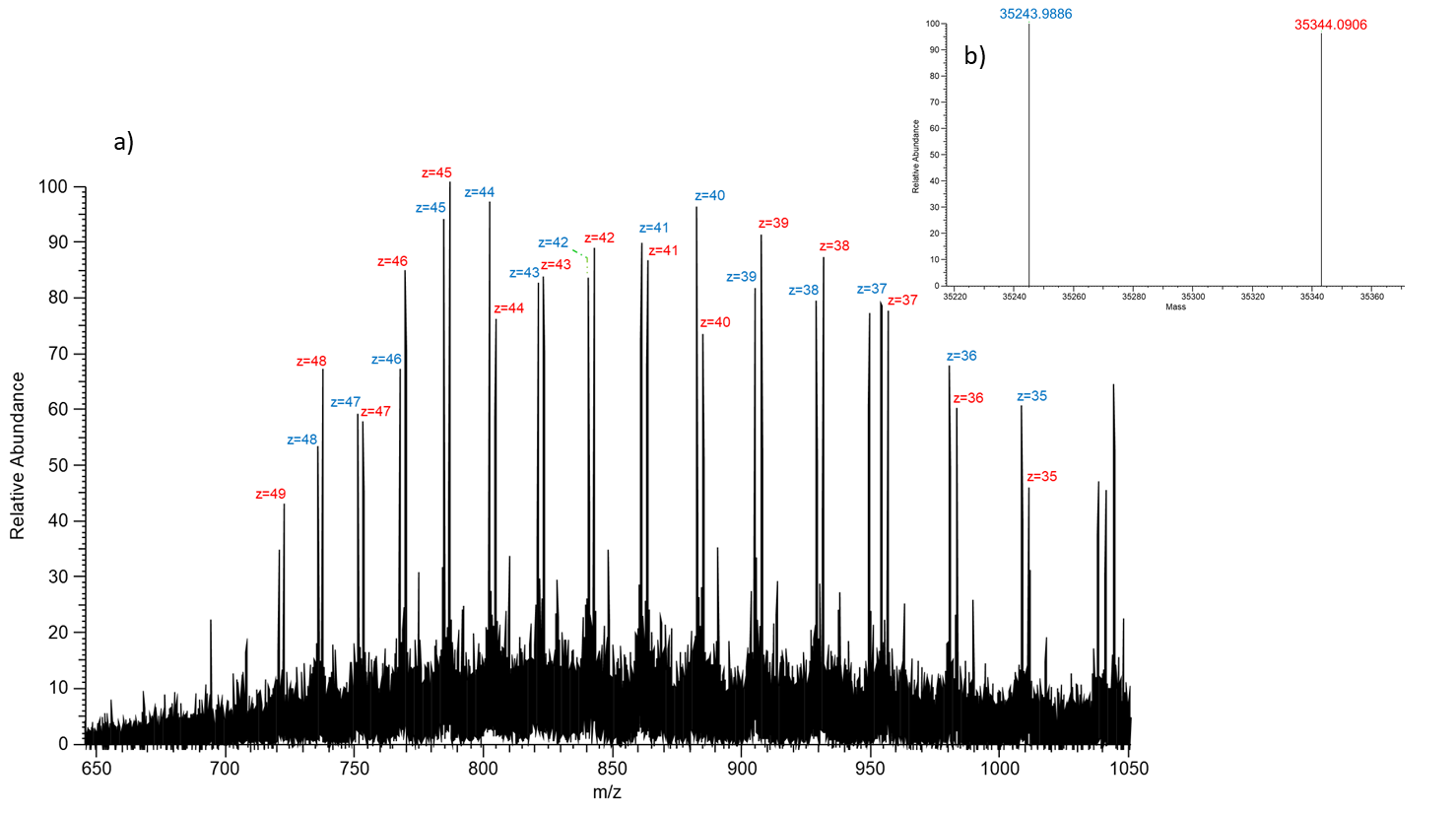
**

**Figure S28.** a) Multi-charged mass spectrum and b) monoisotopic deconvoluted mass spectrum (mass zero-charge) of the two coeluting polypeptides with m_mono_ 35243.9886 and 35344.0906 Da.

***Polypeptide related to the legumin A0A1S2XQ88***

Figures S29 reports the multi-charged ESI MS and the corresponding deconvoluted mass spectra of the polypeptide with experimental monoisotopic molecular masses (m_mono_) of 12498.3445 Da. This component is related to the amino acidic region Lys^328^-Gly^438^ of the vicilin-like protein with the Acc. No. tr|A0A1S2XQ88.


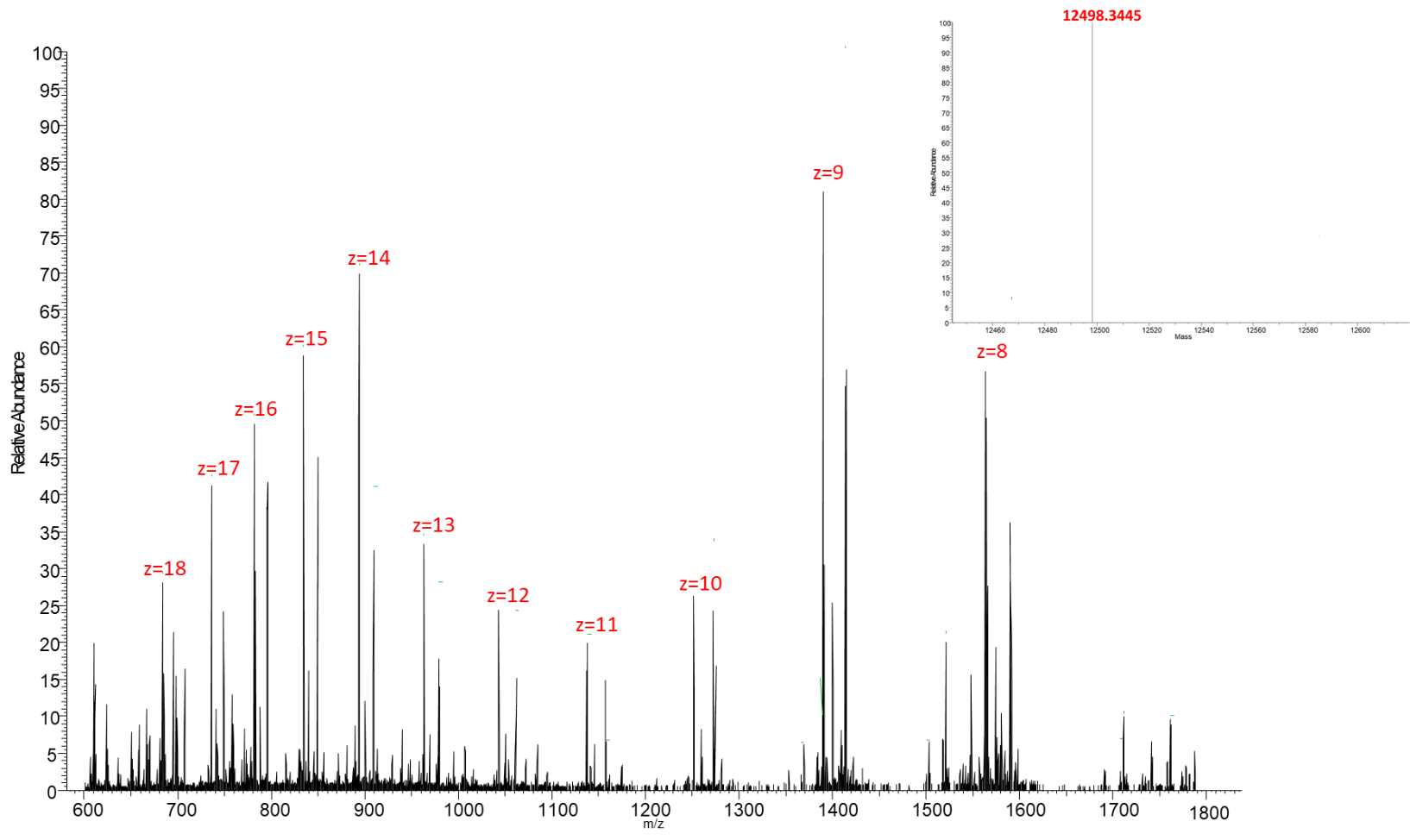


**Figure S29.** a) Multi-charged mass spectrum and b) monoisotopic deconvoluted mass spectrum (mass zero-charge) of the polypeptide with m_mono_ 12498.3445 Da.

Following the TD-MS2 of the multi-charged ion at m/z 834.6984, corresponding to the [M+15H]^15+^ of the polypeptide with m_mono_ 12498.3445 Da


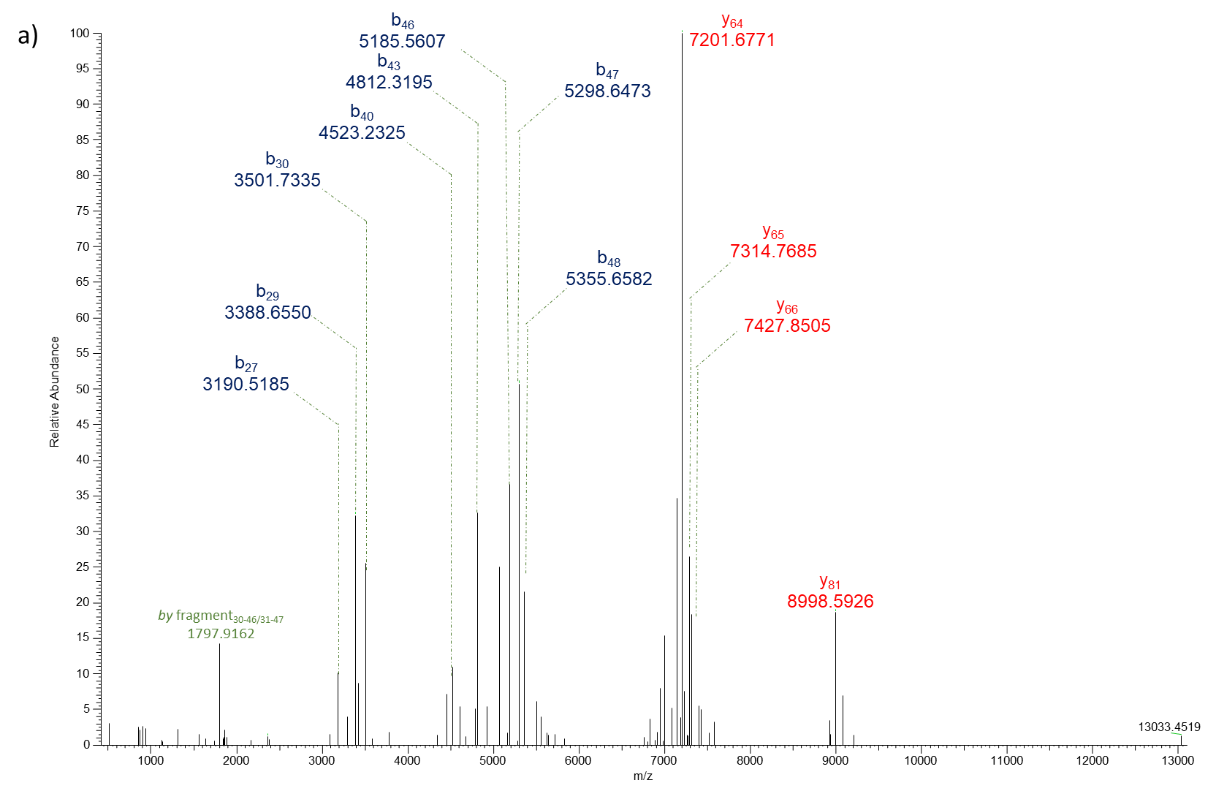


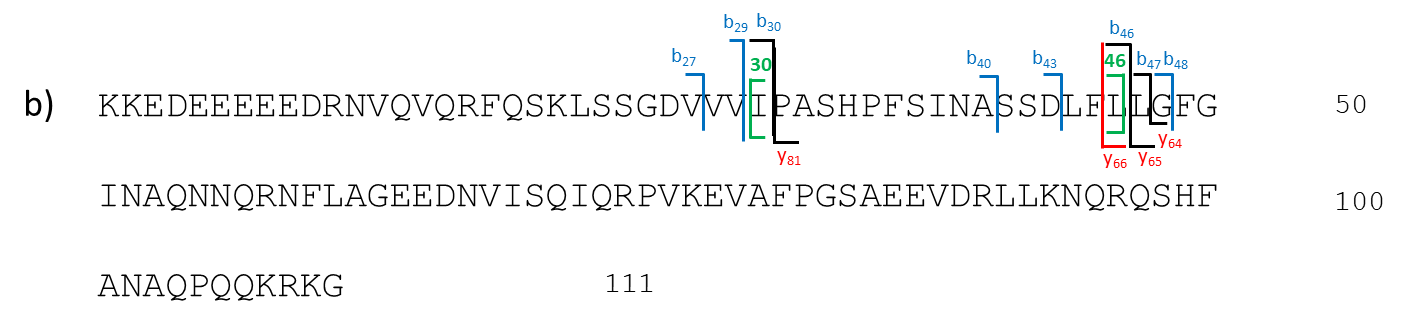


**Figure S30.** a) Deconvoluted MS/MS spectrum of the ion at m/z 834.6984 which corresponds to the multi-charged ion [M+15H]^15+^ of the polypeptide with m_mono_ 12498.3445 Da. The most abundant y- and b-ions are marked in red and blue, respectively. The most intense internal fragment ions are showed in green. b) Sequence coverage map as obtained by the MS/MS showed in the panel a. This polypeptide corresponds to the region Lys328-Gly438 (here renumbered 1-111) of the vicilin-like protein entry with Acc. No. A0A1S2XQ88. b- and y-fragments are reported in blue and red, respectively. The internal fragments are reported in green.

Following the complete report of the ClipsMS interpretation (Fig. S31, Table S11) of the multi-charged ion [M+15H]^15+^ at m/z 834.6984 of the polypeptide with m_mono_ 12498.3445 Da corresponding with the amino acidic region Lys328-Gly438 of the vicilin with the Acc. No. A0A1S2XQ88.


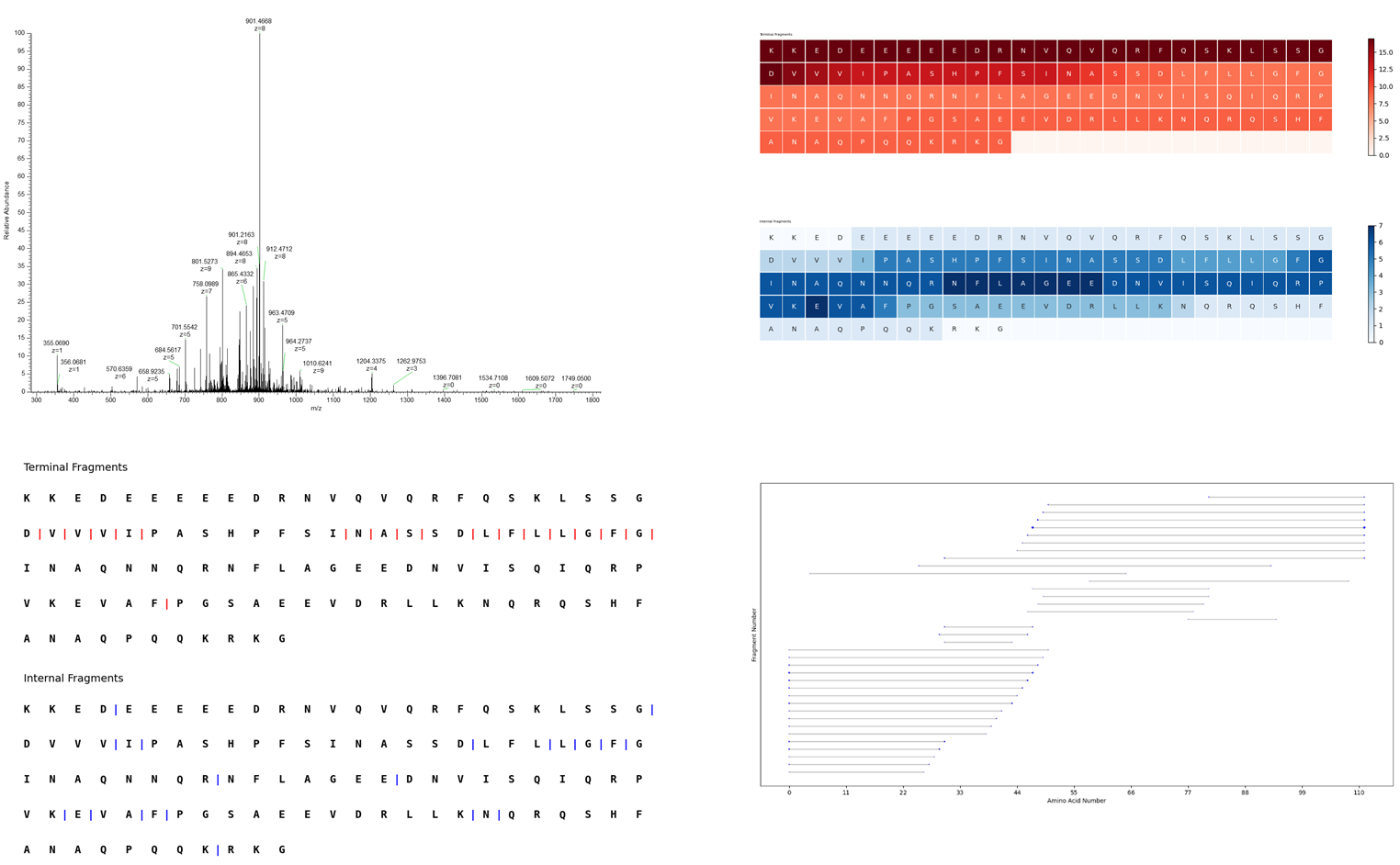


**Figure S31.** a) HCD mass spectrum of the ion at m/z 834.6984 ([M+15H]^15+^); b) sequence coverage map for the terminal and internal fragments. Darker regions indicate more coverage; c) fragment cleavage map indicating the location of inter-amino-acid cleavage sites for terminal and internal fragments); and d) fragment location map indicating the region of the β-chain sequence covered by terminal and internal fragments.

**Table S11**. Sample output of ClipsMS related to the CID mass spectrum of the ion at m/z 834.6984 ([M+15H]^15+^). The Table includes the fragment type, observed mass, theoretical mass, start amino acid, end amino acid, error (ppm), sequence, intensity, and molecular formula. The amino acid positions refer to the sequence reported in Figure S30b.

| **Frag Type** | **Observed Mass** | **Theoretical Mass** | **Start AA** | **End AA** | **Error** | **Sequence** | **Intensity** | **Formula** |
| --- | --- | --- | --- | --- | --- | --- | --- | --- |
| B Fragment | 3091.4513 | 3091.4563 | 1 | 26 | -1.6 | KKEDEEEEEDRNVQVQRFQSKLSSGD | 738.53 | C127 H204 N39 O51 S0 |
| B Fragment | 3190.5185 | 3190.5247 | 1 | 27 | -2.0 | KKEDEEEEEDRNVQVQRFQSKLSSGDV | 5013.94 | C132 H213 N40 O52 S0 |
| B Fragment | 3289.5848 | 3289.5931 | 1 | 28 | -2.5 | KKEDEEEEEDRNVQVQRFQSKLSSGDVV | 1948.19 | C137 H222 N41 O53 S0 |
| B Fragment | 3388.6550 | 3388.6615 | 1 | 29 | -1.9 | KKEDEEEEEDRNVQVQRFQSKLSSGDVVV | 15818.47 | C142 H231 N42 O54 S0 |
| B Fragment | 3501.7335 | 3501.7456 | 1 | 30 | -3.5 | KKEDEEEEEDRNVQVQRFQSKLSSGDVVVI | 12534.78 | C148 H242 N43 O55 S0 |
| B Fragment | 4338.1315 | 4338.1637 | 1 | 38 | -7.4 | KKEDEEEEEDRNVQVQRFQSKLSSGDVVVIPASHPFSI | 725.6 | C188 H298 N53 O65 S0 |
| B Fragment | 4452.1998 | 4452.2066 | 1 | 39 | -1.5 | KKEDEEEEEDRNVQVQRFQSKLSSGDVVVIPASHPFSIN | 3519.98 | C192 H304 N55 O67 S0 |
| B Fragment | 4523.2325 | 4523.2437 | 1 | 40 | -2.5 | KKEDEEEEEDRNVQVQRFQSKLSSGDVVVIPASHPFSINA | 5412.08 | C195 H309 N56 O68 S0 |
| B Fragment | 4610.2679 | 4610.2758 | 1 | 41 | -1.7 | KKEDEEEEEDRNVQVQRFQSKLSSGDVVVIPASHPFSINAS | 2716.54 | C198 H314 N57 O70 S0 |
| B Fragment | 4812.3195 | 4812.3347 | 1 | 43 | -3.2 | KKEDEEEEEDRNVQVQRFQSKLSSGDVVVIPASHPFSINASSD | 16015.1 | C205 H324 N59 O75 S0 |
| B Fragment | 4925.4115 | 4925.4188 | 1 | 44 | -1.5 | KKEDEEEEEDRNVQVQRFQSKLSSGDVVVIPASHPFSINASSDL | 2712.11 | C211 H335 N60 O76 S0 |
| B Fragment | 5072.4768 | 5072.4872 | 1 | 45 | -2.1 | KKEDEEEEEDRNVQVQRFQSKLSSGDVVVIPASHPFSINASSDLF | 12343.47 | C220 H344 N61 O77 S0 |
| B Fragment | 5185.5607 | 5185.5713 | 1 | 46 | -2.0 | KKEDEEEEEDRNVQVQRFQSKLSSGDVVVIPASHPFSINASSDLFL | 17995.03 | C226 H355 N62 O78 S0 |
| B Fragment | 5298.6473 | 5298.6553 | 1 | 47 | -1.5 | KKEDEEEEEDRNVQVQRFQSKLSSGDVVVIPASHPFSINASSDLFLL | 24897.88 | C232 H366 N63 O79 S0 |
| B Fragment | 5355.6582 | 5355.6768 | 1 | 48 | -3.5 | KKEDEEEEEDRNVQVQRFQSKLSSGDVVVIPASHPFSINASSDLFLLG | 10538.41 | C234 H369 N64 O80 S0 |
| B Fragment | 5502.7416 | 5502.7452 | 1 | 49 | -0.7 | KKEDEEEEEDRNVQVQRFQSKLSSGDVVVIPASHPFSINASSDLFLLGF | 3037.73 | C243 H378 N65 O81 S0 |
| B Fragment | 5559.7554 | 5559.7667 | 1 | 50 | -2.0 | KKEDEEEEEDRNVQVQRFQSKLSSGDVVVIPASHPFSINASSDLFLLGFG | 1970.24 | C245 H381 N66 O82 S0 |
| Y Fragment | 3417.7754 | 3417.7847 | 82 | 111 | -2.7 | PGSAEEVDRLLKNQRQSHFANAQPQQKRKG | 4260.82 | C144 H238 N51 O46 S0 |
| Y Fragment | 6940.5904 | 6940.5815 | 51 | 111 | 1.3 | INAQNNQRNFLAGEEDNVISQIQRPVKEVAFPGSAEEVDRLLKNQRQSHFANAQPQQKRKG | 3916.73 | C297 H481 N98 O95 S0 |
| Y Fragment | 6997.6033 | 6997.6029 | 50 | 111 | 0.1 | GINAQNNQRNFLAGEEDNVISQIQRPVKEVAFPGSAEEVDRLLKNQRQSHFANAQPQQKRKG | 7543.58 | C299 H484 N99 O96 S0 |
| Y Fragment | 7144.6584 | 7144.6713 | 49 | 111 | -1.8 | FGINAQNNQRNFLAGEEDNVISQIQRPVKEVAFPGSAEEVDRLLKNQRQSHFANAQPQQKRKG | 17033.32 | C308 H493 N100 O97 S0 |
| Y Fragment | 7201.6771 | 7201.6928 | 48 | 111 | -2.2 | GFGINAQNNQRNFLAGEEDNVISQIQRPVKEVAFPGSAEEVDRLLKNQRQSHFANAQPQQKRKG | 48991.81 | C310 H496 N101 O98 S0 |
| Y Fragment | 7314.7685 | 7314.7769 | 47 | 111 | -1.1 | LGFGINAQNNQRNFLAGEEDNVISQIQRPVKEVAFPGSAEEVDRLLKNQRQSHFANAQPQQKRKG | 9028.73 | C316 H507 N102 O99 S0 |
| Y Fragment | 7427.8505 | 7427.8609 | 46 | 111 | -1.4 | LLGFGINAQNNQRNFLAGEEDNVISQIQRPVKEVAFPGSAEEVDRLLKNQRQSHFANAQPQQKRKG | 2438 | C322 H518 N103 O100 S0 |
| Y Fragment | 7574.9118 | 7574.9293 | 45 | 111 | -2.3 | FLLGFGINAQNNQRNFLAGEEDNVISQIQRPVKEVAFPGSAEEVDRLLKNQRQSHFANAQPQQKRKG | 1631.67 | C331 H527 N104 O101 S0 |
| Y Fragment | 8998.5926 | 8998.6025 | 31 | 111 | -1.1 | PASHPFSINASSDLFLLGFGINAQNNQRNFLAGEEDNVISQIQRPVKEVAFPGSAEEVDRLLKNQRQSHFANAQPQQKRKG | 9136.36 | C394 H620 N121 O122 S0 |
| BY Int Fragment | 1311.5930 | 1311.5964 | 31 | 43 | -2.6 | PASHPFSINASSD | 1103.6 | C57 H83 N16 O20 S0 |
| BY Int Fragment | 1797.9162 | 1797.9170 | 30 | 46 | -0.5 | IPASHPFSINASSDLFL | 6988.72 | C84 H125 N20 O24 S0 |
| BY Int Fragment | 1797.9162 | 1797.9170 | 31 | 47 | -0.5 | PASHPFSINASSDLFLL | 6988.72 | C84 H125 N20 O24 S0 |
| BY Int Fragment | 1855.9372 | 1855.9549 | 78 | 94 | -9.5 | EVAFPGSAEEVDRLLKN | 1040.42 | C82 H131 N22 O27 S0 |
| BY Int Fragment | 3580.8076 | 3580.8255 | 47 | 78 | -5.0 | LGFGINAQNNQRNFLAGEEDNVISQIQRPVKE | 490.64 | C155 H247 N48 O50 S0 |
| BY Int Fragment | 3580.8076 | 3580.8255 | 49 | 80 | -5.0 | FGINAQNNQRNFLAGEEDNVISQIQRPVKEVA | 490.64 | C155 H247 N48 O50 S0 |
| BY Int Fragment | 3580.8076 | 3580.8255 | 50 | 81 | -5.0 | GINAQNNQRNFLAGEEDNVISQIQRPVKEVAF | 490.64 | C155 H247 N48 O50 S0 |
| BY Int Fragment | 3784.8929 | 3784.9154 | 48 | 81 | -6.0 | GFGINAQNNQRNFLAGEEDNVISQIQRPVKEVAF | 956.86 | C166 H259 N50 O52 S0 |
| BY Int Fragment | 5642.8955 | 5642.8851 | 59 | 108 | 1.8 | NFLAGEEDNVISQIQRPVKEVAFPGSAEEVDRLLKNQRQSHFANAQPQQK | 688.4 | C246 H390 N75 O78 S0 |
| BY Int Fragment | 6758.3310 | 6758.3146 | 5 | 65 | 2.4 | EEEEEDRNVQVQRFQSKLSSGDVVVIPASHPFSINASSDLFLLGFGINAQNNQRNFLAGEE | 523.03 | C295 H455 N84 O99 S0 |
| BY Int Fragment | 7401.7936 | 7401.8295 | 26 | 93 | -4.9 | DVVVIPASHPFSINASSDLFLLGFGINAQNNQRNFLAGEEDNVISQIQRPVKEVAFPGSAEEVDRLLK | 2771 | C331 H520 N91 O102 S0 |

***Characterization of the structure of the N-glican linked to the vicilin A0A1S2XQR4***


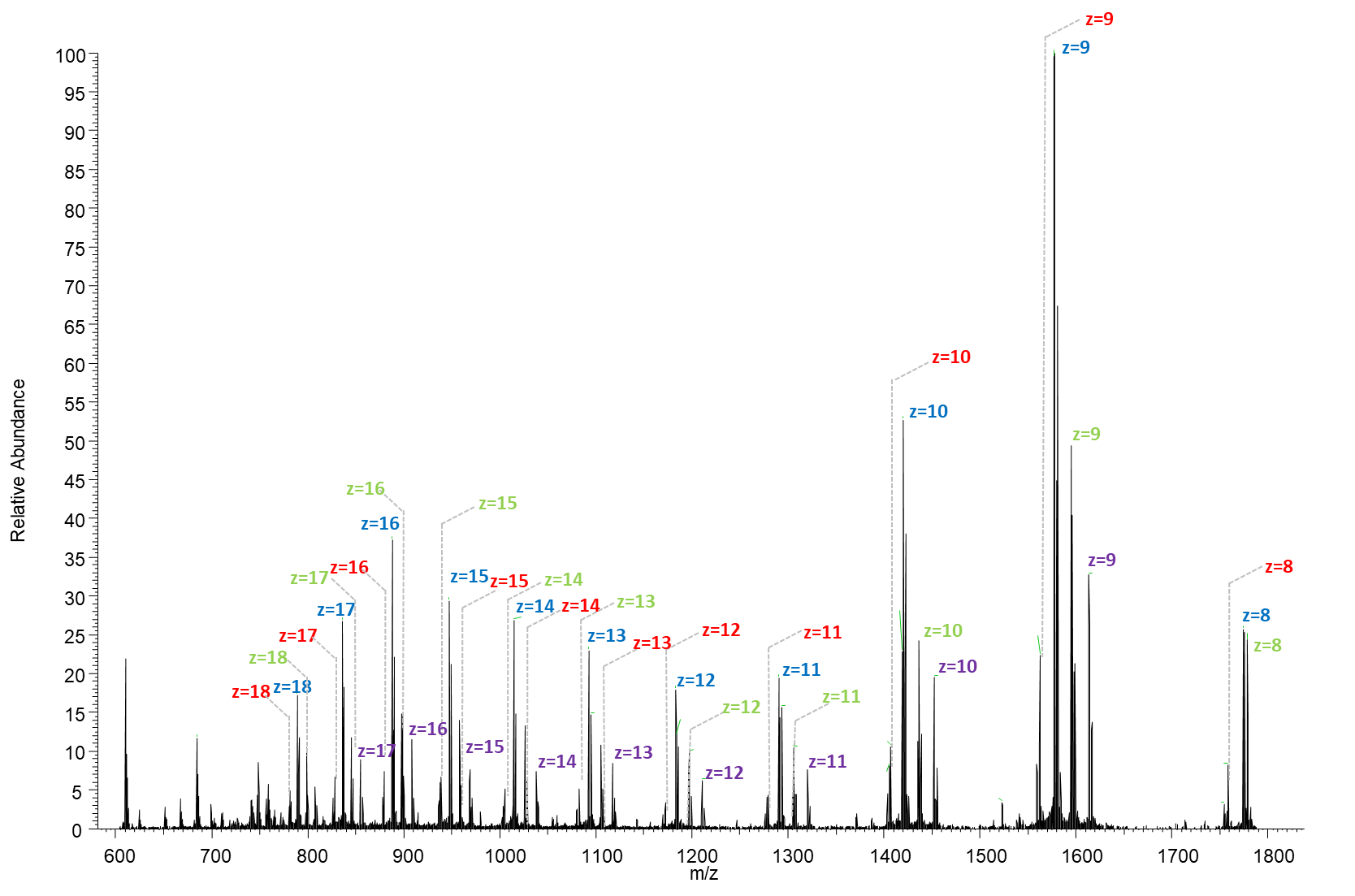


**Figure S32.** a) Multi-charged mass spectrum and b) monoisotopic deconvoluted mass spectrum (mass zero-charge) of the glycol-polypeptides with m_mono_ 14016.8287, 14178.8771, 14340.9381, and 14502.9877 Da


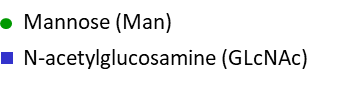


(A)


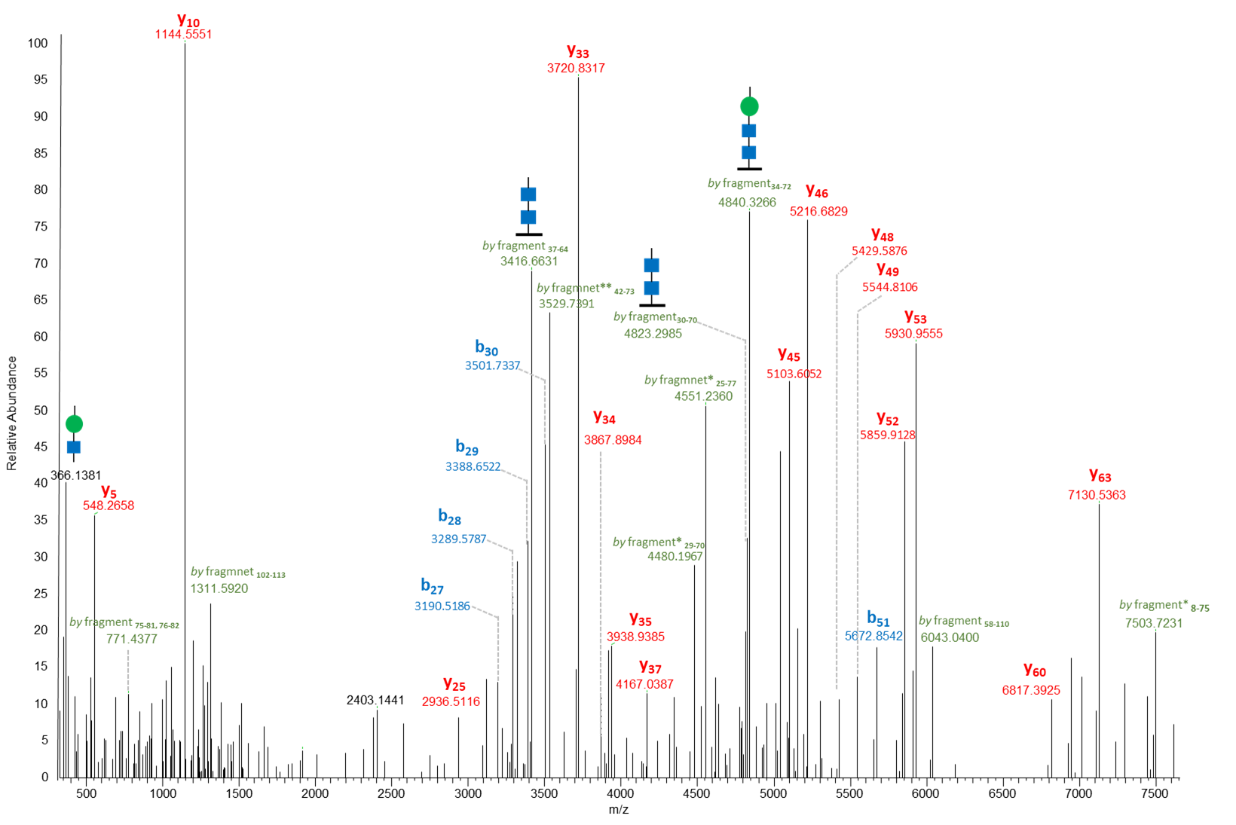


(B)


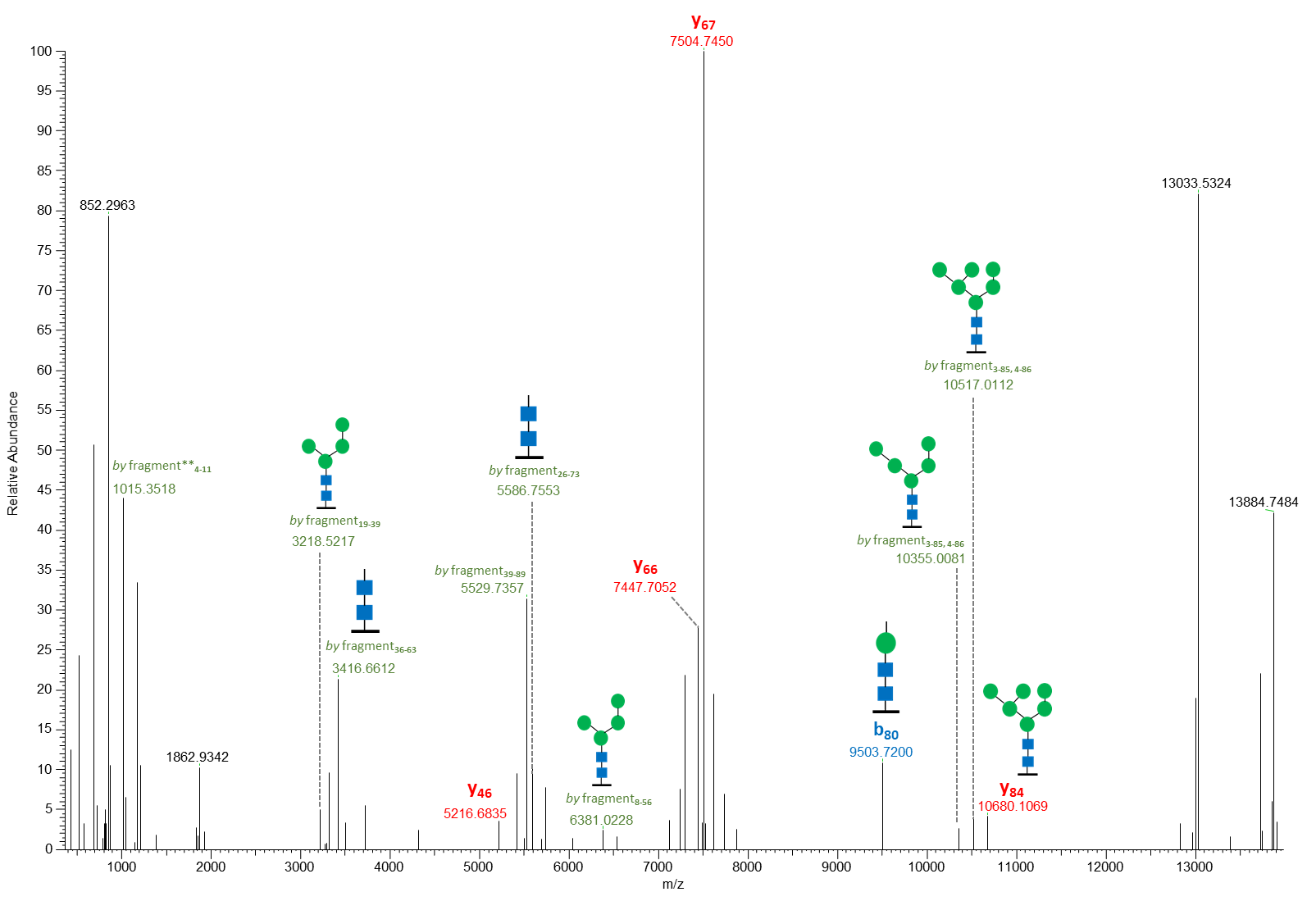


**Figure S33.** Deconvoluted (zero-charge) MS/MS of the spectrum obtained by HCD fragmentation (normalized collision energy: 22) (A) and by CID fragmentation (normalized collision energy: 30) (B) of the ion at m/z 887.6879, which corresponds to the multi-charged ion [M+16H]^16+^ of the polypeptide with m_mono_ 14178.8771 Da. The most abundant y- and b-ions are marked in red and blue, respectively. The most intense internal fragment ions are shown in green. The signals assigned to putative N-glycosylated fragments are labelled on top and show the portion hypothesized structure of the linked-glycan.


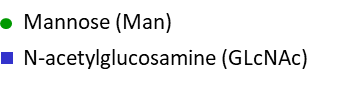


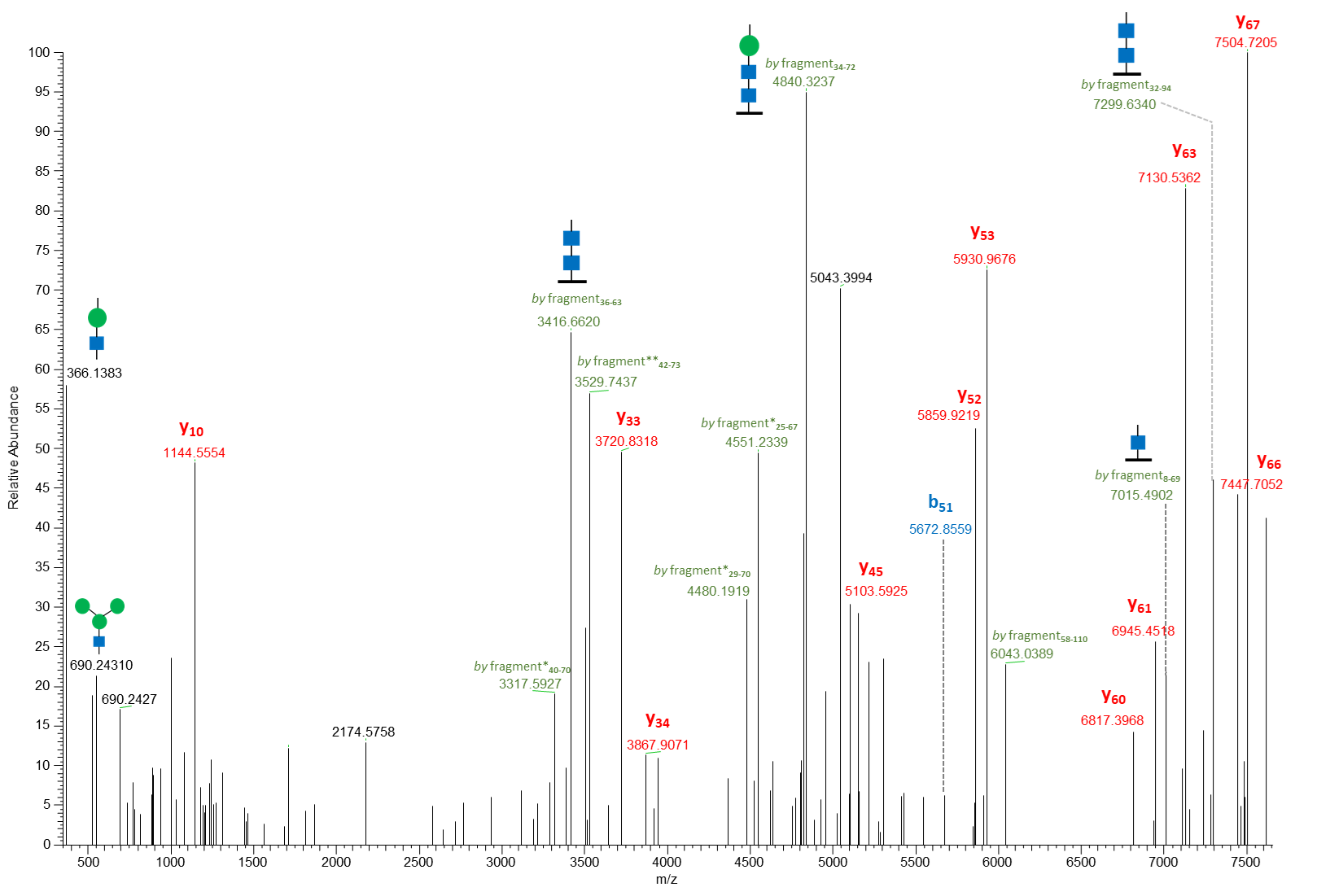


**Figure S34.** Deconvoluted (zero-charge) MS/MS of the spectra obtained by HCD fragmentation (normalized collision energy: 22) of the ion at m/z 946.2655, which corresponds to the multi-charged ion [M+15H]^15+^ of the polypeptide with m_mono_ 14178.8771 Da. The most abundant y- and b-ions are marked in red and blue, respectively. The most intense internal fragment ions are shown in green. The signals assigned to putative N-glycosylated fragments are labelled on top and show the portion hypothesized structure of the linked-glycan.

**Table S12**. Sample output of ClipsMS related to the HCD mass spectrum (Fig. S33A) of the at m/z 887.6879, [M+16H]^16+^. The Table includes the fragment type, observed mass, theoretical mass, start amino acid, end amino acid, error (ppm), sequence, intensity, and molecular formula. The amino acid positions refer to the sequence of gamma-chain of vicilin A0A1S2XQR4.

| **Frag Type** | **Observed Mass** | **Theoretical Mass** | **Start AA** | **End AA** | **Error** | **Sequence** | **Intensity** | **Formula** |
| --- | --- | --- | --- | --- | --- | --- | --- | --- |
| B Fragment | 501.2652 | 501.2667 | 1 | 4 | -3.1 | KKED | 2815.58 | C21 H37 N6 O8 S0 |
| B Fragment | 759.3495 | 759.3519 | 1 | 6 | -3.2 | KKEDEE | 892.7 | C31 H51 N8 O14 S0 |
| B Fragment | 2745.3345 | 2745.3438 | 1 | 22 | -3.4 | KKEDEEEEEDRNVQVQRFQSKL | 996.59 | C115 H186 N35 O43 S0 |
| B Fragment | 3091.4381 | 3091.4563 | 1 | 26 | -5.9 | KKEDEEEEEDRNVQVQRFQSKLSSGD | 1464.36 | C127 H204 N39 O51 S0 |
| B Fragment | 3190.5136 | 3190.5247 | 1 | 27 | -3.5 | KKEDEEEEEDRNVQVQRFQSKLSSGDV | 4308.87 | C132 H213 N40 O52 S0 |
| B Fragment | 3289.5787 | 3289.5931 | 1 | 28 | -4.4 | KKEDEEEEEDRNVQVQRFQSKLSSGDVV | 8239.39 | C137 H222 N41 O53 S0 |
| B Fragment | 3388.6522 | 3388.6615 | 1 | 29 | -2.8 | KKEDEEEEEDRNVQVQRFQSKLSSGDVVV | 10586.98 | C142 H231 N42 O54 S0 |
| B Fragment | 3501.7337 | 3501.7456 | 1 | 30 | -3.4 | KKEDEEEEEDRNVQVQRFQSKLSSGDVVVI | 14868.95 | C148 H242 N43 O55 S0 |
| B Fragment | 3893.9118 | 3893.9264 | 1 | 34 | -3.8 | KKEDEEEEEDRNVQVQRFQSKLSSGDVVVIPASH | 1124.76 | C165 H266 N49 O60 S0 |
| B Fragment | 4138.0343 | 4138.0476 | 1 | 36 | -3.2 | KKEDEEEEEDRNVQVQRFQSKLSSGDVVVIPASHPF | 744.64 | C179 H282 N51 O62 S0 |
| B Fragment | 4452.1925 | 4452.2066 | 1 | 39 | -3.2 | KKEDEEEEEDRNVQVQRFQSKLSSGDVVVIPASHPFSIN | 1203.24 | C192 H304 N55 O67 S0 |
| B Fragment | 4523.2155 | 4523.2437 | 1 | 40 | -6.2 | KKEDEEEEEDRNVQVQRFQSKLSSGDVVVIPASHPFSINA | 3215.93 | C195 H309 N56 O68 S0 |
| B Fragment | 4812.3242 | 4812.3347 | 1 | 43 | -2.2 | KKEDEEEEEDRNVQVQRFQSKLSSGDVVVIPASHPFSINASSD | 6526.44 | C205 H324 N59 O75 S0 |
| B Fragment | 4925.3879 | 4925.4188 | 1 | 44 | -6.3 | KKEDEEEEEDRNVQVQRFQSKLSSGDVVVIPASHPFSINASSDL | 1349.18 | C211 H335 N60 O76 S0 |
| B Fragment | 5672.8542 | 5672.8508 | 1 | 51 | 0.6 | KKEDEEEEEDRNVQVQRFQSKLSSGDVVVIPASHPFSINASSDLFLLGFGI | 5822.23 | C251 H392 N67 O83 S0 |
| Y Fragment | 419.2233 | 419.2249 | 111 | 114 | -3.8 | GSQK | 3644.91 | C16 H31 N6 O7 S0 |
| Y Fragment | 548.2658 | 548.2675 | 110 | 114 | -3.1 | EGSQK | 11696.17 | C21 H38 N7 O10 S0 |
| Y Fragment | 1144.5551 | 1144.5593 | 105 | 114 | -3.7 | PQQKDEGSQK | 32733.52 | C46 H78 N15 O19 S0 |
| Y Fragment | 1457.6912 | 1457.6979 | 102 | 114 | -4.6 | NAQPQQKDEGSQK | 1045.63 | C58 H97 N20 O24 S0 |
| Y Fragment | 1528.7307 | 1528.7350 | 101 | 114 | -2.8 | ANAQPQQKDEGSQK | 397.07 | C61 H102 N21 O25 S0 |
| Y Fragment | 2936.5116 | 2936.5198 | 90 | 114 | -2.8 | RLLKNQRQSHFANAQPQQKDEGSQK | 2711.41 | C123 H203 N44 O40 S0 |
| Y Fragment | 3279.6496 | 3279.6577 | 87 | 114 | -2.5 | EVDRLLKNQRQSHFANAQPQQKDEGSQK | 1518.33 | C137 H224 N47 O47 S0 |
| Y Fragment | 3623.7865 | 3623.7909 | 83 | 114 | -1.2 | GSAEEVDRLLKNQRQSHFANAQPQQKDEGSQK | 2044.02 | C150 H244 N51 O54 S0 |
| Y Fragment | 3720.8317 | 3720.8437 | 82 | 114 | -3.2 | PGSAEEVDRLLKNQRQSHFANAQPQQKDEGSQK | 31255.11 | C155 H251 N52 O55 S0 |
| Y Fragment | 3867.8984 | 3867.9121 | 81 | 114 | -3.6 | FPGSAEEVDRLLKNQRQSHFANAQPQQKDEGSQK | 3763.15 | C164 H260 N53 O56 S0 |
| Y Fragment | 3938.9385 | 3938.9492 | 80 | 114 | -2.7 | AFPGSAEEVDRLLKNQRQSHFANAQPQQKDEGSQK | 5872.28 | C167 H265 N54 O57 S0 |
| Y Fragment | 4038.0064 | 4038.0176 | 79 | 114 | -2.8 | VAFPGSAEEVDRLLKNQRQSHFANAQPQQKDEGSQK | 1803.57 | C172 H274 N55 O58 S0 |
| Y Fragment | 4167.0387 | 4167.0602 | 78 | 114 | -5.2 | EVAFPGSAEEVDRLLKNQRQSHFANAQPQQKDEGSQK | 3747.19 | C177 H281 N56 O61 S0 |
| Y Fragment | 4775.4210 | 4775.4361 | 73 | 114 | -3.2 | QRPVKEVAFPGSAEEVDRLLKNQRQSHFANAQPQQKDEGSQK | 3170.6 | C204 H329 N66 O67 S0 |
| Y Fragment | 4888.5038 | 4888.5201 | 72 | 114 | -3.3 | IQRPVKEVAFPGSAEEVDRLLKNQRQSHFANAQPQQKDEGSQK | 2306.77 | C210 H340 N67 O68 S0 |
| Y Fragment | 5016.5636 | 5016.5787 | 71 | 114 | -3.0 | QIQRPVKEVAFPGSAEEVDRLLKNQRQSHFANAQPQQKDEGSQK | 1712.18 | C215 H348 N69 O70 S0 |
| Y Fragment | 5103.6052 | 5103.6107 | 70 | 114 | -1.1 | SQIQRPVKEVAFPGSAEEVDRLLKNQRQSHFANAQPQQKDEGSQK | 17681.47 | C218 H353 N70 O72 S0 |
| Y Fragment | 5216.6829 | 5216.6948 | 69 | 114 | -2.3 | ISQIQRPVKEVAFPGSAEEVDRLLKNQRQSHFANAQPQQKDEGSQK | 24892.06 | C224 H364 N71 O73 S0 |
| Y Fragment | 5429.7856 | 5429.8061 | 67 | 114 | -3.8 | NVISQIQRPVKEVAFPGSAEEVDRLLKNQRQSHFANAQPQQKDEGSQK | 3512.29 | C233 H379 N74 O76 S0 |
| Y Fragment | 5544.8106 | 5544.8331 | 66 | 114 | -4.1 | DNVISQIQRPVKEVAFPGSAEEVDRLLKNQRQSHFANAQPQQKDEGSQK | 4529.23 | C237 H384 N75 O79 S0 |
| Y Fragment | 5859.9128 | 5859.9397 | 63 | 114 | -4.6 | GEEDNVISQIQRPVKEVAFPGSAEEVDRLLKNQRQSHFANAQPQQKDEGSQK | 14991.2 | C249 H401 N78 O86 S0 |
| Y Fragment | 5930.9555 | 5930.9769 | 62 | 114 | -3.6 | AGEEDNVISQIQRPVKEVAFPGSAEEVDRLLKNQRQSHFANAQPQQKDEGSQK | 19408.41 | C252 H406 N79 O87 S0 |
| Y Fragment | 6191.1074 | 6191.1293 | 60 | 114 | -3.5 | FLAGEEDNVISQIQRPVKEVAFPGSAEEVDRLLKNQRQSHFANAQPQQKDEGSQK | 625.24 | C267 H426 N81 O89 S0 |
| Y Fragment | 6817.3925 | 6817.4178 | 55 | 114 | -3.7 | NNQRNFLAGEEDNVISQIQRPVKEVAFPGSAEEVDRLLKNQRQSHFANAQPQQKDEGSQK | 3482.37 | C290 H464 N93 O98 S0 |
| Y Fragment | 7016.4777 | 7016.5135 | 53 | 114 | -5.1 | AQNNQRNFLAGEEDNVISQIQRPVKEVAFPGSAEEVDRLLKNQRQSHFANAQPQQKDEGSQK | 4523.76 | C298 H477 N96 O101 S0 |
| Y Fragment | 7130.5363 | 7130.5564 | 52 | 114 | -2.8 | NAQNNQRNFLAGEEDNVISQIQRPVKEVAFPGSAEEVDRLLKNQRQSHFANAQPQQKDEGSQK | 12188.5 | C302 H483 N98 O103 S0 |
| Y Fragment | 7447.7018 | 7447.7304 | 49 | 114 | -3.8 | FGINAQNNQRNFLAGEEDNVISQIQRPVKEVAFPGSAEEVDRLLKNQRQSHFANAQPQQKDEGSQK | 3634.84 | C319 H506 N101 O106 S0 |
| Y Fragment | 7617.8117 | 7617.8359 | 47 | 114 | -3.2 | LGFGINAQNNQRNFLAGEEDNVISQIQRPVKEVAFPGSAEEVDRLLKNQRQSHFANAQPQQKDEGSQK | 2395.94 | C327 H520 N103 O108 S0 |
| BY Int Fragment | 502.1763 | 502.1780 | 62 | 66 | -3.4 | AGEED | 1649.12 | C19 H28 N5 O11 S0 |
| BY Int Fragment | 503.2598 | 503.2613 | 49 | 53 | -2.9 | FGINA | 653.75 | C24 H35 N6 O6 S0 |
| BY Int Fragment | 503.2598 | 503.2613 | 59 | 63 | -2.9 | NFLAG | 653.75 | C24 H35 N6 O6 S0 |
| BY Int Fragment | 613.3397 | 613.3416 | 70 | 74 | -3.2 | SQIQR | 1740.5 | C25 H45 N10 O8 S0 |
| BY Int Fragment | 616.2191 | 616.2209 | 62 | 67 | -3.0 | AGEEDN | 1754.52 | C23 H34 N7 O13 S0 |
| BY Int Fragment | 624.3696 | 624.3715 | 75 | 80 | -3.1 | PVKEVA | 1206.09 | C29 H50 N7 O8 S0 |
| BY Int Fragment | 629.3056 | 629.3042 | 100 | 105 | 2.2 | FANAQP | 1684.07 | C29 H41 N8 O8 S0 |
| BY Int Fragment | 715.2870 | 715.2893 | 62 | 68 | -3.3 | AGEEDNV | 1702.09 | C28 H43 N8 O14 S0 |
| BY Int Fragment | 726.4235 | 726.4257 | 69 | 74 | -3.1 | ISQIQR | 2091.46 | C31 H56 N11 O9 S0 |
| BY Int Fragment | 771.4377 | 771.4400 | 75 | 81 | -3.0 | PVKEVAF | 3718.5 | C38 H59 N8 O9 S0 |
| BY Int Fragment | 771.4377 | 771.4400 | 76 | 82 | -3.0 | VKEVAFP | 3718.5 | C38 H59 N8 O9 S0 |
| BY Int Fragment | 837.4220 | 837.4254 | 30 | 37 | -4.0 | IPASHPFS | 1684.03 | C40 H57 N10 O10 S0 |
| BY Int Fragment | 837.4220 | 837.4254 | 31 | 38 | -4.0 | PASHPFSI | 1684.03 | C40 H57 N10 O10 S0 |
| BY Int Fragment | 887.4455 | 887.4482 | 55 | 61 | -3.1 | NNQRNFL | 1418.8 | C38 H59 N14 O11 S0 |
| BY Int Fragment | 922.5442 | 922.5469 | 68 | 75 | -2.9 | VISQIQRP | 1771.53 | C41 H72 N13 O11 S0 |
| BY Int Fragment | 922.5442 | 922.5469 | 69 | 76 | -2.9 | ISQIQRPV | 1771.53 | C41 H72 N13 O11 S0 |
| BY Int Fragment | 927.5384 | 927.5411 | 74 | 81 | -2.9 | RPVKEVAF | 3351.63 | C44 H71 N12 O10 S0 |
| BY Int Fragment | 1022.5015 | 1022.5054 | 31 | 40 | -3.8 | PASHPFSINA | 4315.63 | C47 H68 N13 O13 S0 |
| BY Int Fragment | 1048.5259 | 1048.5310 | 37 | 46 | -4.8 | SINASSDLFL | 948.1 | C47 H74 N11 O16 S0 |
| BY Int Fragment | 1055.5955 | 1055.5996 | 73 | 81 | -3.9 | QRPVKEVAF | 4927.4 | C49 H79 N14 O12 S0 |
| BY Int Fragment | 1109.5318 | 1109.5374 | 31 | 41 | -5.1 | PASHPFSINAS | 1207.66 | C50 H73 N14 O15 S0 |
| BY Int Fragment | 1200.5815 | 1200.5868 | 48 | 58 | -4.5 | GFGINAQNNQR | 6114.45 | C50 H78 N19 O16 S0 |
| BY Int Fragment | 1200.5815 | 1200.5868 | 51 | 60 | -4.5 | INAQNNQRNF | 6114.45 | C50 H78 N19 O16 S0 |
| BY Int Fragment | 1200.5815 | 1200.5868 | 52 | 61 | -4.5 | NAQNNQRNFL | 6114.45 | C50 H78 N19 O16 S0 |
| BY Int Fragment | 1236.7010 | 1236.7059 | 70 | 80 | -4.0 | SQIQRPVKEVA | 912.65 | C54 H94 N17 O16 S0 |
| BY Int Fragment | 1257.6017 | 1257.6083 | 49 | 59 | -5.3 | FGINAQNNQRN | 5020 | C52 H81 N20 O17 S0 |
| BY Int Fragment | 1257.6017 | 1257.6083 | 50 | 60 | -5.3 | GINAQNNQRNF | 5020 | C52 H81 N20 O17 S0 |
| BY Int Fragment | 1271.6213 | 1271.6240 | 52 | 62 | -2.1 | NAQNNQRNFLA | 3243.6 | C53 H83 N20 O17 S0 |
| BY Int Fragment | 1278.7495 | 1278.7528 | 68 | 78 | -2.6 | VISQIQRPVKE | 383.93 | C57 H100 N17 O16 S0 |
| BY Int Fragment | 1278.7495 | 1278.7528 | 69 | 79 | -2.6 | ISQIQRPVKEV | 383.93 | C57 H100 N17 O16 S0 |
| BY Int Fragment | 1311.5920 | 1311.5924 | 102 | 113 | -0.3 | NAQPQQKDEGSQ | 7793.43 | C52 H83 N18 O22 S0 |
| BY Int Fragment | 1328.6423 | 1328.6454 | 52 | 63 | -2.4 | NAQNNQRNFLAG | 330.42 | C55 H86 N21 O18 S0 |
| BY Int Fragment | 1370.6849 | 1370.6924 | 50 | 61 | -5.5 | GINAQNNQRNFL | 1284.02 | C58 H92 N21 O18 S0 |
| BY Int Fragment | 1383.7682 | 1383.7743 | 70 | 81 | -4.4 | SQIQRPVKEVAF | 3393.46 | C63 H103 N18 O17 S0 |
| BY Int Fragment | 1404.6729 | 1404.6767 | 49 | 60 | -2.7 | FGINAQNNQRNF | 446.03 | C61 H90 N21 O18 S0 |
| BY Int Fragment | 1424.6752 | 1424.6805 | 30 | 43 | -3.7 | IPASHPFSINASSD | 1522.37 | C63 H94 N17 O21 S0 |
| BY Int Fragment | 1424.6752 | 1424.6805 | 31 | 44 | -3.7 | PASHPFSINASSDL | 1522.37 | C63 H94 N17 O21 S0 |
| BY Int Fragment | 1441.7248 | 1441.7295 | 50 | 62 | -3.3 | GINAQNNQRNFLA | 370.49 | C61 H97 N22 O19 S0 |
| BY Int Fragment | 1441.7248 | 1441.7295 | 51 | 63 | -3.3 | INAQNNQRNFLAG | 370.49 | C61 H97 N22 O19 S0 |
| BY Int Fragment | 1461.6942 | 1461.6982 | 48 | 60 | -2.7 | GFGINAQNNQRNF | 1592.1 | C63 H93 N22 O19 S0 |
| BY Int Fragment | 1496.8535 | 1496.8584 | 69 | 81 | -3.3 | ISQIQRPVKEVAF | 2368.51 | C69 H114 N19 O18 S0 |
| BY Int Fragment | 1517.7552 | 1517.7608 | 49 | 61 | -3.7 | FGINAQNNQRNFL | 366.36 | C67 H101 N22 O19 S0 |
| BY Int Fragment | 1763.8613 | 1763.8684 | 93 | 107 | -4.1 | KNQRQSHFANAQPQQ | 294.58 | C74 H115 N28 O23 S0 |
| BY Int Fragment | 1763.8613 | 1763.8684 | 94 | 108 | -4.1 | NQRQSHFANAQPQQK | 294.58 | C74 H115 N28 O23 S0 |
| BY Int Fragment | 1914.8638 | 1914.8689 | 52 | 68 | -2.7 | NAQNNQRNFLAGEEDNV | 1243.41 | C78 H120 N27 O30 S0 |
| BY Int Fragment | 2189.9859 | 2189.9959 | 48 | 67 | -4.6 | GFGINAQNNQRNFLAGEEDN | 1132.16 | C92 H137 N30 O33 S0 |
| BY Int Fragment | 2378.1854 | 2378.1960 | 82 | 102 | -4.5 | PGSAEEVDRLLKNQRQSHFAN | 2713.47 | C101 H161 N34 O33 S0 |
| BY Int Fragment | 2455.1947 | 2455.2073 | 54 | 74 | -5.1 | QNNQRNFLAGEEDNVISQIQR | 745.61 | C102 H164 N35 O36 S0 |
| BY Int Fragment | 2577.2838 | 2577.2917 | 82 | 104 | -3.1 | PGSAEEVDRLLKNQRQSHFANAQ | 2443.12 | C109 H174 N37 O36 S0 |
| BY Int Fragment | 2577.2838 | 2577.2917 | 83 | 105 | -3.1 | GSAEEVDRLLKNQRQSHFANAQP | 2443.12 | C109 H174 N37 O36 S0 |
| BY Int Fragment | 2795.3787 | 2795.3972 | 80 | 104 | -6.6 | AFPGSAEEVDRLLKNQRQSHFANAQ | 540.36 | C121 H188 N39 O38 S0 |
| BY Int Fragment | 3371.6374 | 3371.6350 | 4 | 33 | 0.7 | DEEEEEDRNVQVQRFQSKLSSGDVVVIPAS | 474.79 | C142 H228 N41 O54 S0 |
| BY Int Fragment | 3766.8538 | 3766.8784 | 55 | 88 | -6.5 | NNQRNFLAGEEDNVISQIQRPVKEVAFPGSAEEV | 1228.79 | C163 H257 N48 O55 S0 |
| BY Int Fragment | 3960.0447 | 3960.0587 | 70 | 104 | -3.5 | SQIQRPVKEVAFPGSAEEVDRLLKNQRQSHFANAQ | 1049.86 | C172 H276 N55 O53 S0 |
| BY Int Fragment | 4073.1302 | 4073.1428 | 69 | 104 | -3.1 | ISQIQRPVKEVAFPGSAEEVDRLLKNQRQSHFANAQ | 1124.55 | C178 H287 N56 O54 S0 |
| BY Int Fragment | 4149.0109 | 4149.0497 | 77 | 113 | -9.3 | KEVAFPGSAEEVDRLLKNQRQSHFANAQPQQKDEGSQ | 661.8 | C177 H279 N56 O60 S0 |
| BY Int Fragment | 4166.0396 | 4166.0578 | 36 | 73 | -4.4 | FSINASSDLFLLGFGINAQNNQRNFLAGEEDNVISQIQ | 499.99 | C184 H282 N51 O60 S0 |
| BY Int Fragment | 4682.2831 | 4682.3234 | 47 | 89 | -8.6 | LGFGINAQNNQRNFLAGEEDNVISQIQRPVKEVAFPGSAEEVD | 1066.4 | C204 H318 N59 O68 S0 |
| BY Int Fragment | 4716.3781 | 4716.3877 | 63 | 104 | -2.0 | GEEDNVISQIQRPVKEVAFPGSAEEVDRLLKNQRQSHFANAQ | 1323.67 | C203 H324 N63 O67 S0 |
| BY Int Fragment | 4787.4118 | 4787.4248 | 62 | 104 | -2.7 | AGEEDNVISQIQRPVKEVAFPGSAEEVDRLLKNQRQSHFANAQ | 2198.2 | C206 H329 N64 O68 S0 |
| BY Int Fragment | 5086.5909 | 5086.6286 | 16 | 62 | -7.4 | QRFQSKLSSGDVVVIPASHPFSINASSDLFLLGFGINAQNNQRNFLA | 2472.54 | C229 H354 N65 O67 S0 |
| BY Int Fragment | 5099.4608 | 5099.4869 | 3 | 48 | -5.1 | EDEEEEEDRNVQVQRFQSKLSSGDVVVIPASHPFSINASSDLFLLG | 1807.72 | C222 H345 N60 O78 S0 |
| BY Int Fragment | 5303.5454 | 5303.5768 | 3 | 50 | -5.9 | EDEEEEEDRNVQVQRFQSKLSSGDVVVIPASHPFSINASSDLFLLGFG | 3417.01 | C233 H357 N62 O80 S0 |
| BY Int Fragment | 5801.8805 | 5801.9244 | 54 | 104 | -7.6 | QNNQRNFLAGEEDNVISQIQRPVKEVAFPGSAEEVDRLLKNQRQSHFANAQ | 1157.09 | C249 H395 N80 O81 S0 |
| BY Int Fragment | 5801.8943 | 5801.9244 | 54 | 104 | -5.2 | QNNQRNFLAGEEDNVISQIQRPVKEVAFPGSAEEVDRLLKNQRQSHFANAQ | 1679.07 | C249 H395 N80 O81 S0 |
| BY Int Fragment | 5841.8912 | 5841.8743 | 5 | 57 | 2.9 | EEEEEDRNVQVQRFQSKLSSGDVVVIPASHPFSINASSDLFLLGFGINAQNNQ | 3771.35 | C255 H395 N72 O86 S0 |
| BY Int Fragment | 5912.9599 | 5912.9928 | 56 | 107 | -5.6 | NQRNFLAGEEDNVISQIQRPVKEVAFPGSAEEVDRLLKNQRQSHFANAQPQQ | 4772.56 | C255 H404 N81 O82 S0 |
| BY Int Fragment | 6027.0232 | 6027.0357 | 54 | 106 | -2.1 | QNNQRNFLAGEEDNVISQIQRPVKEVAFPGSAEEVDRLLKNQRQSHFANAQPQ | 828.38 | C259 H410 N83 O84 S0 |
| BY Int Fragment | 6027.0232 | 6027.0357 | 55 | 107 | -2.1 | NNQRNFLAGEEDNVISQIQRPVKEVAFPGSAEEVDRLLKNQRQSHFANAQPQQ | 828.38 | C259 H410 N83 O84 S0 |
| BY Int Fragment | 6043.0400 | 6043.0558 | 58 | 110 | -2.6 | RNFLAGEEDNVISQIQRPVKEVAFPGSAEEVDRLLKNQRQSHFANAQPQQKDE | 5857.12 | C261 H414 N81 O85 S0 |
| BY Int Fragment** | 3303.5934 | 3303.6253 | 81 | 109 | -9.7 | FPGSAEEVDRLLKNQRQSHFANAQPQQKD | 387.96 | C143 H223 N46 O46 S0 |
| BY Int Fragment** | 5212.5600 | 5212.5974 | 41 | 88 | -7.2 | SSDLFLLGFGINAQNNQRNFLAGEEDNVISQIQRPVKEVAFPGSAEEV | 524.38 | C231 H359 N64 O75 S0 |
| BY Int Fragment** | 887.4455 | 887.4509 | 40 | 48 | -6.1 | ASSDLFLLG | 1418.8 | C42 H66 N9 O13 S0 |
| BY Int Fragment** | 4612.2762 | 4612.3039 | 73 | 113 | -6.0 | QRPVKEVAFPGSAEEVDRLLKNQRQSHFANAQPQQKDEGSQ | 281.91 | C198 H315 N64 O65 S0 |
| BY Int Fragment** | 2378.1854 | 2378.1986 | 64 | 85 | -5.6 | EEDNVISQIQRPVKEVAFPGSA | 2713.47 | C105 H168 N29 O35 S0 |
| BY Int Fragment** | 2378.1854 | 2378.1986 | 65 | 86 | -5.6 | EDNVISQIQRPVKEVAFPGSAE | 2713.47 | C105 H168 N29 O35 S0 |
| BY Int Fragment** | 2378.1854 | 2378.1986 | 66 | 87 | -5.6 | DNVISQIQRPVKEVAFPGSAEE | 2713.47 | C105 H168 N29 O35 S0 |
| BY Int Fragment** | 1517.7552 | 1517.7634 | 40 | 54 | -5.4 | ASSDLFLLGFGINAQ | 366.36 | C71 H108 N17 O21 S0 |
| BY Int Fragment** | 4167.0387 | 4167.0570 | 28 | 66 | -4.4 | VVIPASHPFSINASSDLFLLGFGINAQNNQRNFLAGEED | 3747.19 | C188 H284 N51 O58 S0 |
| BY Int Fragment** | 627.2235 | 627.2256 | 63 | 68 | -3.4 | GEEDNV | 1258.03 | C25 H38 N7 O13 S0 |
| BY Int Fragment** | 5429.7856 | 5429.8029 | 28 | 77 | -3.2 | VVIPASHPFSINASSDLFLLGFGINAQNNQRNFLAGEEDNVISQIQRPVK | 3512.29 | C244 H382 N69 O73 S0 |
| BY Int Fragment** | 4953.4025 | 4953.4177 | 4 | 48 | -3.1 | DEEEEEDRNVQVQRFQSKLSSGDVVVIPASHPFSINASSDLFLLG | 3356.37 | C217 H338 N59 O75 S0 |
| BY Int Fragment** | 1183.5567 | 1183.5602 | 48 | 58 | -3.0 | GFGINAQNNQR | 529.07 | C50 H78 N19 O16 S0 |
| BY Int Fragment** | 1183.5567 | 1183.5602 | 51 | 60 | -3.0 | INAQNNQRNF | 529.07 | C50 H78 N19 O16 S0 |
| BY Int Fragment** | 1183.5567 | 1183.5602 | 52 | 61 | -3.0 | NAQNNQRNFL | 529.07 | C50 H78 N19 O16 S0 |
| Y Fragment** | 5412.7636 | 5412.7795 | 67 | 114 | -2.9 | NVISQIQRPVKEVAFPGSAEEVDRLLKNQRQSHFANAQPQQKDEGSQK | 363.88 | C233 H379 N74 O76 S0 |
| BY Int Fragment** | 671.3128 | 671.3147 | 48 | 54 | -2.9 | GFGINAQ | 846.96 | C31 H46 N9 O9 S0 |
| Y Fragment** | 7113.5126 | 7113.5298 | 52 | 114 | -2.4 | NAQNNQRNFLAGEEDNVISQIQRPVKEVAFPGSAEEVDRLLKNQRQSHFANAQPQQKDEGSQK | 3007.91 | C302 H483 N98 O103 S0 |
| BY Int Fragment** | 1444.6684 | 1444.6716 | 48 | 60 | -2.2 | GFGINAQNNQRNF | 1509.6 | C63 H93 N22 O19 S0 |
| BY Int Fragment** | 1897.8383 | 1897.8423 | 52 | 68 | -2.1 | NAQNNQRNFLAGEEDNV | 759.68 | C78 H120 N27 O30 S0 |
| Y Fragment** | 6027.0232 | 6027.0343 | 61 | 114 | -1.8 | LAGEEDNVISQIQRPVKEVAFPGSAEEVDRLLKNQRQSHFANAQPQQKDEGSQK | 828.38 | C258 H417 N80 O88 S0 |
| Y Fragment** | 5086.5751 | 5086.5841 | 70 | 114 | -1.8 | SQIQRPVKEVAFPGSAEEVDRLLKNQRQSHFANAQPQQKDEGSQK | 336.39 | C218 H353 N70 O72 S0 |
| BY Int Fragment** | 3720.8317 | 3720.8364 | 53 | 86 | -1.3 | AQNNQRNFLAGEEDNVISQIQRPVKEVAFPGSAE | 31255.11 | C161 H254 N49 O54 S0 |
| Y Fragment** | 3921.9178 | 3921.9226 | 80 | 114 | -1.2 | AFPGSAEEVDRLLKNQRQSHFANAQPQQKDEGSQK | 5690.59 | C167 H265 N54 O57 S0 |
| BY Int Fragment** | 5382.6799 | 5382.6850 | 65 | 112 | -0.9 | EDNVISQIQRPVKEVAFPGSAEEVDRLLKNQRQSHFANAQPQQKDEGS | 421.56 | C231 H369 N72 O78 S0 |
| Y Fragment** | 1255.5905 | 1255.5913 | 104 | 114 | -0.6 | QPQQKDEGSQK | 338.17 | C51 H86 N17 O21 S0 |
| Y Fragment** | 3703.8148 | 3703.8171 | 82 | 114 | -0.6 | PGSAEEVDRLLKNQRQSHFANAQPQQKDEGSQK | 4879.91 | C155 H251 N52 O55 S0 |
| BY Int Fragment** | 1557.7550 | 1557.7557 | 47 | 60 | -0.4 | LGFGINAQNNQRNF | 1587.07 | C69 H104 N23 O20 S0 |
| BY Int Fragment** | 1557.7550 | 1557.7557 | 48 | 61 | -0.4 | GFGINAQNNQRNFL | 1587.07 | C69 H104 N23 O20 S0 |
| BY Int Fragment** | 3279.6496 | 3279.6505 | 53 | 81 | -0.3 | AQNNQRNFLAGEEDNVISQIQRPVKEVAF | 1518.33 | C143 H227 N44 O46 S0 |
| B Fragment** | 4593.2485 | 4593.2492 | 1 | 41 | -0.2 | KKEDEEEEEDRNVQVQRFQSKLSSGDVVVIPASHPFSINAS | 1376.77 | C198 H314 N57 O70 S0 |
| B Fragment** | 3371.6374 | 3371.6349 | 1 | 29 | 0.7 | KKEDEEEEEDRNVQVQRFQSKLSSGDVVV | 474.79 | C142 H231 N42 O54 S0 |
| BY Int Fragment** | 3529.7391 | 3529.7346 | 42 | 73 | 1.3 | SDLFLLGFGINAQNNQRNFLAGEEDNVISQIQ | 20762.89 | C156 H241 N44 O51 S0 |
| BY Int Fragment** | 1294.5675 | 1294.5658 | 102 | 113 | 1.3 | NAQPQQKDEGSQ | 4276.9 | C52 H83 N18 O22 S0 |
| Y Fragment** | 5086.5909 | 5086.5841 | 70 | 114 | 1.3 | SQIQRPVKEVAFPGSAEEVDRLLKNQRQSHFANAQPQQKDEGSQK | 2472.54 | C218 H353 N70 O72 S0 |
| BY Int Fragment** | 1241.5815 | 1241.5796 | 79 | 90 | 1.5 | VAFPGSAEEVDR | 292.89 | C55 H84 N15 O19 S0 |
| BY Int Fragment** | 6798.3675 | 6798.3459 | 2 | 62 | 3.2 | KEDEEEEEDRNVQVQRFQSKLSSGDVVVIPASHPFSINASSDLFLLGFGINAQNNQRNFLA | 593.82 | C298 H462 N85 O99 S0 |
| BY Int Fragment** | 5859.9128 | 5859.8889 | 36 | 89 | 4.1 | FSINASSDLFLLGFGINAQNNQRNFLAGEEDNVISQIQRPVKEVAFPGSAEEVD | 14991.2 | C260 H400 N71 O85 S0 |
| BY Int Fragment** | 5824.8796 | 5824.8477 | 5 | 57 | 5.5 | EEEEEDRNVQVQRFQSKLSSGDVVVIPASHPFSINASSDLFLLGFGINAQNNQ | 326.24 | C255 H395 N72 O86 S0 |
| BY Int Fragment** | 5016.5636 | 5016.5351 | 59 | 103 | 5.7 | NFLAGEEDNVISQIQRPVKEVAFPGSAEEVDRLLKNQRQSHFANA | 1712.18 | C220 H347 N66 O70 S0 |
| BY Int Fragment** | 4348.1481 | 4348.1228 | 50 | 89 | 5.8 | GINAQNNQRNFLAGEEDNVISQIQRPVKEVAFPGSAEEVD | 3616.68 | C187 H295 N56 O65 S0 |
| BY Int Fragment** | 5103.6052 | 5103.5711 | 29 | 75 | 6.7 | VIPASHPFSINASSDLFLLGFGINAQNNQRNFLAGEEDNVISQIQRP | 17681.47 | C228 H352 N65 O70 S0 |
| BY Int Fragment** | 5103.6052 | 5103.5711 | 30 | 76 | 6.7 | IPASHPFSINASSDLFLLGFGINAQNNQRNFLAGEEDNVISQIQRPV | 17681.47 | C228 H352 N65 O70 S0 |
| BY Int Fragment** | 1685.8295 | 1685.8142 | 48 | 63 | 9.0 | GFGINAQNNQRNFLAG | 1383.07 | C74 H112 N25 O22 S0 |
| Y Fragment* | 4149.0109 | 4149.0496 | 78 | 114 | -9.3 | EVAFPGSAEEVDRLLKNQRQSHFANAQPQQKDEGSQK | 661.8 | C177 H281 N56 O61 S0 |
| BY Int Fragment* | 3218.5202 | 3218.5501 | 37 | 66 | -9.3 | SINASSDLFLLGFGINAQNNQRNFLAGEED | 2213.11 | C141 H215 N40 O48 S0 |
| BY Int Fragment* | 1067.4906 | 1067.5003 | 63 | 72 | -9.2 | GEEDNVISQI | 1393.85 | C45 H73 N12 O19 S0 |
| BY Int Fragment* | 3317.5887 | 3317.6185 | 40 | 70 | -9.0 | ASSDLFLLGFGINAQNNQRNFLAGEEDNVIS | 9654.54 | C146 H224 N41 O49 S0 |
| BY Int Fragment* | 7503.7231 | 7503.7857 | 8 | 75 | -8.3 | EEDRNVQVQRFQSKLSSGDVVVIPASHPFSINASSDLFLLGFGINAQNNQRNFLAGEEDNVISQIQRP | 6484.24 | C329 H516 N97 O106 S0 |
| BY Int Fragment* | 5099.4608 | 5099.5021 | 4 | 49 | -8.1 | DEEEEEDRNVQVQRFQSKLSSGDVVVIPASHPFSINASSDLFLLGF | 1807.72 | C226 H347 N60 O76 S0 |
| BY Int Fragment* | 4166.0396 | 4166.0730 | 28 | 66 | -8.0 | VVIPASHPFSINASSDLFLLGFGINAQNNQRNFLAGEED | 499.99 | C188 H284 N51 O58 S0 |
| BY Int Fragment* | 4480.1967 | 4480.2320 | 29 | 70 | -7.9 | VIPASHPFSINASSDLFLLGFGINAQNNQRNFLAGEEDNVIS | 9462.98 | C201 H306 N55 O63 S0 |
| BY Int Fragment* | 5156.4891 | 5156.5236 | 4 | 50 | -6.7 | DEEEEEDRNVQVQRFQSKLSSGDVVVIPASHPFSINASSDLFLLGFG | 6682.34 | C228 H350 N61 O77 S0 |
| Y Fragment* | 5841.8912 | 5841.9291 | 63 | 114 | -6.5 | GEEDNVISQIQRPVKEVAFPGSAEEVDRLLKNQRQSHFANAQPQQKDEGSQK | 3771.35 | C249 H401 N78 O86 S0 |
| B Fragment* | 4794.2963 | 4794.3241 | 1 | 43 | -5.8 | KKEDEEEEEDRNVQVQRFQSKLSSGDVVVIPASHPFSINASSD | 2532.95 | C205 H324 N59 O75 S0 |
| Y Fragment* | 7486.7020 | 7486.7412 | 48 | 114 | -5.2 | GFGINAQNNQRNFLAGEEDNVISQIQRPVKEVAFPGSAEEVDRLLKNQRQSHFANAQPQQKDEGSQK | 1896.01 | C321 H509 N102 O107 S0 |
| Y Fragment* | 3849.8816 | 3849.9015 | 81 | 114 | -5.2 | FPGSAEEVDRLLKNQRQSHFANAQPQQKDEGSQK | 504.9 | C164 H260 N53 O56 S0 |
| Y Fragment* | 6927.4320 | 6927.4658 | 54 | 114 | -4.9 | QNNQRNFLAGEEDNVISQIQRPVKEVAFPGSAEEVDRLLKNQRQSHFANAQPQQKDEGSQK | 1584.69 | C295 H472 N95 O100 S0 |
| B Fragment* | 4320.1352 | 4320.1531 | 1 | 38 | -4.1 | KKEDEEEEEDRNVQVQRFQSKLSSGDVVVIPASHPFSI | 1933.67 | C188 H298 N53 O65 S0 |
| Y Fragment* | 5198.6647 | 5198.6842 | 69 | 114 | -3.8 | ISQIQRPVKEVAFPGSAEEVDRLLKNQRQSHFANAQPQQKDEGSQK | 1961.97 | C224 H364 N71 O73 S0 |
| BY Int Fragment* | 2692.3494 | 2692.3590 | 78 | 101 | -3.6 | EVAFPGSAEEVDRLLKNQRQSHFA | 298.06 | C119 H185 N36 O37 S0 |
| B Fragment* | 3271.5718 | 3271.5825 | 1 | 28 | -3.3 | KKEDEEEEEDRNVQVQRFQSKLSSGDVV | 684.37 | C137 H222 N41 O53 S0 |
| Y Fragment* | 5655.8486 | 5655.8651 | 65 | 114 | -2.9 | EDNVISQIQRPVKEVAFPGSAEEVDRLLKNQRQSHFANAQPQQKDEGSQK | 1714.45 | C242 H391 N76 O82 S0 |
| Y Fragment* | 530.2555 | 530.2569 | 110 | 114 | -2.6 | EGSQK | 2554.72 | C21 H38 N7 O10 S0 |
| BY Int Fragment* | 2844.4026 | 2844.4063 | 45 | 70 | -1.3 | FLLGFGINAQNNQRNFLAGEEDNVIS | 647.79 | C127 H193 N36 O40 S0 |
| Y Fragment* | 5912.9599 | 5912.9663 | 62 | 114 | -1.1 | AGEEDNVISQIQRPVKEVAFPGSAEEVDRLLKNQRQSHFANAQPQQKDEGSQK | 4772.56 | C252 H406 N79 O87 S0 |
| BY Int Fragment* | 819.4144 | 819.4148 | 30 | 37 | -0.5 | IPASHPFS | 657.18 | C40 H57 N10 O10 S0 |
| BY Int Fragment* | 819.4144 | 819.4148 | 31 | 38 | -0.5 | PASHPFSI | 657.18 | C40 H57 N10 O10 S0 |
| BY Int Fragment* | 4637.2826 | 4637.2808 | 31 | 73 | 0.4 | PASHPFSINASSDLFLLGFGINAQNNQRNFLAGEEDNVISQIQ | 3297.79 | C206 H313 N58 O66 S0 |
| BY Int Fragment* | 4551.2360 | 4551.2327 | 25 | 67 | 0.7 | GDVVVIPASHPFSINASSDLFLLGFGINAQNNQRNFLAGEEDN | 16596.31 | C203 H307 N56 O65 S0 |
| BY Int Fragment* | 2316.1595 | 2316.1520 | 45 | 65 | 3.2 | FLLGFGINAQNNQRNFLAGEE | 1266.65 | C105 H157 N30 O31 S0 |
| BY Int Fragment* | 1358.7154 | 1358.7103 | 42 | 54 | 3.8 | SDLFLLGFGINAQ | 1405.24 | C65 H98 N15 O18 S0 |
| BY Int Fragment* | 1022.5015 | 1022.4915 | 95 | 103 | 9.8 | QRQSHFANA | 4315.63 | C44 H66 N17 O13 S0 |
| BY Int Fragment* | 1022.5015 | 1022.4915 | 96 | 104 | 9.8 | RQSHFANAQ | 4315.63 | C44 H66 N17 O13 S0 |

**Table S13**. Sample output of ClipsMS related to the CID mass spectrum (Fig.S33B) of the at m/z 887.6879, [M+16H]^16+^. The Table includes the fragment type, observed mass, theoretical mass, start amino acid, end amino acid, error (ppm), sequence, intensity, and molecular formula. The amino acid positions refer to the sequence of gamma-chain of vicilin A0A1S2XQR4.

| **Frag Type** | **Localized Mod** | **Observed Mass** | **Theoretical Mass** | **Start AA** | **End AA** | **Error** | **Sequence** | **Intensity** | **Formula** |
| --- | --- | --- | --- | --- | --- | --- | --- | --- | --- |
| B Fragment | 0 | 3289.5891 | 3289.5931 | 1 | 28 | -1.2 | KKEDEEEEEDRNVQVQRFQSKLSSGDVV | 267.33 | C137 H222 N41 O53 S0 |
| B Fragment | 0 | 3501.7403 | 3501.7456 | 1 | 30 | -1.5 | KKEDEEEEEDRNVQVQRFQSKLSSGDVVVI | 1078.77 | C148 H242 N43 O55 S0 |
| B Fragment | 0 | 7487.7143 | 7487.6440 | 1 | 67 | 9.4 | KKEDEEEEEDRNVQVQRFQSKLSSGDVVVIPASHPFSINASSDLFLLGFGINAQNNQRNFLAGEEDN | 1086.42 | C324 H502 N93 O112 S0 |
| Y Fragment | 0 | 3720.8326 | 3720.8437 | 82 | 114 | -3.0 | PGSAEEVDRLLKNQRQSHFANAQPQQKDEGSQK | 1774.64 | C155 H251 N52 O55 S0 |
| Y Fragment | 0 | 5216.6835 | 5216.6948 | 69 | 114 | -2.2 | ISQIQRPVKEVAFPGSAEEVDRLLKNQRQSHFANAQPQQKDEGSQK | 1159.18 | C224 H364 N71 O73 S0 |
| Y Fragment | 0 | 7130.5804 | 7130.5564 | 52 | 114 | 3.4 | NAQNNQRNFLAGEEDNVISQIQRPVKEVAFPGSAEEVDRLLKNQRQSHFANAQPQQKDEGSQK | 1193.5 | C302 H483 N98 O103 S0 |
| Y Fragment | 0 | 7243.6279 | 7243.6405 | 51 | 114 | -1.7 | INAQNNQRNFLAGEEDNVISQIQRPVKEVAFPGSAEEVDRLLKNQRQSHFANAQPQQKDEGSQK | 2392.81 | C308 H494 N99 O104 S0 |
| Y Fragment | 0 | 7300.6449 | 7300.6620 | 50 | 114 | -2.3 | GINAQNNQRNFLAGEEDNVISQIQRPVKEVAFPGSAEEVDRLLKNQRQSHFANAQPQQKDEGSQK | 6908.78 | C310 H497 N100 O105 S0 |
| Y Fragment | 0 | 7447.7116 | 7447.7304 | 49 | 114 | -2.5 | FGINAQNNQRNFLAGEEDNVISQIQRPVKEVAFPGSAEEVDRLLKNQRQSHFANAQPQQKDEGSQK | 8845.72 | C319 H506 N101 O106 S0 |
| Y Fragment | 0 | 7504.7450 | 7504.7518 | 48 | 114 | -0.9 | GFGINAQNNQRNFLAGEEDNVISQIQRPVKEVAFPGSAEEVDRLLKNQRQSHFANAQPQQKDEGSQK | 31538.19 | C321 H509 N102 O107 S0 |
| Y Fragment | 0 | 7730.9007 | 7730.9200 | 46 | 114 | -2.5 | LLGFGINAQNNQRNFLAGEEDNVISQIQRPVKEVAFPGSAEEVDRLLKNQRQSHFANAQPQQKDEGSQK | 2206.11 | C333 H531 N104 O109 S0 |
| BY Int Fragment | 0 | 5415.6433 | 5415.6880 | 40 | 89 | -8.3 | ASSDLFLLGFGINAQNNQRNFLAGEEDNVISQIQRPVKEVAFPGSAEEVD | 3022.94 | C238 H369 N66 O79 S0 |
| BY Int Fragment | 0 | 5500.7280 | 5500.7520 | 41 | 90 | -4.4 | SSDLFLLGFGINAQNNQRNFLAGEEDNVISQIQRPVKEVAFPGSAEEVDR | 454.57 | C241 H376 N69 O79 S0 |
| BY Int Fragment | 0 | 5529.7357 | 5529.7310 | 39 | 89 | 0.9 | NASSDLFLLGFGINAQNNQRNFLAGEEDNVISQIQRPVKEVAFPGSAEEVD | 9928.82 | C242 H375 N68 O81 S0 |
| BY Int Fragment | 0 | 6042.0611 | 6042.0718 | 57 | 109 | -1.8 | QRNFLAGEEDNVISQIQRPVKEVAFPGSAEEVDRLLKNQRQSHFANAQPQQKD | 461.13 | C261 H415 N82 O84 S0 |
| BY Int Fragment* | 0 | 1855.9145 | 1855.9045 | 7 | 21 | 5.4 | EEEDRNVQVQRFQSK | 561.82 | C78 H125 N26 O28 S0 |
| BY Int Fragment* | 0 | 3218.5217 | 3218.5501 | 37 | 66 | -8.8 | SINASSDLFLLGFGINAQNNQRNFLAGEED | 1574.44 | C141 H215 N40 O48 S0 |
| BY Int Fragment* | 0 | 3317.5994 | 3317.6185 | 40 | 70 | -5.8 | ASSDLFLLGFGINAQNNQRNFLAGEEDNVIS | 3062.16 | C146 H224 N41 O49 S0 |
| B Fragment** | 0 | 3272.5356 | 3272.5665 | 1 | 28 | -9.5 | KKEDEEEEEDRNVQVQRFQSKLSSGDVV | 237 | C137 H222 N41 O53 S0 |
| B Fragment** | 0 | 4321.1696 | 4321.1371 | 1 | 38 | 7.5 | KKEDEEEEEDRNVQVQRFQSKLSSGDVVVIPASHPFSI | 777.67 | C188 H298 N53 O65 S0 |
| Y Fragment** | 0 | 7487.7143 | 7487.7252 | 48 | 114 | -1.5 | GFGINAQNNQRNFLAGEEDNVISQIQRPVKEVAFPGSAEEVDRLLKNQRQSHFANAQPQQKDEGSQK | 1086.42 | C321 H509 N102 O107 S0 |
| BY Int Fragment** | 0 | 1015.3518 | 1015.3486 | 4 | 11 | 3.1 | DEEEEEDR | 13920.65 | C39 H58 N11 O22 S0 |
| BY Int Fragment** | 0 | 3529.7450 | 3529.7346 | 42 | 73 | 2.9 | SDLFLLGFGINAQNNQRNFLAGEEDNVISQIQ | 6768.78 | C156 H241 N44 O51 S0 |
| BY Int Fragment** | 0 | 3720.8326 | 3720.8364 | 53 | 86 | -1.0 | AQNNQRNFLAGEEDNVISQIQRPVKEVAFPGSAE | 1774.64 | C161 H254 N49 O54 S0 |
| BY Int Fragment** | 0 | 7130.5804 | 7130.6260 | 34 | 97 | -6.4 | HPFSINASSDLFLLGFGINAQNNQRNFLAGEEDNVISQIQRPVKEVAFPGSAEEVDRLLKNQRQ | 1193.5 | C315 H494 N93 O98 S0 |
| BY Int Fragment** | 0 | 7504.7450 | 7504.7697 | 8 | 75 | -3.3 | EEDRNVQVQRFQSKLSSGDVVVIPASHPFSINASSDLFLLGFGINAQNNQRNFLAGEEDNVISQIQRP | 31538.19 | C329 H516 N97 O106 S0 |
| BY Int Fragment** | 0 | 7730.9007 | 7730.8916 | 32 | 101 | 1.2 | ASHPFSINASSDLFLLGFGINAQNNQRNFLAGEEDNVISQIQRPVKEVAFPGSAEEVDRLLKNQRQSHFA | 2206.11 | C342 H530 N101 O106 S0 |
| BY Int Fragment** | 0 | 7876.9739 | 7876.9607 | 36 | 106 | 1.7 | FSINASSDLFLLGFGINAQNNQRNFLAGEEDNVISQIQRPVKEVAFPGSAEEVDRLLKNQRQSHFANAQPQ | 813.16 | C347 H540 N103 O109 S0 |
| BY Int Fragment | 406.1588 | 3416.6612 | 3416.6758 | 36 | 63 | -4.3 | FSINASSDLFLLGFGINAQNNQRNFLAG | 6778.09 | C136 H205 N38 O40 S0 |
| BY Int Fragment | 406.1588 | 5586.7553 | 5586.7664 | 26 | 73 | -2.0 | DVVVIPASHPFSINASSDLFLLGFGINAQNNQRNFLAGEEDNVISQIQ | 3343.99 | C231 H356 N63 O73 S0 |
| B Fragment | 568.2116 | 9503.7200 | 9503.7067 | 1 | 80 | 1.4 | KKEDEEEEEDRNVQVQRFQSKLSSGDVVVIPASHPFSINASSDLFLLGFGINAQNNQRNFLAGEEDNVISQIQRPVKEVA | 3451.98 | C389 H615 N112 O130 S0 |
| BY Int Fragment | 1054.37 | 3218.5217 | 3218.5097 | 19 | 39 | 3.7 | QSKLSSGDVVVIPASHPFSIN | 1574.44 | C97 H155 N26 O30 S0 |
| BY Int Fragment | 1054.37 | 6381.0228 | 6381.0580 | 8 | 56 | -5.5 | EEDRNVQVQRFQSKLSSGDVVVIPASHPFSINASSDLFLLGFGINAQNN | 788.55 | C235 H366 N67 O75 S0 |
| BY Int Fragment | 1216.4228 | 10355.0081 | 10354.9397 | 3 | 85 | 6.6 | EDEEEEEDRNVQVQRFQSKLSSGDVVVIPASHPFSINASSDLFLLGFGINAQNNQRNFLAGEEDNVISQIQRPVKEVAFPGSA | 856.75 | C399 H620 N113 O134 S0 |
| BY Int Fragment | 1216.4228 | 10355.0081 | 10354.9397 | 4 | 86 | 6.6 | DEEEEEDRNVQVQRFQSKLSSGDVVVIPASHPFSINASSDLFLLGFGINAQNNQRNFLAGEEDNVISQIQRPVKEVAFPGSAE | 856.75 | C399 H620 N113 O134 S0 |
| BY Int Fragment | 1378.4756 | 10517.0112 | 10516.9925 | 3 | 85 | 1.8 | EDEEEEEDRNVQVQRFQSKLSSGDVVVIPASHPFSINASSDLFLLGFGINAQNNQRNFLAGEEDNVISQIQRPVKEVAFPGSA | 1284.37 | C399 H620 N113 O134 S0 |
| BY Int Fragment | 1378.4756 | 10517.0112 | 10516.9925 | 4 | 86 | 1.8 | DEEEEEDRNVQVQRFQSKLSSGDVVVIPASHPFSINASSDLFLLGFGINAQNNQRNFLAGEEDNVISQIQRPVKEVAFPGSAE | 1284.37 | C399 H620 N113 O134 S0 |
| Y Fragment | 1378.4756 | 10680.1069 | 10680.1372 | 31 | 114 | -2.8 | PASHPFSINASSDLFLLGFGINAQNNQRNFLAGEEDNVISQIQRPVKEVAFPGSAEEVDRLLKNQRQSHFANAQPQQKDEGSQK | 1313.54 | C405 H633 N122 O131 S0 |

**Table S14**. Sample output of ClipsMS related to the HCD mass spectrum (Fig.S34) of the at m/z 946.2655, [M+15H]^15+^. The Table includes the fragment type, observed mass, theoretical mass, start amino acid, end amino acid, error (ppm), sequence, intensity, and molecular formula. The amino acid positions refer to the sequence of gamma-chain of vicilin A0A1S2XQR4.

| **Frag Type** | **Localized Mod** | **Observed Mass** | **Theoretical Mass** | **Start AA** | **End AA** | **Error** | **Sequence** | **Intensity** | **Formula** |
| --- | --- | --- | --- | --- | --- | --- | --- | --- | --- |
| B Fragment | 0 | 3190.5133 | 3190.5247 | 1 | 27 | -3.6 | KKEDEEEEEDRNVQVQRFQSKLSSGDV | 382.54 | C132 H213 N40 O52 S0 |
| B Fragment | 0 | 3289.5810 | 3289.5931 | 1 | 28 | -3.7 | KKEDEEEEEDRNVQVQRFQSKLSSGDVV | 916.99 | C137 H222 N41 O53 S0 |
| B Fragment | 0 | 3388.6510 | 3388.6615 | 1 | 29 | -3.1 | KKEDEEEEEDRNVQVQRFQSKLSSGDVVV | 1127.23 | C142 H231 N42 O54 S0 |
| B Fragment | 0 | 3501.7320 | 3501.7456 | 1 | 30 | -3.9 | KKEDEEEEEDRNVQVQRFQSKLSSGDVVVI | 3174.55 | C148 H242 N43 O55 S0 |
| B Fragment | 0 | 4523.2231 | 4523.2437 | 1 | 40 | -4.6 | KKEDEEEEEDRNVQVQRFQSKLSSGDVVVIPASHPFSINA | 948.33 | C195 H309 N56 O68 S0 |
| B Fragment | 0 | 4812.3184 | 4812.3347 | 1 | 43 | -3.4 | KKEDEEEEEDRNVQVQRFQSKLSSGDVVVIPASHPFSINASSD | 1238.73 | C205 H324 N59 O75 S0 |
| B Fragment | 0 | 4925.3945 | 4925.4188 | 1 | 44 | -4.9 | KKEDEEEEEDRNVQVQRFQSKLSSGDVVVIPASHPFSINASSDL | 663.92 | C211 H335 N60 O76 S0 |
| B Fragment | 0 | 5672.8559 | 5672.8508 | 1 | 51 | 0.9 | KKEDEEEEEDRNVQVQRFQSKLSSGDVVVIPASHPFSINASSDLFLLGFGI | 727.32 | C251 H392 N67 O83 S0 |
| B Fragment | 0 | 6943.4439 | 6943.4674 | 1 | 62 | -3.4 | KKEDEEEEEDRNVQVQRFQSKLSSGDVVVIPASHPFSINASSDLFLLGFGINAQNNQRNFLA | 356.71 | C304 H474 N87 O100 S0 |
| Y Fragment | 0 | 548.2658 | 548.2675 | 110 | 114 | -3.1 | EGSQK | 2487.1 | C21 H38 N7 O10 S0 |
| Y Fragment | 0 | 1144.5554 | 1144.5593 | 105 | 114 | -3.4 | PQQKDEGSQK | 5585.3 | C46 H78 N15 O19 S0 |
| Y Fragment | 0 | 1457.6928 | 1457.6979 | 102 | 114 | -3.5 | NAQPQQKDEGSQK | 343.04 | C58 H97 N20 O24 S0 |
| Y Fragment | 0 | 2936.5129 | 2936.5198 | 90 | 114 | -2.4 | RLLKNQRQSHFANAQPQQKDEGSQK | 704.24 | C123 H203 N44 O40 S0 |
| Y Fragment | 0 | 3720.8318 | 3720.8437 | 82 | 114 | -3.2 | PGSAEEVDRLLKNQRQSHFANAQPQQKDEGSQK | 5751.68 | C155 H251 N52 O55 S0 |
| Y Fragment | 0 | 3867.9071 | 3867.9121 | 81 | 114 | -1.3 | FPGSAEEVDRLLKNQRQSHFANAQPQQKDEGSQK | 1320.39 | C164 H260 N53 O56 S0 |
| Y Fragment | 0 | 3938.9159 | 3938.9492 | 80 | 114 | -8.5 | AFPGSAEEVDRLLKNQRQSHFANAQPQQKDEGSQK | 1280.65 | C167 H265 N54 O57 S0 |
| Y Fragment | 0 | 4775.4030 | 4775.4361 | 73 | 114 | -6.9 | QRPVKEVAFPGSAEEVDRLLKNQRQSHFANAQPQQKDEGSQK | 693.17 | C204 H329 N66 O67 S0 |
| Y Fragment | 0 | 4888.5021 | 4888.5201 | 72 | 114 | -3.7 | IQRPVKEVAFPGSAEEVDRLLKNQRQSHFANAQPQQKDEGSQK | 368.82 | C210 H340 N67 O68 S0 |
| Y Fragment | 0 | 5103.5925 | 5103.6107 | 70 | 114 | -3.6 | SQIQRPVKEVAFPGSAEEVDRLLKNQRQSHFANAQPQQKDEGSQK | 3524.23 | C218 H353 N70 O72 S0 |
| Y Fragment | 0 | 5216.6815 | 5216.6948 | 69 | 114 | -2.6 | ISQIQRPVKEVAFPGSAEEVDRLLKNQRQSHFANAQPQQKDEGSQK | 2679.17 | C224 H364 N71 O73 S0 |
| Y Fragment | 0 | 5544.8160 | 5544.8331 | 66 | 114 | -3.1 | DNVISQIQRPVKEVAFPGSAEEVDRLLKNQRQSHFANAQPQQKDEGSQK | 701.62 | C237 H384 N75 O79 S0 |
| Y Fragment | 0 | 5859.9219 | 5859.9397 | 63 | 114 | -3.0 | GEEDNVISQIQRPVKEVAFPGSAEEVDRLLKNQRQSHFANAQPQQKDEGSQK | 6098.01 | C249 H401 N78 O86 S0 |
| Y Fragment | 0 | 5930.9676 | 5930.9769 | 62 | 114 | -1.6 | AGEEDNVISQIQRPVKEVAFPGSAEEVDRLLKNQRQSHFANAQPQQKDEGSQK | 8411.64 | C252 H406 N79 O87 S0 |
| Y Fragment | 0 | 6817.3968 | 6817.4178 | 55 | 114 | -3.1 | NNQRNFLAGEEDNVISQIQRPVKEVAFPGSAEEVDRLLKNQRQSHFANAQPQQKDEGSQK | 1648.09 | C290 H464 N93 O98 S0 |
| Y Fragment | 0 | 6945.4518 | 6945.4764 | 54 | 114 | -3.5 | QNNQRNFLAGEEDNVISQIQRPVKEVAFPGSAEEVDRLLKNQRQSHFANAQPQQKDEGSQK | 2983.54 | C295 H472 N95 O100 S0 |
| Y Fragment | 0 | 7130.5362 | 7130.5564 | 52 | 114 | -2.8 | NAQNNQRNFLAGEEDNVISQIQRPVKEVAFPGSAEEVDRLLKNQRQSHFANAQPQQKDEGSQK | 9601.68 | C302 H483 N98 O103 S0 |
| Y Fragment | 0 | 7447.7052 | 7447.7304 | 49 | 114 | -3.4 | FGINAQNNQRNFLAGEEDNVISQIQRPVKEVAFPGSAEEVDRLLKNQRQSHFANAQPQQKDEGSQK | 5132.94 | C319 H506 N101 O106 S0 |
| Y Fragment | 0 | 7504.7205 | 7504.7518 | 48 | 114 | -4.2 | GFGINAQNNQRNFLAGEEDNVISQIQRPVKEVAFPGSAEEVDRLLKNQRQSHFANAQPQQKDEGSQK | 11575.18 | C321 H509 N102 O107 S0 |
| BY Int Fragment | 0 | 734.3676 | 734.3719 | 40 | 46 | -5.9 | ASSDLFL | 623.22 | C34 H52 N7 O11 S0 |
| BY Int Fragment | 0 | 771.4372 | 771.4400 | 75 | 81 | -3.6 | PVKEVAF | 922.37 | C38 H59 N8 O9 S0 |
| BY Int Fragment | 0 | 771.4372 | 771.4400 | 76 | 82 | -3.6 | VKEVAFP | 922.37 | C38 H59 N8 O9 S0 |
| BY Int Fragment | 0 | 1257.6029 | 1257.6083 | 49 | 59 | -4.3 | FGINAQNNQRN | 590.53 | C52 H81 N20 O17 S0 |
| BY Int Fragment | 0 | 1257.6029 | 1257.6083 | 50 | 60 | -4.3 | GINAQNNQRNF | 590.53 | C52 H81 N20 O17 S0 |
| BY Int Fragment | 0 | 1271.6168 | 1271.6240 | 52 | 62 | -5.6 | NAQNNQRNFLA | 622.63 | C53 H83 N20 O17 S0 |
| BY Int Fragment | 0 | 1311.5907 | 1311.5924 | 102 | 113 | -1.3 | NAQPQQKDEGSQ | 1065.18 | C52 H83 N18 O22 S0 |
| BY Int Fragment | 0 | 1461.6934 | 1461.6982 | 48 | 60 | -3.3 | GFGINAQNNQRNF | 463.18 | C63 H93 N22 O19 S0 |
| BY Int Fragment | 0 | 2577.2794 | 2577.2917 | 82 | 104 | -4.8 | PGSAEEVDRLLKNQRQSHFANAQ | 574.08 | C109 H174 N37 O36 S0 |
| BY Int Fragment | 0 | 2577.2794 | 2577.2917 | 83 | 105 | -4.8 | GSAEEVDRLLKNQRQSHFANAQP | 574.08 | C109 H174 N37 O36 S0 |
| BY Int Fragment | 0 | 4755.3228 | 4755.3438 | 24 | 68 | -4.4 | SGDVVVIPASHPFSINASSDLFLLGFGINAQNNQRNFLAGEEDNV | 568.39 | C211 H321 N58 O68 S0 |
| BY Int Fragment | 0 | 5099.4685 | 5099.4869 | 3 | 48 | -3.6 | EDEEEEEDRNVQVQRFQSKLSSGDVVVIPASHPFSINASSDLFLLG | 761.95 | C222 H345 N60 O78 S0 |
| BY Int Fragment | 0 | 5303.5570 | 5303.5768 | 3 | 50 | -3.7 | EDEEEEEDRNVQVQRFQSKLSSGDVVVIPASHPFSINASSDLFLLGFG | 2729.12 | C233 H357 N62 O80 S0 |
| BY Int Fragment | 0 | 5416.6423 | 5416.6608 | 3 | 51 | -3.4 | EDEEEEEDRNVQVQRFQSKLSSGDVVVIPASHPFSINASSDLFLLGFGI | 709.57 | C239 H368 N63 O81 S0 |
| BY Int Fragment | 0 | 5858.9189 | 5858.9413 | 35 | 88 | -3.8 | PFSINASSDLFLLGFGINAQNNQRNFLAGEEDNVISQIQRPVKEVAFPGSAEEV | 627.09 | C261 H402 N71 O83 S0 |
| BY Int Fragment | 0 | 6043.0389 | 6043.0558 | 58 | 110 | -2.8 | RNFLAGEEDNVISQIQRPVKEVAFPGSAEEVDRLLKNQRQSHFANAQPQQKDE | 2643.82 | C261 H414 N81 O85 S0 |
| BY Int Fragment | 0 | 7157.5279 | 7157.5264 | 6 | 70 | 0.2 | EEEEDRNVQVQRFQSKLSSGDVVVIPASHPFSINASSDLFLLGFGINAQNNQRNFLAGEEDNVIS | 526.27 | C312 H484 N89 O105 S0 |
| BY Int Fragment | 203.0794 | 7015.4902 | 7015.4886 | 8 | 69 | 0.2 | EEDRNVQVQRFQSKLSSGDVVVIPASHPFSINASSDLFLLGFGINAQNNQRNFLAGEEDNVI | 2540.07 | C299 H465 N86 O97 S0 |
| BY Int Fragment | 406.1588 | 3416.6620 | 3416.6758 | 36 | 63 | -4.0 | FSINASSDLFLLGFGINAQNNQRNFLAG | 7506.02 | C136 H205 N38 O40 S0 |
| BY Int Fragment | 406.1588 | 7299.6340 | 7299.6622 | 32 | 94 | -3.9 | ASHPFSINASSDLFLLGFGINAQNNQRNFLAGEEDNVISQIQRPVKEVAFPGSAEEVDRLLKN | 5347.98 | C305 H476 N87 O96 S0 |
| BY Int Fragment | 568.2116 | 4840.3237 | 4840.3225 | 34 | 72 | 0.2 | HPFSINASSDLFLLGFGINAQNNQRNFLAGEEDNVISQI | 11006.56 | C190 H288 N53 O60 S0 |
| B Fragment* | 0 | 7469.6924 | 7469.6334 | 1 | 67 | 7.9 | KKEDEEEEEDRNVQVQRFQSKLSSGDVVVIPASHPFSINASSDLFLLGFGINAQNNQRNFLAGEEDN | 569.79 | C324 H502 N93 O112 S0 |
| Y Fragment* | 0 | 3920.9124 | 3920.9386 | 80 | 114 | -6.7 | AFPGSAEEVDRLLKNQRQSHFANAQPQQKDEGSQK | 539.41 | C167 H265 N54 O57 S0 |
| Y Fragment* | 0 | 5912.9265 | 5912.9663 | 62 | 114 | -6.7 | AGEEDNVISQIQRPVKEVAFPGSAEEVDRLLKNQRQSHFANAQPQQKDEGSQK | 725.46 | C252 H406 N79 O87 S0 |
| Y Fragment* | 0 | 7282.6105 | 7282.6514 | 50 | 114 | -5.6 | GINAQNNQRNFLAGEEDNVISQIQRPVKEVAFPGSAEEVDRLLKNQRQSHFANAQPQQKDEGSQK | 743.65 | C310 H497 N100 O105 S0 |
| Y Fragment* | 0 | 7486.7109 | 7486.7412 | 48 | 114 | -4.1 | GFGINAQNNQRNFLAGEEDNVISQIQRPVKEVAFPGSAEEVDRLLKNQRQSHFANAQPQQKDEGSQK | 1221.45 | C321 H509 N102 O107 S0 |
| BY Int Fragment* | 0 | 3218.5258 | 3218.5501 | 37 | 66 | -7.6 | SINASSDLFLLGFGINAQNNQRNFLAGEED | 613.78 | C141 H215 N40 O48 S0 |
| BY Int Fragment* | 0 | 3317.5927 | 3317.6185 | 40 | 70 | -7.8 | ASSDLFLLGFGINAQNNQRNFLAGEEDNVIS | 2219.69 | C146 H224 N41 O49 S0 |
| BY Int Fragment* | 0 | 4480.1919 | 4480.2320 | 29 | 70 | -9.0 | VIPASHPFSINASSDLFLLGFGINAQNNQRNFLAGEEDNVIS | 3596.24 | C201 H306 N55 O63 S0 |
| BY Int Fragment* | 0 | 4551.2339 | 4551.2327 | 25 | 67 | 0.2 | GDVVVIPASHPFSINASSDLFLLGFGINAQNNQRNFLAGEEDN | 5732.16 | C203 H307 N56 O65 S0 |
| BY Int Fragment* | 0 | 4638.2703 | 4638.2648 | 24 | 67 | 1.2 | SGDVVVIPASHPFSINASSDLFLLGFGINAQNNQRNFLAGEEDN | 1227.47 | C206 H312 N57 O67 S0 |
| BY Int Fragment* | 0 | 5024.3891 | 5024.4548 | 3 | 47 | -13.1 | EDEEEEEDRNVQVQRFQSKLSSGDVVVIPASHPFSINASSDLFLL | 467.46 | C220 H342 N59 O77 S0 |
| BY Int Fragment* | 0 | 5099.4685 | 5099.5021 | 4 | 49 | -6.6 | DEEEEEDRNVQVQRFQSKLSSGDVVVIPASHPFSINASSDLFLLGF | 761.95 | C226 H347 N60 O76 S0 |
| BY Int Fragment* | 0 | 5285.5475 | 5285.5662 | 3 | 50 | -3.5 | EDEEEEEDRNVQVQRFQSKLSSGDVVVIPASHPFSINASSDLFLLGFG | 191.24 | C233 H357 N62 O80 S0 |
| BY Int Fragment* | 0 | 5428.7882 | 5428.8189 | 28 | 77 | -5.7 | VVIPASHPFSINASSDLFLLGFGINAQNNQRNFLAGEEDNVISQIQRPVK | 763.4 | C244 H382 N69 O73 S0 |
| BY Int Fragment* | 0 | 5858.9189 | 5858.9049 | 36 | 89 | 2.4 | FSINASSDLFLLGFGINAQNNQRNFLAGEEDNVISQIQRPVKEVAFPGSAEEVD | 627.09 | C260 H400 N71 O85 S0 |
| Y Fragment** | 0 | 3921.9208 | 3921.9226 | 80 | 114 | -0.5 | AFPGSAEEVDRLLKNQRQSHFANAQPQQKDEGSQK | 508.71 | C167 H265 N54 O57 S0 |
| Y Fragment** | 0 | 7113.5024 | 7113.5298 | 52 | 114 | -3.9 | NAQNNQRNFLAGEEDNVISQIQRPVKEVAFPGSAEEVDRLLKNQRQSHFANAQPQQKDEGSQK | 1119.15 | C302 H483 N98 O103 S0 |
| BY Int Fragment** | 0 | 1444.6691 | 1444.6716 | 48 | 60 | -1.7 | GFGINAQNNQRNF | 554.23 | C63 H93 N22 O19 S0 |
| BY Int Fragment** | 0 | 3529.7437 | 3529.7346 | 42 | 73 | 2.6 | SDLFLLGFGINAQNNQRNFLAGEEDNVISQIQ | 6601.76 | C156 H241 N44 O51 S0 |
| BY Int Fragment** | 0 | 3720.8318 | 3720.8364 | 53 | 86 | -1.3 | AQNNQRNFLAGEEDNVISQIQRPVKEVAFPGSAE | 5751.68 | C161 H254 N49 O54 S0 |
| BY Int Fragment** | 0 | 4523.2231 | 4523.2841 | 7 | 47 | -13.5 | EEEDRNVQVQRFQSKLSSGDVVVIPASHPFSINASSDLFLL | 948.33 | C201 H316 N55 O65 S0 |
| BY Int Fragment** | 0 | 4953.4025 | 4953.4177 | 4 | 48 | -3.1 | DEEEEEDRNVQVQRFQSKLSSGDVVVIPASHPFSINASSDLFLLG | 2250.6 | C217 H338 N59 O75 S0 |
| BY Int Fragment** | 0 | 5103.5925 | 5103.5711 | 29 | 75 | 4.2 | VIPASHPFSINASSDLFLLGFGINAQNNQRNFLAGEEDNVISQIQRP | 3524.23 | C228 H352 N65 O70 S0 |
| BY Int Fragment** | 0 | 5103.5925 | 5103.5711 | 30 | 76 | 4.2 | IPASHPFSINASSDLFLLGFGINAQNNQRNFLAGEEDNVISQIQRPV | 3524.23 | C228 H352 N65 O70 S0 |
| BY Int Fragment** | 0 | 5157.4818 | 5157.5076 | 4 | 50 | -5.0 | DEEEEEDRNVQVQRFQSKLSSGDVVVIPASHPFSINASSDLFLLGFG | 787.85 | C228 H350 N61 O77 S0 |
| BY Int Fragment** | 0 | 5859.9219 | 5859.8889 | 36 | 89 | 5.6 | FSINASSDLFLLGFGINAQNNQRNFLAGEEDNVISQIQRPVKEVAFPGSAEEVD | 6098.01 | C260 H400 N71 O85 S0 |
| BY Int Fragment** | 0 | 5930.9676 | 5931.0253 | 29 | 83 | -9.7 | VIPASHPFSINASSDLFLLGFGINAQNNQRNFLAGEEDNVISQIQRPVKEVAFPG | 8411.64 | C268 H413 N74 O80 S0 |
| BY Int Fragment** | 0 | 6817.3968 | 6817.4622 | 43 | 103 | -9.6 | DLFLLGFGINAQNNQRNFLAGEEDNVISQIQRPVKEVAFPGSAEEVDRLLKNQRQSHFANA | 1648.09 | C301 H471 N90 O93 S0 |
| BY Int Fragment** | 0 | 6945.4518 | 6945.5208 | 43 | 104 | -9.9 | DLFLLGFGINAQNNQRNFLAGEEDNVISQIQRPVKEVAFPGSAEEVDRLLKNQRQSHFANAQ | 2983.54 | C306 H479 N92 O95 S0 |
| BY Int Fragment** | 0 | 7504.7205 | 7504.7697 | 8 | 75 | -6.6 | EEDRNVQVQRFQSKLSSGDVVVIPASHPFSINASSDLFLLGFGINAQNNQRNFLAGEEDNVISQIQRP | 11575.18 | C329 H516 N97 O106 S0 |

1. J. Proteome Res. 2021 March; 20, 1928-1935. doi: 10.1021/acs.jproteome.0c00952. [↑](#endnote-ref-1)
2. Anal Chim Acta. 2022 February 15; 1194: 339400. doi:10.1016/j.aca.2021.339400. [↑](#endnote-ref-2)
